# Identification of diagnostic genes and drug prediction of allergic asthma by integrated bioinformatics analysis, machine learning, and molecular docking^☆^

**DOI:** 10.1016/j.waojou.2025.101147

**Published:** 2025-12-07

**Authors:** Heng Wang, Ruoyan Wang, Jie Lan, Wanfeng Zhang, Guang Li, Hui Nie, Longke Ran

**Affiliations:** a Department of Bioinformatics, The Basic Medical School of Chongqing Medical University, Chongqing, 400016, China; b Chongqing Hospital of the Affiliated Hospital of Guangzhou University of Chinese Medicine, Beibei, Chongqing, 400700, China; c Department of Radiotherapy, Chongqing University Cancer Hospital, Chongqing, 400030, China

**Keywords:** Allergic asthma, Biomarkers, Immune infiltration, Machine learning, Molecular docking

## Abstract

**Background:**

Allergic asthma is a heterogeneous inflammatory airway disease with limited biomarkers and unclear immune mechanisms, complicating diagnosis and treatment.

**Objectives:**

This study aimed to identify key genes in allergic asthma patients, assess their role in immune regulation, and screen for potential therapeutic agents.

**Methods:**

RNA was extracted from blood samples of 36 allergic asthma patients and 19 healthy controls for high-throughput sequencing. Differentially Expressed Genes (DEGs) were identified and subjected to Gene Ontology (GO) and Kyoto Encyclopedia of Genes and Genomes (KEGG) enrichment analyses. Four key genes were identified by intersecting DEGs with key modular genes from Weighted Gene Co-expression Network Analysis (WGCNA), top-ranked genes from Random Forest (RF), and significant genes from Extreme Gradient Boosting (XGBoost). Core genes were determined via Protein-protein Interaction (PPI) network analysis and further evaluated using immune infiltration and molecular docking.

**Results:**

A total of 333 DEGs were identified, mainly involved in oxygen transport and pathology. TMIGD2, OBSCN, FCGBP, and FBLN2 were screened as key genes, with OBSCN and FBLN2 designated as core genes. Immune infiltration analysis revealed associations between core genes and various immune cells, and molecular docking showed strong binding affinities of midecamycin and paricalcitol to core genes.

**Conclusions:**

This study highlights the roles of OBSCN and FBLN2 in immune regulation in allergic asthma, suggesting midecamycin and paricalcitol as potential therapeutic agents.

## Introduction

Asthma is a common chronic inflammatory airway disease that affects hundreds of millions of people worldwide, characterized by a complex and multifactorial pathogenesis.^1^^,^^2^ Typical features of asthma include airway hyperresponsiveness, airway obstruction, airway remodeling, and infiltration of various immune cells, particularly eosinophils and neutrophils.^3^ Among the various forms of asthma, allergic asthma is the most prevalent, driven by hypersensitivity to environmental allergens and characterized by a predominant Th2 immune response.^4^ Although several therapeutic approaches, such as inhaled corticosteroids and allergen immunotherapy, are available, asthma (especially allergic asthma) remains inadequately controlled in many patients, and those with severe asthma continue to experience high morbidity, mortality, and reduced quality of life.^5^^,^^6^ Therefore, identifying new biomarkers and potential therapeutic targets is of great clinical significance.

In recent years, advances in high-throughput sequencing technology have provided new insights into the molecular pathology of allergic asthma through gene expression profiling.^7^ RNA Sequencing (RNA-seq) and the analysis of Differentially Expressed Genes (DEGs) allow for a better understanding of gene expression differences between allergic asthma patients and healthy individuals.^8^ Furthermore, the application of the Weighted Gene Co-expression Network Analysis (WGCNA) method enables the systematic exploration of allergic asthma-related gene modules and their functional associations, thereby facilitating a more comprehensive deciphering of gene interactions.^9^ On this basis, the introduction of machine learning algorithms offers a powerful tool for biomarker screening. Machine learning can process complex, high-dimensional data and automatically identify disease-related features, thereby improving the efficiency and accuracy of biomarker discovery.^10^ However, there are still few studies that combine bioinformatics methods with machine learning algorithms for allergic asthma biomarker screening.

The aim of this study is to identify core genes associated with allergic asthma by integrating bioinformatics analyses and machine learning algorithms, to explore their roles in its pathogenesis, and to propose potential drug targets, thereby providing a theoretical basis for personalized treatment of allergic asthma.

## Methods

### Sample collection

Blood samples from 36 allergic asthma patients and 19 healthy controls were collected from May 9, 2024 to May 24, 2024 at Chongqing Hospital of The First Affiliated Hospital of Guangzhou University of Chinese Medicine (Chongqing Beibei Hospital of Traditional Chinese Medicine). The study was approved by the Ethics Committee of Chongqing Beibei Hospital of Traditional Chinese Medicine (BBQZYYEC-2021-0228) and strictly adhered to the ethical guidelines outlined in the Declaration of Helsinki.

### RNA-sequencing and data processing

Blood samples from all patients were collected with PAXgene Blood RNA Tubes (BRT, PreAnalytiX; cat. no. 762165), PAXgene Blood RNA kit for isolation of total RNA from whole blood. High-throughput sequencing was performed by DNBSEQ-T7 sequencer (BGI, Shenzhen, China) according to the manufacturer's recommendations. The sequencing process was carried out by Chongqing Aar Biotechnology Co.

The raw data were subjected to quality control and filtering to retain high-quality, clean reads. We calculated Q20, Q30, and GC content to ensure the high accuracy of the data. Subsequent analyses were performed based on the high-quality clean reads. The reference genome (Homo sapiens GRCh38) and annotation files were downloaded from publicly available databases. HISAT2 was utilized to construct the reference genome index and align the paired-end clean reads to the reference genome.^11^ Read counts for each gene were calculated using featureCounts.^12^

### Identification of DEGs

First, genes with expression levels greater than 0 in at least 50% of the samples were selected for differential analysis. DEGs were identified using 3 methods: the DESeq2 R package (version 1.42.1), the edgeR R package (version 4.0.16), and the Limma R package (version 3.58.1). Genes with |log2(*FC*)| ≥ 0.5 and P < 0.05 were considered significantly different in expression levels.

### GO and KEGG analyses

The clusterProfiler package (version 4.10.1) was used to perform Gene Ontology (GO) enrichment and Kyoto Encyclopedia of Genes and Genomes (KEGG) pathway analyses to systematically investigate gene functions and key biological pathways associated with allergic asthma. Pathways with p < 0.05 were considered statistically significant. All results were visualized using the ggplot2 package in R.

### Weighted gene co-expression network analysis

To identify genes that are highly associated with the phenotype, we screened for those ranked in the top 75% by median absolute deviation (MAD) before constructing a gene co-expression network using the R package WGCNA. This approach allowed us to determine the modules most strongly associated with allergic asthma and identify all the characteristic genes within them.

### Machine learning identifies pivotal genes

DEGs obtained from differential expression analysis were used as input data. Random Forest (RF) and Extreme Gradient Boosting (XGBoost) algorithms were employed to assess gene significance and identify key pivotal genes associated with allergic asthma.

### Construction of protein-protein interaction networks

Protein-protein Interaction (PPI) networks were constructed using the GeneMANIA web platform (https://genemania.org/) to explore the interactions and biological functions of core genes. GeneMANIA facilitates the analysis of the roles of core genes in PPI networks by predicting gene functions and identifying functionally similar genes.^13^

### GSEA for individual core genes

Allergic asthma patients were categorized into high and low expression groups based on the median expression values of the core genes. The clusterProfiler package (version 4.10.1) performs gene set enrichment analysis (GSEA) on core genes. A *p*-value <0.05 was considered statistically significant.

### Immune cell abundance

The CIBERSORT algorithm was used to analyze the relative abundance of different immune cells in each allergic asthma sample. Based on the LM22 gene feature matrix from the CIBERSORT website (http://cibersort.stanford.edu/), CIBERSORT back-convolution analysis facilitated the resolution of immune cell composition in the samples.^14^

### Screening and molecular docking of small molecule drugs

Potential small-molecule drugs associated with the core genes were identified through enrichment analysis using the Enrichr platform (https://maayanlab.cloud/Enrichr/). The “Disease/Drug” module of the DSigDB database was utilized to screen small molecules potentially related to allergic asthma. Only compounds with a statistically significant *p*-value <0.05 were considered potential drug candidates targeting the core genes. DSigDB (https://maayanlab.cloud/DSigDB/)^15^ is a comprehensive database that integrates includes information on drugs, genes, and diseases derived from published literature and experimental evidence. Structures of small molecule drugs were obtained from PubChem (https://pubchem.ncbi.nlm.nih.gov/). The structures of core proteins were retrieved from the RCSB Protein Data Bank (PDB, http://www.rcsb.org/) and AlphaFold (https://alphafold.ebi.ac.uk/). Protein structures were preprocessed using PyMOL (version 2.6) by removing water molecules and non-essential cofactors.^16^ For molecular docking preparation, AutoDock Tools (version 1.5.7) was used to prepare both protein and ligand structures. This process included the addition of hydrogen atoms, calculation of molecular charges, and specification of rotatable bonds for the ligands. Since no previous studies have directly reported interactions between these protein and ligand, a GridBox was generated to encompass the entire protein structure, ensuring a broad search for potential binding sites. Molecular docking was performed using AutoDock 4.2.6 with Genetic Algorithm Parameters.^17^ The minimum binding free energy during ligand-protein interactions is used to evaluate the stability and binding affinity of the ligand-protein complex. Finally, the drug-protein binding targets were visualized using PyMOL.

### Statistical analysis

R software (version 4.3.3) was used to perform all statistical analyses and visualizations. Categorical variables were described as numerical values (percentages), and comparisons between groups were conducted using the chi-square test. Continuous variables were expressed as mean ± standard deviation if normally distributed or as median and Interquartile Range (IQR) if skewed. Spearman correlation analysis was performed to assess the correlation between core genes and immune cell abundance. A *p*-value <0.05 was considered statistically significant.

## Results

### Study design and participants

Supplementary Fig. 1 shows the workflow of this study. The present study included 36 allergic asthmatic patients, of whom 24 were females (66.7%) and 12 were males (33.3%); additionally, 19 controls were included, of whom 16 were females (84.2%) and 3 were males (15.8%). The median ages of the 2 groups were 49.5 years [41.0; 59.2] for allergic asthmatics and 26.0 years [25.0; 28.0] for controls (Table 1).

**Table 1:** Patient characteristics

	Asthma N = 36	Control N = 19	p.value
Sex			0.284
female	24 (66.7%)	16 (84.2%)	
man	12 (33.3%)	3 (15.8%)	
Age	49.5 [41.0; 59.2]	26.0 [25.0; 28.0]	<0.001

### Identification of DEGs

**Fig. 1:**
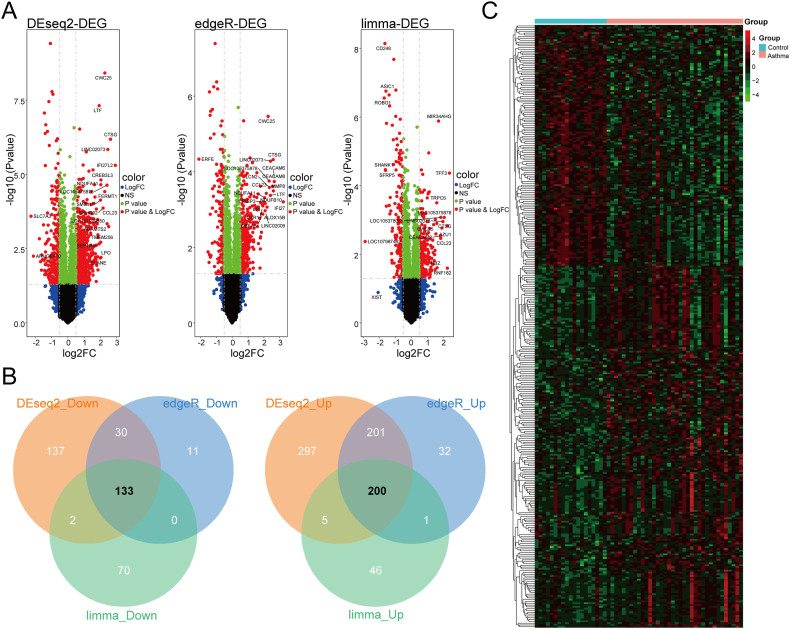
Identification of DEGs. (A) The volcano plot of DEGs. Red dots indicate DEGs with |log (*FC*)| > 0.5 and *p*-value <0.05; blue dots indicate genes with |log (*FC*)| > 0.5 and *p*-value >0.05; green dots indicate genes with |log (*FC*)| < 0.5 and *p*-value <0.05; and black dots indicate genes with |log (*FC*)| < 0.5 and *p-*value >0.05. (B) Three Venn plots of the differential analysis results. Orange circles represent the DESeq2 package, blue circles represent the edgeR package, and green circles represent the limma package. (C) Heatmap of differentially expressed genes that were significant in all 3 differential analysis packages, with red indicating up-regulation and green indicating down-regulation

Based on the screening criteria of |log2(*FC*)| > 0.5 and *p*-value <0.05, we identified 1005 DEGs using the R package DESeq2, of which 703 genes were up-regulated and 302 genes were down-regulated in allergic asthma. Additionally, we identified 782 DEGs using the R package edgeR, of which 608 genes were up-regulated and 174 genes were down-regulated in allergic asthma. We also identified 457 DEGs using the R package limma, with 252 genes up-regulated and 205 genes down-regulated in allergic asthma. Supplementary Tables 1, 2, and 3 contain a detailed list of DEGs. Heatmaps were utilized to describe the differences (Fig. 1A). Venn plots of the intersection of the results from the 3 differential analyses revealed 133 genes that were down-regulated and 200 genes that were up-regulated in allergic asthma (Fig. 1B). A heatmap was utilized to visualize the 333 DEGs (Fig. 1C).

### Pathway enrichment

**Fig. 2:**
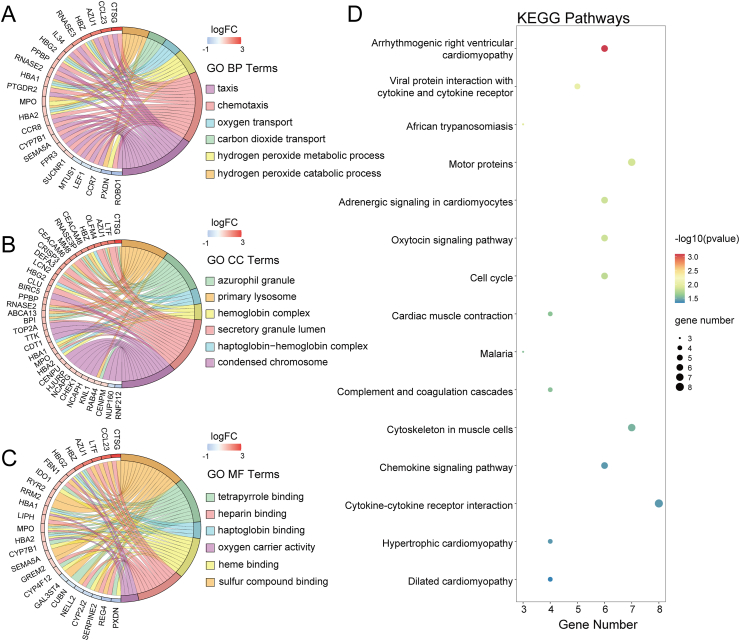
Functional enrichment analysis of DEGs. (A) GO enrichment analysis of BP. (B) GO enrichment analysis of CC. (C) GO enrichment analysis of MF. (D) KEGG enrichment analysis, with bubble plots showing 15 significant enrichment pathways (p < 0.05)

To elucidate the potential mechanisms of DEGs in allergic asthma, we performed GO and KEGG enrichment analyses. GO enrichment analysis showed that the top 5 pathways enriched in Biological Process (BP) included hydrogen peroxide catabolic process, carbon dioxide transport, oxygen transport, hydrogen peroxide metabolic process, chemotaxis, and taxis (Fig. 2A). In Cellular Components (CC), the top 5 items are enriched in primary lysosome, azurophil granule, haptoglobin-hemoglobin complex, hemoglobin complex, and secretory granule lumen (Fig. 2B). The top 5 items enriched in Molecular Function (MF) included sulfur compound binding, tetrapyrrole binding, haptoglobin binding, heme binding, and heparin binding (Fig. 2C). Additionally, KEGG pathway analysis revealed that these DEGs were significantly enriched in Arrhythmogenic right ventricular cardiomyopathy, Viral protein interaction with cytokine and cytokine receptor, African trypanosomiasis, Motor proteins, and Adrenergic signaling in cardiomyocytes (Fig. 2D). Supplementary Tables 4 and 5 present detailed results of GO and KEGG enrichment analyses of DEGs.

### Identification of key modular genes by WGCNA

**Fig. 3:**
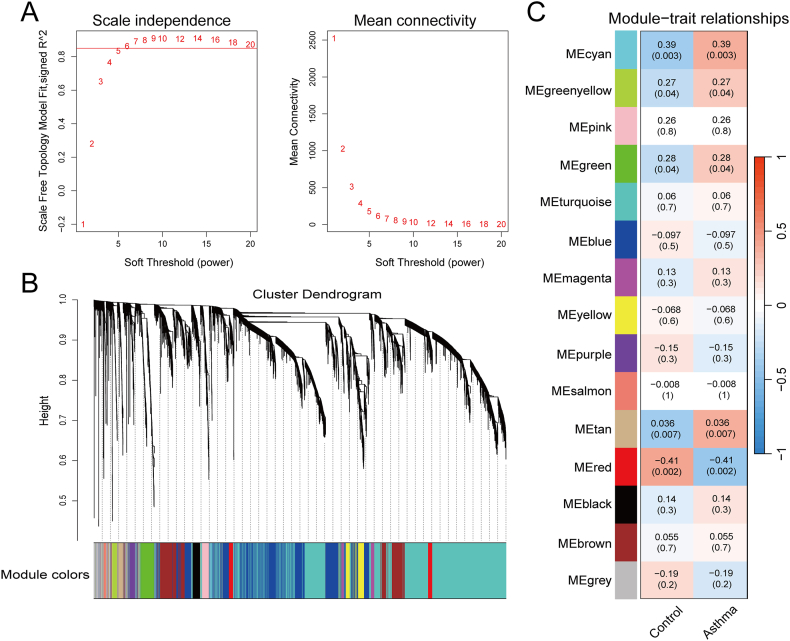
WGCNA Analysis. (A) Determination of soft thresholds. (B) Clustering Dendrogram: Each branch of the dendrogram represents a gene, while the colors below indicate different modules. (C) Heatmap Depicting Correlations Between Modules and Traits: Each color represents a specific module, with the accompanying values denoting module-trait correlation coefficients and corresponding *p*-values. The analysis indicates that the red module exhibits the highest correlation with allergic asthma

To identify disease-related module genes, we performed WGCNA. A value of 6 was chosen as the optimal soft threshold (R^2^ = 0.85) to build the scale-free network (Fig. 3A). Cluster analysis was conducted to identify highly similar modules, with the minimum module size set to 30. In total, we identified 15 modules, each corresponding to a different color (Fig. 3B). The correlation between modules and traits was calculated using the Pearson correlation coefficient (Fig. 3C). The red module, which exhibited the highest positive correlation, and the module with the highest negative correlation were considered the hub modules (cor = 0.41; p = 0.002). Results for the module genes are provided in Supplementary Table 6.

### Machine learning identifies pivotal genes

**Fig. 4:**
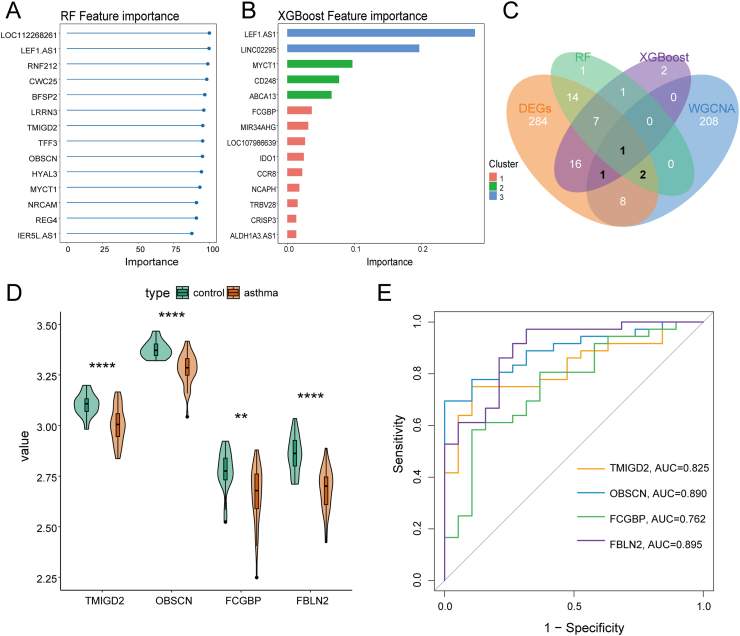
Identification of Key Genes Using Machine Learning Algorithms. (A) Top 14 Genes Identified by the RF Algorithm. (B) Significant Genes Ranked by the XGBoost Algorithm. (C) Venn Plots Illustrating the Intersection of Genes Among the Two Algorithms, Modular Genes, and DEGs. (D) Differential Analysis of Expression Levels for the Four Key Genes. (E) ROC curves demonstrating the diagnostic efficacy of the 4 key genes. ∗∗P < 0.01; ∗∗∗P < 0.001; ∗∗P < 0.0001

To further identify candidate diagnostic gene targets with significant feature values for classifying allergic asthma and control groups, DEGs obtained from the 3 differential analysis packages mentioned above were analyzed using 2 algorithms: RF and XGBoost. The top 26 genes identified by the RF algorithm, significant genes from the XGBoost algorithm, modular genes, and DEGs were intersected using a Venn diagram based on feature importance (Fig. 4A and B), resulting in the identification of 4 key genes: TMIGD2, OBSCN, FCGBP, and FBLN2 (Fig. 4C). To visually represent the expression levels of these genes, violin plots were generated, demonstrating a decreasing trend for these 4 genes in allergic asthma samples compared to the control group (Fig. 4D). Further evaluation using ROC curves revealed higher AUC values for TMIGD2, OBSCN, FCGBP, and FBLN2, with respective values of 0.825, 0.890, 0.762, and 0.895 (Fig. 4E). Based on the aforementioned findings, we conclude that these 4 key genes hold significant potential as diagnostic biomarkers for allergic asthma.

### Protein-protein interaction networks identify core genes

**Fig. 5:**
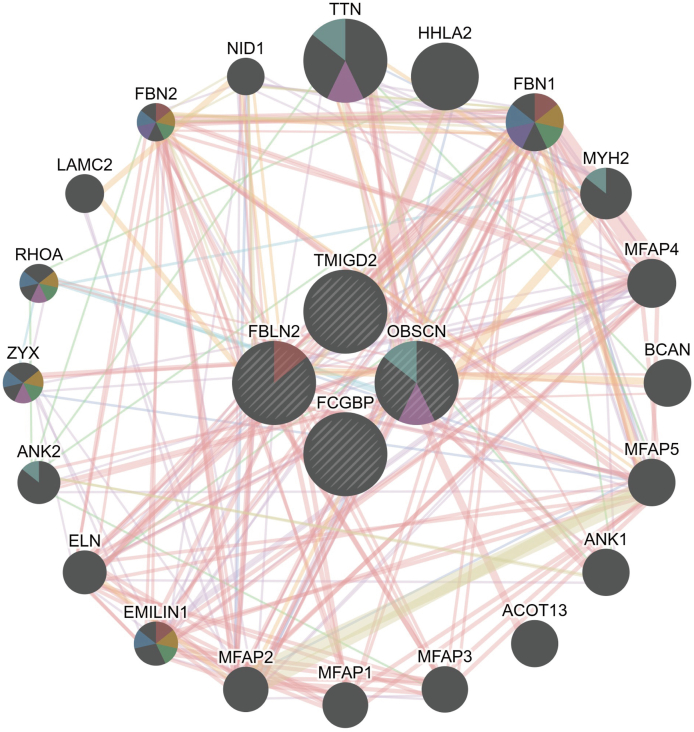
Analysis of PPI Network. PPI Network of TMIGD2, OBSCN, FCGBP, and FBLN2 Proteins

Using the GeneMANIA database, we analyzed a PPI network that includes 4 core genes (TMIGD2, OBSCN, FCGBP, and FBLN2) and their interacting partners (Fig. 5). In this network, the core genes are depicted as circles at the center. Surrounding these core genes are 20 additional circles representing genes closely associated with them, identified based on physical interactions, co-expression, predicted interactions, co-localization, and genetic interactions. FBLN2 and OBSCN were identified as the final core genes for this study through a comprehensive analysis of the PPI network.

### GSEA for core genes

**Fig. 6:**
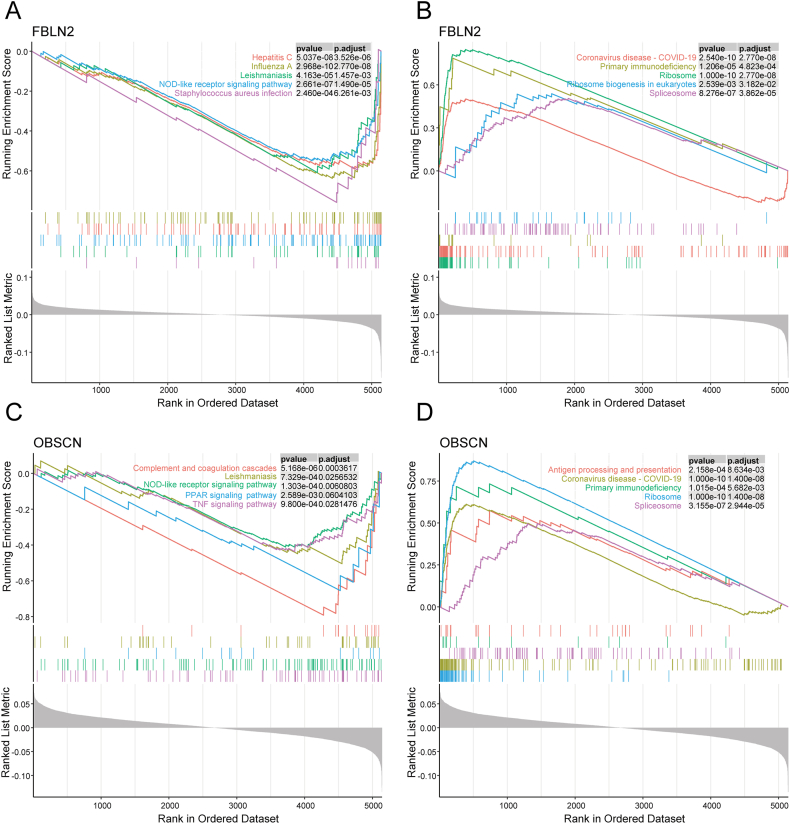
GSEA of FBLN2 and OBSCN. (A) GSEA-KEGG of the low FBLN2 expression allergic asthma group. (B) GSEA-KEGG of the high FBLN2 expression allergic asthma group. (C) GSEA-KEGG of the low OBSCN expression allergic asthma group. (D) GSEA-KEGG of the high OBSCN expression allergic asthma group

To further elucidate the roles of FBLN2 and OBSCN in allergic asthma, we conducted single-gene GSEA for each of the 2 core genes and visualized the top 5 upregulated and downregulated pathways. The GSEA results indicated that the gene set in the low FBLN2 expression allergic asthma group was primarily associated with Influenza A, Hepatitis C, NOD-like receptor signaling pathway, Leishmaniasis, and Measles (Fig. 6A). In contrast, the gene set in the high FBLN2 expression group was primarily associated with Ribosome, Coronavirus disease - COVID-19, Spliceosome, Primary immunodeficiency, and Oxidative phosphorylation (Fig. 6B). The gene set in the low OBSCN expression allergic asthma group was primarily associated with Complement and coagulation cascades, NOD-like receptor signaling pathway, Leishmaniasis, TNF signaling pathway, and Neutrophil extracellular trap formation (Fig. 6C). Conversely, the gene set in the high OBSCN expression group was primarily associated with Ribosome, Coronavirus disease - COVID-19, Spliceosome, Primary immunodeficiency, and Antigen processing and presentation (Fig. 6D). Supplementary Tables 7 and 8 present the GSEA results for the core genes.

### Immune infiltration analysis

The results of the GSEA suggest that immune function is critical for the development of allergic asthma. CIBERSORT analysis was conducted to assess the differences in immune cell infiltration between the allergic asthma and control groups. The proportions of 22 immune cell types are depicted as a bar graph for each group (Supplementary Fig. 2A). Compared to the control group, the allergic asthma group exhibited a higher abundance of resting NK cells, whereas the proportions of naive CD4 T cells and activated NK cells were lower (Supplementary Fig. 2B). The heat map illustrates the correlation among these immune cell types (Supplementary Fig. 2C).

**Fig. 7:**
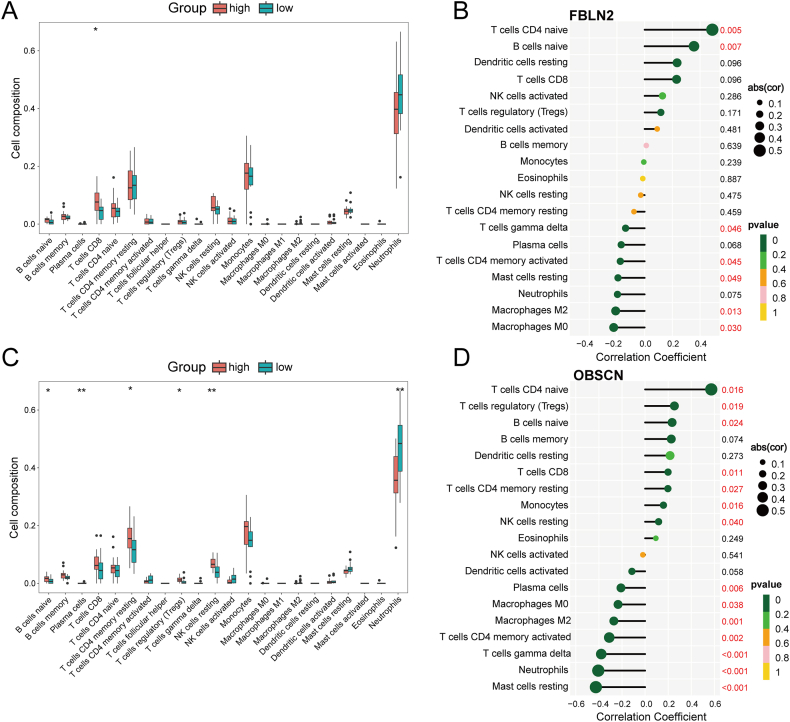
Correlation between FBLN2, OBSCN, and infiltrating immune cells. (A) Comparison of immune infiltration between the high and low FBLN2 expression groups within the allergic asthma cohort. (B) Correlation between immune infiltrating cells and FBLN2 expression levels. (C) Comparison of immune infiltration between the high and low OBSCN expression groups within the allergic asthma cohort. (D) Correlation between immune infiltrating cells and OBSCN expression levels

The relationship between core genes and immune cells was further investigated. Allergic asthma patients with high FBLN2 expression exhibited a higher proportion of CD8 T cells compared to those with low FBLN2 expression (Fig. 7A). Correlation analysis revealed a negative correlation between FBLN2 expression and gamma delta T cells, activated CD4 memory T cells, resting mast cells, M2 macrophages, and M0 macrophages. Conversely, a positive correlation was observed with naive CD4 T cells and naive B cells (Fig. 7B).

In patients with high OBSCN expression, the proportions of naive B cells, resting CD4 memory T cells, regulatory T cells (Tregs), and resting NK cells were higher than in those with low OBSCN expression. Conversely, the proportions of plasma cells and neutrophils were elevated in the low expression group (Fig. 7C). Correlation analysis indicated that OBSCN was positively correlated with naive CD4 T cells, regulatory T cells (Tregs), naive B cells, CD8 T cells, resting CD4 memory T cells, monocytes, and resting NK cells. In contrast, no significant correlation was found with plasma cells, M0 macrophages, M2 macrophages, activated CD4 memory T cells, gamma delta T cells, neutrophils, or resting mast cells, which showed negative correlations (Fig. 7D).

### Identification of small molecule drugs and molecular docking

**Fig. 8:**
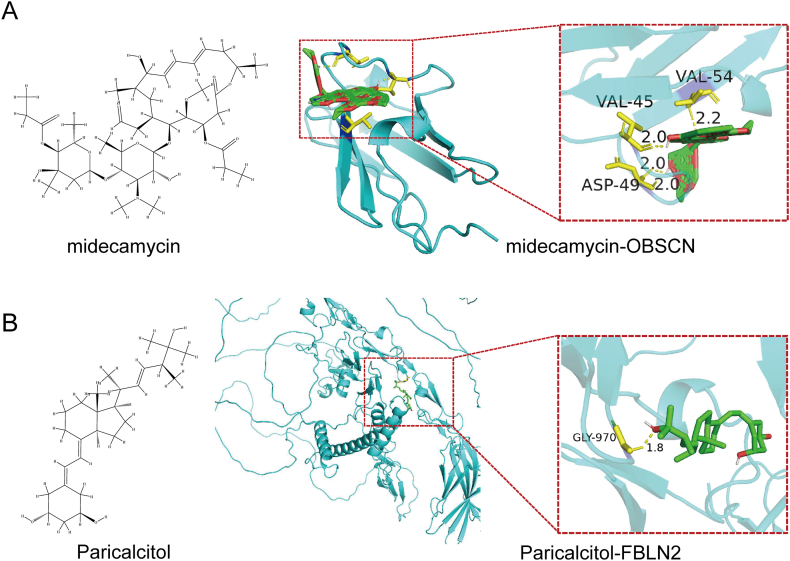
Docking diagrams of potential small molecule drugs with their targets. (A) Chemical structure of midecamycin (left) and the structure of the complex formed by docking midecamycin with OBSCN (right). (B) Chemical structure of paricalcitol (left) and the structure of the complex formed by docking paricalcitol with FBLN2 (right). The proteins are shown in cyan, the small molecule drugs in green, and the yellow dashed lines represent hydrogen bonds

To predict small-molecule drugs with potential therapeutic effects for allergic asthma, the core genes were entered into the Enrichr platform and analyzed using the DSigDB database under the “Diseases/Drugs” module. Based on statistically significant *p*-values, 3 small-molecule candidates were identified as potentially associated with the core genes: midecamycin (PubChem CID: 5282169), PHOSPHINE (PubChem CID: 24404), and paricalcitol (PubChem CID: 5281104). Among these, midecamycin was paired with OBSCN, whereas paricalcitol and PHOSPHINE were associated with FBLN2. The 3D structure of OBSCN was obtained from the RCSB Protein Data Bank (PDB ID: 5TZM), and the structure of FBLN2 was retrieved from AlphaFold3 (UniProt ID: P98095), which provides high-confidence predicted protein models. Candidate protein-ligand pairs were selected not only based on Enrichr *p*-value rankings but also via a literature review. Notably, no direct evidence was found in existing studies indicating specific binding or pharmacological activity of midecamycin, paricalcitol, or PHOSPHINE with either OBSCN or FBLN2. However, data from the DSigDB indicate that paricalcitol, when combined with phosphorus, may upregulate FBLN2 mRNA expression,^18^ indicating a potential indirect regulatory relationship. Molecular docking simulations were conducted performed using AutoDock Tools 1.5.7 to evaluate the binding affinities between the selected protein-ligand pairs (Fig. 8A and B). Binding energy values (ΔG) were used to estimate interaction strength; lower binding energy indicating stronger binding potential. A threshold of −5.0 kcal/mol was used as a cutoff to indicate biologically meaningful interactions. Importantly, PHOSPHINE was excluded from molecular docking with FBLN2, as its predicted binding energy was only −1.8 kcal/mol, which did not meet the threshold. Furthermore, PHOSPHINE lacks a defined binding pocket on FBLN2, and its extremely small molecular size, inorganic gaseous nature, and high toxicity render it unsuitable as a drug candidate.

The binding affinity between the OBSCN protein and midecamycin was −17.99 kcal/mol, while that between the FBLN2 protein and paricalcitol was −5.20 kcal/mol (Table 2), both suggesting favorable interactions. These findings indicate that midecamycin and paricalcitol may represent promising therapeutic leads targeting asthma-related genes and support the utility of computational screening in early-stage drug discovery.

**Table 2:** Results of potential small molecule drug-target docking

Drug	Target	P value	Binding energy (kcal/mol)
Midecamycin	OBSCN	0.017522856	−17.99
Paricalcitol	FBLN2	0.005691947	−5.20

## Discussion

In this study, we screened the DEGs between allergic asthma patients and healthy controls using a bioinformatics approach and ultimately identified 2 core genes, OBSCN and FBLN2, by integrating WGCNA, machine learning, and PPI network analysis. Additionally, immune infiltration analyses elucidated the roles of these 2 core genes in immune processes, further substantiating their significance in the pathogenesis of allergic asthma. Finally, potential drug interactions between OBSCN and midecamycin, as well as between FBLN2 and paricalcitol, were explored through molecular docking analysis, offering new insights for the precision treatment of allergic asthma.

We conducted GO and KEGG analyses to investigate the functions of DEGs in allergic asthma in greater detail. The results of the enrichment analyses indicated that DEGs were significantly enriched in biological processes associated with oxygen transport, hydrogen peroxide metabolism, and inflammatory responses. The significance of these pathways suggests that the pathophysiological mechanisms of allergic asthma may be closely linked to oxidative stress and immune regulation, further supporting the concept of allergic asthma as a chronic inflammatory disease.^19^^,^^20^ Previous studies have demonstrated that oxidative stress plays a crucial role in allergic asthma exacerbations, resulting in increased airway inflammation.^21^ Moreover, research has indicated a correlation between disturbances in hydrogen peroxide metabolism and the severity of allergic asthma, and our findings align with these results, underscoring the potential role of oxidative stress in allergic asthma.^22^ Our findings further corroborate the critical role of oxidative stress and immunomodulation in the pathological processes of allergic asthma, providing new evidence for future therapeutic strategies.

Through WGCNA, machine learning, and PPI network analysis, we identified 2 core genes: OBSCN and FBLN2. Obscurins, encoded by the OBSCN gene, are cytoskeletal proteins that play a crucial role in the structural and functional maintenance of muscle cells.^23^ Recent studies have indicated that OBSCN may be associated with various diseases, including rhabdomyolysis and hypertrophic cardiomyopathy.^24^^,^^25^ In this study, we found that OBSCN was significantly down-regulated in allergic asthma patients and strongly associated with immune cell infiltration, particularly correlating with the expression of regulatory T cells (Tregs) and macrophages. These findings suggest that OBSCN may influence the pathological processes of allergic asthma by modulating the immune response in the airways. FBLN2 (fibulin-2) is an extracellular matrix protein involved in various biological processes, including cell adhesion, migration, and matrix remodeling.^26^ CIBERSORT analyses further revealed differences in immune cell infiltration associated with FBLN2 expression in allergic asthma patients, particularly in CD8 T cells. These differences in immune cell subsets may be closely related to the chronic inflammatory state of allergic asthma. The expression levels of OBSCN and FBLN2 exhibited significant positive and negative correlations with the proportions of various immune cells, further confirming their potential role in allergic asthma through modulation of the immune microenvironment.

As discussed above, we identified paricalcitol and midecamycin as potential therapeutic candidates for allergic asthma through drug prediction and molecular docking analyses. These 2 drugs may have distinct roles in the pathophysiological mechanisms underlying allergic asthma. Paricalcitol is a synthetic derivative of vitamin D2 primarily used for treating secondary hyperparathyroidism in patients with chronic kidney disease.^27^ Studies have demonstrated that vitamin D plays a significant role in regulating the immune response, anti-inflammation, and promoting lung function.^28^ Specifically, paricalcitol can attenuate the immune system's overreaction by inhibiting T cell activation and promoting the production of regulatory T cells,^29^ which is crucial for managing the chronic inflammatory state of allergic asthma. In our study, FBLN2 demonstrated a high binding affinity for paricalcitol, indicating that this drug may alleviate allergic asthma symptoms by modulating FBLN2-associated immune pathways. Conversely, midecamycin is a macrolide antibiotic primarily used for treating bacterial infections.^30^ Midecamycin has been found to exert immunomodulatory and anti-inflammatory effects by regulating the expression of certain pro-inflammatory cytokines,^31^ which may help alleviate symptoms in allergic asthma patients. Additionally, the binding energy of midecamycin to OBSCN was −17.99 kcal/mol, indicating a strong molecular interaction, suggesting that it may influence the pathogenesis of allergic asthma by modulating OBSCN-related signaling pathways. While these 2 drugs show promising potential for allergic asthma treatment, further experimental studies are required to confirm their specific mechanisms of action and clinical efficacy. Future studies should consider conducting randomized controlled trials to evaluate the practical effects of paricalcitol and midecamycin in allergic asthma patients, as well as to explore their applicability in different allergic asthma subtypes.

While this study comprehensively analyzed allergic asthma-associated core genes using various bioinformatics tools and proposed potential therapeutic targets, several limitations remain. Firstly, the relatively small sample size may limit the generalizability of the results; secondly, although molecular docking analyses indicated the potential of small molecule drugs, their actual therapeutic effects require further experimental validation. Future studies should integrate larger-scale clinical data along with in vivo and ex vivo experiments to further elucidate the mechanisms of action of these key genes and drug targets.

## Conclusion

In this study, we identified FBLN2 and OBSCN as core genes in allergic asthma through bioinformatics analysis, machine learning, and molecular docking. Additionally, we predicted potential molecules targeting FBLN2 and OBSCN using the Enrichr platform. Our findings suggest that FBLN2 and OBSCN could serve as potential biomarkers and therapeutic targets for allergic asthma, highlighting their potential in its diagnosis and treatment.

## Abbreviations

RNA-seq, RNA Sequencing; DEGs, Differentially Expressed Genes; WGCNA, Weighted Gene Co-expression Network Analysis; GO, Gene Ontology; KEGG, Kyoto Encyclopedia of Genes and Genomes; MAD, Median Absolute Deviation; RF, Random Forest; XGBoost, Extreme Gradient Boosting; PPI, Protein-protein Interaction; GSEA, Gene Set Enrichment Analysis; PDB, Protein Data Bank; IQR, Interquartile Range; BP, Biological Process; CC, Cellular Components; MF, Molecular Function.

## Data availability statement

All data are included in the article. Request for the dataset supporting our results can be made to the corresponding author and will be given after approval.

## Author contributions

Heng Wang: formal analysis, statistical analysis and writing - original draft, and visualization; Ruoyan Wang and Guang Li: investigation and data curation; Jie Lan and Wanfeng Zhang: writing - review and editing; Hui Nie and Longke Ran: conceptualization, methodology, supervision, project administration, funding acquisition, and writing - review and editing. All authors have reviewed and approved the final version of the manuscript.

## Ethics statement

This study was approved by the Ethics Committee of the Chongqing Hospital of The First Affiliated Hospital of Guangzhou University of Chinese Medicine (Chongqing Beibei Hospital of Traditional Chinese Medicine) (BBQZYYEC-2021-0228). All participants gave informed consent before participation.

## Declaration of Generative AI and AI-assisted technologies in the writing process

Generative artificial intelligence (AI) and AI-assisted technologies were not used in any stage of the preparation of this manuscript.

## Funding

This study was supported by the Program for Youth Innovation in Future Medicine, Chongqing Medical University (grant no. W0102); Chongqing Science and Health Joint Scientific Research Project on Traditional Chinese Medicine (2021ZY024259); Chongqing medical scientific research project (Joint project of Chongqing Health Commission and Science and Technology Bureau) (2022MSXM165).

## Declaration of competing interest

All authors report no conflict of interest.

## Footnotes

Appendix A Supplementary data to this article can be found online at https://doi.org/10.1016/j.waojou.2025.101147.
