# Supplementary material for: Identification of diagnostic genes and drug prediction of allergic asthma by integrated bioinformatics analysis, machine learning, and molecular docking
Source: World Allergy Organ J. 2025 Dec 7;18(12):101147. doi: 10.1016/j.waojou.2025.101147 (PMC12741285; doi:10.1016/j.waojou.2025.101147)
Supplement: Multimedia component 1 [file mmc1.docx]

**Identification of diagnostic genes and drug prediction of allergic asthma by integrated bioinformatics analysis, machine learning, and molecular docking**


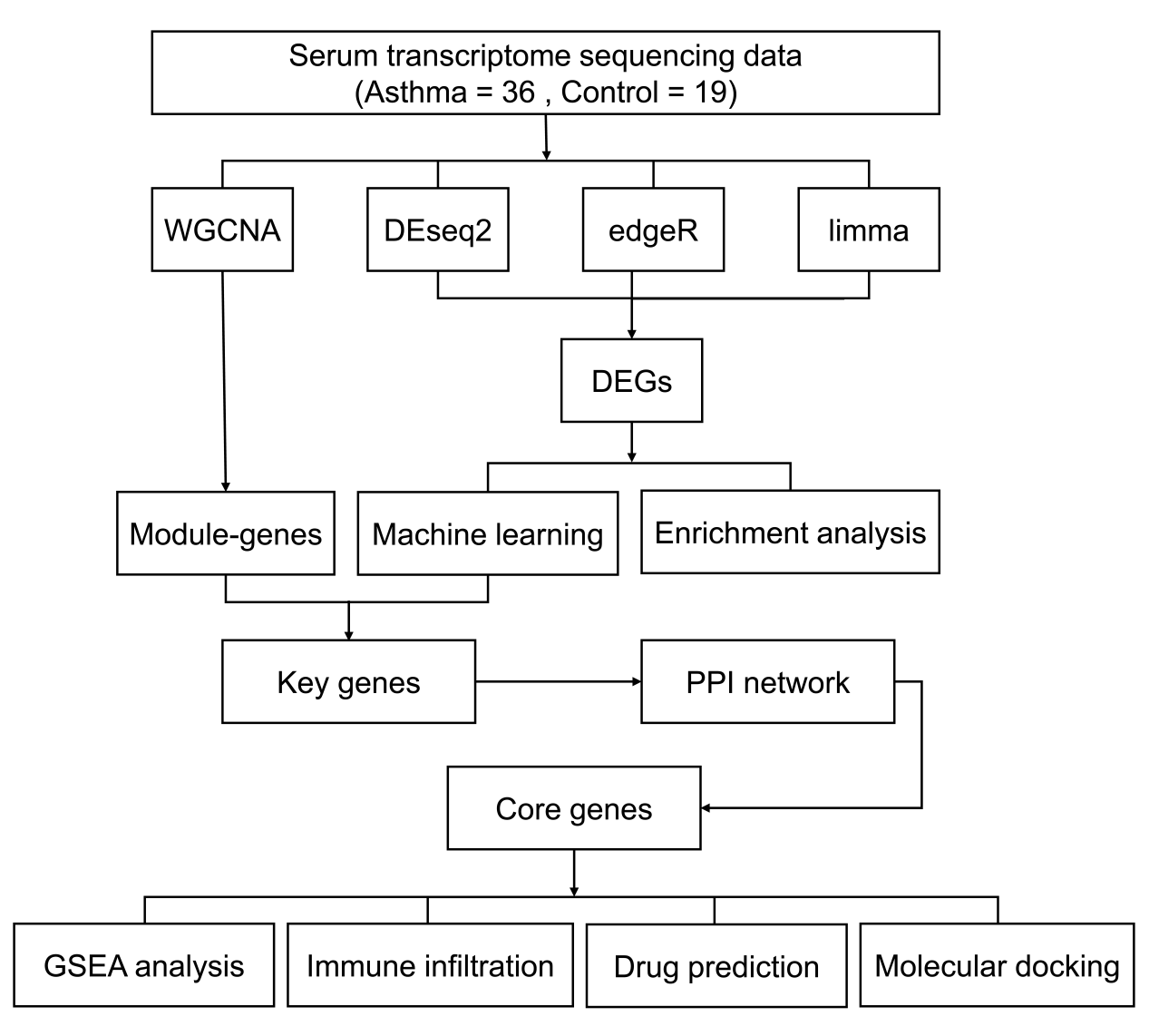


**Supplementary Fig. 1** The flow chart of this study.


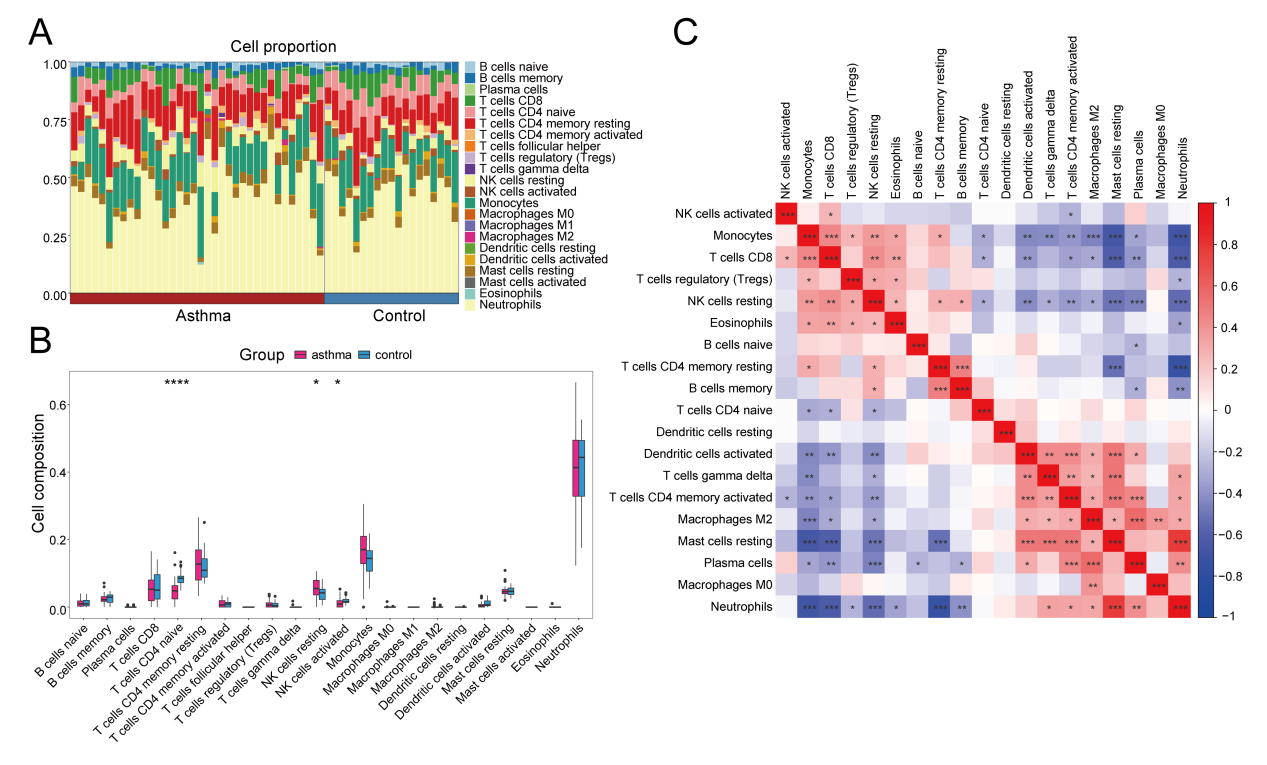


**Supplementary** **Fig. 2** Immune Cell Infiltration Analysis. (A) Bar graph depicting the composition of 22 immune cell types. (B) Differences in immune cell infiltration between the allergic asthma and control groups. (C) Correlation heatmap of the 22 immune cell types. Red and blue colors represent positive and negative correlations, respectively. Darker rectangles indicate a stronger correlation coefficient. *P < 0.05, **P < 0.01, ***P < 0.001, **P < 0.001.

| **Supplementary Table 1: DEGs from DEseq2** | | | | | | |
| --- | --- | --- | --- | --- | --- | --- |
| symbol | baseMean | log2FoldChange | lfcSE | stat | pvalue | padj |
| IFI27L2 | 19.30424217 | 2.926109369 | 0.640636877 | 4.567500673 | 4.94E-06 | 0.003971912 |
| CTSG | 74.75130837 | 2.626649851 | 0.528037807 | 4.974359442 | 6.55E-07 | 0.000921729 |
| ADAMTS2 | 5.811549804 | 2.516123258 | 0.730530949 | 3.444239102 | 0.000572669 | 0.047630598 |
| LINC02073 | 14.64124765 | 2.46435061 | 0.51144165 | 4.818439424 | 1.45E-06 | 0.001617917 |
| LOC101929950 | 6.984120793 | 2.418950248 | 0.69039869 | 3.503700519 | 0.000458841 | 0.042865758 |
| LOC105375878 | 16.16830387 | 2.411037811 | 0.569539774 | 4.233308932 | 2.30E-05 | 0.00965795 |
| CWC25 | 61.88521984 | 2.278406671 | 0.386807772 | 5.890281518 | 3.86E-09 | 3.80E-05 |
| FERMT1 | 3.127759873 | 2.273052931 | 0.551721385 | 4.119928994 | 3.79E-05 | 0.01061992 |
| SLC7A3 | 4.644550161 | -2.268461154 | 0.620364842 | -3.656656535 | 0.000255526 | 0.032713737 |
| CCL23 | 11.3153495 | 2.171598127 | 0.569221299 | 3.815033149 | 0.000136165 | 0.024195705 |
| SNHG32 | 16.95093802 | 2.169396295 | 0.568511459 | 3.815923606 | 0.000135674 | 0.024195705 |
| ARHGEF10 | 13.13696861 | -2.116729785 | 0.764660179 | -2.768196702 | 0.005636742 | 0.156364456 |
| LPO | 2.498875589 | 2.043855017 | 0.747337696 | 2.734848018 | 0.006240908 | NA |
| CREB3L3 | 5.955269768 | 2.038764242 | 0.471615072 | 4.322941233 | 1.54E-05 | 0.007986615 |
| NDUFA11 | 36.06430476 | 2.029412828 | 0.478524284 | 4.240981906 | 2.23E-05 | 0.009536498 |
| TMEM256 | 6.269307582 | 2.025087915 | 0.652372401 | 3.104190049 | 0.001908007 | 0.091733267 |
| SMIM11 | 19.55392207 | 2.013962603 | 0.507851584 | 3.965651908 | 7.32E-05 | 0.015855297 |
| ELANE | 3.132594189 | 1.988622363 | 0.731155111 | 2.719836507 | 0.00653142 | 0.167017195 |
| LTF | 1214.71336 | 1.930379288 | 0.353776671 | 5.456491189 | 4.86E-08 | 0.000159547 |
| SEPTIN5 | 0.944350384 | 1.922756563 | 0.62769872 | 3.063183818 | 0.002189955 | NA |
| FIBCD1 | 3.884721444 | 1.861396938 | 0.579338859 | 3.212967524 | 0.001313711 | 0.074627896 |
| MAGED4 | 4.363715384 | 1.855538856 | 0.773520085 | 2.398824402 | 0.0164478 | 0.254892552 |
| TRIB1AL | 1.733698348 | 1.85365267 | 0.567048678 | 3.268948048 | 0.001079481 | NA |
| OLAH | 11.88324928 | 1.83962362 | 0.512410826 | 3.590134176 | 0.000330508 | 0.03582433 |
| SPC25 | 1.773096316 | 1.82886821 | 0.663967547 | 2.754454217 | 0.005879011 | NA |
| AZU1 | 10.14806304 | 1.808371752 | 0.516662401 | 3.500103254 | 0.000465078 | 0.043243477 |
| LILRA3 | 2.67536658 | 1.780629155 | 0.704659525 | 2.526935479 | 0.011506264 | NA |
| KLLN | 1.063242821 | 1.766277317 | 0.582239524 | 3.033592265 | 0.002416608 | NA |
| OLFM4 | 208.0215195 | 1.758216517 | 0.463078564 | 3.796799619 | 0.000146576 | 0.024324274 |
| MYT1L | 3.145996262 | -1.750196667 | 0.55024278 | -3.180771711 | 0.001468833 | 0.078678373 |
| LOC124900992 | 59.92226012 | 1.745738559 | 0.850751451 | 2.051995983 | 0.040170053 | 0.372977899 |
| HBZ | 19.4614454 | 1.732828416 | 0.649786253 | 2.666766816 | 0.007658479 | 0.179541636 |
| CYP4B1 | 2.496020864 | 1.729923156 | 0.662897376 | 2.60963947 | 0.009063769 | NA |
| SLC39A7 | 8.382965393 | 1.721562713 | 0.487319293 | 3.532720203 | 0.000411308 | 0.041001449 |
| OPRM1 | 1.382654083 | 1.710327445 | 0.630520226 | 2.712565551 | 0.006676457 | NA |
| MANBAL | 10.58151816 | 1.706044697 | 0.496172054 | 3.438413514 | 0.000585133 | 0.048260041 |
| ARFGEF3 | 2.414392877 | 1.705647192 | 0.622431168 | 2.740298495 | 0.006138341 | NA |
| RNF182 | 94.23192907 | 1.700689546 | 0.601541177 | 2.8272205 | 0.004695398 | 0.143855267 |
| LOC105372956 | 1.199372376 | 1.689693467 | 0.639577905 | 2.641888428 | 0.008244521 | NA |
| SCARNA10 | 14.86862818 | -1.677825097 | 0.639151983 | -2.625080015 | 0.008662861 | 0.188271565 |
| KLHDC8A | 26.9391481 | 1.670461895 | 0.415404748 | 4.021287439 | 5.79E-05 | 0.014627548 |
| LOC124902876 | 1.696556377 | 1.648846685 | 0.614634837 | 2.68264437 | 0.007304262 | NA |
| NECAP2 | 105.2679585 | 1.643976313 | 0.387490873 | 4.242619446 | 2.21E-05 | 0.009536498 |
| FAM83A | 2.646378422 | -1.61996579 | 0.677239512 | -2.392013107 | 0.016756244 | NA |
| VSTM2B | 5.939482067 | 1.603468019 | 0.460136507 | 3.484765919 | 0.000492568 | 0.044394952 |
| ACAP1 | 420.0739355 | 1.600069727 | 0.409759981 | 3.904895063 | 9.43E-05 | 0.018744467 |
| SNAP25 | 1.470643472 | 1.599918964 | 0.532709526 | 3.003360903 | 0.002670156 | NA |
| GON7 | 3.537161941 | -1.598903751 | 0.571298609 | -2.798718089 | 0.005130591 | 0.148523663 |
| TFF3 | 17.21638519 | 1.596098394 | 0.442665227 | 3.605655686 | 0.000311366 | 0.034872964 |
| TM9SF1 | 14.32244187 | 1.579590107 | 0.404733948 | 3.902786296 | 9.51E-05 | 0.018744467 |
| TNNI3 | 2.287025055 | 1.571097999 | 0.605598645 | 2.594289156 | 0.009478673 | NA |
| LOC654780 | 1.946525295 | -1.559546383 | 0.582286975 | -2.678312326 | 0.007399419 | NA |
| TRPC6 | 39.21023975 | 1.550784358 | 0.34528459 | 4.491322234 | 7.08E-06 | 0.005167637 |
| LOC105375879 | 1.553473516 | 1.548685533 | 0.688666849 | 2.248816732 | 0.024524158 | NA |
| IFI27 | 25.70527182 | 1.545399255 | 0.65336336 | 2.365298317 | 0.018015553 | 0.263835495 |
| TRHDE | 2.973668714 | 1.537899939 | 0.584943994 | 2.629140488 | 0.008560099 | NA |
| LOC124902719 | 3.136744566 | 1.536222775 | 0.536127643 | 2.8654049 | 0.004164763 | 0.136145634 |
| LOC105369565 | 4.634801508 | 1.53323028 | 0.435413346 | 3.521321279 | 0.000429402 | 0.041364025 |
| GRID1 | 3.289607079 | 1.521253042 | 0.542841477 | 2.802389105 | 0.005072565 | 0.148353718 |
| LOC101928906 | 2.454230204 | 1.519688291 | 0.512714526 | 2.964004753 | 0.003036636 | NA |
| LOC107987441 | 6.082096652 | 1.502070834 | 0.617984167 | 2.430597603 | 0.015073945 | 0.245365493 |
| LOC124904544 | 1.225229055 | 1.500536063 | 0.586933402 | 2.556569548 | 0.010570994 | NA |
| SLC35G3 | 1.46581636 | -1.497710524 | 0.430270144 | -3.480860906 | 0.000499805 | NA |
| LOC105371499 | 6.06510184 | 1.492962447 | 0.402701905 | 3.707363753 | 0.000209428 | 0.029801836 |
| LOC124901100 | 1.692671458 | 1.492341201 | 0.594691555 | 2.509437352 | 0.012092366 | NA |
| LOC124902552 | 2.111894486 | 1.491719736 | 0.507039676 | 2.94201777 | 0.003260812 | NA |
| LOC105378061 | 30.18470688 | 1.488707162 | 0.412296599 | 3.610767507 | 0.000305292 | 0.034787422 |
| LOC124905209 | 1.471946164 | -1.462602357 | 0.550206274 | -2.658280042 | 0.00785406 | NA |
| LINC01579 | 2.378041001 | 1.461260898 | 0.600395432 | 2.433830807 | 0.014939977 | NA |
| CRISP2 | 8.042312205 | 1.448306584 | 0.50901263 | 2.845325439 | 0.004436607 | 0.140828335 |
| CEACAM8 | 177.4828425 | 1.447387371 | 0.421808022 | 3.431388918 | 0.000600499 | 0.048913374 |
| ASIC1 | 29.58015598 | -1.441157287 | 0.269169776 | -5.354082867 | 8.60E-08 | 0.000242152 |
| RNASE3 | 110.9345392 | 1.430156154 | 0.368523187 | 3.880776588 | 0.000104123 | 0.0197354 |
| LOC105369690 | 11.58888381 | 1.429896498 | 0.516075721 | 2.770710653 | 0.005593411 | 0.156010441 |
| HLA-DRB3 | 228.044352 | -1.422426395 | 0.628700449 | -2.262486684 | 0.023667351 | 0.296770316 |
| TRAPPC4 | 38.03520716 | -1.420888897 | 0.596226086 | -2.383137756 | 0.017165769 | 0.259487459 |
| CTSE | 34.15165531 | 1.420693265 | 0.345268668 | 4.11474714 | 3.88E-05 | 0.01061992 |
| LYSET | 5.237649254 | 1.412333378 | 0.465535672 | 3.033781218 | 0.002415095 | 0.103944003 |
| LOC107985357 | 35.68209189 | 1.408112815 | 0.425720474 | 3.307599469 | 0.000940993 | 0.063091332 |
| MMP8 | 278.5706127 | 1.407495395 | 0.430228959 | 3.271503151 | 0.001069774 | 0.06802382 |
| LINC02768 | 1.55928443 | 1.406035515 | 0.563365482 | 2.495778602 | 0.012568102 | NA |
| NRCAM | 70.63995819 | -1.391292612 | 0.274904408 | -5.061005105 | 4.17E-07 | 0.000685077 |
| GTF2H2C_2 | 24.98575519 | -1.385519547 | 0.452563219 | -3.06149393 | 0.002202355 | 0.09668778 |
| ROBO1 | 21.41918883 | -1.385149592 | 0.303546339 | -4.563222858 | 5.04E-06 | 0.003971912 |
| LOC124904038 | 1.249407945 | -1.383462672 | 0.543771996 | -2.544196248 | 0.010952953 | NA |
| FN1 | 29.15295725 | 1.379410237 | 0.381659937 | 3.61423902 | 0.000301231 | 0.034787422 |
| LOC124901298 | 20.4738741 | -1.375744446 | 0.507208877 | -2.712382431 | 0.006680147 | 0.16914422 |
| CDCP2 | 1.415711902 | -1.370113978 | 0.477029816 | -2.872176813 | 0.004076548 | NA |
| LOC124903545 | 1.38978793 | 1.369921774 | 0.591989122 | 2.314099571 | 0.020662261 | NA |
| SMN1 | 4.202684867 | 1.368538285 | 0.467579993 | 2.926853813 | 0.003424098 | 0.122095121 |
| LOC112268317 | 20.65007894 | 1.366173953 | 0.554125861 | 2.465457848 | 0.013683827 | 0.23434892 |
| EMX2OS | 2.577125875 | 1.365543786 | 0.518908203 | 2.631571016 | 0.00849911 | NA |
| CUTA | 20.40615511 | 1.365000599 | 0.366630543 | 3.7230957 | 0.000196795 | 0.028949409 |
| RMRP | 68.69179957 | -1.361581834 | 0.596030763 | -2.284415368 | 0.022347131 | 0.290955516 |
| CEACAM6 | 87.97981214 | 1.361228521 | 0.400527899 | 3.398586028 | 0.000677352 | 0.052774522 |
| C17orf99 | 4.804116866 | 1.359593119 | 0.451813271 | 3.009192527 | 0.002619431 | 0.107347645 |
| MYH16 | 1.134891886 | -1.358170152 | 0.528460516 | -2.570050382 | 0.010168373 | NA |
| PCOLCE2 | 5.590261943 | 1.355849545 | 0.644692009 | 2.103096559 | 0.03545733 | 0.357462274 |
| CD248 | 99.17717363 | -1.355827935 | 0.261382193 | -5.187147296 | 2.14E-07 | 0.000526162 |
| IGHV3-49 | 12.6076157 | 1.355655925 | 0.450888664 | 3.006631201 | 0.0026416 | 0.10780792 |
| LOC105371649 | 2.035148794 | 1.348741818 | 0.498923322 | 2.703304816 | 0.006865376 | NA |
| LOC105374406 | 3.523078557 | 1.342838359 | 0.532952508 | 2.519621051 | 0.011748124 | 0.21700366 |
| RNF187 | 82.9547735 | 1.342520643 | 0.381446319 | 3.519553275 | 0.000432274 | 0.041364025 |
| LOC102723407 | 40.75589336 | -1.339011206 | 0.587974143 | -2.277330087 | 0.022766522 | 0.293316126 |
| BEAN1-AS1 | 0.926836809 | -1.334476499 | 0.570295195 | -2.339975002 | 0.019285031 | NA |
| ZNHIT3 | 12.36553388 | -1.33291408 | 0.432642833 | -3.080864812 | 0.002064003 | 0.093730735 |
| MISP3 | 1.876803438 | 1.327071449 | 0.636450948 | 2.085111905 | 0.037059144 | NA |
| FBL | 112.1406656 | 1.324261849 | 0.541829297 | 2.4440573 | 0.01452312 | 0.240994289 |
| LOC105373595 | 7.420080296 | 1.324206756 | 0.583069908 | 2.271094321 | 0.023141269 | 0.29456331 |
| EIF3K | 170.3352769 | 1.315438748 | 0.566801943 | 2.320808465 | 0.020297183 | 0.277076233 |
| COL9A1 | 1.867547734 | 1.307398342 | 0.480586722 | 2.72042127 | 0.00651988 | NA |
| FRG1EP | 6.05620981 | 1.305252694 | 0.485900508 | 2.686255054 | 0.007225791 | 0.175349788 |
| LOC102724530 | 1.246534694 | 1.303378928 | 0.601903797 | 2.165427322 | 0.030354976 | NA |
| SFRP1 | 5.409986881 | 1.30188095 | 0.52511359 | 2.479236825 | 0.013166385 | 0.229885608 |
| MIR34AHG | 8.542535929 | 1.300507975 | 0.290843887 | 4.471498396 | 7.77E-06 | 0.00546821 |
| LOC105371287 | 3.717208089 | -1.299987163 | 0.392334192 | -3.31346895 | 0.000921463 | 0.062418853 |
| C4B | 2.133231309 | 1.298790236 | 0.563067928 | 2.306631531 | 0.02107537 | NA |
| LOC105373165 | 28.27924223 | 1.297622539 | 0.528982651 | 2.453053112 | 0.014164942 | 0.239811366 |
| TMIGD3 | 50.63806174 | 1.294857242 | 0.366568456 | 3.53237498 | 0.000411845 | 0.041001449 |
| DUXAP9 | 2.023303617 | 1.292301107 | 0.581721329 | 2.221512333 | 0.02631628 | NA |
| SLC17A7 | 2.561373377 | 1.287412812 | 0.372911198 | 3.452330795 | 0.000555766 | NA |
| SCGB3A1 | 6.220678493 | 1.27317661 | 0.456676555 | 2.787917609 | 0.005304803 | 0.151328919 |
| LOC105371498 | 29.59624773 | 1.272470073 | 0.349107911 | 3.644919041 | 0.000267476 | 0.033354367 |
| LOC105375587 | 7.714718857 | 1.270221035 | 0.392597592 | 3.235427471 | 0.001214607 | 0.071477518 |
| AREG | 5.825365782 | 1.269443114 | 0.425566193 | 2.982951032 | 0.002854837 | 0.111099203 |
| KRT79 | 3.162261531 | 1.268131925 | 0.597334488 | 2.122984612 | 0.033755141 | 0.350016489 |
| DEPDC1 | 14.08101115 | 1.267722494 | 0.354390535 | 3.57719061 | 0.000347307 | 0.036610236 |
| LOC102723564 | 2.021833157 | 1.265046117 | 0.585262349 | 2.161502646 | 0.030656533 | NA |
| CRISP3 | 169.1424369 | 1.263142148 | 0.39229121 | 3.21990938 | 0.001282311 | 0.073845303 |
| LOC124903456 | 2.860221317 | -1.26262451 | 0.558908934 | -2.25908808 | 0.023877906 | NA |
| LOC107985138 | 1.003826593 | 1.258997481 | 0.593189858 | 2.122419096 | 0.03380256 | NA |
| PXDN | 11.76206476 | -1.258051564 | 0.402632123 | -3.124568288 | 0.001780662 | 0.086882194 |
| DEFA3 | 201.8074454 | 1.257912829 | 0.42412661 | 2.965889902 | 0.003018085 | 0.115254238 |
| DNAAF4-CCPG1 | 0.937304312 | 1.256507177 | 0.563985793 | 2.227905724 | 0.025886802 | NA |
| LINC02542 | 1.661940509 | 1.255593622 | 0.633538699 | 1.981873601 | 0.047493389 | NA |
| LOC124907761 | 1.65921813 | 1.253314694 | 0.612229933 | 2.047130703 | 0.040645253 | NA |
| LOC105370195 | 2.785538841 | 1.252693959 | 0.529149422 | 2.367372817 | 0.017914876 | NA |
| TRIM27 | 11.00538783 | 1.252569946 | 0.386710661 | 3.239036498 | 0.001199342 | 0.071450155 |
| REG4 | 20.46740172 | -1.251327967 | 0.245816886 | -5.090488239 | 3.57E-07 | 0.00064 |
| SFRP5 | 34.21443625 | -1.249953211 | 0.335899933 | -3.721207086 | 0.000198273 | 0.02895076 |
| GPR176 | 4.050827478 | 1.242986997 | 0.441140641 | 2.817666029 | 0.00483741 | 0.145850614 |
| STAC | 7.70983483 | 1.238848485 | 0.413620104 | 2.995136048 | 0.002743225 | 0.108662409 |
| SCARNA2 | 38.18255954 | -1.235219239 | 0.468039296 | -2.63913575 | 0.008311769 | 0.18496315 |
| LOC105378781 | 4.642449181 | 1.224634081 | 0.412973694 | 2.965404571 | 0.003022851 | 0.115254238 |
| IL34 | 20.56076088 | 1.223615001 | 0.362205352 | 3.378235563 | 0.000729526 | 0.055004498 |
| LOC124907910 | 1.139605722 | 1.221225174 | 0.584565217 | 2.089117071 | 0.036697186 | NA |
| BMP10 | 2.103376598 | 1.21910551 | 0.465670458 | 2.617957591 | 0.00884578 | NA |
| LOC105378834 | 2.454225267 | 1.218818445 | 0.477005495 | 2.555145501 | 0.010614342 | NA |
| LOC124903385 | 3.430787618 | 1.216487814 | 0.387760913 | 3.137211033 | 0.001705633 | 0.0840536 |
| SGPP1 | 5.86240922 | 1.213878632 | 0.380041401 | 3.194069455 | 0.001402824 | 0.077026349 |
| SHANK1 | 13.75789587 | -1.212370102 | 0.34889893 | -3.474846145 | 0.000511146 | 0.044980885 |
| LOC124903995 | 1.569270409 | -1.207963324 | 0.505304349 | -2.390565859 | 0.016822432 | NA |
| LOC124904887 | 2.016558009 | -1.201475127 | 0.475631038 | -2.526065437 | 0.011534799 | NA |
| LOC124907870 | 1.027937596 | 1.197459758 | 0.556472732 | 2.151874997 | 0.031407201 | NA |
| E2F8 | 23.06624532 | 1.196814841 | 0.333689291 | 3.586614469 | 0.000334999 | 0.03582433 |
| CETN2 | 6.529085476 | 1.19405051 | 0.460304746 | 2.594043447 | 0.00948545 | 0.194275683 |
| TMEM14C | 11.28356837 | 1.189186281 | 0.424636306 | 2.800481881 | 0.005102637 | 0.148523663 |
| LOC105377923 | 2.192699488 | 1.188950464 | 0.58493078 | 2.032634466 | 0.042089467 | NA |
| PFDN5 | 109.996293 | -1.186612749 | 0.374170865 | -3.171312519 | 0.001517518 | 0.079617828 |
| LOC124900379 | 1.014460651 | -1.186359745 | 0.544225539 | -2.17990458 | 0.029264535 | NA |
| PMP22 | 57.94776507 | 1.184798248 | 0.298174388 | 3.973507769 | 7.08E-05 | 0.015511558 |
| LCN2 | 350.1332595 | 1.17929946 | 0.358434909 | 3.290135614 | 0.001001391 | 0.066416132 |
| MEG3 | 9.541956353 | 1.178047612 | 0.35240661 | 3.342864687 | 0.000829183 | 0.059435853 |
| IGHV4-34 | 18.8062881 | 1.177416007 | 0.404312317 | 2.912144789 | 0.003589562 | 0.124583992 |
| MMP1 | 4.50848887 | 1.174025218 | 0.578816841 | 2.028319037 | 0.042527697 | 0.384715961 |
| RNF212 | 28.79050031 | -1.166179192 | 0.211544282 | -5.512695419 | 3.53E-08 | 0.000139316 |
| PSMC4 | 22.91204876 | -1.159093474 | 0.365696772 | -3.169548009 | 0.001526762 | 0.079617828 |
| TMEM45A | 11.79027792 | 1.157986995 | 0.458132569 | 2.527624257 | 0.011483718 | 0.214305233 |
| GCLC-AS1 | 1.129591274 | -1.157799994 | 0.539818544 | -2.144794777 | 0.031969259 | NA |
| LYPD2 | 60.08256391 | 1.157798262 | 0.319065792 | 3.628713237 | 0.000284837 | 0.033823586 |
| IL9R | 1.376411574 | 1.157614518 | 0.541237465 | 2.138829241 | 0.0324495 | NA |
| SAMD14 | 44.14668499 | 1.15051912 | 0.323636518 | 3.554973115 | 0.000378018 | 0.038608734 |
| LOC285847 | 4.207264517 | 1.149860152 | 0.571897387 | 2.01060571 | 0.04436712 | 0.391654581 |
| OLR1 | 49.57949587 | 1.14789633 | 0.371118085 | 3.093075694 | 0.001980936 | 0.093362898 |
| OLIG2 | 185.2372843 | 1.147799911 | 0.332849469 | 3.448405416 | 0.000563907 | 0.047591088 |
| LRRN3 | 530.0827016 | -1.140013216 | 0.233631632 | -4.879532807 | 1.06E-06 | 0.001310077 |
| MICB-DT | 7.16509991 | 1.139916294 | 0.570869416 | 1.996807434 | 0.045846106 | 0.397588401 |
| LOC100507336 | 2.258555629 | -1.138488608 | 0.517019454 | -2.202022764 | 0.0276637 | NA |
| OVOS2 | 3.517866528 | 1.138335046 | 0.564660153 | 2.015964895 | 0.043803647 | 0.389327317 |
| LOC105369595 | 18.99019765 | 1.137473973 | 0.367461817 | 3.095488895 | 0.001964887 | 0.093362898 |
| CNTN4 | 2.58192478 | 1.13746048 | 0.48268802 | 2.356512764 | 0.018447434 | NA |
| UNC5C | 2.258528347 | -1.134256493 | 0.429392925 | -2.641535124 | 0.008253125 | NA |
| ADORA3 | 173.7266722 | 1.133517062 | 0.237075 | 4.781259348 | 1.74E-06 | 0.001807284 |
| DDAH2 | 3.951271485 | -1.132493552 | 0.467700872 | -2.421405687 | 0.015460612 | 0.247973629 |
| IGSF3 | 5.013871754 | 1.12979215 | 0.399525673 | 2.827833666 | 0.004686415 | 0.143855267 |
| LOC124902245 | 1.802159981 | 1.12886806 | 0.502567028 | 2.246203981 | 0.024690945 | NA |
| LOC643327 | 5.037849598 | 1.123818351 | 0.397220103 | 2.829208143 | 0.004666334 | 0.143855267 |
| PLAAT5 | 42.67096045 | 1.117871548 | 0.285949328 | 3.909334413 | 9.26E-05 | 0.018690355 |
| LRCOL1 | 2.216803204 | -1.11720956 | 0.484601984 | -2.305416811 | 0.021143241 | NA |
| HBG2 | 6497.101243 | 1.116331953 | 0.467431275 | 2.388226917 | 0.016929885 | 0.257899454 |
| DXO | 3.550212959 | -1.115643994 | 0.500886193 | -2.227340282 | 0.02592454 | 0.308776156 |
| LINC02009 | 35.10770272 | 1.114559042 | 0.417174412 | 2.671686017 | 0.007547123 | 0.179239623 |
| LOC124900823 | 1.139442407 | -1.112492769 | 0.531024348 | -2.094993897 | 0.036171535 | NA |
| CDR2L | 6.628997339 | 1.109577072 | 0.380869901 | 2.913270566 | 0.003576646 | 0.124583992 |
| ST3GAL6-AS1 | 2.274357174 | 1.108216619 | 0.479538449 | 2.311006806 | 0.020832479 | NA |
| LOC124900812 | 320.9084763 | 1.105557274 | 0.298665756 | 3.701653945 | 0.000214199 | 0.030159167 |
| MAP3K19 | 2.230723302 | 1.104315795 | 0.449695792 | 2.455695194 | 0.014061237 | NA |
| SEMA3B-AS1 | 1.999359263 | 1.104226475 | 0.442606803 | 2.494824902 | 0.01260193 | NA |
| LOC105374128 | 4.24533867 | 1.103654717 | 0.458159782 | 2.408886072 | 0.016001292 | 0.252132271 |
| TRBV28 | 25.88332383 | 1.103629486 | 0.310574083 | 3.553514431 | 0.00038012 | 0.038623362 |
| RN7SK | 824.1690317 | -1.100266518 | 0.486467317 | -2.261748076 | 0.023712973 | 0.296770316 |
| FCER1A | 11.27292805 | -1.100036313 | 0.486013417 | -2.263386719 | 0.023611861 | 0.296456687 |
| INHBA | 8.184738547 | 1.096321098 | 0.457295716 | 2.397400762 | 0.016511853 | 0.255680791 |
| IGFBP2 | 19.17358951 | 1.096127602 | 0.554373143 | 1.977237925 | 0.048014743 | 0.404645835 |
| BEX1 | 9.141684269 | 1.094122293 | 0.448992064 | 2.436841052 | 0.01481619 | 0.243848404 |
| HTRA3 | 5.867476302 | 1.089679527 | 0.453737212 | 2.401565264 | 0.016325097 | 0.253386068 |
| LOC105376363 | 2.201095436 | 1.088247734 | 0.486238639 | 2.238093904 | 0.025214932 | NA |
| RNF135 | 38.3586445 | 1.087888687 | 0.3704012 | 2.937054973 | 0.003313454 | 0.120413728 |
| TNNT1 | 105.0591027 | 1.085710509 | 0.306072934 | 3.54722809 | 0.000389307 | 0.039353986 |
| ALOX15 | 1315.193066 | 1.082792765 | 0.343859164 | 3.148942587 | 0.001638624 | 0.08201928 |
| LOC124909353 | 1.565069135 | -1.081886747 | 0.545244564 | -1.984222894 | 0.047230997 | NA |
| PAX2 | 1.885388055 | 1.081437882 | 0.537198325 | 2.013107322 | 0.04410334 | NA |
| FOXC1 | 6.554950482 | 1.081054384 | 0.438874607 | 2.463242041 | 0.013768693 | 0.234972283 |
| RIBC2 | 3.528209642 | 1.081006243 | 0.414205537 | 2.609830495 | 0.00905871 | 0.191403941 |
| TAX1BP1-AS1 | 1.742940931 | 1.080737666 | 0.486857234 | 2.2198246 | 0.026430676 | NA |
| HNRNPL | 255.2989038 | -1.078058667 | 0.390246924 | -2.762503943 | 0.005735987 | 0.158030123 |
| LOC101929547 | 6.422093773 | 1.075767802 | 0.336233591 | 3.19946558 | 0.001376826 | 0.076337862 |
| SLC43A2 | 419.335132 | 1.074743983 | 0.323193132 | 3.325392393 | 0.000882942 | 0.061367977 |
| FBLN2 | 84.69972748 | -1.073416783 | 0.171555432 | -6.256967631 | 3.93E-10 | 7.74E-06 |
| CDRT3 | 2.194165204 | 1.071822527 | 0.508907767 | 2.106123341 | 0.03519364 | NA |
| LOC105377938 | 3.958965192 | 1.071267973 | 0.356567216 | 3.004392788 | 0.002661115 | 0.10792696 |
| LOC124902802 | 1.570224247 | 1.071018356 | 0.465759875 | 2.299507564 | 0.021476134 | NA |
| MED18 | 12.85223209 | -1.065716825 | 0.408612037 | -2.608138594 | 0.009103609 | 0.191403941 |
| LOC101927377 | 2.439695436 | 1.064831747 | 0.457109068 | 2.329491628 | 0.019833036 | NA |
| SCARNA7 | 6.478480043 | -1.064804819 | 0.518543181 | -2.053454483 | 0.04002852 | 0.372633128 |
| SOCAR | 3.797753306 | 1.063553815 | 0.413221692 | 2.573809253 | 0.010058571 | 0.200489302 |
| LOC124900693 | 3.164718543 | 1.062863341 | 0.368910033 | 2.881090903 | 0.003963013 | 0.132180909 |
| PRSS33 | 503.2338545 | 1.055665169 | 0.372929848 | 2.830733916 | 0.004644134 | 0.143855267 |
| PCDHGA9 | 2.043958074 | 1.053157647 | 0.495546504 | 2.125244833 | 0.033566189 | NA |
| LOC124902652 | 7.232162784 | 1.052499368 | 0.391229604 | 2.690234475 | 0.007140183 | 0.175349788 |
| RGL2 | 50.76189204 | 1.050265877 | 0.341572588 | 3.074795556 | 0.002106469 | 0.093730735 |
| NDUFB10 | 21.01322663 | 1.050254444 | 0.38030287 | 2.761626395 | 0.005751425 | 0.158030123 |
| LOC124902659 | 2.070367434 | 1.049936076 | 0.485772548 | 2.161373838 | 0.030666473 | NA |
| CCDC192 | 1.217324098 | -1.043759253 | 0.469079554 | -2.225122039 | 0.026073047 | NA |
| TXNDC2 | 3.107320254 | 1.039484801 | 0.381539963 | 2.724445409 | 0.006440958 | 0.165987045 |
| PRSS42P | 1.087092145 | -1.033950743 | 0.515221181 | -2.006809465 | 0.044769955 | NA |
| LOC102724135 | 2.618142881 | -1.032726087 | 0.38004079 | -2.717408536 | 0.006579534 | NA |
| SCRG1 | 8.145743093 | 1.03077252 | 0.317227788 | 3.249313457 | 0.001156839 | 0.070381529 |
| METAP1D | 3.947710916 | 1.028333446 | 0.497626239 | 2.06647754 | 0.038783411 | 0.369420081 |
| LOC124904605 | 2.943556234 | 1.027768246 | 0.420996934 | 2.441272522 | 0.014635606 | NA |
| LOC105370655 | 55.94280675 | -1.02543324 | 0.305577403 | -3.355723396 | 0.000791577 | 0.057790982 |
| TUSC3 | 5.811596421 | 1.025092771 | 0.498855822 | 2.054887859 | 0.039889837 | 0.372481514 |
| CRHR2 | 2.137628518 | -1.024938508 | 0.408413241 | -2.50956239 | 0.012088086 | NA |
| CAMSAP3 | 6.77863881 | -1.024218792 | 0.33687292 | -3.040371402 | 0.002362866 | 0.102592091 |
| TP73-AS3 | 1.51663516 | 1.022920555 | 0.505563324 | 2.023328246 | 0.043039317 | NA |
| CNN3-DT | 2.988291409 | -1.022119224 | 0.422007979 | -2.422037674 | 0.01543375 | NA |
| ITGA2B | 1106.165826 | 1.01857909 | 0.338389039 | 3.010082993 | 0.002611763 | 0.107256406 |
| ALB | 2.071735011 | -1.01651887 | 0.403360877 | -2.520122616 | 0.011731396 | NA |
| RNASE1 | 17.98319624 | 1.01595487 | 0.383778659 | 2.647241698 | 0.008115133 | 0.182817714 |
| UQCRC2 | 127.0812331 | -1.015749923 | 0.493016481 | -2.060275795 | 0.039372183 | 0.371834908 |
| LOC105373074 | 1.616329062 | 1.014664749 | 0.479161657 | 2.117583354 | 0.034210369 | NA |
| LOC105379396 | 5.246689318 | -1.012403468 | 0.254109611 | -3.984121114 | 6.77E-05 | 0.015511558 |
| RAB3B | 1.472599393 | -1.011667437 | 0.515657113 | -1.961899508 | 0.049774185 | NA |
| LOC112267871 | 1.474079329 | 1.010776839 | 0.512592985 | 1.971889723 | 0.048622197 | NA |
| SERPINB10 | 55.40835807 | 1.009629599 | 0.289195737 | 3.491163492 | 0.000480922 | 0.043888573 |
| GPR85 | 4.295241302 | 1.009430249 | 0.452445072 | 2.231055902 | 0.025677426 | 0.30750512 |
| LOC107985090 | 2.253188917 | -1.009111061 | 0.457720823 | -2.204643113 | 0.027479148 | NA |
| NELFE | 5.567744085 | -1.008271972 | 0.433760991 | -2.324487431 | 0.020099384 | 0.275520908 |
| CENPN | 4.764273504 | 1.007196579 | 0.384023149 | 2.622749646 | 0.008722334 | 0.188428302 |
| DTL | 47.79927685 | 1.006009321 | 0.307425286 | 3.272370127 | 0.001066499 | 0.06802382 |
| TRAV40 | 3.453538582 | -1.005402873 | 0.427185761 | -2.35354959 | 0.018595127 | 0.266925611 |
| RIPK3 | 28.42020149 | 1.002765965 | 0.44765351 | 2.240049373 | 0.025087718 | 0.303909072 |
| CEP55 | 47.10534513 | 1.001317821 | 0.277255232 | 3.611538052 | 0.000304386 | 0.034787422 |
| IGHV2-5 | 9.364686554 | 0.997753132 | 0.402329042 | 2.479943102 | 0.013140335 | 0.229885608 |
| LOC112268261 | 92.39361204 | -0.994724698 | 0.241050367 | -4.126625942 | 3.68E-05 | 0.01061992 |
| CLU | 1472.316745 | 0.992971553 | 0.26521684 | 3.743998884 | 0.000181114 | 0.027675412 |
| VWCE | 161.8094296 | 0.992721659 | 0.310471464 | 3.19746506 | 0.001386412 | 0.076337862 |
| FSTL1 | 90.23134679 | 0.991921105 | 0.332174738 | 2.986142511 | 0.002825209 | 0.110497082 |
| GYPB | 35.33302161 | 0.991384556 | 0.45664865 | 2.171000736 | 0.029931117 | 0.330339194 |
| IFNG-AS1 | 106.3163689 | -0.990678719 | 0.268551632 | -3.688969274 | 0.000225164 | 0.031178636 |
| MIR6843 | 2.46153267 | 0.989888059 | 0.466525748 | 2.121829426 | 0.033852065 | NA |
| LOC124901471 | 1.272738422 | -0.98689289 | 0.500585908 | -1.971475574 | 0.048669504 | NA |
| LOC101927511 | 3.067059476 | 0.986649118 | 0.397767408 | 2.480467473 | 0.013121023 | NA |
| LOC124905038 | 2.342549314 | 0.986582101 | 0.491917713 | 2.005583605 | 0.044900693 | NA |
| DYNC1I2 | 98.20872927 | 0.983851412 | 0.309850098 | 3.175249641 | 0.001497076 | 0.079617828 |
| KIF26A | 10.58798714 | 0.983631314 | 0.363553679 | 2.705601319 | 0.006818085 | 0.170366407 |
| PDE6B-AS1 | 1.737867977 | -0.98352433 | 0.473135176 | -2.078738549 | 0.037641388 | NA |
| LOC124902798 | 4.229406001 | 0.982722094 | 0.342077604 | 2.872804539 | 0.004068457 | 0.134785602 |
| PERCC1 | 3.374259541 | -0.982394594 | 0.350907819 | -2.799580233 | 0.00511691 | 0.148523663 |
| CACHD1 | 45.9534165 | -0.980858714 | 0.232488694 | -4.21895232 | 2.45E-05 | 0.009676233 |
| CAGE1 | 1.62182221 | 0.978920415 | 0.47482649 | 2.061638167 | 0.039242197 | NA |
| LINC02964 | 5.605133563 | 0.977251056 | 0.344931304 | 2.833175895 | 0.004608802 | 0.143520855 |
| INAVA | 5.816686868 | 0.976843185 | 0.393519659 | 2.482323725 | 0.013052863 | 0.229614885 |
| LOC100131532 | 4.31769269 | -0.976140736 | 0.380674932 | -2.564236973 | 0.010340292 | 0.202461893 |
| LOC101929698 | 49.3836737 | -0.974173703 | 0.172529982 | -5.646402394 | 1.64E-08 | 9.88E-05 |
| UGT3A2 | 2.677890534 | 0.973202403 | 0.438545601 | 2.219158966 | 0.026475912 | NA |
| HSF1 | 92.52223142 | 0.971778228 | 0.405106848 | 2.398819556 | 0.016448018 | 0.254892552 |
| LOC124905210 | 2.194251468 | 0.970422106 | 0.46468964 | 2.088323093 | 0.0367687 | NA |
| LOC107986639 | 13.1841016 | 0.969901738 | 0.305740777 | 3.172300883 | 0.001512362 | 0.079617828 |
| BTBD17 | 3.9168611 | 0.967415404 | 0.487708801 | 1.98359226 | 0.047301313 | 0.401852773 |
| FBN1 | 31.90115474 | 0.966250264 | 0.268352664 | 3.600673267 | 0.000317394 | 0.035134003 |
| MCM10 | 25.2634758 | 0.966033691 | 0.324696307 | 2.975191492 | 0.002928057 | 0.112950803 |
| LOC642131 | 3.944005211 | 0.965831621 | 0.407610235 | 2.369497963 | 0.017812253 | 0.262847174 |
| PCDHGA10 | 7.414125476 | 0.964889728 | 0.286494042 | 3.367922494 | 0.000757369 | 0.056555296 |
| IL5RA | 751.3905422 | 0.964507279 | 0.263877009 | 3.655139502 | 0.000257042 | 0.032713737 |
| HNRNPA3P1 | 1.444808419 | -0.961067946 | 0.482822625 | -1.990519698 | 0.046533716 | NA |
| PRSS41 | 33.74522462 | 0.960186961 | 0.344669852 | 2.785816502 | 0.005339309 | 0.15187367 |
| TICRR | 16.56762928 | 0.959168135 | 0.352300275 | 2.722586956 | 0.006477298 | 0.166467415 |
| LOC124900667 | 1.820405282 | -0.958468807 | 0.400889955 | -2.390852639 | 0.016809298 | NA |
| MYCT1 | 48.68394427 | 0.95577829 | 0.219843928 | 4.347530998 | 1.38E-05 | 0.007841458 |
| CEBPE | 264.3203321 | 0.953393211 | 0.241970783 | 3.940117064 | 8.14E-05 | 0.016898757 |
| IL36A | 5.165085691 | -0.952845711 | 0.243291036 | -3.916485076 | 8.98E-05 | 0.018449072 |
| SLC28A3 | 24.2054661 | 0.951339647 | 0.302071569 | 3.149384926 | 0.001636145 | 0.08201928 |
| MYL9 | 535.6533723 | 0.950212305 | 0.353996467 | 2.684242341 | 0.007269439 | 0.175555308 |
| BIRC5 | 58.88752334 | 0.950179686 | 0.290596963 | 3.269750912 | 0.001076422 | 0.06822648 |
| CCDC3 | 8.773095138 | 0.947971842 | 0.36147015 | 2.622545298 | 0.008727566 | 0.188428302 |
| SUGT1P1 | 2.579165422 | -0.945641298 | 0.447460408 | -2.113351888 | 0.034570661 | NA |
| TUBB2A | 186.4961016 | 0.944542749 | 0.394209455 | 2.396042854 | 0.016573153 | 0.255681257 |
| GATA1 | 409.1120303 | 0.941786747 | 0.206006259 | 4.571641415 | 4.84E-06 | 0.003971912 |
| AFAP1L1 | 4.112736234 | -0.941310228 | 0.391577431 | -2.403892954 | 0.016221523 | 0.253173913 |
| DDX11L1 | 7.220875468 | 0.940641149 | 0.451034467 | 2.085519439 | 0.037022175 | 0.363254118 |
| IDO1 | 394.6220163 | 0.940366219 | 0.268002893 | 3.508791304 | 0.000450148 | 0.042456062 |
| NOG | 239.8328045 | -0.939176259 | 0.226627134 | -4.144147441 | 3.41E-05 | 0.01061992 |
| DLGAP5 | 55.44024037 | 0.93864007 | 0.320435525 | 2.929263442 | 0.003397663 | 0.122095121 |
| FAM88F | 1.227035841 | -0.938634829 | 0.460542165 | -2.038108345 | 0.041539099 | NA |
| EBF3 | 3.462926481 | -0.937229176 | 0.329725875 | -2.842449581 | 0.004476831 | 0.141421948 |
| NMRAL2P | 4.827043586 | 0.936866199 | 0.422920706 | 2.215228967 | 0.026744353 | 0.314361764 |
| LOC107984613 | 1.780926085 | -0.936078346 | 0.452138304 | -2.070336305 | 0.038420862 | NA |
| LOC107984706 | 14.197491 | -0.935412314 | 0.362676974 | -2.579188591 | 0.00990327 | 0.198994156 |
| CD24 | 1392.095503 | 0.933955401 | 0.221725704 | 4.212210788 | 2.53E-05 | 0.009773444 |
| LOC105377276 | 7.079237721 | 0.931805612 | 0.310483466 | 3.001144065 | 0.002689673 | 0.107981325 |
| TMEM184B-AS1 | 2.631091893 | 0.930973979 | 0.373624947 | 2.491733988 | 0.01271212 | NA |
| LOC101927369 | 12.28842766 | 0.930685419 | 0.301725455 | 3.084543928 | 0.002038645 | 0.093362898 |
| PPBP | 2783.953389 | 0.928532667 | 0.28538558 | 3.253607518 | 0.001139496 | 0.069756969 |
| GCK | 2.543846266 | -0.928171334 | 0.436327443 | -2.127235748 | 0.0334005 | NA |
| SIGLEC8 | 465.2532945 | 0.927540517 | 0.404997111 | 2.290239837 | 0.022007417 | 0.288554917 |
| MIR31HG | 2.765722215 | -0.925508926 | 0.448274097 | -2.064604966 | 0.038960393 | NA |
| RNASE2 | 899.4203683 | 0.924683041 | 0.286327204 | 3.229462754 | 0.00124023 | 0.072592129 |
| LOC107985293 | 1.372889809 | -0.924111834 | 0.426271215 | -2.167896404 | 0.030166569 | NA |
| LOC107984156 | 2.109546822 | 0.923336382 | 0.455268976 | 2.028111799 | 0.042548839 | NA |
| ZNF251 | 2.785347998 | -0.922539069 | 0.349291612 | -2.641171554 | 0.008261987 | NA |
| NHIP | 34.64978637 | -0.922042777 | 0.304869149 | -3.024388595 | 0.002491362 | 0.105372881 |
| GSDMD | 132.9179997 | 0.918265872 | 0.447131503 | 2.053681895 | 0.04000649 | 0.372633128 |
| SLC29A1 | 819.9907075 | 0.918213014 | 0.252242413 | 3.640200725 | 0.000272426 | 0.033354367 |
| LEF1-AS1 | 45.23720226 | -0.917624936 | 0.163524203 | -5.611554236 | 2.01E-08 | 9.88E-05 |
| LOC105374555 | 1.286275388 | -0.915795383 | 0.4535216 | -2.019298272 | 0.043456227 | NA |
| CHST6 | 3.550692711 | 0.914809754 | 0.444921671 | 2.056114171 | 0.039771513 | 0.372140818 |
| MIR10523 | 4.71228078 | 0.913602012 | 0.459376053 | 1.988788936 | 0.046724504 | 0.400797838 |
| HOXC5 | 7.841359446 | 0.913188348 | 0.358385033 | 2.54806497 | 0.01083223 | 0.206903981 |
| IL1RL1 | 52.9773426 | 0.913022854 | 0.309058773 | 2.954204621 | 0.003134761 | 0.117699819 |
| SRD5A3-AS1 | 3.54965849 | 0.912794518 | 0.341174621 | 2.675446708 | 0.007462974 | 0.178748649 |
| LOC124901436 | 2.589299633 | 0.910113742 | 0.437333638 | 2.081051311 | 0.03742921 | NA |
| SYT3 | 4.220151787 | -0.910007439 | 0.336631942 | -2.703271218 | 0.00686607 | 0.170888844 |
| HMOX2 | 64.7336886 | -0.908903185 | 0.450714395 | -2.016583437 | 0.043739003 | 0.389327317 |
| MIR4477A | 2.117700394 | 0.908509094 | 0.448272521 | 2.026689239 | 0.042694204 | NA |
| ANLN | 33.74113771 | 0.906689333 | 0.309837412 | 2.926339095 | 0.003429769 | 0.122095121 |
| TNNI2 | 23.5954393 | -0.905314123 | 0.410842909 | -2.203552995 | 0.027555796 | 0.319061291 |
| SLC25A28-DT | 2.382517897 | -0.90434097 | 0.387489214 | -2.333848109 | 0.019603677 | NA |
| ECRG4 | 14.07343102 | -0.903935251 | 0.227098523 | -3.980366057 | 6.88E-05 | 0.015511558 |
| RCAN2 | 42.41171741 | 0.903866775 | 0.240091979 | 3.764668763 | 0.00016677 | 0.026298963 |
| LOC105374836 | 1.862216176 | 0.902775434 | 0.449337394 | 2.009125984 | 0.044523775 | NA |
| CLC | 4485.256706 | 0.902072269 | 0.28896811 | 3.121701794 | 0.00179809 | 0.087515912 |
| CMTM5 | 143.2125681 | 0.90160501 | 0.285472749 | 3.158287486 | 0.00158699 | 0.080978003 |
| LOC124900753 | 4.489741636 | -0.90014625 | 0.335086802 | -2.68630768 | 0.007224653 | 0.175349788 |
| NPC1L1 | 2.606061087 | -0.899359329 | 0.374274657 | -2.402939421 | 0.016263881 | NA |
| LOC124905044 | 3.329673055 | -0.899179817 | 0.329500623 | -2.728916893 | 0.006354272 | 0.164700379 |
| LOC107985926 | 27.28089303 | 0.898973407 | 0.367283627 | 2.447627228 | 0.014380035 | 0.240219696 |
| PDE4DIPP1 | 4.185400301 | 0.898456308 | 0.414880322 | 2.165579471 | 0.030343337 | 0.33229325 |
| LOC124902302 | 26.46344258 | 0.898103233 | 0.271834295 | 3.30386286 | 0.000953625 | 0.063721525 |
| STOX1 | 15.31105088 | -0.898095415 | 0.418615009 | -2.145397072 | 0.031921113 | 0.340860769 |
| KIR3DX1 | 1.743631425 | 0.897028782 | 0.425738437 | 2.106995057 | 0.035118008 | NA |
| CARMIL3 | 8.040691057 | 0.896093793 | 0.367741393 | 2.436749876 | 0.014819926 | 0.243848404 |
| LOC105376289 | 7.798825838 | -0.89538197 | 0.302808202 | -2.956927736 | 0.003107209 | 0.117427632 |
| SCARNA21 | 7.818019649 | -0.894811783 | 0.380536766 | -2.351446334 | 0.018700587 | 0.267313974 |
| ETV1 | 3.725028855 | 0.890521012 | 0.439191571 | 2.027636847 | 0.042597325 | 0.384820568 |
| RYR2 | 11.93670604 | 0.888182281 | 0.29344801 | 3.026710869 | 0.002472303 | 0.105257104 |
| CXCL5 | 161.2142811 | 0.888023695 | 0.320706766 | 2.768958405 | 0.005623581 | 0.156349841 |
| ABCA13 | 54.44155129 | 0.887897687 | 0.341076615 | 2.603220653 | 0.009235249 | 0.192434794 |
| LINC01529 | 3.618127057 | 0.887161913 | 0.444502347 | 1.995854283 | 0.045949786 | 0.397818343 |
| TREML1 | 455.4395585 | 0.885659819 | 0.276402042 | 3.204244842 | 0.001354173 | 0.075833667 |
| MSRB1 | 115.1424566 | 0.884462845 | 0.286588365 | 3.086178481 | 0.002027471 | 0.093362898 |
| FILIP1L | 29.57504678 | 0.883116254 | 0.297601976 | 2.967440824 | 0.003002901 | 0.115203891 |
| LOC124909396 | 3.540741501 | -0.879860422 | 0.359434019 | -2.447905251 | 0.014368944 | 0.240219696 |
| KIF20A | 20.91525282 | 0.879492564 | 0.292884256 | 3.002867329 | 0.00267449 | 0.10792696 |
| LOC107987207 | 24.11412274 | -0.878835122 | 0.336563472 | -2.611201734 | 0.009022465 | 0.191403941 |
| LINC01562 | 2.219762351 | -0.87414967 | 0.355998493 | -2.455486994 | 0.014069384 | NA |
| KRT8 | 3.864008228 | 0.873943309 | 0.410151281 | 2.130782833 | 0.033107037 | 0.347165935 |
| CA6 | 82.88695227 | -0.873917755 | 0.22954711 | -3.807139001 | 0.000140584 | 0.024324274 |
| LOC124900336 | 4.780221595 | 0.87296603 | 0.362568619 | 2.407726385 | 0.016052207 | 0.252533684 |
| SLC25A24 | 13.31834519 | 0.871794186 | 0.392735569 | 2.219799413 | 0.026432387 | 0.311997131 |
| CLIC6 | 4.947903643 | 0.870089053 | 0.384897439 | 2.260573766 | 0.023785664 | 0.296770316 |
| LOC124904760 | 3.53576303 | -0.869741235 | 0.295949515 | -2.938816216 | 0.003294684 | 0.120334569 |
| THBS1 | 388.0625857 | 0.866278437 | 0.25768487 | 3.361774541 | 0.000774433 | 0.057389582 |
| TTC24 | 17.18826811 | -0.865596503 | 0.337305789 | -2.566207073 | 0.010281743 | 0.202461893 |
| CDK15 | 13.6195494 | 0.865149207 | 0.365032886 | 2.370058261 | 0.017785282 | 0.262847174 |
| KIF18B | 24.1050143 | 0.863978322 | 0.297085019 | 2.908185418 | 0.003635327 | 0.125498372 |
| CA12 | 8.355663077 | 0.863220569 | 0.397227423 | 2.173114237 | 0.02977172 | 0.330042436 |
| HLA-DRA | 261.1336827 | -0.863201128 | 0.408520456 | -2.11299365 | 0.034601311 | 0.354070238 |
| DOCK6-AS1 | 7.699948379 | 0.862831762 | 0.307064439 | 2.809937111 | 0.004955118 | 0.146439713 |
| TMEM52B | 3.712243206 | 0.862265928 | 0.419873113 | 2.05363454 | 0.040011076 | 0.372633128 |
| FOXJ1 | 9.607384653 | -0.862216073 | 0.24530852 | -3.514823189 | 0.000440047 | 0.041904366 |
| COBL | 3.138984029 | -0.861136114 | 0.421329721 | -2.043853235 | 0.040968048 | 0.376729189 |
| LONRF2 | 10.65533061 | 0.860861475 | 0.352230289 | 2.444030233 | 0.014524209 | 0.240994289 |
| MIOX | 4.597291721 | 0.85999721 | 0.353088066 | 2.435645081 | 0.014865262 | 0.244186705 |
| ADAMTSL2 | 5.514678214 | -0.859110717 | 0.305186725 | -2.815033043 | 0.004877222 | 0.146262666 |
| PIP4P1 | 127.2449706 | -0.858568836 | 0.431856277 | -1.988089284 | 0.046801816 | 0.40081035 |
| BPI | 337.2078705 | 0.858395687 | 0.305331146 | 2.811359727 | 0.00493326 | 0.146262666 |
| TOP2A | 162.7771876 | 0.857507055 | 0.268627112 | 3.192183571 | 0.001412016 | 0.0773157 |
| ASCL5 | 4.278749213 | -0.853558255 | 0.334493971 | -2.551789656 | 0.01071712 | 0.205701915 |
| CD300A | 243.1286122 | 0.853232538 | 0.42880836 | 1.989775893 | 0.046615628 | 0.400386606 |
| ACKR1 | 14.66300033 | 0.852333415 | 0.359540604 | 2.370617964 | 0.017758376 | 0.262847174 |
| ASPM | 65.60374244 | 0.852247698 | 0.284574512 | 2.994813874 | 0.002746124 | 0.108662409 |
| DMC1 | 5.533521146 | 0.850872029 | 0.347595347 | 2.447880956 | 0.014369912 | 0.240219696 |
| NSF | 7.069187742 | 0.850717607 | 0.39275891 | 2.166004601 | 0.030310835 | 0.332121838 |
| TTK | 28.92349169 | 0.849232828 | 0.25901541 | 3.278696148 | 0.001042879 | 0.067682688 |
| PCLAF | 61.32753216 | 0.849125408 | 0.280792254 | 3.0240343 | 0.002494282 | 0.105372881 |
| CDT1 | 53.06261726 | 0.847925208 | 0.240389502 | 3.527297162 | 0.000419825 | 0.041364025 |
| RRM2 | 262.5356355 | 0.847855314 | 0.287798016 | 2.946008201 | 0.003219039 | 0.119273853 |
| KLC4-AS1 | 2.523158345 | 0.846694406 | 0.37027391 | 2.286670444 | 0.022215066 | NA |
| LOC124900837 | 10.12849556 | -0.846285381 | 0.256826146 | -3.295168325 | 0.000983627 | 0.065504269 |
| VSTM2L | 3.018394242 | 0.845358388 | 0.379935765 | 2.225003451 | 0.026081006 | NA |
| CKAP2L | 20.80405596 | 0.840931038 | 0.304017983 | 2.766056898 | 0.005673863 | 0.156862827 |
| FAM9C | 4.138513085 | 0.839038129 | 0.311900766 | 2.690080369 | 0.007143481 | 0.175349788 |
| SYT1 | 5.606128012 | 0.837919532 | 0.322556825 | 2.597742376 | 0.009383887 | 0.193488679 |
| LOC124907884 | 2.738651382 | -0.836706103 | 0.390160324 | -2.144518679 | 0.03199135 | NA |
| LGALS12 | 395.1891193 | 0.836696657 | 0.193105054 | 4.33285738 | 1.47E-05 | 0.007841458 |
| LOC124909350 | 6.409059485 | 0.836545486 | 0.369409661 | 2.264546857 | 0.023540501 | 0.29593773 |
| DCLK2 | 17.43060728 | 0.835884305 | 0.345971702 | 2.416048192 | 0.015689985 | 0.24991923 |
| KDM4E | 1.980326802 | -0.835570618 | 0.426018959 | -1.961346087 | 0.049838665 | NA |
| LINC01869 | 3.075260106 | -0.833002392 | 0.399893949 | -2.083058259 | 0.037245914 | NA |
| CACNG6 | 114.6683006 | 0.832925005 | 0.3192713 | 2.608831441 | 0.009085198 | 0.191403941 |
| LOC105370821 | 3.196088763 | -0.832690915 | 0.336576189 | -2.474004232 | 0.013360811 | 0.232247191 |
| HBA1 | 20468.19457 | 0.831286326 | 0.324292336 | 2.563385668 | 0.010365683 | 0.202461893 |
| OR10AD1 | 4.020913773 | -0.83047858 | 0.301857334 | -2.751228766 | 0.005937216 | 0.160321111 |
| GYPA | 16.0609743 | 0.830161755 | 0.39278905 | 2.113505342 | 0.034557538 | 0.353869192 |
| AICDA | 12.13326681 | 0.829564893 | 0.3152792 | 2.631207178 | 0.008508215 | 0.187091308 |
| ACTN1-DT | 3.034576789 | -0.826528428 | 0.325005899 | -2.543118235 | 0.010986804 | NA |
| HTRA1 | 29.04637892 | 0.825591792 | 0.352014366 | 2.345335509 | 0.019009965 | 0.26978588 |
| LOC124901435 | 4.255344107 | 0.823656392 | 0.371268167 | 2.218494518 | 0.026521133 | 0.312615791 |
| PPIP5K1P1-CATSPER2 | 20.27642087 | -0.823583853 | 0.400380331 | -2.057003776 | 0.039685863 | 0.372140818 |
| LOC107984468 | 3.108685025 | 0.823323186 | 0.388194508 | 2.120903747 | 0.033929903 | 0.35021048 |
| NPM2 | 6.838728593 | 0.821465395 | 0.311777406 | 2.634781671 | 0.008419142 | 0.186307922 |
| GINS2 | 44.8243718 | 0.820877943 | 0.238285022 | 3.444941432 | 0.000571183 | 0.047630598 |
| BCAT1 | 226.0883877 | 0.820000993 | 0.197817137 | 4.145247508 | 3.39E-05 | 0.01061992 |
| LOC107985695 | 3.95687159 | 0.818221633 | 0.319861092 | 2.558053025 | 0.010526004 | 0.203804613 |
| LOC124902639 | 4.124687027 | 0.817827546 | 0.35518088 | 2.302566357 | 0.021303254 | 0.283736311 |
| OR10G2 | 12.26905847 | -0.817490932 | 0.307976125 | -2.6543971 | 0.007945027 | 0.181402317 |
| KHDRBS3 | 9.795529888 | 0.815536649 | 0.332506402 | 2.452694576 | 0.014179067 | 0.239811366 |
| PTGDR2 | 735.7353925 | 0.814708501 | 0.250123138 | 3.257229645 | 0.001125054 | 0.06973918 |
| LOC124905090 | 4.764410644 | 0.814321183 | 0.382728451 | 2.127673501 | 0.033364163 | 0.347975868 |
| SV2B | 4.903687305 | 0.813712506 | 0.383980167 | 2.119152433 | 0.034077587 | 0.350719276 |
| LOC124900728 | 2.638245198 | 0.813423662 | 0.397670631 | 2.045470793 | 0.040808466 | NA |
| LOC101929322 | 28.72777108 | 0.813314681 | 0.268297061 | 3.031396157 | 0.002434256 | 0.104313177 |
| LOC107985900 | 37.84049434 | -0.812731647 | 0.209087216 | -3.887046105 | 0.000101471 | 0.019419475 |
| PROS1 | 87.24781316 | 0.81269706 | 0.258096855 | 3.148806515 | 0.001639387 | 0.08201928 |
| EFCC1 | 4.670179187 | -0.811998719 | 0.269632015 | -3.011507065 | 0.002599544 | 0.107042579 |
| LOC124903192 | 22.17529839 | 0.811541524 | 0.397018694 | 2.044088947 | 0.040944761 | 0.376729189 |
| NOS1AP | 4.080358897 | 0.810986355 | 0.360939955 | 2.246873319 | 0.024648124 | 0.302342145 |
| LOC107986131 | 4.072727188 | -0.810385714 | 0.327004786 | -2.478207501 | 0.013204433 | 0.230341397 |
| OR2W3 | 316.0630831 | 0.810066689 | 0.360936953 | 2.244344015 | 0.024810276 | 0.303199421 |
| LIPH | 13.97249614 | 0.808365432 | 0.2677383 | 3.019237183 | 0.002534121 | 0.105936619 |
| SCARA5 | 11.09684329 | -0.808194574 | 0.283385234 | -2.851929027 | 0.00434548 | 0.139735898 |
| HP | 239.1451171 | 0.808139152 | 0.27649866 | 2.922759739 | 0.003469441 | 0.123002921 |
| VMO1 | 15.52993709 | 0.807982195 | 0.315234217 | 2.563117047 | 0.010373707 | 0.202461893 |
| LOC105369382 | 25.95937996 | 0.807711336 | 0.272644822 | 2.962503848 | 0.00305148 | 0.116121183 |
| CYB5R2 | 18.143761 | 0.807629629 | 0.291320633 | 2.772304938 | 0.005566087 | 0.156010441 |
| LOC124902679 | 3.573834431 | 0.80683866 | 0.357492354 | 2.256939628 | 0.024011847 | 0.29806367 |
| NECTIN2 | 143.4579006 | 0.8051377 | 0.233443347 | 3.448964004 | 0.000562742 | 0.047591088 |
| EGF | 86.75707505 | 0.804319734 | 0.247998002 | 3.243250861 | 0.001181741 | 0.071236945 |
| PPP1R3G | 13.72151925 | 0.80426821 | 0.281273667 | 2.859379691 | 0.004244704 | 0.137844487 |
| GP9 | 153.8950857 | 0.803005061 | 0.278628082 | 2.881996152 | 0.003951646 | 0.132025149 |
| LYPD6B | 6.966667773 | 0.801634278 | 0.375944126 | 2.132322923 | 0.032980309 | 0.346724185 |
| IER5L-AS1 | 11.85837866 | -0.801324561 | 0.248778109 | -3.221041284 | 0.001277257 | 0.073833723 |
| FAM27E4 | 4.87491437 | 0.801186933 | 0.33589141 | 2.385255795 | 0.01706725 | 0.258792017 |
| HMMR | 32.30354788 | 0.800657932 | 0.296532122 | 2.700071503 | 0.006932457 | 0.171890065 |
| ERBB3 | 14.72638476 | -0.800246724 | 0.218118294 | -3.668865686 | 0.000243629 | 0.032016102 |
| FHL1P1 | 6.259380095 | 0.79961873 | 0.314531186 | 2.542255795 | 0.011013954 | 0.208756782 |
| ADGRE4P | 1086.165197 | 0.799391085 | 0.257616927 | 3.103022356 | 0.001915552 | 0.09187191 |
| IGLV3-21 | 169.5030315 | 0.79918211 | 0.382491638 | 2.089410671 | 0.036670771 | 0.362160521 |
| ITGB8-AS1 | 3.545012139 | 0.798597235 | 0.348290971 | 2.29290249 | 0.021853619 | 0.287515558 |
| LOC124902749 | 1.560785691 | -0.798529875 | 0.387334203 | -2.061604342 | 0.03924542 | NA |
| ELAVL4 | 2.291880941 | 0.798417352 | 0.400863292 | 1.991744736 | 0.046399073 | NA |
| MPO | 179.0891681 | 0.798391869 | 0.215218877 | 3.709673986 | 0.000207526 | 0.029801836 |
| RAB3IL1 | 30.87392181 | 0.796789146 | 0.323232418 | 2.465065702 | 0.013698813 | 0.234401911 |
| LOC124909430 | 5.415645422 | 0.796551031 | 0.366817007 | 2.171521535 | 0.029891771 | 0.330339194 |
| SERPINE2 | 58.07866927 | -0.792283711 | 0.199212505 | -3.977078197 | 6.98E-05 | 0.015511558 |
| VWDE | 11.97545917 | 0.79198854 | 0.353949984 | 2.237571902 | 0.025248986 | 0.30464142 |
| NEAT1 | 758.1544072 | 0.791156705 | 0.242684551 | 3.260020884 | 0.00111404 | 0.069496072 |
| CLEC2L | 10.25472031 | 0.791119864 | 0.376015697 | 2.103954357 | 0.035382429 | 0.357304526 |
| SIAH3 | 6.449599664 | -0.791000825 | 0.29715257 | -2.661934992 | 0.007769288 | 0.180254393 |
| LINC02295 | 100.1422329 | -0.790332667 | 0.187927278 | -4.205523937 | 2.60E-05 | 0.009773444 |
| ITGB3 | 1285.146016 | 0.790255766 | 0.297191469 | 2.65907958 | 0.007835445 | 0.180254393 |
| IGHE | 5.832910857 | 0.788737842 | 0.364301342 | 2.165069825 | 0.030382338 | 0.332387751 |
| LINC02086 | 26.12326914 | 0.785837919 | 0.256433063 | 3.064495311 | 0.002180376 | 0.09593654 |
| LOC124905111 | 4.211944531 | 0.785541642 | 0.337888024 | 2.324857902 | 0.02007956 | 0.275440696 |
| SMARCA1 | 12.47772109 | -0.784933341 | 0.217312999 | -3.611994417 | 0.000303851 | 0.034787422 |
| SLC16A14 | 35.64399783 | 0.784879277 | 0.228902786 | 3.428876039 | 0.000606086 | 0.048984533 |
| GK3 | 5.387253246 | 0.784302124 | 0.370486433 | 2.116952349 | 0.034263892 | 0.351409907 |
| S100B | 127.1190286 | -0.783832371 | 0.341244474 | -2.296981875 | 0.021619804 | 0.286211935 |
| STPG4 | 3.43305099 | 0.782949679 | 0.367702485 | 2.129302113 | 0.033229273 | 0.347275732 |
| ZBTB20-AS1 | 14.39460708 | 0.782838015 | 0.215628925 | 3.630487033 | 0.000282887 | 0.033795557 |
| TPX2 | 96.70903218 | 0.782271503 | 0.253152218 | 3.090123048 | 0.002000736 | 0.093362898 |
| PDE7A-DT | 6.624360192 | -0.782017341 | 0.264316713 | -2.958637513 | 0.003090023 | 0.117135657 |
| CRACD | 11.19229201 | 0.781510577 | 0.288595779 | 2.707976467 | 0.006769482 | 0.169771035 |
| SEC14L3 | 5.726102182 | 0.78149754 | 0.362344987 | 2.156777572 | 0.031022999 | 0.335068467 |
| LOC105376568 | 107.8251532 | -0.779082121 | 0.318716386 | -2.444436985 | 0.014507842 | 0.240994289 |
| PCDHGB5 | 3.930842728 | 0.778298074 | 0.38487802 | 2.022194133 | 0.0431563 | 0.38756127 |
| LOC100134317 | 4.719240062 | -0.778294105 | 0.386206348 | -2.015228671 | 0.043880695 | 0.389528484 |
| LOC124902787 | 19.90436737 | 0.777920555 | 0.305780044 | 2.544052728 | 0.010957454 | 0.208487778 |
| CDC45 | 28.18914286 | 0.777049256 | 0.285994099 | 2.717011497 | 0.006587432 | 0.167550269 |
| BFSP2 | 9.132825865 | 0.774150815 | 0.24822944 | 3.118690579 | 0.001816566 | 0.088197421 |
| SEC14L4 | 10.19825662 | 0.771872185 | 0.37294171 | 2.069685865 | 0.038481771 | 0.368930494 |
| NPAS2 | 53.23823033 | -0.771742673 | 0.221251625 | -3.488076858 | 0.000486508 | 0.044193778 |
| CDC20 | 49.35968417 | 0.770705752 | 0.2952308 | 2.610519474 | 0.009040482 | 0.191403941 |
| DYNLT5 | 24.23047263 | 0.770513289 | 0.338906141 | 2.273530028 | 0.022994259 | 0.293754263 |
| IGSF11 | 4.426874183 | -0.77051234 | 0.337556521 | -2.282617256 | 0.022452924 | 0.291178967 |
| CFAP141 | 3.128446432 | 0.769051823 | 0.324517421 | 2.369832166 | 0.017796162 | 0.262847174 |
| HBA2 | 235189.852 | 0.768941431 | 0.314336285 | 2.44623821 | 0.014435559 | 0.240739202 |
| B4GALNT4 | 18.26530561 | -0.766853684 | 0.319104621 | -2.403141896 | 0.016254879 | 0.253259671 |
| SMPD3 | 454.9600601 | 0.766059187 | 0.279950009 | 2.736414224 | 0.006211279 | 0.164700379 |
| TMEM132C | 13.36301084 | -0.765510906 | 0.297286784 | -2.574991376 | 0.010024259 | 0.200403839 |
| UNC5B | 22.13886144 | 0.763045061 | 0.321340645 | 2.374567525 | 0.017569522 | 0.262400088 |
| KIF14 | 21.22440351 | 0.76209124 | 0.346577885 | 2.198903261 | 0.027884802 | 0.320691489 |
| ZSCAN2 | 2.700905858 | 0.762062349 | 0.314857471 | 2.420340693 | 0.015505972 | NA |
| LAMB2 | 80.14155317 | 0.761052747 | 0.22898467 | 3.323596935 | 0.000888645 | 0.061463076 |
| PDZK1IP1 | 545.7593272 | 0.760964184 | 0.283213356 | 2.686893705 | 0.00721199 | 0.175349788 |
| CACNG8 | 135.6183782 | 0.760368738 | 0.292359351 | 2.600801836 | 0.009300616 | 0.193186243 |
| SUMO1P3 | 6.505419997 | 0.758527727 | 0.300924685 | 2.52065638 | 0.011713618 | 0.21700366 |
| MELK | 33.57926589 | 0.758456151 | 0.240386957 | 3.155146849 | 0.001604173 | 0.080978003 |
| TINF2 | 56.53958403 | 0.757842102 | 0.384546042 | 1.970744774 | 0.048753076 | 0.405333161 |
| GPR152 | 5.340295993 | -0.756488046 | 0.234594928 | -3.224656442 | 0.001261239 | 0.0733379 |
| CCNB2 | 55.82249042 | 0.754553571 | 0.261554116 | 2.8848851 | 0.003915565 | 0.131937821 |
| CYP2J2 | 12.58822909 | -0.75237568 | 0.223570425 | -3.365273733 | 0.000764678 | 0.056880468 |
| SMIM24 | 116.4030551 | 0.751626139 | 0.281216498 | 2.672766868 | 0.007522851 | 0.179094736 |
| GFAP | 3.50089184 | 0.751470254 | 0.341678399 | 2.19934961 | 0.027853073 | 0.320633667 |
| CLDN5 | 93.2735648 | 0.751410316 | 0.280949225 | 2.674541338 | 0.007483155 | 0.178910723 |
| TBCE | 17.26359832 | 0.751335248 | 0.374885109 | 2.004174693 | 0.04505135 | 0.393120167 |
| LOC105370943 | 4.760860009 | 0.751240344 | 0.31793094 | 2.362904171 | 0.018132358 | 0.264827082 |
| TEAD3 | 9.025614713 | 0.749486556 | 0.31648232 | 2.368178276 | 0.01787592 | 0.263125193 |
| SKA3 | 14.7903614 | 0.744527088 | 0.328180862 | 2.268648707 | 0.023289697 | 0.294964762 |
| LOC105375589 | 7.941684876 | 0.742010093 | 0.322544147 | 2.300491576 | 0.021420386 | 0.284719251 |
| MMP9 | 2304.440941 | 0.741443239 | 0.340153308 | 2.179732558 | 0.029277292 | 0.328278711 |
| LINC00989 | 192.3598386 | 0.740543501 | 0.278633775 | 2.657766454 | 0.007866038 | 0.180717186 |
| CENPU | 58.19244516 | 0.740370933 | 0.17648278 | 4.195145453 | 2.73E-05 | 0.009773444 |
| SLC16A10 | 140.3127744 | -0.740289805 | 0.163805044 | -4.519334601 | 6.20E-06 | 0.004703153 |
| LINC01750 | 8.938111556 | 0.739308535 | 0.358473155 | 2.062381866 | 0.039171394 | 0.370963863 |
| CFAP43 | 5.313593722 | 0.739207791 | 0.287285052 | 2.573081282 | 0.010079753 | 0.200489302 |
| NBEA | 68.82680192 | -0.738670809 | 0.185158776 | -3.989391303 | 6.62E-05 | 0.015511558 |
| RNA45SN5 | 7.78053226 | -0.738380631 | 0.369664675 | -1.997433568 | 0.045778104 | 0.397357532 |
| DNAH6 | 52.6185224 | -0.737936703 | 0.194870388 | -3.786807798 | 0.000152595 | 0.02485912 |
| TPST1 | 321.684637 | 0.737799697 | 0.319354933 | 2.310281196 | 0.020872591 | 0.281038606 |
| CACNA1D | 38.68305565 | 0.737168392 | 0.179160522 | 4.114569345 | 3.88E-05 | 0.01061992 |
| PTPRF | 21.03218757 | 0.735791191 | 0.280847529 | 2.619895552 | 0.00879567 | 0.189279749 |
| IL21-AS1 | 5.7768292 | 0.735187744 | 0.300355236 | 2.44772741 | 0.014376037 | 0.240219696 |
| EPHA10 | 2.784344068 | 0.734591394 | 0.360031428 | 2.040353526 | 0.041315127 | NA |
| OR56B1 | 3.967713898 | 0.733838868 | 0.31472932 | 2.33165079 | 0.01971907 | 0.274229829 |
| THRB | 16.83943465 | 0.733641717 | 0.260324042 | 2.818186564 | 0.004829574 | 0.145850614 |
| KIF4A | 27.83735311 | 0.73356374 | 0.315097164 | 2.328055671 | 0.019909148 | 0.27489072 |
| OLFM1 | 99.80997196 | 0.732033911 | 0.260177394 | 2.813595366 | 0.004899085 | 0.146262666 |
| TRAJ22 | 3.16016019 | -0.731895402 | 0.309182324 | -2.367196782 | 0.0179234 | 0.263268303 |
| HES4 | 98.95292237 | 0.731836939 | 0.307796695 | 2.377663405 | 0.017422722 | 0.261765768 |
| HJURP | 45.87071973 | 0.731379582 | 0.27295525 | 2.679485302 | 0.007373544 | 0.177036906 |
| LPL | 48.08821753 | 0.731362888 | 0.314229104 | 2.327482974 | 0.019939574 | 0.27489072 |
| HYAL3 | 81.04797015 | 0.73089485 | 0.142610769 | 5.125102796 | 2.97E-07 | 0.000586186 |
| LOC101593348 | 10.13042758 | 0.730543384 | 0.235197196 | 3.106088836 | 0.001895798 | 0.091369097 |
| LINC01993 | 4.861819976 | -0.729970843 | 0.347699716 | -2.099428931 | 0.035779107 | 0.358747484 |
| RRN3P3 | 3.650021624 | -0.728195166 | 0.315358223 | -2.309104735 | 0.02093777 | 0.281072121 |
| LOC107986876 | 2.869509478 | 0.726708196 | 0.327239424 | 2.220723244 | 0.026369712 | NA |
| SLC35G2 | 41.22062747 | 0.72638462 | 0.219454655 | 3.309953116 | 0.000933116 | 0.062776732 |
| NCAPG | 60.40453859 | 0.725526414 | 0.265886995 | 2.728702151 | 0.006358411 | 0.164700379 |
| PF4 | 1252.713296 | 0.725308078 | 0.256457665 | 2.828178594 | 0.004681368 | 0.143855267 |
| LOC283028 | 19.13751852 | 0.724947809 | 0.353672046 | 2.049774128 | 0.040386477 | 0.374085878 |
| LOC124903725 | 5.407826066 | -0.724060905 | 0.227308331 | -3.185368967 | 0.001445696 | 0.078289975 |
| LOC112267986 | 4.346263252 | -0.723045663 | 0.337684343 | -2.141188 | 0.03225888 | 0.342796253 |
| DEPDC1B | 35.48506314 | 0.723028553 | 0.235862472 | 3.065466702 | 0.002173306 | 0.095839381 |
| ID1 | 3.588507158 | -0.722645699 | 0.353488869 | -2.044323773 | 0.040921572 | 0.376729189 |
| WFIKKN1 | 23.31634619 | 0.722336818 | 0.274196842 | 2.634373216 | 0.008429278 | 0.186307922 |
| LOC124902314 | 11.41182315 | 0.722231781 | 0.264038552 | 2.735327005 | 0.006231833 | 0.164700379 |
| PLEKHD1 | 18.16356807 | -0.718559585 | 0.291299001 | -2.46674236 | 0.013634842 | 0.234101472 |
| PKP2 | 50.93445863 | 0.717448703 | 0.191467509 | 3.747104188 | 0.000178888 | 0.027548721 |
| CCR8 | 37.88741648 | 0.716425629 | 0.197140646 | 3.634083816 | 0.00027897 | 0.033715775 |
| ALDH1A3-AS1 | 11.74788299 | 0.715438683 | 0.215168953 | 3.325008895 | 0.000884157 | 0.061367977 |
| GPC4 | 23.97921125 | 0.715425569 | 0.319896423 | 2.236428786 | 0.025323697 | 0.304738588 |
| VAC14-AS1 | 2.699913082 | -0.715308465 | 0.318240331 | -2.247698975 | 0.024595391 | NA |
| SEPT5-GP1BB | 28.65501933 | 0.715064401 | 0.225326777 | 3.17345507 | 0.001506362 | 0.079617828 |
| RDUR | 18.965085 | 0.714603511 | 0.254607523 | 2.806686553 | 0.005005392 | 0.146915366 |
| ARG1 | 263.2709799 | 0.71414548 | 0.363729204 | 1.963398791 | 0.049599854 | 0.408571804 |
| CKB | 105.1784529 | 0.712275245 | 0.200126745 | 3.559120716 | 0.000372098 | 0.038202111 |
| HSPG2 | 45.54458101 | -0.710208076 | 0.143431616 | -4.951544827 | 7.36E-07 | 0.000967552 |
| LOC105377267 | 467.1387999 | 0.708434472 | 0.326689746 | 2.168523745 | 0.03011886 | 0.330753743 |
| PIK3R6 | 689.0026415 | 0.708333257 | 0.186579873 | 3.796407652 | 0.000146808 | 0.024324274 |
| E2F2 | 9.145029426 | 0.707916091 | 0.329134915 | 2.150838635 | 0.031488938 | 0.337837162 |
| HOXB7 | 7.794571834 | 0.707748008 | 0.296012018 | 2.39094349 | 0.016805139 | 0.256924868 |
| LINC01655 | 1.738000745 | -0.707557551 | 0.356881548 | -1.982611751 | 0.047410814 | NA |
| PLCH1 | 10.14562044 | 0.707376698 | 0.292235097 | 2.420574068 | 0.015496022 | 0.248339502 |
| LOC124900692 | 6.468654474 | 0.706401478 | 0.345059156 | 2.047189489 | 0.040639483 | 0.375391514 |
| CDKN3 | 26.14145276 | 0.70572952 | 0.240170656 | 2.938450233 | 0.003298576 | 0.120334569 |
| NUDT9P1 | 8.218253978 | -0.705588908 | 0.208637354 | -3.381891568 | 0.000719886 | 0.054832287 |
| GJA3 | 14.75931941 | -0.705523287 | 0.26429635 | -2.66944015 | 0.007597781 | 0.179447145 |
| LOC124903833 | 9.657257571 | 0.705359644 | 0.267790936 | 2.633993717 | 0.008438705 | 0.186307922 |
| CNKSR3 | 14.61274923 | 0.705185431 | 0.324529579 | 2.172946554 | 0.02978434 | 0.330042436 |
| GPR82 | 78.08756449 | 0.705122342 | 0.228101102 | 3.091271089 | 0.001993016 | 0.093362898 |
| GUSBP14 | 4.604058939 | 0.704758193 | 0.28214064 | 2.497896772 | 0.012493257 | 0.225519302 |
| LOC101929098 | 7.068155397 | 0.704659369 | 0.241472442 | 2.918177177 | 0.003520842 | 0.123933649 |
| EDA2R | 9.293177133 | 0.703505203 | 0.272587661 | 2.580840228 | 0.009856018 | 0.198246771 |
| LOC107986854 | 5.768245248 | 0.703466374 | 0.29393033 | 2.393309921 | 0.01669713 | 0.256734661 |
| RRS1-DT | 4.070910644 | -0.70128308 | 0.330253429 | -2.123469491 | 0.033714529 | 0.349963562 |
| MEIS2 | 9.402268379 | 0.700827419 | 0.323940684 | 2.163443662 | 0.030507072 | 0.333285648 |
| EDAR | 145.5531347 | -0.700179874 | 0.175327412 | -3.993556203 | 6.51E-05 | 0.015511558 |
| DSC1 | 55.27729383 | -0.696765846 | 0.205449503 | -3.391421426 | 0.000695311 | 0.053960511 |
| CDK1 | 51.55440663 | 0.696645206 | 0.265208573 | 2.62678238 | 0.008619644 | 0.187746324 |
| ERG | 20.91906485 | 0.696274354 | 0.302366783 | 2.302747502 | 0.021293054 | 0.283736311 |
| LOC102724008 | 19.01074479 | 0.694477416 | 0.224509977 | 3.093303142 | 0.001979418 | 0.093362898 |
| AQP7 | 5.427600481 | -0.693907717 | 0.2883724 | -2.406290326 | 0.016115452 | 0.252669012 |
| LOC124901809 | 48.76199127 | -0.693170192 | 0.165800029 | -4.18076037 | 2.91E-05 | 0.009993553 |
| CLSTN3 | 67.83921245 | 0.689638462 | 0.339713789 | 2.030057314 | 0.042350714 | 0.383471415 |
| PDK4 | 787.9065076 | 0.688174858 | 0.158738506 | 4.335273611 | 1.46E-05 | 0.007841458 |
| LOC124906109 | 7.382947394 | 0.687813944 | 0.263751173 | 2.607813784 | 0.009112251 | 0.191403941 |
| DUSP13B | 14.68371104 | 0.687392257 | 0.349555112 | 1.966477485 | 0.049243481 | 0.406992468 |
| FOXD1 | 7.60946816 | 0.687293958 | 0.316645712 | 2.170545606 | 0.029965538 | 0.330358321 |
| PLXDC1 | 433.5837755 | -0.687022808 | 0.159782469 | -4.299738349 | 1.71E-05 | 0.008426874 |
| GFI1B | 422.6119374 | 0.686888717 | 0.189086702 | 3.632665378 | 0.000280509 | 0.033715775 |
| GRAPL | 53.03277493 | -0.686281049 | 0.251022587 | -2.733941423 | 0.006258118 | 0.164700379 |
| CHEK1 | 55.27346352 | 0.686190523 | 0.2051809 | 3.34431968 | 0.000824846 | 0.059340768 |
| PTPRB | 5.697219335 | -0.685659741 | 0.31763857 | -2.158616129 | 0.03087996 | 0.334923452 |
| NEIL3 | 16.73107695 | 0.685335167 | 0.297219272 | 2.30582345 | 0.021120499 | 0.282240104 |
| PKMYT1 | 30.6058123 | 0.685173469 | 0.281527728 | 2.433769038 | 0.014942526 | 0.24434832 |
| CALD1 | 48.16378076 | 0.684389507 | 0.284943234 | 2.40184509 | 0.016312615 | 0.253386068 |
| MACIR | 230.3969586 | 0.684299586 | 0.202118014 | 3.38564373 | 0.000710115 | 0.054678863 |
| ARHGEF4 | 46.26213245 | -0.684203053 | 0.173214963 | -3.950022806 | 7.81E-05 | 0.016563113 |
| LOC124904621 | 7.730232482 | -0.68397449 | 0.19564305 | -3.496032656 | 0.000472231 | 0.043702421 |
| STYK1 | 52.53274207 | 0.683865126 | 0.163006501 | 4.195324253 | 2.72E-05 | 0.009773444 |
| LOC645967 | 2.740232889 | -0.683459357 | 0.346604629 | -1.971870249 | 0.04862442 | NA |
| MYL4 | 859.7900131 | 0.683009609 | 0.260923903 | 2.617658261 | 0.008853542 | 0.189915642 |
| PDE3A | 15.85834978 | 0.682257472 | 0.278409689 | 2.450552184 | 0.014263729 | 0.240219477 |
| EXO1 | 25.51285186 | 0.681802464 | 0.246616791 | 2.764623047 | 0.005698861 | 0.157333258 |
| LOC105369322 | 12.90098657 | 0.681080185 | 0.295299666 | 2.306403502 | 0.021088096 | 0.282205402 |
| ALPK2 | 20.44116652 | 0.678600058 | 0.327913225 | 2.069450104 | 0.038503869 | 0.368930494 |
| CYP7B1 | 26.14827124 | 0.678506188 | 0.261119185 | 2.598453993 | 0.009364459 | 0.193488679 |
| RBM20 | 16.2523508 | -0.678501199 | 0.203610278 | -3.332352397 | 0.000861151 | 0.060409309 |
| LOC112268474 | 17.81727691 | 0.678380706 | 0.287433926 | 2.360127475 | 0.018268656 | 0.265304222 |
| RUNDC3A-AS1 | 7.501252305 | 0.677949679 | 0.311601595 | 2.175693865 | 0.029578159 | 0.329922295 |
| TROAP | 19.16373253 | 0.677623524 | 0.288270289 | 2.350653365 | 0.018740483 | 0.267690145 |
| ZNF667-AS1 | 74.37701161 | -0.677020035 | 0.164428596 | -4.117410533 | 3.83E-05 | 0.01061992 |
| ZNF718 | 174.1828844 | 0.676489848 | 0.214251154 | 3.15746187 | 0.00159149 | 0.080978003 |
| ZNF185 | 71.21272817 | -0.67534081 | 0.29208739 | -2.312119019 | 0.020771125 | 0.280055009 |
| PRSS8 | 3.815816212 | 0.675166911 | 0.342241976 | 1.972776452 | 0.048521037 | 0.405333161 |
| MINAR1 | 53.87891572 | 0.675079828 | 0.213880461 | 3.156341749 | 0.001597616 | 0.080978003 |
| SPARC | 2748.442738 | 0.674860395 | 0.268467586 | 2.513750003 | 0.011945508 | 0.219559818 |
| LOC124906363 | 4.765981103 | 0.674729021 | 0.271949635 | 2.481080807 | 0.013098468 | 0.229885608 |
| TNR | 12.59870823 | -0.67417196 | 0.211839556 | -3.182464936 | 0.001460272 | 0.078647216 |
| LOC105374869 | 18.56654769 | -0.672414839 | 0.202522505 | -3.32019812 | 0.000899536 | 0.061782756 |
| SEMA5A | 15.90179368 | 0.672105142 | 0.196403926 | 3.42205553 | 0.000621496 | 0.04980054 |
| MIR1285-1 | 2.48001601 | 0.671053556 | 0.32165043 | 2.086282164 | 0.036953071 | NA |
| KCNE1 | 78.31820227 | 0.670562752 | 0.2435752 | 2.753000929 | 0.005905173 | 0.160084069 |
| SPTA1 | 36.94656929 | 0.669542698 | 0.295040514 | 2.269324607 | 0.023248593 | 0.294964762 |
| ESCO2 | 31.07091552 | 0.668503111 | 0.22622081 | 2.955091137 | 0.003125767 | 0.117586105 |
| LINC02694 | 5.84971818 | 0.668467921 | 0.28952769 | 2.308822077 | 0.020953456 | 0.281072121 |
| SLC5A9 | 73.41932326 | 0.668418868 | 0.273001987 | 2.448402939 | 0.014349108 | 0.240219696 |
| ARHGAP29 | 19.24053462 | 0.668119404 | 0.280564248 | 2.381341918 | 0.017249693 | 0.259958672 |
| LOC124902709 | 10.93809737 | 0.667445759 | 0.259467582 | 2.572366662 | 0.010100586 | 0.200489302 |
| SPEG | 107.7038674 | -0.66688969 | 0.155830124 | -4.279594156 | 1.87E-05 | 0.008743942 |
| LINC00540 | 17.17045536 | -0.666169623 | 0.212925038 | -3.128657995 | 0.001756066 | 0.086108378 |
| COPG2IT1 | 13.22233572 | -0.664836775 | 0.235034139 | -2.828681735 | 0.004674016 | 0.143855267 |
| PCOLCE-AS1 | 4.006023655 | 0.663754587 | 0.31842722 | 2.084478165 | 0.037116694 | 0.363470669 |
| IGHV3-74 | 14.58680627 | 0.662868262 | 0.330744242 | 2.004171738 | 0.045051667 | 0.393120167 |
| HBQ1 | 246.4696466 | 0.662788399 | 0.281432773 | 2.355050521 | 0.018520188 | 0.266474407 |
| PKD2L1 | 6.018268415 | -0.661873935 | 0.308260564 | -2.147124907 | 0.031783339 | 0.340126588 |
| LOC101927401 | 52.56285177 | -0.661684848 | 0.153416006 | -4.313010513 | 1.61E-05 | 0.008139866 |
| SPNS3 | 313.0831935 | 0.661018002 | 0.221671063 | 2.981976964 | 0.002863935 | 0.111129717 |
| TLCD4 | 32.37326476 | 0.660520711 | 0.329218917 | 2.006326724 | 0.044821401 | 0.392811673 |
| ABLIM3 | 226.2737108 | 0.660358564 | 0.28877507 | 2.28675752 | 0.02220998 | 0.289935848 |
| EREG | 19.56457905 | 0.656937586 | 0.22599618 | 2.906852607 | 0.003650852 | 0.125813969 |
| SAMSN1 | 1419.68342 | 0.656777349 | 0.204116925 | 3.217652577 | 0.001292443 | 0.073845303 |
| PHGDH | 78.53929875 | -0.656431892 | 0.161614075 | -4.061724761 | 4.87E-05 | 0.012802678 |
| CALML6 | 4.361472258 | -0.655858227 | 0.278103892 | -2.358320927 | 0.018357815 | 0.265795799 |
| LINC02883 | 4.993502475 | -0.65559612 | 0.269870782 | -2.429296401 | 0.015128159 | 0.245639432 |
| NELL2 | 1742.465466 | -0.653828403 | 0.158663883 | -4.120839543 | 3.77E-05 | 0.01061992 |
| HRH4 | 250.1984657 | 0.653658986 | 0.262554618 | 2.489611464 | 0.012788281 | 0.228214905 |
| DRD3 | 4.92720024 | 0.653206378 | 0.308789194 | 2.115379655 | 0.034397602 | 0.352414521 |
| LOC105370668 | 10.06981252 | 0.652726252 | 0.322393174 | 2.024628012 | 0.042905576 | 0.38618742 |
| NRGN | 4134.655506 | 0.652690465 | 0.235054993 | 2.776756443 | 0.00549043 | 0.154831708 |
| LILRA5 | 17.52659296 | 0.652442202 | 0.260056358 | 2.508849262 | 0.012112516 | 0.220727151 |
| DZIP1L | 23.0323133 | 0.650795046 | 0.298035992 | 2.183612263 | 0.028990752 | 0.326993038 |
| GATA6 | 8.652164255 | 0.650789773 | 0.312779489 | 2.080666399 | 0.037464452 | 0.365412801 |
| ESPN | 232.2909834 | 0.650439747 | 0.259202257 | 2.509390758 | 0.012093962 | 0.220727151 |
| MTARC1 | 1865.211792 | 0.650300271 | 0.247155027 | 2.631143213 | 0.008509817 | 0.187091308 |
| ADGRA3 | 48.62628851 | -0.650066137 | 0.222177365 | -2.925888227 | 0.003434743 | 0.122095121 |
| LOC105374399 | 8.534106773 | 0.648894198 | 0.253583509 | 2.558897462 | 0.010500471 | 0.20352535 |
| PRG2 | 17.21421674 | 0.648302152 | 0.261215488 | 2.481867203 | 0.013069597 | 0.229614885 |
| FCGBP | 69.80306782 | -0.647952877 | 0.21510268 | -3.012295698 | 0.002592799 | 0.107042579 |
| LOC105376995 | 26.16558489 | 0.647522838 | 0.288754364 | 2.242469442 | 0.024931048 | 0.303733511 |
| UICLM | 155.9351277 | 0.647240821 | 0.214819738 | 3.012948567 | 0.002587228 | 0.107042579 |
| ESPNL | 9.879980299 | -0.647102056 | 0.242347619 | -2.670139934 | 0.007581964 | 0.179447145 |
| NRXN2 | 13.71389537 | 0.646766944 | 0.286859301 | 2.254648681 | 0.024155389 | 0.298527294 |
| NAGPA-AS1 | 2.98569886 | -0.646577228 | 0.324748703 | -1.991007881 | 0.046480021 | NA |
| LINC00853 | 11.00784046 | 0.646048426 | 0.288062807 | 2.242734606 | 0.024913934 | 0.303712715 |
| NPRL3 | 2307.159018 | 0.645786177 | 0.234643088 | 2.752206262 | 0.005919522 | 0.160084069 |
| ZNF667 | 37.4058254 | -0.645636944 | 0.228482868 | -2.82575648 | 0.004716911 | 0.143931497 |
| CDKN1C | 19.972157 | 0.645615299 | 0.31532675 | 2.047448557 | 0.040614063 | 0.375332587 |
| TRIP13 | 28.86080943 | 0.64242398 | 0.246042719 | 2.611026177 | 0.009027098 | 0.191403941 |
| KRT73-AS1 | 35.61363784 | -0.641747857 | 0.233984614 | -2.742692542 | 0.006093771 | 0.162764796 |
| HTRA4 | 6.73628326 | 0.640968675 | 0.316164198 | 2.027328452 | 0.042628834 | 0.384928799 |
| MKI67 | 306.4541409 | 0.640951428 | 0.259867028 | 2.466459218 | 0.013645627 | 0.234101472 |
| CCNA2 | 102.8094063 | 0.640872176 | 0.229342542 | 2.794388562 | 0.005199796 | 0.149512944 |
| COL9A2 | 552.2151113 | 0.640716878 | 0.23633046 | 2.711105792 | 0.006705923 | 0.16914422 |
| CALHM5 | 12.61190838 | 0.640099538 | 0.239349577 | 2.674329101 | 0.007487893 | 0.178910723 |
| GREM2 | 17.20639163 | 0.639788856 | 0.28073428 | 2.278983726 | 0.022668033 | 0.292429491 |
| CERCAM | 97.58083672 | 0.639682399 | 0.225864886 | 2.832146294 | 0.004623669 | 0.143756722 |
| NCAPH | 75.18575233 | 0.63674291 | 0.174480232 | 3.64936991 | 0.000262884 | 0.033217794 |
| TRAV39 | 27.69046108 | -0.636274929 | 0.172759151 | -3.683017219 | 0.00023049 | 0.031551463 |
| TK1 | 97.35479156 | 0.634143796 | 0.215140322 | 2.947582249 | 0.003202695 | 0.119116098 |
| BACE2 | 301.3532044 | 0.634065296 | 0.157497663 | 4.025871138 | 5.68E-05 | 0.014531781 |
| LOC101926935 | 7.390539647 | -0.634008657 | 0.255417452 | -2.482244854 | 0.013055753 | 0.229614885 |
| OBSCN | 1025.880456 | -0.633505819 | 0.127179702 | -4.981186523 | 6.32E-07 | 0.000921729 |
| FAM178B | 10.31738544 | -0.633294662 | 0.230099813 | -2.752260652 | 0.005918539 | 0.160084069 |
| NUP160 | 96.28336906 | -0.632708219 | 0.191125129 | -3.31043972 | 0.000931495 | 0.062776732 |
| PTX3 | 67.16797229 | 0.632530996 | 0.192404631 | 3.287504018 | 0.001010797 | 0.066416132 |
| MTUS2 | 5.914114486 | 0.632490068 | 0.259947323 | 2.433147073 | 0.01496822 | 0.24434832 |
| KLF1 | 106.0924887 | 0.631923654 | 0.312433427 | 2.022586573 | 0.04311579 | 0.387550591 |
| RETREG1-AS1 | 14.32981947 | -0.631778768 | 0.181977549 | -3.471740173 | 0.000517097 | 0.045214936 |
| SYT15-AS1 | 3.332680353 | -0.631416224 | 0.31755326 | -1.988378971 | 0.046769793 | 0.40081035 |
| LOC105372798 | 5.561418305 | -0.630083032 | 0.256987372 | -2.451805424 | 0.01421415 | 0.240093681 |
| CHIT1 | 43.31507024 | 0.629835047 | 0.281488916 | 2.237512781 | 0.025252845 | 0.30464142 |
| POLQ | 35.24683905 | 0.629754934 | 0.234047856 | 2.69071011 | 0.007130012 | 0.175349788 |
| GGNBP1 | 5.739947841 | -0.628867061 | 0.207282988 | -3.033857567 | 0.002414484 | 0.103944003 |
| NUAK1 | 35.13011242 | 0.628057492 | 0.286789635 | 2.189958828 | 0.028527223 | 0.324670102 |
| INSC | 28.00810607 | -0.627193502 | 0.245322687 | -2.556606198 | 0.01056988 | 0.204068049 |
| LOC124902942 | 4.195382623 | -0.62699382 | 0.260409534 | -2.407722218 | 0.01605239 | 0.252533684 |
| GPR173 | 5.790261323 | -0.626764121 | 0.262980405 | -2.38331111 | 0.017157687 | 0.259487459 |
| FAM118A | 1708.628671 | -0.626762744 | 0.28664269 | -2.186564548 | 0.028774328 | 0.326539752 |
| SLC25A39 | 15659.147 | 0.626506672 | 0.276335013 | 2.267199752 | 0.023378026 | 0.294964762 |
| EZHIP | 5.923856217 | -0.626256696 | 0.283210754 | -2.211274422 | 0.027016839 | 0.316538665 |
| HRK | 80.98325143 | 0.626092034 | 0.264113951 | 2.370537532 | 0.01776224 | 0.262847174 |
| RUNDC3A | 496.1909922 | 0.625989657 | 0.317344694 | 1.972585867 | 0.048542765 | 0.405333161 |
| LOC124902908 | 92.97990541 | 0.625859948 | 0.306226868 | 2.043778692 | 0.040975415 | 0.376729189 |
| CELA1 | 13.52995577 | -0.625846254 | 0.226800528 | -2.759456774 | 0.005789755 | 0.158731075 |
| FPR3 | 67.6728666 | 0.625570623 | 0.250637995 | 2.495912971 | 0.012563342 | 0.226187432 |
| SEC14L5 | 54.30493021 | 0.625276414 | 0.274924498 | 2.274356846 | 0.02294454 | 0.293754263 |
| TKTL1 | 260.8999994 | 0.625062808 | 0.279092183 | 2.239628504 | 0.025115051 | 0.303909072 |
| LOC105378005 | 15.14990438 | -0.624326906 | 0.183375841 | -3.404630099 | 0.000662537 | 0.051825142 |
| HBB | 2652096.302 | 0.62306389 | 0.277736833 | 2.243360676 | 0.024873566 | 0.303432688 |
| SLC24A3 | 107.9822791 | 0.62263913 | 0.20248011 | 3.075063175 | 0.00210458 | 0.093730735 |
| EPPK1 | 71.2102322 | -0.620541696 | 0.178426067 | -3.477864566 | 0.000505425 | 0.044736474 |
| VCAN-AS1 | 7.845604831 | 0.620217704 | 0.245961307 | 2.5216068 | 0.011682021 | 0.216832384 |
| SUCNR1 | 33.61176251 | 0.619960703 | 0.179547572 | 3.452904961 | 0.000554584 | 0.047401697 |
| SMG1P3 | 29.51672203 | 0.619837463 | 0.238561444 | 2.598229847 | 0.009370575 | 0.193488679 |
| PDGFRB | 177.5137182 | 0.61977247 | 0.232225134 | 2.668843202 | 0.007611297 | 0.179466379 |
| LOC112268067 | 464.3841786 | -0.619619805 | 0.297012181 | -2.086176411 | 0.036962646 | 0.363033225 |
| FAM86B1 | 5.317611212 | -0.618683795 | 0.311318019 | -1.987304805 | 0.04688863 | 0.40081035 |
| E2F7 | 16.22225314 | 0.618029561 | 0.271715198 | 2.274549108 | 0.022932992 | 0.293754263 |
| LOC124904930 | 5.117689895 | 0.617938355 | 0.29560874 | 2.09039271 | 0.036582537 | 0.361823871 |
| TRAJ24 | 2.490449523 | -0.617893919 | 0.311597852 | -1.98298517 | 0.047369086 | NA |
| TDRP | 51.13337726 | 0.617734982 | 0.276060129 | 2.237682729 | 0.025241752 | 0.30464142 |
| FDPSP2 | 10.88606896 | 0.617522207 | 0.215892822 | 2.86031838 | 0.004232159 | 0.137663888 |
| DCHS1 | 308.9073701 | -0.617226546 | 0.146935423 | -4.200665374 | 2.66E-05 | 0.009773444 |
| ACBD3-AS1 | 3.298464307 | 0.614914325 | 0.30903251 | 1.98980465 | 0.046612459 | 0.400386606 |
| SLC22A16 | 63.86348771 | 0.614865202 | 0.253168527 | 2.428679462 | 0.015153923 | 0.245855256 |
| LOC105374426 | 10.37940715 | 0.614652616 | 0.189641559 | 3.241128259 | 0.001190576 | 0.071333236 |
| C4BPB | 8.64746782 | -0.614431957 | 0.254559247 | -2.413709041 | 0.015791067 | 0.250622792 |
| ADIRF | 2.504409126 | -0.614378052 | 0.306778764 | -2.002674646 | 0.045212222 | NA |
| MT1E | 36.91902917 | 0.613526296 | 0.224815711 | 2.729018776 | 0.006352309 | 0.164700379 |
| LOC283194 | 13.10686793 | 0.61331119 | 0.1992742 | 3.077725012 | 0.002085873 | 0.093730735 |
| ACOT11 | 42.71969025 | 0.61278288 | 0.242093522 | 2.531182474 | 0.011367869 | 0.213209741 |
| RIMKLA | 12.5857532 | 0.612782322 | 0.242991522 | 2.52182593 | 0.011674746 | 0.216832384 |
| CDCA2 | 21.7094144 | 0.612343053 | 0.303058514 | 2.020543969 | 0.043326994 | 0.387681211 |
| EFCAB8 | 41.51301764 | 0.611796387 | 0.206919592 | 2.956686608 | 0.00310964 | 0.117427632 |
| SPC24 | 22.39070787 | 0.611169427 | 0.267124385 | 2.287958197 | 0.022139956 | 0.28978938 |
| LOC124900390 | 6.483113163 | 0.609958617 | 0.287949767 | 2.118281337 | 0.034151248 | 0.350719276 |
| DQX1 | 6.1489215 | 0.609690914 | 0.299235464 | 2.037495507 | 0.041600412 | 0.379924655 |
| LOC124902457 | 5.766678058 | 0.609209639 | 0.310448782 | 1.96235152 | 0.049721573 | 0.40923242 |
| TRAJ35 | 3.299467343 | -0.609189831 | 0.276424032 | -2.203823695 | 0.027536745 | 0.319061291 |
| TTC7B | 194.1201115 | 0.60896497 | 0.174290799 | 3.493959372 | 0.000475913 | 0.04383741 |
| CROCC2 | 77.48304752 | 0.608942121 | 0.228875485 | 2.660582543 | 0.00780056 | 0.180254393 |
| GDF10 | 6.936914725 | -0.608215031 | 0.298349914 | -2.038596297 | 0.041490335 | 0.379692426 |
| HIGD1B | 5.344213579 | -0.607849993 | 0.243873771 | -2.492477934 | 0.012685521 | 0.22711807 |
| TMEM40 | 156.4301601 | 0.607697218 | 0.218805379 | 2.777341311 | 0.005480559 | 0.154774768 |
| LINC02615 | 20.58900389 | -0.606514595 | 0.179009935 | -3.38816164 | 0.000703628 | 0.054391812 |
| HMGB3 | 56.88604742 | 0.606062939 | 0.145190833 | 4.174250709 | 2.99E-05 | 0.009993553 |
| SAP30 | 230.9986865 | 0.605216927 | 0.201837124 | 2.998541177 | 0.002712755 | 0.108425688 |
| DUSP4 | 43.39402622 | 0.605065297 | 0.178928227 | 3.381608961 | 0.000720626 | 0.054832287 |
| FRRS1 | 245.8314359 | 0.603426964 | 0.160999818 | 3.747997793 | 0.000178252 | 0.027548721 |
| KNL1 | 66.39161396 | 0.603331654 | 0.217676104 | 2.771694474 | 0.005576535 | 0.156010441 |
| TFPI | 16.59159532 | 0.603083412 | 0.255545574 | 2.359983794 | 0.018275733 | 0.265304222 |
| PRSS57 | 40.42542729 | 0.602860154 | 0.289567573 | 2.081932542 | 0.037348632 | 0.364899751 |
| HEATR6-DT | 5.403401642 | 0.602637824 | 0.291230078 | 2.069284283 | 0.038519418 | 0.368930494 |
| LMCD1 | 8.24311903 | 0.600908923 | 0.262772669 | 2.286801462 | 0.022207414 | 0.289935848 |
| LOC105372879 | 36.14614723 | -0.600489374 | 0.130625899 | -4.597016203 | 4.29E-06 | 0.003840115 |
| LOC107987228 | 31.07938151 | 0.598120287 | 0.283092815 | 2.112806312 | 0.034617349 | 0.354070238 |
| ARHGEF38 | 4.417810458 | 0.597745601 | 0.263513983 | 2.268363878 | 0.023307038 | 0.294964762 |
| LINC00563 | 15.20542799 | -0.597599689 | 0.244717705 | -2.441996135 | 0.014606303 | 0.241745965 |
| ZNF135 | 51.86411604 | -0.596718159 | 0.206174797 | -2.894234255 | 0.003800845 | 0.1291763 |
| ITGB5 | 363.596104 | 0.596097867 | 0.231623503 | 2.573563814 | 0.010065708 | 0.200489302 |
| AVPR1A | 21.60824813 | 0.595985234 | 0.292553101 | 2.037186519 | 0.041631354 | 0.379924655 |
| LOC124901419 | 10.92702996 | 0.59580625 | 0.218693153 | 2.724393711 | 0.006441966 | 0.165987045 |
| FAXDC2 | 483.2678821 | 0.594545788 | 0.244856417 | 2.428140523 | 0.015176462 | 0.246018432 |
| LOC101929045 | 13.75138018 | -0.594419582 | 0.29116927 | -2.041491471 | 0.041202001 | 0.377796255 |
| TRABD2A | 1068.344557 | -0.593138581 | 0.124650438 | -4.758415538 | 1.95E-06 | 0.001923088 |
| CCDC163 | 46.25639829 | 0.593090235 | 0.221990129 | 2.671696429 | 0.007546889 | 0.179239623 |
| NUSAP1 | 178.3179083 | 0.593071111 | 0.158581338 | 3.739854383 | 0.000184127 | 0.027706178 |
| TMCC2 | 507.8240077 | 0.592101215 | 0.255808558 | 2.314626297 | 0.020633392 | 0.27934439 |
| CTDSPL | 126.2896471 | 0.590513682 | 0.243823956 | 2.421885416 | 0.015440218 | 0.247876513 |
| GATM | 58.2695176 | -0.589487747 | 0.186442728 | -3.16176315 | 0.001568171 | 0.080738272 |
| LINC00997 | 51.77402599 | 0.589339333 | 0.268704683 | 2.193260369 | 0.028288625 | 0.323409735 |
| LOC124900465 | 12.38424301 | 0.586828582 | 0.213619046 | 2.747079875 | 0.006012849 | 0.161611496 |
| RORB | 8.483087893 | 0.586572075 | 0.281286596 | 2.085318261 | 0.037040421 | 0.363254118 |
| LOC105373488 | 21.84242028 | -0.586354529 | 0.203330896 | -2.883745365 | 0.003929764 | 0.132025149 |
| LOC124901570 | 5.209137465 | 0.585174903 | 0.296770955 | 1.971806515 | 0.048631698 | 0.405333161 |
| LOC124902699 | 12.89804853 | 0.585159194 | 0.216062157 | 2.708291002 | 0.006763069 | 0.169771035 |
| LOC105373386 | 6.786191608 | 0.584894982 | 0.271447177 | 2.154728546 | 0.031183083 | 0.33594466 |
| MFSD2B | 82.72070949 | 0.584440593 | 0.258919691 | 2.257227288 | 0.023993876 | 0.29806367 |
| IRAK3 | 3667.760667 | 0.584403076 | 0.211195116 | 2.767124008 | 0.005655324 | 0.156569865 |
| LOC105369147 | 6.631061351 | 0.58412964 | 0.25051249 | 2.331738588 | 0.019714447 | 0.274229829 |
| CYP4F12 | 206.0811727 | 0.583953724 | 0.226083401 | 2.582912857 | 0.009797007 | 0.19771552 |
| TRGJP1 | 7.545241445 | 0.583786049 | 0.289287625 | 2.018012516 | 0.043589958 | 0.388891528 |
| IGHJ6 | 12.13461848 | -0.583781731 | 0.225505213 | -2.588772666 | 0.009631866 | 0.19553382 |
| AP3S2 | 4.90363844 | 0.583141066 | 0.262699517 | 2.219802578 | 0.026432172 | 0.311997131 |
| CYSLTR2 | 550.7419156 | 0.580823479 | 0.205461641 | 2.826919304 | 0.004699817 | 0.143855267 |
| JDP2-AS1 | 31.01166684 | 0.58080457 | 0.19950379 | 2.911245802 | 0.003599907 | 0.124583992 |
| USP7-AS1 | 20.58251572 | 0.5806462 | 0.284998154 | 2.037368284 | 0.04161315 | 0.379924655 |
| KLHL29 | 26.85642597 | -0.580350987 | 0.184907116 | -3.138608188 | 0.001697523 | 0.083863572 |
| TMEM273 | 1624.867502 | 0.580278302 | 0.152292496 | 3.810288202 | 0.000138805 | 0.024324274 |
| LOC124908011 | 26.66521462 | 0.58013041 | 0.271656117 | 2.135532295 | 0.032717555 | 0.345471275 |
| LOC124902052 | 33.10584536 | -0.579558265 | 0.135554539 | -4.275461886 | 1.91E-05 | 0.008743942 |
| MIR641 | 7.973430282 | -0.579320982 | 0.247410553 | -2.341537073 | 0.019204519 | 0.270806197 |
| MGLL | 389.2340269 | 0.578951335 | 0.212967735 | 2.718493187 | 0.006558001 | 0.167017195 |
| LOC107984192 | 21.09136845 | 0.578688951 | 0.264751393 | 2.185782459 | 0.028831525 | 0.326993038 |
| ANKRD18A | 14.38106442 | 0.578659904 | 0.243757799 | 2.373913397 | 0.017600678 | 0.262439159 |
| TRPC2 | 19.40690561 | 0.578644368 | 0.229549742 | 2.520779872 | 0.011709508 | 0.21700366 |
| GPR153 | 109.6468959 | 0.577871282 | 0.185921914 | 3.108139692 | 0.001882691 | 0.090959795 |
| TRNP1 | 22.02950316 | 0.577536139 | 0.267706639 | 2.157347091 | 0.03097863 | 0.335068467 |
| NT5E | 461.7225901 | -0.577351067 | 0.160964633 | -3.586819388 | 0.000334736 | 0.03582433 |
| IGSF22 | 10.68982973 | -0.576596007 | 0.199641531 | -2.888156601 | 0.003875069 | 0.131021199 |
| SLC34A1 | 6.736628675 | 0.576189628 | 0.292626545 | 1.969027204 | 0.048949967 | 0.405931279 |
| LINC02288 | 104.8852348 | 0.575596667 | 0.278221047 | 2.0688466 | 0.038560484 | 0.368930494 |
| LOC105369608 | 6.472282415 | -0.575281811 | 0.269327821 | -2.135991039 | 0.032680144 | 0.345410726 |
| LOC107984214 | 9.092702751 | -0.575221681 | 0.238977385 | -2.407013032 | 0.016083596 | 0.252621388 |
| LOC105376093 | 6.042422111 | 0.57501814 | 0.272604826 | 2.109346881 | 0.034914651 | 0.3553111 |
| PLLP | 19.74168916 | -0.574960915 | 0.210263856 | -2.73447337 | 0.006248015 | 0.164700379 |
| VSIG2 | 200.8176708 | 0.574775398 | 0.217795356 | 2.639061771 | 0.008313583 | 0.18496315 |
| ANKHD1-EIF4EBP3 | 8.285208199 | 0.574357366 | 0.21226758 | 2.70581765 | 0.006813645 | 0.170366407 |
| TXNRD3 | 24.0562881 | -0.574184619 | 0.165032019 | -3.479231611 | 0.000502854 | 0.044736474 |
| LOC107984356 | 16.67485389 | -0.5737949 | 0.203305804 | -2.822324246 | 0.004767695 | 0.144808624 |
| SLC6A4 | 22.12967783 | 0.573340329 | 0.203522825 | 2.817081223 | 0.004846227 | 0.145850614 |
| GALNT16 | 7.225698005 | -0.573145611 | 0.241965522 | -2.368707763 | 0.017850352 | 0.263125193 |
| GPRC5D-AS1 | 12.41974434 | -0.573139901 | 0.268549375 | -2.134206797 | 0.032825857 | 0.345471275 |
| CLEC1B | 56.77235049 | 0.572941474 | 0.214913491 | 2.665916748 | 0.00767787 | 0.179541636 |
| LMNA | 507.2884562 | 0.572639961 | 0.144553885 | 3.961429054 | 7.45E-05 | 0.015962975 |
| RNR1 | 39531.39437 | 0.57160158 | 0.20755179 | 2.754019039 | 0.005886834 | 0.160084069 |
| SNORD21 | 4.588329325 | -0.57129585 | 0.250565681 | -2.280024332 | 0.022606245 | 0.292206104 |
| LINC02970 | 83.06380691 | 0.571097369 | 0.220026654 | 2.59558267 | 0.009443068 | 0.19389767 |
| SLC18A2-AS1 | 108.2790468 | 0.571055707 | 0.249969692 | 2.284499784 | 0.022342175 | 0.290955516 |
| LOC124904314 | 42.11303382 | 0.571014018 | 0.218979943 | 2.607608759 | 0.00911771 | 0.191403941 |
| LEFTY1 | 16.64113844 | 0.5699586 | 0.263527483 | 2.162805158 | 0.030556169 | 0.333327724 |
| CDCA5 | 54.32543863 | 0.569918102 | 0.243902724 | 2.336661486 | 0.019456793 | 0.272201772 |
| AK5 | 204.54282 | -0.568552523 | 0.163853007 | -3.469893732 | 0.000520664 | 0.045214936 |
| PDGFA | 16.23472638 | 0.5680267 | 0.221967585 | 2.559052478 | 0.01049579 | 0.20352535 |
| LINC02446 | 405.8712484 | -0.567218593 | 0.184428095 | -3.075554144 | 0.002101118 | 0.093730735 |
| CD9 | 693.0507743 | 0.566284697 | 0.183607391 | 3.084215144 | 0.002040899 | 0.093362898 |
| TUBA8 | 226.3051071 | 0.565878539 | 0.198854182 | 2.845695949 | 0.004431449 | 0.140828335 |
| TMEM50B | 55.15167397 | 0.564422863 | 0.24848883 | 2.271421469 | 0.023121476 | 0.29456331 |
| HROB | 26.15992789 | 0.564389989 | 0.274320166 | 2.05741341 | 0.039646476 | 0.372140818 |
| LOC105369914 | 17.60653239 | 0.563029901 | 0.252836594 | 2.226852896 | 0.025957107 | 0.308790881 |
| LOC101927344 | 27.45465814 | 0.562847818 | 0.20702659 | 2.718722349 | 0.006553459 | 0.167017195 |
| LOC124904029 | 4.768440822 | -0.562456364 | 0.273199127 | -2.058778046 | 0.039515506 | 0.371922854 |
| DACH1 | 124.3276933 | 0.561107885 | 0.197753161 | 2.837415508 | 0.004548038 | 0.1425293 |
| CCR7 | 4642.827002 | -0.560473446 | 0.127337966 | -4.401463785 | 1.08E-05 | 0.006837076 |
| LOC105375024 | 10.50614688 | -0.560387066 | 0.210563165 | -2.661372735 | 0.007782275 | 0.180254393 |
| LOC112268199 | 198.4315527 | -0.560235471 | 0.132767124 | -4.219685224 | 2.45E-05 | 0.009676233 |
| RAB44 | 800.8903205 | 0.560150582 | 0.199259433 | 2.811162179 | 0.00493629 | 0.146262666 |
| PCDHGA7 | 6.46021064 | 0.559736396 | 0.265542566 | 2.107897068 | 0.035039894 | 0.356038226 |
| CXCL9 | 18.23294372 | 0.559664845 | 0.258908514 | 2.161631678 | 0.030646578 | 0.333759856 |
| CENPM | 33.90442261 | 0.559493365 | 0.210884415 | 2.65308067 | 0.007976081 | 0.181402317 |
| CCDC116 | 5.139009509 | 0.559086878 | 0.277061981 | 2.017912653 | 0.043600359 | 0.388891528 |
| LOC105377921 | 10.31710878 | 0.558941448 | 0.271650081 | 2.057578799 | 0.039630584 | 0.372140818 |
| C3AR1 | 1263.756038 | 0.558765122 | 0.208380937 | 2.681459875 | 0.00733017 | 0.176470153 |
| TMIGD2 | 286.5608257 | -0.558115658 | 0.140160295 | -3.981981196 | 6.83E-05 | 0.015511558 |
| GFRA2 | 45.99313921 | 0.558089218 | 0.212697611 | 2.623862181 | 0.008693896 | 0.188323155 |
| LOC124905203 | 7.897430233 | 0.55778273 | 0.242981992 | 2.295572297 | 0.021700348 | 0.286316777 |
| LOC107985876 | 134.532791 | -0.557618988 | 0.16789306 | -3.32127479 | 0.000896073 | 0.061760085 |
| GUK1 | 8090.398792 | 0.556574042 | 0.175573486 | 3.170034693 | 0.001524207 | 0.079617828 |
| LOC101927610 | 7.791785735 | -0.556015552 | 0.253592255 | -2.192557308 | 0.02833929 | 0.323652423 |
| LRRC63 | 14.87531898 | 0.55589079 | 0.22345441 | 2.487714568 | 0.012856687 | 0.228728356 |
| ELOVL7 | 113.9031727 | 0.555769493 | 0.246384886 | 2.25569637 | 0.024089653 | 0.298336333 |
| ZNF578 | 25.42364449 | -0.555410768 | 0.157802329 | -3.5196614 | 0.000432098 | 0.041364025 |
| LOC102723750 | 304.2103197 | 0.555117418 | 0.18831419 | 2.947825742 | 0.003200174 | 0.119116098 |
| CD70 | 40.61647072 | 0.554613076 | 0.183056522 | 3.029736762 | 0.00244767 | 0.104660446 |
| ZNF462 | 5.456625286 | -0.553832112 | 0.257115024 | -2.154024704 | 0.031238235 | 0.336301519 |
| HGD | 57.9426485 | 0.553810671 | 0.193813162 | 2.857446143 | 0.004270651 | 0.138005037 |
| GAS2L1 | 338.6097457 | 0.553710132 | 0.171021282 | 3.23766799 | 0.00120511 | 0.071477518 |
| LOC124907727 | 20.04279215 | 0.553597666 | 0.258615775 | 2.140618323 | 0.03230483 | 0.342914813 |
| LOC100287036 | 61.92733279 | 0.553043053 | 0.23631606 | 2.340268591 | 0.019269876 | 0.271126194 |
| TNNT3 | 11.32539326 | -0.552867509 | 0.280807433 | -1.968849267 | 0.048970403 | 0.405931279 |
| ZNF391 | 47.8202084 | -0.552695498 | 0.169868695 | -3.253663065 | 0.001139273 | 0.069756969 |
| RBFOX3 | 9.442543748 | -0.552170089 | 0.219760616 | -2.512598023 | 0.011984581 | 0.219883033 |
| C1orf226 | 16.55448183 | 0.551990442 | 0.278125274 | 1.984682776 | 0.047179776 | 0.401557751 |
| FUT2 | 11.19414691 | -0.551762916 | 0.200014523 | -2.758614266 | 0.005804701 | 0.158919807 |
| TFE3 | 114.8781194 | 0.550522733 | 0.252938804 | 2.176505636 | 0.029517473 | 0.329922295 |
| ADGRE1 | 2223.838695 | 0.549775146 | 0.199094159 | 2.761382603 | 0.005755721 | 0.158030123 |
| USP44 | 81.32003621 | -0.548354713 | 0.163801155 | -3.347685259 | 0.000814895 | 0.059055921 |
| SORCS2 | 26.1238681 | 0.548230829 | 0.17359935 | 3.158023513 | 0.001588427 | 0.080978003 |
| TOMM20L-DT | 4.63608524 | 0.547988022 | 0.247748157 | 2.211875269 | 0.026975285 | 0.316538665 |
| CUBN | 200.773149 | -0.547940444 | 0.164285889 | -3.335286108 | 0.000852117 | 0.060204036 |
| LIF | 9.317940838 | 0.547938209 | 0.265851699 | 2.061067173 | 0.039296632 | 0.371339986 |
| TGFB1I1 | 24.36869115 | 0.547897137 | 0.244031483 | 2.245190378 | 0.024755914 | 0.303098489 |
| GTSE1 | 47.35382625 | 0.547806567 | 0.241632247 | 2.267108691 | 0.023383587 | 0.294964762 |
| GNAI1 | 31.09320233 | -0.547034459 | 0.155232502 | -3.523968576 | 0.000425135 | 0.041364025 |
| ENO1-AS1 | 3.77435156 | -0.546967507 | 0.274826846 | -1.9902259 | 0.046566056 | 0.400309684 |
| TYMS | 31.63229936 | 0.546616504 | 0.26328355 | 2.076151373 | 0.037879953 | 0.366588995 |
| LOC124907872 | 5.801332239 | -0.54621655 | 0.254181222 | -2.148925656 | 0.031640294 | 0.338963842 |
| LINC02421 | 7.165899323 | -0.545996929 | 0.201359932 | -2.711547043 | 0.006697004 | 0.16914422 |
| BAG6 | 39.75034878 | 0.545648938 | 0.246712015 | 2.211683683 | 0.026988529 | 0.316538665 |
| MYOSLID | 21.31358566 | 0.545406705 | 0.221327448 | 2.464252444 | 0.013729937 | 0.234730721 |
| METTL21EP | 12.24733442 | 0.545197345 | 0.219421044 | 2.484708555 | 0.012965754 | 0.229229811 |
| SATB2 | 28.49892365 | 0.544985239 | 0.193278534 | 2.819688394 | 0.00480703 | 0.145554814 |
| IRS2 | 1549.675975 | 0.544739905 | 0.25601345 | 2.127778465 | 0.033355456 | 0.347975868 |
| LOC112694756 | 8.166855316 | 0.544667196 | 0.263336141 | 2.068334388 | 0.03860859 | 0.368930494 |
| VEPH1 | 32.90517317 | 0.544542026 | 0.254516862 | 2.139512573 | 0.032394179 | 0.343567003 |
| G0S2 | 50.1450432 | -0.543299088 | 0.268105385 | -2.026438551 | 0.042719864 | 0.385397691 |
| CARMIL1 | 136.1322171 | -0.543146156 | 0.140527208 | -3.86506047 | 0.000111062 | 0.020653282 |
| TAFA2 | 178.3005946 | 0.543019193 | 0.254343432 | 2.134984136 | 0.032762306 | 0.345471275 |
| FAM111B | 84.65492274 | 0.542704921 | 0.225449957 | 2.407207912 | 0.016075015 | 0.252621388 |
| LOC112268090 | 35.4345785 | -0.542609427 | 0.207289881 | -2.61763587 | 0.008854123 | 0.189915642 |
| MYEOV | 18.16410127 | 0.542596683 | 0.254176355 | 2.134725249 | 0.032783459 | 0.345471275 |
| RAD51 | 39.3526771 | 0.542149512 | 0.201991833 | 2.68401699 | 0.007274341 | 0.175555308 |
| LOC124902519 | 11.57119841 | -0.541998931 | 0.261309523 | -2.074164485 | 0.038064037 | 0.367262998 |
| LOC100132249 | 49.55414753 | -0.541166081 | 0.226759719 | -2.386517694 | 0.017008789 | 0.258104116 |
| GRB10 | 291.4452825 | 0.54086824 | 0.208545658 | 2.593524336 | 0.009499782 | 0.194275683 |
| ADAM12 | 73.63756131 | -0.539370762 | 0.206289779 | -2.614626687 | 0.008932503 | 0.190560058 |
| LOC105375588 | 25.75383625 | 0.538071874 | 0.266507035 | 2.018978123 | 0.043489493 | 0.388606026 |
| ZNF662 | 65.23019198 | -0.537825999 | 0.16508845 | -3.257805127 | 0.001122775 | 0.06973918 |
| KIAA1143 | 10.79377653 | 0.537053904 | 0.22269425 | 2.411619989 | 0.015881825 | 0.251254041 |
| LOC124901207 | 8.274337135 | 0.536534555 | 0.202619393 | 2.647992116 | 0.008097141 | 0.182673117 |
| MS4A7 | 1721.928959 | 0.53540477 | 0.157029008 | 3.409591501 | 0.000650602 | 0.051094328 |
| LDB2 | 15.29760368 | -0.535294848 | 0.246345203 | -2.172946099 | 0.029784374 | 0.330042436 |
| TRBV6-3 | 4.341486569 | -0.534678248 | 0.255048216 | -2.096381058 | 0.036048401 | 0.360091972 |
| TRR-ACG2-1 | 4.630984199 | -0.533994199 | 0.22534337 | -2.369691192 | 0.017802948 | 0.262847174 |
| FKBP8 | 50394.20377 | 0.533819438 | 0.23458326 | 2.275607549 | 0.022869509 | 0.293754263 |
| WNT8B | 5.378172712 | -0.533777839 | 0.238284632 | -2.240085038 | 0.025085403 | 0.303909072 |
| GZMB | 2568.413444 | 0.533470099 | 0.178805453 | 2.983522541 | 0.00284951 | 0.111099203 |
| RAB13 | 93.1472857 | 0.533232018 | 0.178141579 | 2.993304651 | 0.002759741 | 0.108662409 |
| RNF222 | 7.417067593 | -0.533140671 | 0.25895765 | -2.05879483 | 0.039513897 | 0.371922854 |
| HSD3B7 | 106.9871648 | 0.532992349 | 0.173305353 | 3.075452308 | 0.002101835 | 0.093730735 |
| B3GAT1 | 559.7085019 | 0.532886904 | 0.217253087 | 2.452839271 | 0.014173365 | 0.239811366 |
| ATP6V0E2-AS1 | 91.08386873 | -0.532368376 | 0.128773325 | -4.134151043 | 3.56E-05 | 0.01061992 |
| RTN4RL1 | 13.87714615 | -0.531594602 | 0.252608719 | -2.104419054 | 0.035341909 | 0.35707827 |
| RAMP1 | 15.14907014 | 0.531550134 | 0.256077417 | 2.075739981 | 0.037918006 | 0.366588995 |
| PEAR1 | 131.8341497 | 0.531508611 | 0.253326353 | 2.098118117 | 0.035894713 | 0.35953078 |
| PCSK6 | 111.916109 | 0.531069672 | 0.262014763 | 2.026869276 | 0.042675783 | 0.385176301 |
| ST7-AS1 | 5.84738317 | 0.530586736 | 0.244352366 | 2.171400031 | 0.029900947 | 0.330339194 |
| CDC25C | 11.87424334 | 0.529556411 | 0.268083686 | 1.975339935 | 0.048229584 | 0.404801657 |
| TMEM204 | 874.2889724 | -0.52921523 | 0.141638436 | -3.73638149 | 0.000186687 | 0.027878648 |
| EGFL7 | 45.11896448 | 0.528481268 | 0.199585368 | 2.647895854 | 0.008099447 | 0.182673117 |
| LOC105376483 | 6.731168466 | 0.528212896 | 0.244484195 | 2.160519602 | 0.030732468 | 0.334141426 |
| SHCBP1 | 77.59176125 | 0.527497795 | 0.2005976 | 2.629631631 | 0.008547743 | 0.187091308 |
| TNIP3 | 37.18846248 | 0.52737749 | 0.164115254 | 3.213458085 | 0.001311469 | 0.074627896 |
| CLEC4D | 733.4347859 | 0.52717216 | 0.242980011 | 2.169611232 | 0.03003631 | 0.330753743 |
| CGN | 12.32507906 | -0.527008118 | 0.189892833 | -2.775292309 | 0.005515211 | 0.15530835 |
| COL6A4P2 | 18.23944096 | -0.527002474 | 0.205258582 | -2.567505183 | 0.010243326 | 0.202118557 |
| CDKN2B-AS1 | 14.30329192 | 0.526529538 | 0.153106409 | 3.438977784 | 0.000583915 | 0.048260041 |
| LOC107986075 | 9.373262175 | -0.524999075 | 0.211685582 | -2.480088964 | 0.013134961 | 0.229885608 |
| GAL3ST4 | 243.0224015 | -0.521934671 | 0.140341343 | -3.71903716 | 0.000199984 | 0.028985863 |
| PRRG1 | 10.91512081 | 0.520856279 | 0.243986071 | 2.134778743 | 0.032779087 | 0.345471275 |
| LINC02298 | 7.514413009 | -0.520854241 | 0.240465543 | -2.166024433 | 0.03030932 | 0.332121838 |
| LINC02725 | 9.155283829 | 0.520746496 | 0.249285702 | 2.088954523 | 0.036711817 | 0.362160521 |
| LOC124903659 | 115.6179378 | 0.520547846 | 0.256127011 | 2.032381681 | 0.042115031 | 0.382391292 |
| TMEM220-AS1 | 5.662545669 | -0.520163286 | 0.209808414 | -2.479229866 | 0.013166642 | 0.229885608 |
| CNN3 | 48.30813301 | -0.518454325 | 0.174720903 | -2.967328563 | 0.003003998 | 0.115203891 |
| ERVH48-1 | 13.35860299 | -0.518027862 | 0.200904707 | -2.578475483 | 0.009923734 | 0.199008915 |
| KIF2C | 56.60809086 | 0.51679272 | 0.20904204 | 2.472195166 | 0.013428618 | 0.233041977 |
| PLEKHH2 | 26.61519572 | -0.516277614 | 0.23089636 | -2.235971214 | 0.025353657 | 0.304738588 |
| TRAV1-2 | 141.9941062 | -0.515401289 | 0.213583956 | -2.41310864 | 0.015817104 | 0.250632442 |
| MIR12136 | 17.32177271 | 0.51539962 | 0.234639958 | 2.196555201 | 0.028052229 | 0.321491588 |
| CCDC194 | 36.60517157 | 0.515339043 | 0.218697345 | 2.356402835 | 0.018452894 | 0.266088849 |
| CHST13 | 203.2413004 | 0.514869954 | 0.211924374 | 2.429498528 | 0.015119726 | 0.245639432 |
| LEF1 | 3294.117673 | -0.514534975 | 0.131649494 | -3.908370326 | 9.29E-05 | 0.018690355 |
| LINC02561 | 11.794951 | 0.514377187 | 0.226894569 | 2.26703173 | 0.023388287 | 0.294964762 |
| EMID1 | 22.20558558 | 0.514034114 | 0.245329085 | 2.09528403 | 0.036145751 | 0.360091972 |
| LOC107985549 | 7.09453385 | -0.513970477 | 0.192425578 | -2.671009142 | 0.007562359 | 0.179385337 |
| LTBP1 | 115.9111256 | 0.513788866 | 0.243886951 | 2.106668121 | 0.035146357 | 0.356038226 |
| ARHGEF17 | 33.04040398 | 0.513324057 | 0.219678391 | 2.336707107 | 0.019454419 | 0.272201772 |
| MTUS1 | 115.8447002 | -0.51331196 | 0.208071765 | -2.466994792 | 0.013625234 | 0.234101472 |
| DNASE1L2 | 55.23648801 | 0.513091233 | 0.147253493 | 3.484407889 | 0.000493227 | 0.044394952 |
| LOC105369747 | 48.61543347 | -0.512759104 | 0.143765451 | -3.566636487 | 0.000361593 | 0.037637338 |
| PRRT4 | 20.64245743 | 0.512690809 | 0.224422645 | 2.284487861 | 0.022342875 | 0.290955516 |
| SNED1-AS1 | 8.779388098 | -0.512441634 | 0.255242214 | -2.007668037 | 0.044678579 | 0.392755955 |
| FAM72B | 13.27877625 | 0.51156803 | 0.197194548 | 2.59423009 | 0.009480302 | 0.194275683 |
| AMIGO1 | 605.4206388 | -0.510149067 | 0.11639143 | -4.383046651 | 1.17E-05 | 0.007209112 |
| SELP | 259.763179 | 0.509484603 | 0.197592643 | 2.578459383 | 0.009924197 | 0.199008915 |
| MMRN1 | 114.3945176 | 0.509376571 | 0.238114191 | 2.139211312 | 0.032418558 | 0.343567003 |
| TMEM254-AS1 | 36.41740376 | -0.508981292 | 0.21096441 | -2.412640557 | 0.01583743 | 0.250752941 |
| RBM26-AS1 | 25.26442506 | -0.50758748 | 0.215296662 | -2.357618905 | 0.018392564 | 0.265795799 |
| GZMH | 3682.997022 | 0.507197911 | 0.178738652 | 2.837650985 | 0.004544685 | 0.1425293 |
| LOC101928214 | 10.0964049 | 0.50718883 | 0.245601479 | 2.065088664 | 0.038914612 | 0.370036096 |
| CDC6 | 49.24354129 | 0.507121765 | 0.226391189 | 2.240024303 | 0.025089345 | 0.303909072 |
| LOC107986759 | 8.479961886 | -0.50660482 | 0.224628976 | -2.255295956 | 0.024114758 | 0.298336333 |
| NEURL1 | 222.3840799 | 0.506570472 | 0.16775859 | 3.019639542 | 0.002530757 | 0.105936619 |
| TLR2 | 16079.39535 | 0.50617779 | 0.236832462 | 2.137282129 | 0.032575051 | 0.344669573 |
| IGLV1-51 | 4.650901802 | -0.505933799 | 0.222738932 | -2.271420604 | 0.023121528 | 0.29456331 |
| AMN | 27.94910917 | -0.505645123 | 0.150618175 | -3.357132192 | 0.000787554 | 0.057711056 |
| FKBP1B | 83.07702214 | 0.505611529 | 0.189726935 | 2.664943319 | 0.00770013 | 0.179627174 |
| LOC105374334 | 19.82650813 | -0.505423436 | 0.15437774 | -3.273939863 | 0.001060592 | 0.067877875 |
| CPT1A | 2172.230452 | 0.504832403 | 0.120982206 | 4.172782258 | 3.01E-05 | 0.009993553 |
| LOC105370792 | 49.09529485 | -0.503898625 | 0.212537803 | -2.370865882 | 0.017746469 | 0.262847174 |
| GADD45G | 54.76981801 | 0.503487271 | 0.179800579 | 2.800253897 | 0.005106243 | 0.148523663 |
| RNR2 | 130528.3942 | 0.503263268 | 0.171133741 | 2.940760052 | 0.00327408 | 0.119960361 |
| ENKUR | 122.1246707 | 0.503010448 | 0.206348796 | 2.437670861 | 0.014782227 | 0.243671243 |
| BUB1 | 103.459561 | 0.502987168 | 0.240414446 | 2.092166987 | 0.036423581 | 0.361480514 |
| SMANTIS | 212.9567646 | 0.50284052 | 0.231853284 | 2.168787565 | 0.030098816 | 0.330753743 |
| FKBP14-AS1 | 6.901606257 | 0.502671806 | 0.232375536 | 2.163187283 | 0.030526778 | 0.333285648 |
| EML5 | 26.01057958 | -0.501328168 | 0.136213752 | -3.680451937 | 0.000232821 | 0.031633606 |
| MPP2 | 34.01252601 | -0.500991914 | 0.173833056 | -2.882029024 | 0.003951233 | 0.132025149 |
| KLHL34 | 18.59130175 | -0.500425084 | 0.189994965 | -2.633886032 | 0.008441382 | 0.186307922 |
| LOC105375337 | 7.289143508 | -0.50031056 | 0.224223234 | -2.231305611 | 0.025660892 | 0.307493926 |
| KIF11 | 144.9297944 | 0.500249258 | 0.18497905 | 2.704356294 | 0.006843686 | 0.170547089 |

| **Supplementary Table 2: DEGs from edgeR** | | | | | |
| --- | --- | --- | --- | --- | --- |
| symbol | logFC | logCPM | F | PValue | FDR |
| IFI27 | 3.22003303 | 1.407933988 | 13.14257758 | 0.000626936 | 0.067632945 |
| CEACAM6 | 2.652003181 | 2.795535926 | 16.08846745 | 0.000182093 | 0.039707854 |
| MMP8 | 2.598230975 | 4.364007902 | 14.71559323 | 0.000321387 | 0.053072557 |
| CTSG | 2.582817268 | 1.406870163 | 19.44220949 | 4.79E-05 | 0.030198328 |
| CEACAM8 | 2.541705574 | 3.631185925 | 15.37805782 | 0.000243915 | 0.045394522 |
| LINC02073 | 2.411773489 | -0.778807906 | 19.16639252 | 5.27E-05 | 0.031126214 |
| LTF | 2.406899577 | 6.547384029 | 14.06723473 | 0.000422314 | 0.057339756 |
| LCN2 | 2.341612257 | 4.646659055 | 15.21896554 | 0.000260543 | 0.046784476 |
| LOC105375878 | 2.32806398 | -0.687199091 | 16.15388287 | 0.000175822 | 0.038928222 |
| CWC25 | 2.262833138 | 1.158307483 | 26.55899703 | 3.45E-06 | 0.005865645 |
| ALOX15B | 2.196071432 | 1.260430058 | 12.40352975 | 0.000864018 | 0.078479712 |
| CCL23 | 2.15060242 | -1.103539699 | 14.34133528 | 0.000372842 | 0.054202373 |
| ERFE | -2.0868159 | -0.350668167 | 19.49267904 | 4.65E-05 | 0.030198328 |
| NDUFB10 | 2.081510913 | 0.521235192 | 12.91319357 | 0.000690702 | 0.070348192 |
| LINC02009 | 2.076478047 | 1.183930404 | 11.91114101 | 0.00107261 | 0.087386124 |
| NDUFA11 | 2.045578365 | 0.457198108 | 13.71865214 | 0.000488158 | 0.061504299 |
| CRISP3 | 1.999804246 | 3.228836822 | 13.12306218 | 0.000632232 | 0.067632945 |
| DEFA3 | 1.93082557 | 3.425801337 | 11.43744043 | 0.001323326 | 0.096748942 |
| OLR1 | 1.93027424 | 1.51500544 | 12.57709279 | 0.000800998 | 0.075673438 |
| BPI | 1.904455122 | 4.456161442 | 12.75552397 | 0.000741194 | 0.073296347 |
| ABCA13 | 1.851357212 | 1.77789954 | 11.47912295 | 0.001298987 | 0.096482397 |
| RPS3A | 1.846651424 | 3.171754292 | 9.016452318 | 0.004003385 | 0.157471864 |
| OTOF | 1.78919994 | 2.679061536 | 8.439331548 | 0.005260057 | 0.17829736 |
| AZU1 | 1.784633294 | -1.240371219 | 13.01747025 | 0.00065493 | 0.068763569 |
| MPO | 1.784024226 | 3.488176594 | 15.40057816 | 0.000241652 | 0.045394522 |
| ARG1 | 1.776898202 | 4.439876132 | 8.28727557 | 0.005655993 | 0.182116995 |
| DAAM2 | 1.776048424 | 2.832530508 | 6.907466441 | 0.011074287 | 0.233992166 |
| LOC124900992 | 1.756515575 | 1.140625046 | 4.648482672 | 0.035421528 | 0.370988163 |
| OLFM4 | 1.733535387 | 2.879609373 | 10.87160858 | 0.001705278 | 0.106245694 |
| HBZ | 1.655109827 | -0.465904995 | 7.57649927 | 0.007959947 | 0.207375909 |
| TMIGD3 | 1.647126067 | 1.175953608 | 12.41414703 | 0.000857631 | 0.078479712 |
| KLHDC8A | 1.645706289 | 0.008523345 | 12.16739772 | 0.000953152 | 0.08171281 |
| NECAP2 | 1.637062587 | 1.92128779 | 14.26057448 | 0.000388271 | 0.055529496 |
| ACAP1 | 1.597299736 | 3.906123425 | 12.01080456 | 0.001026498 | 0.08481683 |
| RNASE1 | 1.594941238 | -0.052249375 | 9.75034207 | 0.002834745 | 0.132482766 |
| TFF3 | 1.561919555 | -0.567004756 | 12.39201299 | 0.000861598 | 0.078479712 |
| CHIT1 | 1.558879983 | 1.392494097 | 10.32247015 | 0.002184472 | 0.115816285 |
| TRPC6 | 1.557251521 | 0.555208803 | 17.38687785 | 0.000106351 | 0.035090707 |
| SERPINB10 | 1.548635432 | 1.459122254 | 13.75701827 | 0.000480233 | 0.061251662 |
| TRAPPC4 | -1.514245076 | 0.478276041 | 6.556377102 | 0.013193912 | 0.246339455 |
| LOC105378061 | 1.495594625 | 0.194931101 | 12.2259575 | 0.000928337 | 0.08171281 |
| COX5B | 1.488889241 | 0.404101108 | 10.30368907 | 0.002198146 | 0.115816285 |
| NDUFB9 | -1.449817044 | 1.556954052 | 5.298065654 | 0.025117695 | 0.325264313 |
| ASIC1 | -1.424569524 | 0.177343977 | 30.83255605 | 7.74E-07 | 0.002631553 |
| HLA-DRB3 | -1.423430041 | 3.029788817 | 6.225116308 | 0.015591133 | 0.265264738 |
| RNASE3 | 1.418220773 | 1.990146265 | 12.64933747 | 0.000774408 | 0.075673438 |
| LOC107985357 | 1.400755738 | 0.415740484 | 10.31678354 | 0.002184628 | 0.115816285 |
| NRCAM | -1.383718409 | 1.373645783 | 26.7164506 | 3.22E-06 | 0.005865645 |
| CTSE | 1.383123924 | 0.311030356 | 13.87317402 | 0.000454276 | 0.059897549 |
| IL1R2 | 1.377655028 | 7.0255005 | 9.544545539 | 0.003128595 | 0.140037565 |
| GTF2H2C_2 | -1.374601281 | -0.061425119 | 11.40729361 | 0.001334561 | 0.0970066 |
| RMRP | -1.372545498 | 1.33343695 | 5.925311253 | 0.018161506 | 0.282624938 |
| PRRT4 | 1.367651384 | 0.290513761 | 10.01544974 | 0.002507444 | 0.123713769 |
| COL17A1 | 1.365498701 | 0.354984671 | 6.868047407 | 0.011281266 | 0.235461555 |
| ROBO1 | -1.363676346 | -0.251819393 | 22.81180605 | 1.30E-05 | 0.012996487 |
| IGHV3-49 | 1.355181846 | -0.951093481 | 7.78843358 | 0.007164809 | 0.200108758 |
| CD248 | -1.348812296 | 1.851819246 | 27.44047735 | 2.51E-06 | 0.005331477 |
| LOC112268252 | 1.345786324 | 0.462034883 | 8.043746978 | 0.006344564 | 0.188991778 |
| RNF187 | 1.335994076 | 1.584308512 | 10.31371952 | 0.002191655 | 0.115816285 |
| FBL | 1.334394612 | 2.029160833 | 4.649786963 | 0.035396774 | 0.370957322 |
| LOC102723407 | -1.328422408 | 0.606139716 | 6.114661254 | 0.016489989 | 0.27099346 |
| FN1 | 1.328403407 | 0.102458008 | 10.08600349 | 0.00242595 | 0.12076922 |
| LOC112268317 | 1.31111189 | -0.351254006 | 5.304087903 | 0.02502056 | 0.324370959 |
| ERG | 1.299884769 | 0.141243572 | 8.992392889 | 0.004035173 | 0.157471864 |
| LOC105373165 | 1.29124562 | 0.100337333 | 7.123368087 | 0.009932456 | 0.225857151 |
| SLC1A3 | 1.276835091 | 0.971931277 | 7.995597525 | 0.006496666 | 0.190091645 |
| MIR34AHG | 1.275881195 | -1.420587254 | 18.89203493 | 5.79E-05 | 0.032822444 |
| LOC105371498 | 1.270890201 | 0.167777358 | 12.54772955 | 0.000804501 | 0.075673438 |
| SCARNA2 | -1.244025611 | 0.514092147 | 7.616719522 | 0.007806 | 0.207074613 |
| PXDN | -1.2406784 | -1.030081159 | 12.24559659 | 0.000915697 | 0.08171281 |
| REG4 | -1.236362457 | -0.314265916 | 27.69919592 | 2.25E-06 | 0.005331477 |
| SFRP5 | -1.231632128 | 0.376150927 | 15.00786943 | 0.000281336 | 0.049020357 |
| DEPDC1 | 1.229099985 | -0.844801153 | 11.19269626 | 0.00146143 | 0.099288115 |
| IL34 | 1.209160252 | -0.325448751 | 10.75570667 | 0.001782068 | 0.108287189 |
| ECHDC3 | 1.201513543 | 2.633873221 | 8.467220196 | 0.00519066 | 0.177285016 |
| PMP22 | 1.187138014 | 1.092211623 | 14.19069781 | 0.000396923 | 0.055795536 |
| SHANK1 | -1.177404588 | -0.817706722 | 13.89339342 | 0.000447829 | 0.059508792 |
| E2F8 | 1.169431286 | -0.193991491 | 11.02819947 | 0.001574931 | 0.102636025 |
| LYPD2 | 1.163263512 | 1.147369587 | 11.49665 | 0.001281531 | 0.095946912 |
| SLC25A6 | 1.158511801 | 2.32153486 | 9.575256676 | 0.003079816 | 0.138217927 |
| IL18R1 | 1.158302374 | 5.688988268 | 9.645675645 | 0.002985327 | 0.135768531 |
| RNF212 | -1.154668275 | 0.14027696 | 31.76444605 | 5.68E-07 | 0.002631553 |
| OLIG2 | 1.153574445 | 2.736436457 | 10.70276323 | 0.001837763 | 0.108690202 |
| MEG3 | 1.139882071 | -1.29733476 | 10.3298515 | 0.002159178 | 0.115816285 |
| LRRN3 | -1.136791438 | 4.243272604 | 23.58147213 | 1.01E-05 | 0.011946479 |
| TPST1 | 1.135885101 | 3.830999008 | 7.840378809 | 0.007012112 | 0.198121287 |
| LOC102724008 | 1.134801178 | -0.119173373 | 11.08029221 | 0.001538279 | 0.101021567 |
| MICB-DT | 1.133129553 | -1.610077329 | 5.259971638 | 0.025554882 | 0.327306469 |
| ADORA3 | 1.129773292 | 2.639457339 | 19.67461891 | 4.33E-05 | 0.029430758 |
| SAMD14 | 1.121358817 | 0.681832743 | 10.40803377 | 0.002091623 | 0.114393594 |
| SUCNR1 | 1.117907647 | 0.702923723 | 12.3277903 | 0.000885396 | 0.079680945 |
| PLAAT5 | 1.11737179 | 0.666864343 | 13.79468994 | 0.000468017 | 0.060767247 |
| GSTO2 | -1.115752958 | 1.186957819 | 15.39943154 | 0.000239092 | 0.045394522 |
| RN7SK | -1.111817464 | 4.875775559 | 4.82492501 | 0.032236045 | 0.358836968 |
| LOC124900812 | 1.11157053 | 3.522975496 | 11.34409738 | 0.001379578 | 0.097312715 |
| HBG2 | 1.110175446 | 7.846449663 | 4.493797331 | 0.038493917 | 0.386607251 |
| TRBV28 | 1.09972928 | -0.014909134 | 11.26810428 | 0.001413667 | 0.097744133 |
| TCN1 | 1.094644754 | 3.509939272 | 8.205865973 | 0.005880755 | 0.184576822 |
| HNRNPL | -1.093303358 | 3.189005968 | 7.782457183 | 0.007211501 | 0.200559749 |
| ALOX15 | 1.086907692 | 5.552512765 | 8.626462608 | 0.004812428 | 0.170175863 |
| TNNT1 | 1.086554481 | 1.926672744 | 10.76459224 | 0.00178261 | 0.108287189 |
| LOC105369595 | 1.078735731 | -0.484023881 | 8.071498001 | 0.006240547 | 0.18876876 |
| RNF135 | 1.076618633 | 0.509419557 | 7.070568563 | 0.010191946 | 0.226773268 |
| FBLN2 | -1.069015957 | 1.628069923 | 40.15220951 | 4.07E-08 | 0.000691706 |
| SLC43A2 | 1.068446957 | 3.898427969 | 9.52804749 | 0.003152643 | 0.140140902 |
| PRSS33 | 1.058905647 | 4.16952303 | 7.181006438 | 0.009673685 | 0.222532151 |
| BEX1 | 1.053444047 | -1.373838733 | 5.698024585 | 0.020345262 | 0.295014967 |
| LINC00664 | 1.050960948 | -0.428953079 | 4.44210668 | 0.039544939 | 0.389504812 |
| RGL2 | 1.02961791 | 0.89622174 | 7.561029929 | 0.008010228 | 0.207375909 |
| RAP1GAP | -1.019400417 | 1.909966286 | 4.113025027 | 0.047354226 | 0.415393519 |
| LOC105370655 | -1.018530957 | 1.045348393 | 11.76510122 | 0.001136063 | 0.089459685 |
| FAM156B | 1.014325391 | -1.176807779 | 6.815209213 | 0.011541379 | 0.237364724 |
| ITGA2B | 1.007856153 | 5.291444946 | 7.755597914 | 0.007305981 | 0.201021829 |
| UQCRC2 | -1.005615021 | 2.204485083 | 4.043122733 | 0.049209078 | 0.420018181 |
| LOC112268261 | -0.98770334 | 1.75096656 | 17.18397754 | 0.000115079 | 0.035588598 |
| CYP1B1-AS1 | 0.986984339 | 1.195932391 | 5.856229581 | 0.018796158 | 0.285380723 |
| CLU | 0.985459134 | 5.706109608 | 11.75459209 | 0.001149461 | 0.090097581 |
| DTL | 0.984349757 | 0.804849876 | 8.920843822 | 0.004168356 | 0.15932487 |
| DYNC1I2 | 0.984257575 | 1.836301944 | 8.508257223 | 0.005076644 | 0.174441684 |
| CEP55 | 0.980676574 | 0.783203585 | 11.28744579 | 0.001401706 | 0.097312715 |
| IFNG-AS1 | -0.980505722 | 1.951669935 | 13.25849031 | 0.00059216 | 0.067123506 |
| CACHD1 | -0.97887836 | 0.770638778 | 18.65295555 | 6.36E-05 | 0.033945237 |
| RIPK3 | 0.97823938 | 0.082081932 | 4.177513907 | 0.045672455 | 0.411027928 |
| VWCE | 0.976898391 | 2.526717853 | 8.284772737 | 0.005655043 | 0.182116995 |
| MS4A3 | 0.976447 | 1.193689517 | 4.846910971 | 0.031842149 | 0.356317841 |
| FKBP5 | 0.975673875 | 7.920735672 | 7.190592637 | 0.009628135 | 0.222358824 |
| HSF1 | 0.973880293 | 1.749470595 | 5.041509672 | 0.028728596 | 0.341366779 |
| LOC101929698 | -0.972160144 | 0.873762977 | 32.67797826 | 4.21E-07 | 0.002631553 |
| LINC02207 | 0.969384577 | 1.53598817 | 5.584785717 | 0.021622181 | 0.303692555 |
| IL5RA | 0.969017249 | 4.745784549 | 11.36023238 | 0.001369678 | 0.097312715 |
| FSTL1 | 0.966311773 | 1.688355143 | 7.291965441 | 0.009143279 | 0.217507739 |
| FBN1 | 0.965317163 | 0.267259946 | 12.08290082 | 0.000982394 | 0.082313025 |
| KIF26A | 0.959626248 | -1.166916544 | 7.169685199 | 0.009676455 | 0.222532151 |
| PRSS41 | 0.959564346 | 0.348180964 | 7.578601466 | 0.007933678 | 0.207375909 |
| CAMP | 0.956525165 | 4.310913053 | 6.798230257 | 0.01169219 | 0.238742441 |
| MYCT1 | 0.952014392 | 0.84400183 | 17.26400183 | 0.000110653 | 0.035090707 |
| CEBPE | 0.948609786 | 3.238465106 | 13.49782424 | 0.000535734 | 0.066031103 |
| COL9A2 | 0.948425849 | 4.534066928 | 9.183391151 | 0.003701996 | 0.151728313 |
| LOC107986639 | 0.946611452 | -0.895184786 | 9.367691739 | 0.003368999 | 0.144699489 |
| MYL9 | 0.94004082 | 4.248298181 | 6.151089615 | 0.016187607 | 0.269252407 |
| IDO1 | 0.939896781 | 3.817421528 | 10.47036028 | 0.002043862 | 0.11398378 |
| MCM10 | 0.938825045 | -0.067601573 | 7.511601971 | 0.008188002 | 0.209476784 |
| LOC112268474 | 0.936036526 | -0.347941809 | 7.024678167 | 0.010404087 | 0.229371864 |
| GATA1 | 0.935999866 | 3.864653023 | 18.52849462 | 6.78E-05 | 0.033945237 |
| NOG | -0.933153103 | 3.108010656 | 16.97650403 | 0.000125884 | 0.035686036 |
| SIGLEC8 | 0.931622131 | 4.056797324 | 5.236730326 | 0.025936936 | 0.329470761 |
| TUBB2A | 0.928309251 | 2.728974889 | 4.7995609 | 0.032674353 | 0.36011026 |
| LOC101927369 | 0.927707478 | -0.986987612 | 9.130531626 | 0.003765035 | 0.152527385 |
| SLC28A3 | 0.927444579 | -0.122554915 | 8.665683075 | 0.004693649 | 0.167719061 |
| BIRC5 | 0.927440621 | 1.091933759 | 9.014555919 | 0.003988301 | 0.157394443 |
| TICRR | 0.926366109 | -0.630059567 | 6.561320409 | 0.013109092 | 0.245487186 |
| LOC107984706 | -0.92595797 | -0.787302552 | 7.666457978 | 0.007589885 | 0.204162115 |
| CD24 | 0.921808929 | 5.621832561 | 15.65400929 | 0.000217069 | 0.0437774 |
| SLC29A1 | 0.921798358 | 4.871320285 | 11.38510855 | 0.001354561 | 0.097312715 |
| RNASE2 | 0.920306688 | 4.998046687 | 8.703072786 | 0.004640897 | 0.166953016 |
| DLGAP5 | 0.917980546 | 1.00904565 | 7.192728325 | 0.009592488 | 0.222173786 |
| PPBP | 0.916634825 | 6.621589378 | 8.745998152 | 0.004547608 | 0.165987685 |
| NHIP | -0.915571841 | 0.388853844 | 9.667829646 | 0.002934416 | 0.134260182 |
| HMOX2 | -0.914044711 | 1.247539102 | 4.023149214 | 0.049739307 | 0.420320697 |
| LEF1-AS1 | -0.913044061 | 0.755556278 | 31.38118959 | 6.45E-07 | 0.002631553 |
| IL1RL1 | 0.911274734 | 0.96001165 | 7.839628729 | 0.006989223 | 0.197803143 |
| RCAN2 | 0.907744024 | 0.659247779 | 13.15760992 | 0.000614062 | 0.067123506 |
| CLC | 0.906157528 | 7.320219031 | 8.145682067 | 0.00605295 | 0.187190232 |
| TNNI2 | -0.904495067 | -0.133016513 | 5.71160815 | 0.020227659 | 0.294061754 |
| ECRG4 | -0.89331391 | -0.801900454 | 16.16641667 | 0.000172915 | 0.038928222 |
| LOC124902302 | 0.89326295 | 0.011672877 | 10.08734381 | 0.002413253 | 0.12072654 |
| IGHV3-74 | -0.891850667 | -0.2810845 | 4.148092963 | 0.046409987 | 0.412521606 |
| CARMIL3 | 0.890775991 | -1.484404042 | 6.142215204 | 0.016194086 | 0.269252407 |
| CMTM5 | 0.887036928 | 2.353996209 | 8.426993995 | 0.005277425 | 0.17829736 |
| STOX1 | -0.880057001 | -0.679938048 | 5.265467297 | 0.025497765 | 0.32709446 |
| CXCL5 | 0.877441883 | 2.526068467 | 6.273816724 | 0.015200324 | 0.2633889 |
| TREML1 | 0.876380485 | 4.015354705 | 8.629119636 | 0.004806369 | 0.170175863 |
| ANLN | 0.876010461 | 0.314454541 | 7.18113955 | 0.009634808 | 0.222358824 |
| PIP4P1 | -0.87352828 | 2.195480318 | 4.031047353 | 0.049537417 | 0.420018181 |
| CA6 | -0.867386686 | 1.59814824 | 14.56636168 | 0.000337277 | 0.053703003 |
| HLA-DRA | -0.862633659 | 3.226170284 | 4.334445416 | 0.041962108 | 0.39828878 |
| LONRF2 | 0.86230739 | -1.150666323 | 6.094316322 | 0.016594061 | 0.272076428 |
| CDK15 | 0.86061338 | -0.849843266 | 5.810920211 | 0.019191886 | 0.288073651 |
| RYR2 | 0.858957124 | -1.051524372 | 8.383192019 | 0.005365921 | 0.178958732 |
| KIF20A | 0.856348194 | -0.320180591 | 8.033766411 | 0.006346839 | 0.188991778 |
| THBS1 | 0.855995155 | 3.785922209 | 9.651841637 | 0.00297323 | 0.135580861 |
| LOC107987207 | -0.855813823 | -0.097855826 | 7.472472848 | 0.008345209 | 0.211025938 |
| FILIP1L | 0.853944444 | 0.143699203 | 7.652185864 | 0.007642963 | 0.204723071 |
| FOXJ1 | -0.847030495 | -1.271372625 | 13.15171237 | 0.000615631 | 0.067123506 |
| TOP2A | 0.84299039 | 2.537576889 | 8.718652833 | 0.004593951 | 0.166606651 |
| CLEC4E | 0.841961285 | 7.358836119 | 6.731102675 | 0.012089925 | 0.241361502 |
| KIF18B | 0.841653722 | -0.129454885 | 7.499252827 | 0.008233189 | 0.209952491 |
| TTC24 | -0.839104941 | -0.539056185 | 6.627983378 | 0.012677792 | 0.243208759 |
| LGALS12 | 0.838814867 | 3.820883449 | 17.00237724 | 0.000124515 | 0.035686036 |
| ASPM | 0.837087973 | 1.250554076 | 7.684260634 | 0.007536221 | 0.203466008 |
| LOC124900837 | -0.836995456 | -1.211997705 | 11.23125136 | 0.001435014 | 0.098439201 |
| CACNG6 | 0.835039078 | 2.053082784 | 5.962969494 | 0.017798928 | 0.279024846 |
| TTK | 0.833774379 | 0.120541277 | 9.930408277 | 0.002594206 | 0.125194563 |
| SURF4 | 0.833563107 | 0.734531506 | 7.333718595 | 0.008939698 | 0.215681318 |
| RRM2 | 0.832481856 | 3.221316987 | 7.233062532 | 0.009421961 | 0.220025287 |
| LOC107985926 | 0.830875575 | -0.016892265 | 4.517207602 | 0.037956079 | 0.383369926 |
| CDT1 | 0.829453766 | 0.952226883 | 11.14329966 | 0.001493466 | 0.100404597 |
| HBA1 | 0.824840124 | 9.504692769 | 6.449451165 | 0.013908572 | 0.252748888 |
| PCLAF | 0.823813747 | 1.146062901 | 7.731741987 | 0.007362939 | 0.20121458 |
| PTGDR2 | 0.818173043 | 4.715139236 | 9.239363972 | 0.003606357 | 0.14961104 |
| LOC101929322 | 0.81720077 | 0.127380049 | 8.69437367 | 0.004626348 | 0.166953016 |
| ACKR1 | 0.812557537 | -0.812973642 | 4.813636094 | 0.032352587 | 0.359283204 |
| CKAP2L | 0.811803094 | -0.332751224 | 6.761800253 | 0.011851086 | 0.239685054 |
| DCLK2 | 0.811438713 | -0.546241267 | 5.232438442 | 0.025924305 | 0.329470761 |
| VMO1 | 0.81136734 | -0.680654544 | 6.4957222 | 0.013540668 | 0.247753847 |
| BCAT1 | 0.811339113 | 3.013650365 | 15.66316945 | 0.000214359 | 0.0437774 |
| LOC124902872 | 0.806508977 | 3.156734083 | 4.698171292 | 0.034491525 | 0.369670033 |
| KHDRBS3 | 0.805196979 | -1.261728099 | 5.868833876 | 0.018623156 | 0.28533693 |
| GRB10 | 0.804888809 | 3.576254887 | 8.617301479 | 0.004827416 | 0.170351707 |
| ADGRE4P | 0.804277773 | 5.27638133 | 8.372832482 | 0.005429499 | 0.180107282 |
| SEPTIN4 | 0.803067242 | 1.395408228 | 6.945449651 | 0.010837641 | 0.233014111 |
| LOC105369382 | 0.80262305 | -0.018239719 | 8.15754527 | 0.005979378 | 0.185836125 |
| LOC107985900 | -0.80259282 | 0.509889399 | 15.27608294 | 0.000249866 | 0.045698696 |
| PPP1R3G | 0.802236848 | -0.843423561 | 7.944714068 | 0.006625866 | 0.191015842 |
| GINS2 | 0.801639672 | 0.713349473 | 10.68032581 | 0.001840366 | 0.108690202 |
| VWDE | 0.799342418 | -1.014784428 | 5.272274603 | 0.02537734 | 0.326754868 |
| CYB5R2 | 0.797745686 | -0.490490566 | 7.232646736 | 0.009380507 | 0.219770024 |
| PROS1 | 0.797204779 | 1.649182068 | 8.633699563 | 0.004774708 | 0.169546981 |
| OR2W3 | 0.797009272 | 3.488904577 | 4.410066778 | 0.040275932 | 0.393256787 |
| HP | 0.794070446 | 3.089003712 | 7.254346512 | 0.009320146 | 0.218959058 |
| RHCE | 0.793806139 | 0.071585976 | 6.134128414 | 0.016270752 | 0.270262915 |
| OR10G2 | -0.792358941 | -0.968666347 | 7.360310696 | 0.008809371 | 0.214055127 |
| GP9 | 0.791497904 | 2.458786239 | 7.091749562 | 0.010089323 | 0.226195569 |
| SERPINE2 | -0.791191749 | 1.097496437 | 16.36403496 | 0.000159466 | 0.038928222 |
| IGKV1-33 | 0.790407944 | -0.383371981 | 5.091940309 | 0.02791442 | 0.33980362 |
| EGF | 0.790166002 | 1.641334144 | 9.219329955 | 0.003620569 | 0.149835166 |
| ERBB3 | -0.78892367 | -0.743179351 | 12.81817342 | 0.000711518 | 0.071816786 |
| LIPH | 0.787954811 | -0.835748263 | 8.419816535 | 0.005272767 | 0.17829736 |
| NECTIN2 | 0.787877534 | 2.353939791 | 10.49394325 | 0.002011336 | 0.11398378 |
| PPIP5K1P1-CATSPER2 | -0.787135682 | -0.323629422 | 4.171897212 | 0.045794124 | 0.411046846 |
| IER5L-AS1 | -0.786269397 | -1.013444396 | 10.44932625 | 0.002044456 | 0.11398378 |
| NEAT1 | 0.786159704 | 4.750400296 | 9.298521556 | 0.003508091 | 0.147828457 |
| SLC16A14 | 0.785713618 | 0.421999117 | 11.09004877 | 0.001529088 | 0.101021567 |
| LOC124902787 | 0.784618815 | -0.352240728 | 6.3835872 | 0.014330476 | 0.256036839 |
| LINC02295 | -0.784082578 | 1.865834008 | 17.62704555 | 9.56E-05 | 0.035090707 |
| SCARA5 | -0.783878398 | -1.09439445 | 8.110990759 | 0.006114885 | 0.188079714 |
| LOC105376568 | -0.783832273 | 1.959870772 | 5.843646867 | 0.018917326 | 0.285759146 |
| ITGB3 | 0.782849889 | 5.510308173 | 5.880553621 | 0.018582381 | 0.285221288 |
| LINC02086 | 0.78268449 | -6.70E-05 | 8.793982233 | 0.004412901 | 0.163668988 |
| ZBTB20-AS1 | 0.782532326 | -0.769041217 | 13.29258002 | 0.000579277 | 0.067123506 |
| HMMR | 0.778317187 | 0.261924574 | 6.323507703 | 0.014779898 | 0.258993199 |
| HTRA1 | 0.777811137 | 0.103025534 | 4.536777251 | 0.037555141 | 0.381508564 |
| S100B | -0.777659082 | 2.202858956 | 5.502064168 | 0.022569496 | 0.308045372 |
| RPL7A | -0.777186612 | 1.757056506 | 17.34570869 | 0.000107071 | 0.035090707 |
| PER1 | 0.776821698 | 5.004505631 | 6.097687263 | 0.016632932 | 0.272290215 |
| AICDA | 0.772587616 | -1.059409498 | 5.918956991 | 0.018150333 | 0.282624938 |
| SMPD3 | 0.76992982 | 4.0252467 | 6.502968058 | 0.01355232 | 0.247753847 |
| SMARCA1 | -0.768774765 | -0.943604968 | 13.02849834 | 0.000649379 | 0.06860427 |
| BFSP2 | 0.767849789 | -1.338270762 | 9.566014516 | 0.003071405 | 0.138217927 |
| TPX2 | 0.766117386 | 1.797496991 | 8.339547074 | 0.005493832 | 0.180394945 |
| EIF5A | -0.765462706 | 2.073021145 | 4.034650804 | 0.049424747 | 0.420018181 |
| CACNG8 | 0.764766271 | 2.293678041 | 6.059597635 | 0.016935508 | 0.274600625 |
| NPAS2 | -0.764055376 | 0.979846628 | 12.1631206 | 0.000948001 | 0.08171281 |
| HBA2 | 0.761601369 | 13.02672776 | 5.712816944 | 0.020192178 | 0.294048591 |
| LAMB2 | 0.76058216 | 1.543497711 | 10.20350622 | 0.002290706 | 0.118444397 |
| RAB3IL1 | 0.757695883 | 0.179512456 | 5.083973994 | 0.028041949 | 0.33993868 |
| UNC5B | 0.751065265 | -0.231805586 | 5.320810313 | 0.024751022 | 0.322104152 |
| CDC45 | 0.750775818 | 0.079002594 | 6.508720799 | 0.01345745 | 0.24665707 |
| PDZK1IP1 | 0.749609337 | 4.276408417 | 6.016602808 | 0.017334177 | 0.277023525 |
| DYNLT5 | 0.74862441 | -0.125867219 | 4.729200757 | 0.03385086 | 0.365003628 |
| CDC20 | 0.748133977 | 0.848195299 | 5.806135054 | 0.019259429 | 0.288365885 |
| B4GALNT4 | -0.746063032 | -0.46206898 | 5.895164057 | 0.018379687 | 0.284266477 |
| CRACD | 0.743357264 | -1.125432218 | 6.530325454 | 0.013306925 | 0.246483618 |
| MELK | 0.743121905 | 0.322703066 | 9.128480992 | 0.003768665 | 0.152527385 |
| CLDN5 | 0.740689848 | 1.747626394 | 6.165468275 | 0.016036719 | 0.268472991 |
| CCNB2 | 0.739036362 | 1.02478137 | 7.348568212 | 0.008867742 | 0.214958011 |
| SLC16A10 | -0.737874321 | 2.341147956 | 21.00726551 | 2.56E-05 | 0.023045214 |
| TMEM132C | -0.7378708 | -0.857344518 | 6.707191111 | 0.012177164 | 0.242247223 |
| OLFM1 | 0.736848173 | 1.859483973 | 7.324000466 | 0.008988657 | 0.216249035 |
| CYP2J2 | -0.736718715 | -0.934261217 | 11.28749855 | 0.001399244 | 0.097312715 |
| SMIM24 | 0.736554538 | 2.05878992 | 6.027557001 | 0.017208877 | 0.277023525 |
| CENPU | 0.733917731 | 1.09321001 | 16.76055961 | 0.000135661 | 0.036390791 |
| NBEA | -0.733736157 | 1.335162706 | 16.22511311 | 0.000168801 | 0.038928222 |
| CACNA1D | 0.733162126 | 0.53026782 | 16.12017689 | 0.000176229 | 0.038928222 |
| KIF14 | 0.732804104 | -0.303297641 | 4.371883242 | 0.041036755 | 0.395212462 |
| LINC00989 | 0.732584491 | 2.780920223 | 6.016507361 | 0.017319554 | 0.277023525 |
| DNAH6 | -0.731122852 | 0.964147364 | 14.46058773 | 0.000351801 | 0.054015226 |
| SLC35G2 | 0.730411345 | 0.625458965 | 10.46384278 | 0.002030958 | 0.11398378 |
| HES4 | 0.728971064 | 1.841064663 | 4.876078051 | 0.031337249 | 0.35252332 |
| HYAL3 | 0.726132936 | 1.55565935 | 25.71936913 | 4.51E-06 | 0.006445499 |
| SKA3 | 0.726035258 | -0.762855729 | 4.792411628 | 0.03271035 | 0.36011026 |
| LOC101593348 | 0.722455134 | -1.221115918 | 9.563852618 | 0.003074496 | 0.138217927 |
| MMP9 | 0.722152615 | 6.342160055 | 4.087625035 | 0.048019281 | 0.418192865 |
| PF4 | 0.717073231 | 5.473810831 | 6.875778371 | 0.011249892 | 0.235461555 |
| RDUR | 0.716717231 | -0.419669382 | 7.474887509 | 0.008327999 | 0.211025938 |
| CCR8 | 0.714534596 | 0.501540214 | 12.54234186 | 0.000802551 | 0.075673438 |
| LOC105377267 | 0.714256885 | 4.062327326 | 4.341818414 | 0.041794397 | 0.397578484 |
| CKB | 0.712486322 | 1.929874235 | 11.64526028 | 0.00119378 | 0.091877879 |
| NCAPG | 0.711148075 | 1.132594117 | 6.560764779 | 0.013119399 | 0.245487186 |
| HJURP | 0.711134858 | 0.748352684 | 6.298505168 | 0.014968129 | 0.260586391 |
| PLEKHD1 | -0.710755176 | -0.486504256 | 6.243124557 | 0.015384573 | 0.263778801 |
| PIK3R6 | 0.710558645 | 4.619567834 | 13.28367116 | 0.000587082 | 0.067123506 |
| CNKSR3 | 0.710510888 | -0.75762519 | 4.782490191 | 0.032882853 | 0.360376576 |
| PKP2 | 0.709214244 | 0.90164815 | 13.2781243 | 0.000582903 | 0.067123506 |
| GPR82 | 0.708936675 | 1.51116473 | 8.841870566 | 0.004318237 | 0.162497566 |
| LOC124902314 | 0.70870675 | -1.086573102 | 6.822465099 | 0.01149583 | 0.237364724 |
| LOC124903833 | 0.708480668 | -1.263532286 | 7.078397139 | 0.01012319 | 0.226195569 |
| HSPG2 | -0.70800436 | 0.7580179 | 24.88304533 | 6.08E-06 | 0.007960541 |
| ALDH1A3-AS1 | 0.70757525 | -1.041997105 | 10.28958422 | 0.002199345 | 0.115816285 |
| WFIKKN1 | 0.707547957 | -0.173800445 | 6.309836419 | 0.014873061 | 0.259462457 |
| VSIG4 | 0.70524148 | 2.034062642 | 4.698579731 | 0.034450475 | 0.36946288 |
| KIF4A | 0.704226289 | 0.057738765 | 4.639668774 | 0.035511791 | 0.371589161 |
| DEPDC1B | 0.703764363 | 0.39279759 | 8.545083383 | 0.004966798 | 0.173470764 |
| MEIS2 | 0.701076175 | -1.301798073 | 4.793528944 | 0.032689627 | 0.36011026 |
| SEPT5-GP1BB | 0.700071372 | 0.103407066 | 9.363287673 | 0.003375941 | 0.144699489 |
| GPC4 | 0.698794278 | -0.14081526 | 4.41813955 | 0.040018065 | 0.392089439 |
| LPL | 0.698133791 | 0.803410143 | 4.528573748 | 0.037722254 | 0.38237057 |
| EDAR | -0.698002904 | 2.394035294 | 16.28601549 | 0.000164745 | 0.038928222 |
| PTPRF | 0.698000155 | -0.329664084 | 5.884106892 | 0.018477709 | 0.285197233 |
| THRB | 0.696309419 | -0.624502299 | 6.990134172 | 0.010575885 | 0.230532458 |
| CDKN3 | 0.692709986 | -0.014177996 | 8.068797574 | 0.006240491 | 0.18876876 |
| DSC1 | -0.692350267 | 1.03035915 | 11.70570066 | 0.001160839 | 0.090572058 |
| PLCH1 | 0.692190908 | -1.231332647 | 5.524739663 | 0.022245002 | 0.30661895 |
| NUDT9P1 | -0.692184714 | -1.450829284 | 11.16524883 | 0.001478211 | 0.099773348 |
| LOC124901809 | -0.690845152 | 0.856082278 | 17.64228447 | 9.50E-05 | 0.035090707 |
| GJA3 | -0.688836153 | -0.73579004 | 7.255382105 | 0.009276002 | 0.218525645 |
| PDK4 | 0.68680304 | 4.812125437 | 17.54937893 | 9.98E-05 | 0.035090707 |
| LOC105369322 | 0.685661152 | -0.913622489 | 5.340863057 | 0.02448227 | 0.320783834 |
| PFKFB2 | 0.685612022 | 4.787033333 | 4.915519799 | 0.030721364 | 0.350933303 |
| SLC2A5 | 0.685123222 | 2.21340133 | 6.021600942 | 0.01725963 | 0.277023525 |
| GFI1B | 0.684943024 | 3.914340604 | 12.12561994 | 0.000970674 | 0.082140231 |
| PLXDC1 | -0.683425858 | 3.954721487 | 18.65030876 | 6.43E-05 | 0.033945237 |
| CCR9 | -0.682915886 | 1.922232793 | 8.467876592 | 0.005165573 | 0.176783176 |
| ARHGEF4 | -0.682658859 | 0.779708859 | 15.81118567 | 0.00020014 | 0.042552167 |
| E2F2 | 0.682587571 | -1.375162425 | 4.316009346 | 0.042286407 | 0.400171212 |
| STYK1 | 0.682005343 | 0.954294814 | 16.89969358 | 0.000128212 | 0.035750077 |
| GRAPL | -0.681693316 | 0.975306496 | 7.531702078 | 0.008105801 | 0.20889631 |
| CYP7B1 | 0.680045447 | -0.000535199 | 6.515586874 | 0.013405956 | 0.246483618 |
| ZNF185 | -0.679529708 | 1.372247047 | 5.34045104 | 0.024525957 | 0.320893845 |
| MINAR1 | 0.676493135 | 0.99135135 | 9.335475188 | 0.003420128 | 0.145796883 |
| CHEK1 | 0.675238991 | 1.012196088 | 10.4422018 | 0.002051115 | 0.11398378 |
| MYL4 | 0.674430596 | 4.932176893 | 5.862594127 | 0.01875417 | 0.285380723 |
| CDK1 | 0.674289363 | 0.907447315 | 6.048681074 | 0.016994975 | 0.274779024 |
| ZNF718 | 0.673721683 | 2.647455263 | 9.028043537 | 0.003963276 | 0.156770612 |
| ZNF667-AS1 | -0.673706728 | 1.445005645 | 17.32658391 | 0.000107898 | 0.035090707 |
| LINC01127 | 0.670294491 | 3.071926171 | 4.610884276 | 0.036129029 | 0.374172702 |
| CALD1 | 0.668908842 | 0.817261369 | 5.033216199 | 0.028803745 | 0.341448168 |
| MACIR | 0.667793972 | 3.031310593 | 10.60887553 | 0.001908671 | 0.110800624 |
| SPARC | 0.667196335 | 6.605792149 | 5.408591561 | 0.023710473 | 0.315834301 |
| TNR | -0.66707141 | -0.942057077 | 10.4007327 | 0.002090327 | 0.114393594 |
| SEMA5A | 0.666872669 | -0.655926585 | 11.52592079 | 0.001257656 | 0.095073207 |
| IGKV2-28 | -0.664556725 | 0.348059471 | 4.661684704 | 0.035105624 | 0.370876748 |
| RBM20 | -0.66454404 | -0.608791974 | 11.18493817 | 0.001465184 | 0.099288115 |
| SPEG | -0.66354974 | 1.966893631 | 18.47032872 | 6.84E-05 | 0.033945237 |
| SPNS3 | 0.663396719 | 3.486988817 | 8.079986446 | 0.006231602 | 0.18876876 |
| EXO1 | 0.663228334 | -0.052439284 | 7.009743191 | 0.010473495 | 0.229995514 |
| LOC105374869 | -0.662029225 | -0.440328451 | 11.09417957 | 0.001526246 | 0.101021567 |
| LOC101927401 | -0.65984267 | 0.962220968 | 18.37749401 | 7.09E-05 | 0.033945237 |
| HRH4 | 0.658646093 | 3.166658872 | 5.552482963 | 0.021986958 | 0.306251806 |
| GATA6 | 0.657074605 | -1.399818957 | 4.398777609 | 0.040426102 | 0.393499336 |
| LINC00540 | -0.656341609 | -0.546422441 | 9.980175499 | 0.002535332 | 0.12391799 |
| TROAP | 0.656307647 | -0.429568239 | 5.045934963 | 0.028588138 | 0.341366779 |
| PKMYT1 | 0.655998072 | 0.185706858 | 5.151759726 | 0.027043622 | 0.336677391 |
| PDE3A | 0.655039514 | -0.689091973 | 5.475544128 | 0.022821341 | 0.310038494 |
| ESCO2 | 0.654109398 | 0.216957053 | 8.160084082 | 0.00597208 | 0.185836125 |
| PHGDH | -0.653993534 | 1.520177972 | 17.0148845 | 0.000122367 | 0.035686036 |
| LOC105370668 | 0.652562735 | -1.209046164 | 4.141645187 | 0.046515619 | 0.412521606 |
| LOC105374399 | 0.651980645 | -1.407717026 | 7.050116678 | 0.010265967 | 0.227876666 |
| LOC124902709 | 0.6518761 | -1.140008196 | 6.41462111 | 0.014105719 | 0.254426486 |
| NELL2 | -0.650938244 | 5.956501696 | 17.10044644 | 0.000119644 | 0.035686036 |
| HBQ1 | 0.649970263 | 3.132555931 | 4.673183891 | 0.034939956 | 0.369883846 |
| UICLM | 0.649005046 | 2.491593045 | 8.350799881 | 0.005463893 | 0.180107282 |
| ABLIM3 | 0.648671119 | 3.010086906 | 4.434488171 | 0.039730509 | 0.390848016 |
| NEIL3 | 0.648251993 | -0.620288528 | 4.671440666 | 0.034896457 | 0.369883846 |
| FCGBP | -0.648142622 | 1.352531335 | 9.240983876 | 0.003575482 | 0.149372 |
| NRGN | 0.64748852 | 7.196729566 | 6.817840092 | 0.011568896 | 0.237364724 |
| EREG | 0.645429787 | -0.400199523 | 8.124359404 | 0.006075645 | 0.187551084 |
| GREM2 | 0.645330519 | -0.546567538 | 5.155574657 | 0.026981669 | 0.336459826 |
| KCNE1 | 0.644833274 | 1.485837483 | 6.6256933 | 0.012693764 | 0.243208759 |
| COPG2IT1 | -0.642915253 | -0.869794928 | 7.80311734 | 0.007096445 | 0.199509814 |
| SAMSN1 | 0.642062529 | 5.64806325 | 9.485295405 | 0.00321011 | 0.141293756 |
| ZNF667 | -0.641976259 | 0.486233677 | 8.022701649 | 0.006380824 | 0.188991778 |
| ESPN | 0.640725971 | 3.050342945 | 5.408061513 | 0.023699223 | 0.315834301 |
| ESPNL | -0.640578673 | -1.240833284 | 7.384858378 | 0.008703822 | 0.213484463 |
| ADGRA3 | -0.639838959 | 0.855723369 | 8.504260076 | 0.005064407 | 0.174441684 |
| ARHGAP29 | 0.639262392 | -0.436881114 | 5.061752082 | 0.028350191 | 0.341024332 |
| SPTA1 | 0.638553854 | 0.431846331 | 4.357563392 | 0.041356305 | 0.39607511 |
| CERCAM | 0.638060243 | 1.82251785 | 7.414447291 | 0.008586272 | 0.212424218 |
| LILRA5 | 0.637234654 | -0.552263373 | 5.803479951 | 0.01925943 | 0.288365885 |
| NPRL3 | 0.636363197 | 6.353985428 | 6.636908735 | 0.012669154 | 0.243208759 |
| NUAK1 | 0.63560191 | 0.403552874 | 4.579704547 | 0.03667364 | 0.376494491 |
| MTARC1 | 0.635486046 | 6.040657516 | 6.031036675 | 0.017207084 | 0.277023525 |
| BACE2 | 0.634249549 | 3.43059823 | 15.46769573 | 0.00023136 | 0.045394522 |
| NRXN2 | 0.634181501 | -0.852661551 | 4.797553535 | 0.032619484 | 0.360042048 |
| SLC5A9 | 0.633931485 | 1.390270386 | 4.959973746 | 0.029953469 | 0.347766935 |
| OBSCN | -0.632553776 | 5.192709865 | 25.72154468 | 4.55E-06 | 0.006445499 |
| TRIP13 | 0.632070396 | 0.123621573 | 6.364808691 | 0.014465125 | 0.257146927 |
| KRT73-AS1 | -0.631320048 | 0.426608691 | 7.534746505 | 0.008087624 | 0.208744151 |
| MKI67 | 0.630696368 | 3.446445784 | 5.296216635 | 0.025127715 | 0.325264313 |
| NUP160 | -0.630641175 | 1.806432055 | 11.08399492 | 0.001533262 | 0.101021567 |
| TRAV39 | -0.630499948 | 0.086555939 | 12.59152545 | 0.00078547 | 0.075673438 |
| FAM178B | -0.629678157 | -1.194862388 | 7.659456966 | 0.007610017 | 0.204162115 |
| NCAPH | 0.629055126 | 1.447393593 | 12.78455839 | 0.00072201 | 0.071816786 |
| INSC | -0.628660878 | 0.086300419 | 6.8639919 | 0.011260367 | 0.235461555 |
| SMG1P3 | 0.628601597 | 0.173923496 | 6.649926691 | 0.0125314 | 0.243208759 |
| LOC112268067 | -0.627985529 | 4.046782292 | 4.254824223 | 0.043820338 | 0.405516941 |
| CCNA2 | 0.627536763 | 1.885099947 | 7.048269499 | 0.010287 | 0.227876666 |
| DZIP1L | 0.626756931 | -0.189227474 | 4.188159162 | 0.045352364 | 0.409579144 |
| FAM118A | -0.625355261 | 5.92926561 | 4.428478337 | 0.039876578 | 0.391312607 |
| RETREG1-AS1 | -0.625304392 | -0.773697554 | 11.86763431 | 0.001080293 | 0.087386124 |
| PTX3 | 0.622493021 | 1.28873658 | 10.31447288 | 0.002174427 | 0.115816285 |
| HRK | 0.621723708 | 1.551315694 | 5.058765073 | 0.028428449 | 0.341366779 |
| TK1 | 0.621562477 | 1.809709027 | 7.955510837 | 0.006596816 | 0.191015842 |
| EPPK1 | -0.621080694 | 1.38220567 | 12.36499222 | 0.000867434 | 0.078479712 |
| FPR3 | 0.621052258 | 1.307299044 | 5.638112465 | 0.020987913 | 0.299436092 |
| CALHM5 | 0.618425311 | -0.973182142 | 6.525510884 | 0.01333919 | 0.246483618 |
| PDGFRB | 0.61821241 | 2.672282481 | 6.495016444 | 0.013575989 | 0.247753847 |
| POLQ | 0.618121688 | 0.392556456 | 6.743637561 | 0.011957239 | 0.240402698 |
| CELA1 | -0.617308614 | -0.84744081 | 7.928009041 | 0.006679631 | 0.192239994 |
| LOC105376995 | 0.617020258 | -0.030815671 | 4.334588258 | 0.04186774 | 0.397837085 |
| SLC25A39 | 0.616743137 | 9.116508247 | 4.343236384 | 0.041741259 | 0.397578484 |
| ACOT11 | 0.616555561 | 0.669289908 | 6.11362835 | 0.016431549 | 0.27099346 |
| DCHS1 | -0.616141729 | 3.467986399 | 18.09321403 | 7.96E-05 | 0.033945237 |
| LOC105378005 | -0.615249954 | -0.700030619 | 10.99882836 | 0.001593271 | 0.10307774 |
| RIMKLA | 0.614818765 | -0.947020511 | 6.386141758 | 0.014310028 | 0.255940342 |
| SLC24A3 | 0.614733482 | 1.956571126 | 8.793245446 | 0.004417488 | 0.163668988 |
| HBB | 0.613662198 | 16.52030242 | 4.120122363 | 0.047068638 | 0.414384298 |
| TKTL1 | 0.61359945 | 3.21523831 | 4.283961543 | 0.0431131 | 0.402783462 |
| PRG2 | 0.612199076 | -0.597640102 | 5.427501138 | 0.023399583 | 0.314130623 |
| MT1E | 0.611260489 | 0.464220614 | 7.073688149 | 0.010146817 | 0.226195569 |
| SEC14L5 | 0.611191638 | 0.985960134 | 4.551810632 | 0.037239211 | 0.379282476 |
| CROCC2 | 0.610876146 | 1.501302429 | 6.643337389 | 0.0125806 | 0.243208759 |
| LOC105374426 | 0.609121847 | -1.198363806 | 10.10885398 | 0.002389501 | 0.120245629 |
| FDPSP2 | 0.605744133 | -1.152091748 | 8.193164351 | 0.005877836 | 0.184576822 |
| FRRS1 | 0.605710893 | 3.140006051 | 13.40902859 | 0.000551989 | 0.067123506 |
| DUSP4 | 0.605170677 | 0.693071661 | 10.85909082 | 0.001697109 | 0.106125479 |
| TTC7B | 0.605134757 | 2.796653301 | 11.57306365 | 0.001233364 | 0.09407307 |
| LOC283194 | 0.602958996 | -0.914690108 | 9.426223653 | 0.003278147 | 0.142969221 |
| SLC22A16 | 0.602638549 | 1.215030936 | 5.304966915 | 0.024958996 | 0.324314414 |
| TDRP | 0.602088492 | 0.900454817 | 4.423151111 | 0.039915772 | 0.391312607 |
| HMGB3 | 0.601721222 | 1.060486139 | 17.24719727 | 0.000111406 | 0.035090707 |
| LOC105372879 | -0.601623256 | 0.444665456 | 20.98770687 | 2.57E-05 | 0.023045214 |
| C4BPB | -0.600354402 | -1.390911634 | 5.833890208 | 0.01896053 | 0.286157634 |
| LINC02615 | -0.600311081 | -0.305837584 | 10.33360804 | 0.00215547 | 0.115816285 |
| E2F7 | 0.598007267 | -0.646146426 | 4.812486767 | 0.032360625 | 0.359283204 |
| TMEM40 | 0.597462606 | 2.484289309 | 6.942587635 | 0.010849972 | 0.233014111 |
| EFCAB8 | 0.596973807 | 0.605187461 | 8.167820296 | 0.005949898 | 0.185836125 |
| LINC00853 | 0.595510545 | -1.164661505 | 4.238410468 | 0.044114803 | 0.406693053 |
| KNL1 | 0.592760729 | 1.270596121 | 7.092524725 | 0.010052653 | 0.226195569 |
| CCDC163 | 0.59228571 | 0.777233348 | 6.744322846 | 0.011953145 | 0.240402698 |
| TRABD2A | -0.591616121 | 5.251327641 | 23.39158605 | 1.05E-05 | 0.011946479 |
| LINC00563 | -0.591444174 | -0.698865738 | 6.201838596 | 0.015710459 | 0.266419936 |
| LINC00997 | 0.590980226 | 0.933918319 | 4.471910776 | 0.038872119 | 0.388231661 |
| LOC124901419 | 0.589925848 | -1.125569668 | 6.703980873 | 0.012196739 | 0.242309305 |
| SAP30 | 0.588685046 | 3.036227297 | 8.264112265 | 0.005696175 | 0.182116995 |
| ZNF135 | -0.588487963 | 0.941983738 | 8.349661867 | 0.005452722 | 0.180107282 |
| ITGB5 | 0.588411809 | 3.693075058 | 5.899827458 | 0.018383981 | 0.284266477 |
| GATM | -0.587708344 | 1.103190646 | 10.22536414 | 0.002265032 | 0.1181777 |
| SPC24 | 0.587180701 | -0.227135584 | 4.714802282 | 0.034094851 | 0.366573527 |
| LOC101929045 | -0.586296188 | -0.844196906 | 4.412840775 | 0.040118899 | 0.392625059 |
| NUSAP1 | 0.586170144 | 2.674454969 | 13.55538315 | 0.000517314 | 0.064226228 |
| FAXDC2 | 0.585788045 | 4.10272181 | 5.152436947 | 0.027098086 | 0.336677391 |
| CYSLTR2 | 0.585741041 | 4.299616904 | 7.378959144 | 0.008766667 | 0.213787593 |
| CYP4F12 | 0.585679734 | 2.887358116 | 6.121951413 | 0.016399299 | 0.270811339 |
| RORB | 0.585477846 | -1.434055701 | 4.302158859 | 0.042606649 | 0.401271592 |
| TRNP1 | 0.585316643 | -0.223388576 | 4.585298616 | 0.036549741 | 0.376299122 |
| BCL2L15 | 0.58361479 | 1.542376517 | 6.986855079 | 0.010596542 | 0.230532458 |
| TMCC2 | 0.582438244 | 4.17418583 | 4.618175537 | 0.035998894 | 0.374172702 |
| LOC124902699 | 0.581566718 | -0.916377229 | 6.917431055 | 0.010964841 | 0.233671688 |
| ANKRD18A | 0.581027126 | -0.77645164 | 5.660214101 | 0.020736656 | 0.297645385 |
| CTDSPL | 0.580373975 | 2.179039553 | 5.204668274 | 0.026329962 | 0.331310365 |
| LOC124902052 | -0.579584851 | 0.324374556 | 17.58727502 | 9.72E-05 | 0.035090707 |
| LOC124900465 | 0.579473246 | -0.981892657 | 7.453392087 | 0.008416148 | 0.211025938 |
| KLHL29 | -0.577743583 | 0.042206681 | 9.927031446 | 0.002598253 | 0.125194563 |
| GDF10 | -0.577505335 | -1.638265221 | 4.088079904 | 0.047905207 | 0.417794966 |
| GPR153 | 0.577279928 | 1.988970385 | 9.124739872 | 0.003775297 | 0.152527385 |
| NT5E | -0.57718278 | 4.043753779 | 13.18093281 | 0.000611348 | 0.067123506 |
| LOC105373488 | -0.575308219 | -0.225758264 | 8.359797076 | 0.00542633 | 0.180107282 |
| TMEM273 | 0.57473255 | 5.850072131 | 14.03416511 | 0.000424764 | 0.057339756 |
| IGHJ6 | -0.574059122 | -0.990968965 | 6.785842882 | 0.011707806 | 0.23877467 |
| LOC124908011 | 0.572633602 | 0.020486018 | 4.255533815 | 0.043704108 | 0.405102543 |
| TFPI | 0.572477799 | -0.643121275 | 4.933608399 | 0.030340778 | 0.3489292 |
| MGLL | 0.572221866 | 3.791906561 | 6.733242603 | 0.012056659 | 0.241361502 |
| LMNA | 0.571545937 | 4.177976784 | 14.99376433 | 0.000282438 | 0.049020357 |
| PLLP | -0.57095923 | -0.369032288 | 7.607225819 | 0.007806325 | 0.207074613 |
| TXNRD3 | -0.569848554 | -0.102883754 | 11.6548075 | 0.00118743 | 0.091804501 |
| IRAK3 | 0.569834926 | 7.016083929 | 6.96960102 | 0.01072582 | 0.2324763 |
| JDP2-AS1 | 0.568347819 | 0.215781407 | 8.174862146 | 0.005929783 | 0.185745256 |
| VSIG2 | 0.5680118 | 2.842786533 | 6.322986872 | 0.014804938 | 0.25907118 |
| B3GALNT1 | -0.567851961 | -0.094617612 | 5.236441479 | 0.025858814 | 0.32896976 |
| TMEM50B | 0.567600549 | 1.026045506 | 4.804352589 | 0.032513166 | 0.359748205 |
| LINC02446 | -0.567314454 | 3.859808198 | 9.517705722 | 0.003155621 | 0.140140902 |
| MFSD2B | 0.566597568 | 1.571265209 | 4.403646618 | 0.040355415 | 0.393499336 |
| GPRC5D-AS1 | -0.566374398 | -0.957839396 | 4.712192975 | 0.034142524 | 0.366622591 |
| LOC107984356 | -0.565570224 | -0.588263902 | 7.297010787 | 0.009087825 | 0.217038321 |
| IGSF22 | -0.565482183 | -1.141421903 | 8.262313482 | 0.005685852 | 0.182116995 |
| AK5 | -0.56528999 | 2.879241423 | 12.13885397 | 0.00095932 | 0.08171281 |
| RNR1 | 0.564988312 | 10.45207103 | 6.916370615 | 0.010977913 | 0.233671688 |
| LOC124907727 | 0.564780679 | -0.342056726 | 4.677533831 | 0.034782601 | 0.369883846 |
| TRPC2 | 0.563729503 | -0.419718464 | 5.980125883 | 0.017590547 | 0.278323364 |
| LOC101927344 | 0.563565606 | 0.062389137 | 7.14020811 | 0.009818385 | 0.224518291 |
| SLC18A2-AS1 | 0.562609561 | 1.960616741 | 4.653490073 | 0.035271647 | 0.370957322 |
| CD9 | 0.56217908 | 4.623532086 | 8.931937057 | 0.0041536 | 0.15932487 |
| RAB44 | 0.562130963 | 4.835726465 | 7.319190705 | 0.009027949 | 0.216802679 |
| LOC107984214 | -0.561491038 | -1.337759084 | 5.772604893 | 0.019568036 | 0.290176435 |
| CCR7 | -0.561132356 | 7.367277191 | 20.5091657 | 3.09E-05 | 0.025051767 |
| LOC105377921 | 0.560812867 | -1.196489591 | 4.289539563 | 0.042900709 | 0.402783462 |
| MIR641 | -0.560428918 | -1.48471194 | 5.444705178 | 0.023190739 | 0.312065882 |
| CLEC1B | 0.56009016 | 1.045886316 | 6.521267638 | 0.013367694 | 0.246483618 |
| SLC6A4 | 0.559435608 | -0.241887636 | 7.462832193 | 0.008377315 | 0.211025938 |
| TUBA8 | 0.559065264 | 3.014315721 | 7.468068511 | 0.008374519 | 0.211025938 |
| LOC112268199 | -0.558022953 | 2.834679445 | 18.23707458 | 7.50E-05 | 0.033945237 |
| TMIGD2 | -0.55781088 | 3.360169469 | 16.41044362 | 0.000156512 | 0.038928222 |
| LOC107985876 | -0.557707958 | 2.277857419 | 11.44059574 | 0.001306523 | 0.096620249 |
| DACH1 | 0.55674601 | 2.160368376 | 7.602313858 | 0.007830943 | 0.207074613 |
| GFRA2 | 0.555852092 | 0.76760474 | 6.424158824 | 0.014037989 | 0.253995677 |
| CXCL9 | 0.555448762 | -0.476410952 | 4.554092315 | 0.03716932 | 0.379224181 |
| LINC02970 | 0.55453357 | 1.579813459 | 6.129279759 | 0.016307306 | 0.270520133 |
| LOC105375024 | -0.554435811 | -1.165647774 | 6.773164249 | 0.011782153 | 0.239429677 |
| LRRC63 | 0.554365623 | -0.736202133 | 6.068890963 | 0.016810624 | 0.273873928 |
| ERLIN1 | 0.554151994 | 5.209808176 | 7.391792227 | 0.00871224 | 0.213484463 |
| GALNT16 | -0.553148683 | -1.593101876 | 5.321560149 | 0.024730708 | 0.322086224 |
| ZNF578 | -0.552742286 | -0.028869885 | 12.40409739 | 0.000852672 | 0.078479712 |
| ADGRE1 | 0.552615921 | 6.307841253 | 7.042326713 | 0.010347349 | 0.228866135 |
| LOC124904314 | 0.552433092 | 0.623929021 | 6.1101838 | 0.016460411 | 0.27099346 |
| CD70 | 0.552390625 | 0.598298347 | 8.827504614 | 0.004343414 | 0.16308417 |
| CDCA5 | 0.552381786 | 0.984346993 | 4.873759942 | 0.031329525 | 0.35252332 |
| PDGFA | 0.551707065 | -0.649835323 | 6.185308648 | 0.015842984 | 0.267511485 |
| TFE3 | 0.551239847 | 2.054284759 | 4.277788301 | 0.043223163 | 0.403503174 |
| ZNF391 | -0.550939749 | 0.826634584 | 10.73774163 | 0.001793024 | 0.108532184 |
| C3AR1 | 0.550670656 | 5.485509015 | 6.570946672 | 0.013087526 | 0.245487186 |
| LOC112268090 | -0.550217447 | 0.402446968 | 7.276537371 | 0.009179864 | 0.217543447 |
| GUK1 | 0.550133523 | 8.16547431 | 9.368785375 | 0.003377371 | 0.144699489 |
| TNNT3 | -0.549799427 | -1.087815113 | 4.12118959 | 0.047041049 | 0.414355877 |
| LOC124905203 | 0.549699529 | -1.522558337 | 5.184581985 | 0.026573008 | 0.333073174 |
| GAS2L1 | 0.549698012 | 3.594151044 | 9.909302279 | 0.00262861 | 0.126299523 |
| CENPM | 0.548166115 | 0.338009926 | 6.650857332 | 0.012525556 | 0.243208759 |
| G0S2 | -0.548093193 | 0.881606653 | 4.137081647 | 0.046648948 | 0.412737595 |
| LOC102723750 | 0.547774524 | 3.438529626 | 8.027062651 | 0.006385901 | 0.188991778 |
| USP44 | -0.547292401 | 1.569738672 | 11.32535671 | 0.001375694 | 0.097312715 |
| BAG6 | 0.546294923 | 0.557437387 | 4.581508519 | 0.036624393 | 0.376494491 |
| LIF | 0.545969983 | -1.323419081 | 4.173752004 | 0.045703696 | 0.411046846 |
| CUBN | -0.545869278 | 2.85189934 | 11.32670276 | 0.001376288 | 0.097312715 |
| SATB2 | 0.545580438 | 0.115863825 | 7.84088607 | 0.00696758 | 0.197519286 |
| ELOVL7 | 0.545577921 | 2.032308743 | 4.530736208 | 0.037678748 | 0.38215732 |
| CARMIL1 | -0.545333455 | 2.294538088 | 15.63080778 | 0.000215637 | 0.0437774 |
| HGD | 0.54485783 | 1.078422319 | 7.620265233 | 0.007756822 | 0.207074613 |
| GNAI1 | -0.544757821 | 0.238825827 | 12.48388547 | 0.000823359 | 0.076527356 |
| RBFOX3 | -0.542553387 | -1.296331251 | 6.522359087 | 0.013360356 | 0.246483618 |
| SORCS2 | 0.541989604 | -0.010705045 | 9.780897433 | 0.002779849 | 0.132073891 |
| LOC107984192 | 0.541911633 | -0.322486301 | 4.112913842 | 0.047255448 | 0.41488911 |
| LOC100132249 | -0.541691053 | 0.873774842 | 5.848077829 | 0.018822773 | 0.285380723 |
| LOC100287036 | 0.54132637 | 1.172615783 | 4.936951541 | 0.030296008 | 0.348650073 |
| MYOSLID | 0.540920902 | -0.278366465 | 5.952483447 | 0.017841208 | 0.279173055 |
| FUT2 | -0.540254533 | -1.07892813 | 7.210599859 | 0.009483035 | 0.220381399 |
| LEFTY1 | 0.539804862 | -0.625046751 | 4.102970688 | 0.047514447 | 0.416155116 |
| FAM111B | 0.539648068 | 1.622595304 | 5.378297123 | 0.024018621 | 0.3179243 |
| LOC101927610 | -0.536748621 | -1.510181301 | 4.492881204 | 0.03841775 | 0.386426676 |
| MS4A7 | 0.536587768 | 5.93949929 | 10.96196327 | 0.001629342 | 0.103408505 |
| ADAM12 | -0.536456171 | 1.429608868 | 6.886249234 | 0.01113627 | 0.23442676 |
| KIAA1143 | 0.534594986 | -1.137399786 | 6.313764535 | 0.01484351 | 0.259212801 |
| ZNF662 | -0.534090196 | 1.262081632 | 10.69307537 | 0.001829741 | 0.108690202 |
| METTL21EP | 0.532819429 | -1.002582138 | 5.712437643 | 0.020184658 | 0.294048591 |
| GZMB | 0.532613889 | 6.513691311 | 8.334113695 | 0.005516459 | 0.18078892 |
| ATP6V0E2-AS1 | -0.532599075 | 1.730042904 | 16.82316397 | 0.000132255 | 0.036282723 |
| B3GAT1 | 0.532086155 | 4.319023756 | 5.506235861 | 0.022520677 | 0.307921372 |
| HSD3B7 | 0.531068877 | 1.949936802 | 9.057237583 | 0.003897085 | 0.155599804 |
| VEPH1 | 0.530779608 | 0.289272795 | 4.149695956 | 0.046310571 | 0.412521606 |
| TMEM204 | -0.53074866 | 4.962263677 | 14.52831785 | 0.000344149 | 0.053703003 |
| LINC02725 | 0.530371643 | -1.320667673 | 4.614822861 | 0.03597379 | 0.374172702 |
| LOC105369914 | 0.529999076 | -0.563188976 | 4.325824301 | 0.042061047 | 0.399005216 |
| GTSE1 | 0.529473805 | 0.793919316 | 4.61075894 | 0.036054331 | 0.374172702 |
| RAD51 | 0.528820594 | 0.540130483 | 6.820259583 | 0.01150848 | 0.237364724 |
| TGFB1I1 | 0.528549382 | -0.121576105 | 4.542831687 | 0.037395662 | 0.380420344 |
| LOC124901207 | 0.526730116 | -1.456285288 | 6.559884406 | 0.013110643 | 0.245487186 |
| TNIP3 | 0.526517217 | 0.479009052 | 10.05096108 | 0.002453993 | 0.121690844 |
| FKBP8 | 0.525830261 | 10.80348475 | 4.569056593 | 0.036896701 | 0.377421222 |
| RAB13 | 0.524549001 | 1.747755726 | 8.536766281 | 0.004986522 | 0.173802786 |
| CDKN2B-AS1 | 0.522491248 | -0.788061315 | 10.42845762 | 0.002064025 | 0.11398378 |
| LOC124902519 | -0.521175134 | -1.04456693 | 4.044232211 | 0.049076237 | 0.420018181 |
| MYEOV | 0.520653338 | -0.505726355 | 4.139862738 | 0.046561149 | 0.412521606 |
| RAMP1 | 0.520558341 | -0.724911012 | 4.068196106 | 0.048432437 | 0.419090985 |
| GAL3ST4 | -0.519873955 | 3.124292142 | 14.09196384 | 0.000411304 | 0.05687701 |
| PRRG1 | 0.519027229 | -1.12820553 | 4.535618311 | 0.037541434 | 0.381508564 |
| SHCBP1 | 0.518836501 | 1.490952495 | 6.513653681 | 0.013419003 | 0.246483618 |
| CGN | -0.518343348 | -0.967253019 | 6.791422433 | 0.011675245 | 0.238682992 |
| COL6A4P2 | -0.517519069 | -0.46508492 | 6.262466791 | 0.015234372 | 0.2633889 |
| EGFL7 | 0.516829193 | 0.729118009 | 6.488482495 | 0.013590119 | 0.247753847 |
| LOC107986075 | -0.516125895 | -1.304489731 | 6.034447083 | 0.017108806 | 0.276094578 |
| LDB2 | -0.515590515 | -0.684735941 | 4.6711806 | 0.034901325 | 0.369883846 |
| CNN3 | -0.515392381 | 0.842730578 | 8.89624224 | 0.004204501 | 0.160245032 |
| CBX2 | -0.514757925 | -0.393764361 | 4.410756664 | 0.040164266 | 0.392749683 |
| LEF1 | -0.513749659 | 6.87305361 | 15.87761961 | 0.000195254 | 0.042039006 |
| RTN4RL1 | -0.513712516 | -0.826568455 | 4.244679519 | 0.04396396 | 0.405962539 |
| LOC105369747 | -0.513291055 | 0.85234751 | 12.91629754 | 0.00068179 | 0.070044476 |
| CLEC4D | 0.513159171 | 4.695961088 | 4.139697683 | 0.046663202 | 0.412737595 |
| LINC02561 | 0.512525951 | -1.031588617 | 5.004399029 | 0.029223239 | 0.342776359 |
| TRAV1-2 | -0.51132238 | 2.359119809 | 5.706036326 | 0.020276732 | 0.294272135 |
| ERVH48-1 | -0.510866384 | -0.868159547 | 6.632929311 | 0.012638633 | 0.243208759 |
| RBM26-AS1 | -0.51032697 | -0.044941257 | 5.788261757 | 0.019410888 | 0.288898446 |
| AMIGO1 | -0.509556911 | 4.4340917 | 19.83178736 | 4.02E-05 | 0.029430758 |
| CHST13 | 0.509179462 | 2.863557536 | 5.391907062 | 0.023874291 | 0.316786932 |
| DNASE1L2 | 0.508754274 | 1.015964231 | 12.09187705 | 0.000978267 | 0.082313025 |
| NEURL1 | 0.508595842 | 2.997855353 | 8.650396716 | 0.004728592 | 0.168613445 |
| GZMH | 0.508034304 | 7.03423595 | 7.563698163 | 0.007999808 | 0.207375909 |
| KIF2C | 0.507420924 | 1.049451491 | 5.718915754 | 0.020117284 | 0.294048591 |
| MTUS1 | -0.506602262 | 2.072048693 | 5.948384953 | 0.017889009 | 0.279663743 |
| PLEKHH2 | -0.504901846 | 0.034822707 | 4.946927777 | 0.03012706 | 0.348244588 |
| LOC105374334 | -0.504793913 | -0.358669947 | 10.7063008 | 0.001818788 | 0.108690202 |
| FAM72B | 0.503668651 | -0.900127445 | 6.719195125 | 0.01210426 | 0.241361502 |
| TMEM254-AS1 | -0.503259709 | 0.458199556 | 5.866608468 | 0.01864445 | 0.28533693 |
| EML5 | -0.503039087 | -0.000588823 | 13.24954742 | 0.000590139 | 0.067123506 |
| AMN | -0.50261197 | 0.092449021 | 10.97888949 | 0.001607672 | 0.103327155 |
| CPT1A | 0.50243417 | 6.27221338 | 16.96089812 | 0.000125425 | 0.035686036 |
| SNED1-AS1 | -0.501814021 | -1.378845434 | 4.178147288 | 0.045593747 | 0.410536817 |
| SELP | 0.501564416 | 3.209733239 | 6.182378312 | 0.015895613 | 0.267511485 |
| MIR12136 | 0.501260637 | -0.566470069 | 4.522599478 | 0.037806077 | 0.38253633 |
| CCDC194 | 0.500005481 | 0.433640185 | 5.103925852 | 0.027726054 | 0.339688116 |

| **Supplementary Table 3: DEGs from limma** | | | | | | |
| --- | --- | --- | --- | --- | --- | --- |
| Gene | logFC | AveExpr | t | P.Value | adj.P.Val | B |
| LOC107987462 | -2.881154773 | 1.228561832 | -2.979390366 | 0.004243866 | 0.1761692 | -2.082381057 |
| TFF3 | 2.391490511 | -1.705037184 | 4.433764453 | 4.29E-05 | 0.01622871 | 0.916752971 |
| CTSG | 2.266831703 | -0.276792331 | 3.343046808 | 0.001471354 | 0.104712398 | -1.232548076 |
| RNF182 | 2.259973372 | -0.004397008 | 2.296615182 | 0.025349778 | 0.371871947 | -3.374480651 |
| LOC105375878 | 2.062610953 | -2.303143324 | 3.526378242 | 0.000841232 | 0.080672346 | -1.145133814 |
| LINC02073 | 1.979391461 | -2.251304846 | 3.436244827 | 0.001109566 | 0.09056146 | -1.327968081 |
| AZU1 | 1.920296482 | -2.586422284 | 3.291803842 | 0.001715232 | 0.113141233 | -1.640424072 |
| LOC105378061 | 1.84835686 | -0.798483163 | 3.528326086 | 0.000836179 | 0.080672346 | -0.852207298 |
| CCL23 | 1.844439124 | -2.693781253 | 3.054718019 | 0.003427237 | 0.160148008 | -2.103433913 |
| CEACAM8 | 1.816403734 | 1.773344642 | 3.111488018 | 0.002911508 | 0.14699845 | -1.711590321 |
| MIR34AHG | 1.711418214 | -2.106850681 | 5.410742516 | 1.31E-06 | 0.00222477 | 3.136629448 |
| ROBO1 | -1.688663761 | -0.939024801 | -5.827124029 | 2.78E-07 | 0.000789367 | 5.716706158 |
| CD248 | -1.643686545 | 1.354625709 | -6.791963231 | 7.17E-09 | 0.000122039 | 9.838039348 |
| SHANK1 | -1.616118589 | -1.62249268 | -4.500386806 | 3.41E-05 | 0.014331674 | 1.632373965 |
| HBZ | 1.613859437 | -2.229382466 | 2.308328512 | 0.024643847 | 0.368592121 | -3.374032552 |
| SFRP5 | -1.603657988 | -0.364604253 | -4.486016676 | 3.59E-05 | 0.014331674 | 1.963077284 |
| CWC25 | 1.598859126 | 0.162121881 | 3.396299817 | 0.001252873 | 0.09642588 | -1.022915261 |
| TRPC6 | 1.595903254 | -0.206637532 | 3.836924801 | 0.000315175 | 0.048734622 | 0.03445097 |
| ASIC1 | -1.580326537 | -0.399498332 | -5.950803158 | 1.75E-07 | 0.000744333 | 6.373267293 |
| PXDN | -1.554843164 | -2.014900032 | -3.765980883 | 0.000395859 | 0.055189825 | -0.335697317 |
| NRCAM | -1.545502027 | 0.81834186 | -5.369487793 | 1.52E-06 | 0.002353909 | 4.922073446 |
| LTF | 1.543257368 | 4.74411668 | 3.15854431 | 0.0025401 | 0.137594117 | -1.912915386 |
| IL34 | 1.513383193 | -1.095302967 | 3.482606111 | 0.00096275 | 0.086642376 | -0.988298592 |
| AOC1 | 1.494945979 | 0.246765529 | 2.254630577 | 0.02802986 | 0.391513747 | -3.480571334 |
| LINC02009 | 1.487065177 | -0.441848684 | 2.762257436 | 0.007720523 | 0.229938983 | -2.480921128 |
| CEACAM6 | 1.463206015 | 0.886364855 | 2.65469801 | 0.010280492 | 0.260573156 | -2.727107916 |
| MMP8 | 1.440805795 | 2.378585337 | 2.499761341 | 0.015341276 | 0.308486683 | -3.202175643 |
| CTSE | 1.402264547 | -0.41026987 | 3.368347726 | 0.001363404 | 0.099417656 | -1.143121696 |
| REG4 | -1.379060759 | -0.814756895 | -5.882502402 | 2.26E-07 | 0.000769647 | 5.92275387 |
| LOC107984706 | -1.370323301 | -1.596868737 | -3.782889799 | 0.000374998 | 0.054053796 | -0.203244666 |
| ACAP1 | 1.368260077 | 2.921796494 | 2.704964897 | 0.009000385 | 0.247714488 | -2.819585574 |
| DEFA3 | 1.361053221 | 1.958324736 | 2.400003458 | 0.01969648 | 0.333019093 | -3.364813066 |
| LRRN3 | -1.347897441 | 3.881550114 | -5.684966608 | 4.74E-07 | 0.001150767 | 6.161996463 |
| VWDE | 1.345589147 | -1.815159044 | 3.012169183 | 0.003868445 | 0.168602845 | -2.097916299 |
| LCN2 | 1.343122076 | 3.042558129 | 2.846688199 | 0.0061372 | 0.20950801 | -2.496914943 |
| LOC107987207 | -1.334924416 | -0.798839939 | -3.601323894 | 0.000666349 | 0.072861687 | -0.516351025 |
| NHIP | -1.310176869 | -0.197114718 | -3.947161438 | 0.000220279 | 0.040725261 | 0.46213161 |
| LOC105369595 | 1.286584966 | -1.283339227 | 2.821394685 | 0.006576846 | 0.215956712 | -2.41073094 |
| OLR1 | 1.281647471 | 0.160319016 | 2.559417693 | 0.013172755 | 0.288731172 | -2.895534765 |
| S100P | 1.279974132 | 4.107838419 | 2.244707888 | 0.028698791 | 0.394388954 | -3.998980577 |
| LOC105370655 | -1.272229804 | 0.463642919 | -3.664656489 | 0.000546158 | 0.065883723 | -0.26732953 |
| LOC107985357 | 1.271115641 | -0.60351308 | 2.21853014 | 0.030531716 | 0.406907137 | -3.507693525 |
| CRISP3 | 1.240404806 | 1.849106023 | 2.445176039 | 0.017602768 | 0.317545 | -3.265707259 |
| HBG2 | 1.236299872 | 6.645282652 | 2.326969477 | 0.023556417 | 0.363255749 | -4.008330801 |
| CPLANE2 | -1.222913778 | -2.694934155 | -2.684097417 | 0.009512953 | 0.252033992 | -2.718435938 |
| MEG3 | 1.210365567 | -2.112412094 | 2.88598771 | 0.005507712 | 0.199320585 | -2.378817605 |
| OLFM4 | 1.210359209 | 1.627241515 | 2.211054052 | 0.031073785 | 0.409758041 | -3.709621747 |
| HRAS | -1.207037915 | -1.10750768 | -2.510390656 | 0.014932714 | 0.308486683 | -2.980800009 |
| LOC105377781 | 1.190146525 | -2.725062295 | 2.118436257 | 0.03852543 | 0.437451295 | -3.663741972 |
| TNNI2 | -1.189815266 | -1.077315038 | -2.437972453 | 0.017922626 | 0.320562595 | -3.119228085 |
| HNRNPL | -1.186943707 | 2.370881541 | -3.219838148 | 0.00212277 | 0.127134463 | -1.567585458 |
| LOC389834 | 1.184492497 | 1.486042821 | 2.193995715 | 0.032342486 | 0.416349015 | -3.733052841 |
| SERPINB10 | 1.184415708 | 0.609157616 | 3.041377465 | 0.00356024 | 0.161483008 | -1.852154184 |
| CACHD1 | -1.18157502 | 0.399805437 | -5.335447531 | 1.72E-06 | 0.002444963 | 4.706516871 |
| MICB-DT | 1.173869526 | -3.034505213 | 2.034100982 | 0.046621412 | 0.457935097 | -3.783192439 |
| COL4A1 | 1.169121956 | -2.722956371 | 2.250740584 | 0.028290439 | 0.392809866 | -3.47091405 |
| S100B | -1.166020728 | 1.597045488 | -3.287177005 | 0.001739038 | 0.113330653 | -1.317048047 |
| GATA6 | 1.154017654 | -2.074858999 | 3.283001678 | 0.001760788 | 0.114310104 | -1.593724421 |
| RNASE3 | 1.147749377 | 1.218454681 | 2.59429362 | 0.012037749 | 0.2752017 | -2.890819874 |
| HBG1 | 1.138911757 | 2.641806485 | 2.141229496 | 0.036559725 | 0.430640145 | -3.982702559 |
| BFSP2 | 1.128554717 | -1.859411916 | 4.061038064 | 0.000151388 | 0.035406146 | 0.158811506 |
| NOC4L | 1.12562027 | -0.212854556 | 2.014150248 | 0.04873898 | 0.464166467 | -3.878047509 |
| NOG | -1.118433562 | 2.782746469 | -5.066513291 | 4.59E-06 | 0.004594824 | 4.050193705 |
| RHAG | 1.117736997 | -1.848989856 | 2.527289953 | 0.014303377 | 0.302971525 | -2.990075562 |
| LOC112268261 | -1.102831426 | 1.372410471 | -4.59916683 | 2.42E-05 | 0.011756596 | 2.509680861 |
| MYCT1 | 1.098419806 | 0.530008812 | 4.821453053 | 1.11E-05 | 0.007677615 | 2.984155353 |
| CDK15 | 1.09391601 | -1.602087571 | 2.581898925 | 0.012430516 | 0.280040593 | -2.875241397 |
| OLIG2 | 1.091986045 | 2.125149052 | 2.717698557 | 0.008700174 | 0.244677002 | -2.708454293 |
| LOC105371498 | 1.090192114 | -0.546675104 | 2.461257113 | 0.016907185 | 0.313322893 | -3.073149948 |
| ADORA3 | 1.085778689 | 2.283846036 | 4.047004918 | 0.000158591 | 0.035493063 | 0.821206498 |
| GYPA | 1.085206932 | -1.551972719 | 2.712324222 | 0.008825742 | 0.246093519 | -2.633391209 |
| FBLN2 | -1.084071809 | 1.375743927 | -6.513365516 | 2.08E-08 | 0.000176739 | 8.860985142 |
| TMIGD3 | 1.075567761 | 0.179090553 | 2.688257622 | 0.009408682 | 0.250834272 | -2.632649864 |
| PLAAT5 | 1.074388979 | 0.172983598 | 3.187911622 | 0.002331358 | 0.132180208 | -1.510317726 |
| PIP4P1 | -1.06928863 | 1.239649035 | -2.435422755 | 0.018037086 | 0.320912965 | -3.296012295 |
| MGAM2 | -1.06653771 | -2.071634654 | -2.0315862 | 0.046883877 | 0.458342334 | -3.788275706 |
| ALDH1A3-AS1 | 1.052329916 | -1.4696386 | 4.020069958 | 0.000173355 | 0.035557323 | 0.212107604 |
| ACKR1 | 1.051130432 | -1.569330551 | 2.561997646 | 0.013085578 | 0.288182322 | -2.910439344 |
| LONRF2 | 1.045221532 | -1.89330098 | 2.601212181 | 0.011823454 | 0.272869908 | -2.860281411 |
| ADCK5 | 1.03258392 | -1.240691228 | 2.112066392 | 0.039091002 | 0.439728956 | -3.66874245 |
| CRACD | 1.030677292 | -1.734947278 | 2.78179569 | 0.007323846 | 0.22616136 | -2.518810349 |
| RYR2 | 1.029662347 | -1.599934226 | 3.437827886 | 0.00110422 | 0.09056146 | -1.160083688 |
| CYP7B1 | 1.021745396 | -0.442398732 | 3.01028975 | 0.003889105 | 0.168749476 | -1.94489561 |
| CARMIL3 | 1.019170453 | -2.322868274 | 2.408195009 | 0.019300983 | 0.331593267 | -3.216005171 |
| IFNG-AS1 | -1.017747572 | 1.506155145 | -3.822519215 | 0.00033016 | 0.049696432 | 0.167188996 |
| UNC5B | 1.013329445 | -0.841614213 | 2.628350173 | 0.011016001 | 0.265699504 | -2.75436201 |
| LOC107986639 | 1.00936173 | -1.515834632 | 2.787633038 | 0.007209026 | 0.22457568 | -2.484318334 |
| LOC124900812 | 1.009026905 | 2.987542163 | 2.912719987 | 0.005114292 | 0.192595541 | -2.351029258 |
| LOC105376568 | -1.007281937 | 1.410492181 | -3.122957864 | 0.002816555 | 0.145610261 | -1.721383694 |
| RCAN2 | 1.005083318 | 0.299212429 | 3.938793691 | 0.000226389 | 0.041404912 | 0.421104506 |
| LOC124901419 | 0.992626593 | -1.578224834 | 3.436625873 | 0.001108277 | 0.09056146 | -1.15145585 |
| SPATA3-AS1 | 0.986003116 | -0.702460525 | 3.151371893 | 0.002593683 | 0.13786234 | -1.649232355 |
| LOC112268067 | -0.98299062 | 3.577981862 | -3.071692395 | 0.003264713 | 0.155544845 | -2.10605438 |
| SLC4A10 | -0.973601124 | 3.191338698 | -2.889324953 | 0.00545711 | 0.199320585 | -2.519617968 |
| LOC101929698 | -0.966772586 | 0.614911638 | -5.496982925 | 9.52E-07 | 0.00202311 | 5.269250121 |
| LEF1-AS1 | -0.958780832 | 0.513911016 | -5.969339848 | 1.63E-07 | 0.000744333 | 6.772278436 |
| KIF26A | 0.954210699 | -1.975156934 | 2.193361601 | 0.032390513 | 0.416349015 | -3.539263016 |
| IER5L-AS1 | -0.953486894 | -1.529375563 | -3.588966669 | 0.000692574 | 0.073330745 | -0.673649285 |
| LYPD2 | 0.951546168 | 0.546650237 | 2.597906913 | 0.011925394 | 0.273960437 | -2.842690434 |
| TMEM132C | -0.951205749 | -1.462901871 | -3.038226215 | 0.003592354 | 0.162506244 | -1.912973129 |
| DEPDC1 | 0.950595386 | -1.604165034 | 2.624371387 | 0.01113115 | 0.267020625 | -2.795162979 |
| ECRG4 | -0.950024947 | -1.220252083 | -4.494929568 | 3.48E-05 | 0.014331674 | 1.715127186 |
| LOC107985900 | -0.943403177 | 0.197427666 | -4.65709946 | 1.98E-05 | 0.010497997 | 2.55661213 |
| IGHV3-49 | 0.931050489 | -2.092407263 | 2.12599623 | 0.037863466 | 0.434268173 | -3.643006223 |
| LOC102723345 | 0.927616259 | 0.20505937 | 2.150010293 | 0.035826198 | 0.429489821 | -3.686652061 |
| RNF212 | -0.927081518 | -0.228952359 | -4.881666915 | 8.92E-06 | 0.007229835 | 3.130582405 |
| TTC5 | -0.921023822 | -0.866124732 | -2.108959072 | 0.039369514 | 0.439728956 | -3.717986066 |
| LOC124901207 | 0.920483741 | -1.908880032 | 3.615952832 | 0.000636527 | 0.07183434 | -0.831051948 |
| UGT2B28 | 0.91915536 | -2.255438551 | 2.116636451 | 0.038684505 | 0.438072396 | -3.659363003 |
| MOCS1 | -0.917527664 | -1.075567994 | -3.133217534 | 0.002734087 | 0.143531128 | -1.672995745 |
| HBD | 0.917322724 | 3.891832304 | 2.25453906 | 0.028035966 | 0.391513747 | -3.974708182 |
| FBN1 | 0.906539286 | -0.178618046 | 3.041393495 | 0.003560078 | 0.161483008 | -1.860529342 |
| LOC124902787 | 0.906243307 | -0.919707814 | 2.506153942 | 0.015094368 | 0.308486683 | -2.987968279 |
| LINC02295 | -0.906171675 | 1.628299154 | -5.084821124 | 4.30E-06 | 0.004594824 | 4.083071234 |
| SCARA5 | -0.903082841 | -1.682890291 | -3.099574701 | 0.003013302 | 0.149459502 | -1.804735756 |
| ABCA13 | 0.903070202 | 0.460699101 | 2.087572566 | 0.041333727 | 0.444122151 | -3.81956672 |
| NPAS2 | -0.901792971 | 0.660132137 | -4.051746232 | 0.000156121 | 0.035406146 | 0.821574921 |
| HLA-DRA | -0.901242945 | 2.361399162 | -2.141580212 | 0.036530177 | 0.43059029 | -4.043214005 |
| LINC02086 | 0.900923735 | -0.436363241 | 2.771238954 | 0.007535782 | 0.229938983 | -2.456333852 |
| ACTN4 | -0.900638011 | 2.519684531 | -2.348404255 | 0.022359007 | 0.354501728 | -3.652711166 |
| FOXJ1 | -0.895181918 | -1.763846104 | -3.968632305 | 0.000205318 | 0.039684761 | 0.181527016 |
| PMP22 | 0.891858658 | 0.561598109 | 2.430421151 | 0.018263526 | 0.323006282 | -3.18867084 |
| OR10G2 | -0.891793241 | -1.612889153 | -2.725094759 | 0.008530044 | 0.242842681 | -2.571144049 |
| ALPK2 | 0.889855515 | -0.940864484 | 2.464021355 | 0.016790143 | 0.313322893 | -3.066665893 |
| LIPH | 0.875704267 | -1.328462011 | 2.915348963 | 0.005077044 | 0.192293069 | -2.210229509 |
| IDO1 | 0.875084466 | 3.400713445 | 2.854725624 | 0.006003287 | 0.207278859 | -2.555406974 |
| IARS1 | -0.864329948 | 0.691242417 | -2.219449677 | 0.030465622 | 0.406907137 | -3.662753028 |
| BPI | 0.863560091 | 3.172680338 | 2.144496199 | 0.036285312 | 0.429489821 | -4.078712747 |
| LOC124902699 | 0.860150669 | -1.311431429 | 3.343094858 | 0.001471142 | 0.104712398 | -1.295921462 |
| NBEA | -0.856716092 | 1.107834271 | -4.9695678 | 6.51E-06 | 0.005829117 | 3.658068302 |
| LOC124902314 | 0.85190008 | -1.592670458 | 2.591047363 | 0.012139511 | 0.276413574 | -2.853867718 |
| LOC102724008 | 0.84973921 | -0.704145653 | 2.96643435 | 0.004401396 | 0.181267189 | -2.052670874 |
| IL5RA | 0.847606236 | 4.338572836 | 2.826860398 | 0.00647945 | 0.214723553 | -2.75793221 |
| IL23R | -0.845279957 | -1.366231021 | -2.689185615 | 0.009385566 | 0.250665532 | -2.632935996 |
| SH3RF3-AS1 | -0.845037449 | -1.736237998 | -2.769149583 | 0.007578392 | 0.229938983 | -2.492674041 |
| CACNG6 | 0.843487094 | 1.492277703 | 2.248230232 | 0.028459736 | 0.393344073 | -3.64206756 |
| CEBPE | 0.842368657 | 2.89637189 | 3.21423446 | 0.002158062 | 0.127926532 | -1.576766361 |
| LOC124903833 | 0.840160515 | -1.823618333 | 2.619271065 | 0.011280363 | 0.268357563 | -2.816204424 |
| CA6 | -0.833686999 | 1.266386884 | -3.733622486 | 0.000438921 | 0.059182348 | -0.078153578 |
| LOC105369322 | 0.833186042 | -1.466276268 | 2.514071719 | 0.014793534 | 0.308486683 | -2.985951436 |
| SLC16A14 | 0.827555209 | 0.089087355 | 3.290163278 | 0.001723638 | 0.113141233 | -1.263860777 |
| CCDC187 | -0.821755179 | -2.040596505 | -2.299699409 | 0.025162178 | 0.37119123 | -3.360813215 |
| LGALS12 | 0.821575724 | 3.591648033 | 4.097371433 | 0.000134176 | 0.033156373 | 0.887668157 |
| LOC124905203 | 0.820051116 | -2.018030542 | 3.063183055 | 0.003345265 | 0.158054471 | -2.000561325 |
| NUDT9P1 | -0.817584089 | -1.937525728 | -3.51625764 | 0.000867958 | 0.081282842 | -0.93052965 |
| SMARCA1 | -0.814711342 | -1.371143181 | -3.387280734 | 0.001287574 | 0.097769429 | -1.126036429 |
| ZNF667 | -0.812566684 | 0.136949002 | -2.880643704 | 0.005589649 | 0.200281409 | -2.228464647 |
| TNNT1 | 0.811846952 | 1.378865691 | 2.247771152 | 0.028490793 | 0.393344073 | -3.630196058 |
| TICRR | 0.807068551 | -1.360276872 | 2.023999341 | 0.047683455 | 0.461346919 | -3.804918753 |
| SLC29A1 | 0.806467678 | 4.505059296 | 2.870496961 | 0.005748337 | 0.203107172 | -2.672538215 |
| ESAM-AS1 | 0.805828169 | -1.774681483 | 2.665579058 | 0.009990052 | 0.256677949 | -2.729255792 |
| LOC124902271 | 0.805063108 | -2.046359333 | 2.482299759 | 0.016034419 | 0.309217041 | -3.070868363 |
| GATA1 | 0.803770932 | 3.603755411 | 3.600408037 | 0.000668259 | 0.072861687 | -0.595092103 |
| LOC124902302 | 0.80334741 | -0.465480602 | 2.264925226 | 0.02735047 | 0.385695976 | -3.439161779 |
| KRT73-AS1 | -0.803048331 | 0.082578906 | -3.531860445 | 0.000827084 | 0.080672346 | -0.634024543 |
| FCGBP | -0.800457503 | 1.071806788 | -3.389379674 | 0.001279418 | 0.097585762 | -1.010478571 |
| CLC | 0.797866941 | 6.877807846 | 2.469403473 | 0.016564351 | 0.312085906 | -3.692490065 |
| FAM178B | -0.796081938 | -1.658813601 | -3.47560438 | 0.000983673 | 0.08751694 | -0.970837096 |
| TTK | 0.793001174 | -0.286351266 | 2.981748761 | 0.004215761 | 0.175749715 | -1.997212953 |
| ARHGEF4 | -0.791088212 | 0.561835009 | -4.643848157 | 2.07E-05 | 0.010664222 | 2.568111754 |
| LOC124900837 | -0.78929538 | -1.755283474 | -2.840413583 | 0.006243656 | 0.210293765 | -2.353351482 |
| CNKSR3 | 0.789121629 | -1.389754227 | 2.028756004 | 0.047180787 | 0.459000071 | -3.797184191 |
| RAB3IL1 | 0.787496864 | -0.391043618 | 2.082575564 | 0.041804765 | 0.4461432 | -3.757720336 |
| SLC25A26 | 0.786861884 | 0.918750366 | 2.228096915 | 0.029850227 | 0.402198619 | -3.618951815 |
| LOC101593348 | 0.786795457 | -1.667307979 | 2.911659541 | 0.005129389 | 0.192595541 | -2.248354255 |
| SUCNR1 | 0.786228625 | 0.197108567 | 3.166199348 | 0.002484058 | 0.135796569 | -1.558448083 |
| LOC105374426 | 0.784282584 | -1.562494087 | 3.550418849 | 0.000780854 | 0.079530187 | -0.880137927 |
| RBM20 | -0.782926732 | -0.949657154 | -4.169434037 | 0.000105462 | 0.029406555 | 0.911678314 |
| LOC105371401 | -0.780718883 | -1.798177947 | -2.372686713 | 0.021068391 | 0.344901123 | -3.233425479 |
| ERBB3 | -0.778157247 | -1.150562724 | -3.302334209 | 0.001662194 | 0.111748033 | -1.29561316 |
| DSC1 | -0.777740807 | 0.75885731 | -3.798849233 | 0.00035628 | 0.052695352 | 0.106110727 |
| PLEKHD1 | -0.775050066 | -1.023422494 | -2.690848839 | 0.009344265 | 0.250665532 | -2.622850277 |
| ZBTB20-AS1 | 0.774443205 | -1.134045784 | 3.533626191 | 0.000822576 | 0.080672346 | -0.823915182 |
| LOC105370355 | -0.773531681 | -1.623451657 | -2.341330252 | 0.022748038 | 0.358087416 | -3.287593272 |
| LAMB2 | 0.772935104 | 1.239014586 | 3.06360504 | 0.003341227 | 0.158054471 | -1.825009475 |
| ADGRA3 | -0.768459385 | 0.55099312 | -3.427539288 | 0.001139406 | 0.09056146 | -0.898464914 |
| CYP2J2 | -0.766055449 | -1.364996096 | -3.26459851 | 0.001859749 | 0.11629583 | -1.407856415 |
| DYNC1I2 | 0.764919704 | 1.282321948 | 2.179739215 | 0.033437523 | 0.420689598 | -3.751291536 |
| LOC124903484 | -0.75902998 | 0.348246655 | -2.025214885 | 0.047554563 | 0.460623892 | -3.971679297 |
| CEP55 | 0.757426219 | 0.341600193 | 2.762452115 | 0.007716475 | 0.229938983 | -2.482981202 |
| CFAP97D2 | -0.756474594 | -1.611343874 | -2.477059621 | 0.016247873 | 0.310516929 | -3.044366123 |
| LINC02751 | -0.756468968 | -1.955837519 | -2.068711944 | 0.043136105 | 0.450428112 | -3.732741748 |
| PIK3R3 | 0.755966508 | -1.721150132 | 2.947412219 | 0.004642562 | 0.185800804 | -2.182618203 |
| CACNG8 | 0.748969746 | 1.826104836 | 2.154171284 | 0.03548312 | 0.427651753 | -3.869324041 |
| EDAR | -0.748087035 | 2.197944079 | -4.39374448 | 4.93E-05 | 0.017682861 | 1.870192326 |
| PLXDC1 | -0.747411319 | 3.792916617 | -4.815609165 | 1.13E-05 | 0.007677615 | 3.15442335 |
| SLC16A10 | -0.744480766 | 2.162156311 | -4.710896921 | 1.63E-05 | 0.009267503 | 2.882240943 |
| TRBV28 | 0.743120383 | -0.610430944 | 2.168148348 | 0.034351676 | 0.421868345 | -3.602853439 |
| LOC107984214 | -0.741909159 | -1.854473729 | -2.793393226 | 0.007097351 | 0.223361139 | -2.454618479 |
| SBNO2 | -0.734338313 | 4.386557405 | -2.797179913 | 0.007024808 | 0.223336382 | -2.886077571 |
| LOC124901809 | -0.733650404 | 0.650957803 | -4.744565064 | 1.45E-05 | 0.009013264 | 2.888995079 |
| EPHB4 | -0.732265314 | 3.922723577 | -2.689103254 | 0.009387615 | 0.250665532 | -3.096735857 |
| LOC101927369 | 0.729167272 | -1.5684787 | 2.250973728 | 0.028274762 | 0.392809866 | -3.441636426 |
| IFIT1B | 0.72908998 | 2.458141684 | 2.035069091 | 0.046520711 | 0.457560939 | -4.176552872 |
| LOC101929322 | 0.728377001 | -0.309706518 | 2.463678992 | 0.0168046 | 0.313322893 | -3.076944787 |
| HSPG2 | -0.724265911 | 0.583582525 | -5.187081231 | 2.97E-06 | 0.003603599 | 4.259694189 |
| PHLDA3 | 0.723989728 | -1.156545096 | 2.475047342 | 0.016330521 | 0.311047954 | -3.047858408 |
| LOC107984356 | -0.72217762 | -0.961874662 | -2.725558734 | 0.008519474 | 0.242842681 | -2.551236116 |
| PRRG1 | 0.72116769 | -1.589925634 | 2.53679853 | 0.013959925 | 0.297922676 | -2.949193128 |
| KIF18B | 0.718988182 | -0.637886641 | 2.467998451 | 0.016623029 | 0.312632554 | -3.060780294 |
| PDK4 | 0.718163309 | 4.667774234 | 4.409438331 | 4.67E-05 | 0.017261288 | 1.778332862 |
| LOC124902519 | -0.715774454 | -1.587376046 | -2.296707847 | 0.025344123 | 0.371871947 | -3.36550351 |
| CCR8 | 0.713482267 | 0.25032323 | 3.711258837 | 0.000471267 | 0.060219706 | -0.182139955 |
| GPR82 | 0.71179591 | 1.215567041 | 2.951547586 | 0.004589111 | 0.185406644 | -2.095643886 |
| MPO | 0.710797597 | 2.514852112 | 2.303753556 | 0.02491746 | 0.369806736 | -3.668011993 |
| CENPU | 0.70880301 | 0.89271971 | 4.055365599 | 0.00015426 | 0.035406146 | 0.81757509 |
| CALHM5 | 0.70829624 | -1.397937591 | 2.539959441 | 0.013847419 | 0.297763265 | -2.935927709 |
| TRBV6-4 | -0.70688996 | -0.992557642 | -2.915637599 | 0.005072969 | 0.192293069 | -2.151352153 |
| CDKN2B-AS1 | 0.706819381 | -1.063946356 | 3.559353337 | 0.000759485 | 0.077819779 | -0.748830838 |
| SPEG | -0.706671235 | 1.798439499 | -4.799173744 | 1.20E-05 | 0.007825885 | 3.16040777 |
| SCN4B | 0.705332902 | -1.901267775 | 2.013155835 | 0.048846662 | 0.464671628 | -3.809641353 |
| NRXN2 | 0.70350077 | -1.387149716 | 2.185246353 | 0.03301072 | 0.418389965 | -3.549987373 |
| GALNT16 | -0.701796659 | -2.147881134 | -2.838178861 | 0.006281981 | 0.210749942 | -2.394033967 |
| NELL2 | -0.700628982 | 5.82791015 | -4.147576699 | 0.000113475 | 0.030322254 | 0.891721721 |
| RETREG1-AS1 | -0.699621663 | -1.099425592 | -3.716041766 | 0.000464163 | 0.060219706 | -0.295948687 |
| LOC112268090 | -0.69708887 | 0.128657307 | -3.191644983 | 0.002306007 | 0.131620382 | -1.492829825 |
| LOC105374399 | 0.695493822 | -1.903030896 | 2.700437469 | 0.009109393 | 0.249473825 | -2.671008127 |
| PIK3R6 | 0.692832292 | 4.42474888 | 3.614018194 | 0.000640396 | 0.07183434 | -0.656925321 |
| PTGDR2 | 0.69130388 | 4.362010841 | 2.453120713 | 0.017255967 | 0.315938369 | -3.63991878 |
| RDUR | 0.691064657 | -0.846274181 | 2.316177638 | 0.024180636 | 0.36568898 | -3.336551241 |
| ANKRD18A | 0.69068319 | -1.198352217 | 2.661035897 | 0.010110395 | 0.258209777 | -2.698586421 |
| CACNA1D | 0.690435769 | 0.309307135 | 3.568284342 | 0.000738683 | 0.076146982 | -0.5509227 |
| FPR3 | 0.688177243 | 0.961901479 | 2.593493452 | 0.01206276 | 0.275403325 | -2.887436056 |
| DNAH6 | -0.687138224 | 0.709349666 | -3.465098851 | 0.001015879 | 0.08846016 | -0.801066182 |
| LOC101927401 | -0.686931043 | 0.780628703 | -4.607149813 | 2.35E-05 | 0.011756596 | 2.481500079 |
| CDKN1C | 0.68632374 | -0.912733897 | 2.172943352 | 0.033970876 | 0.421215941 | -3.582325051 |
| OBSCN | -0.68527035 | 5.095250145 | -5.445200085 | 1.15E-06 | 0.002177216 | 5.273372773 |
| SERPINE2 | -0.684906691 | 0.83079441 | -3.237017459 | 0.002017929 | 0.123021328 | -1.394432404 |
| PPBP | 0.683788327 | 6.150584175 | 2.412951482 | 0.019074618 | 0.330723927 | -3.831342394 |
| BLACAT1 | 0.682744092 | -1.775990698 | 2.368013832 | 0.021311453 | 0.346089641 | -3.249987835 |
| GREM2 | 0.682461111 | -1.026905658 | 2.368790179 | 0.021270898 | 0.345885957 | -3.239809182 |
| CDT1 | 0.681729024 | 0.630073814 | 3.074185312 | 0.003241461 | 0.154870793 | -1.777861183 |
| SEPT5-GP1BB | 0.680839632 | -0.209865134 | 2.790115185 | 0.007160707 | 0.223890551 | -2.413629368 |
| SMG1P3 | 0.679017719 | -0.173739901 | 2.844085615 | 0.006181151 | 0.20950801 | -2.297141335 |
| STRCP1 | -0.677283478 | -1.61693806 | -2.362876391 | 0.02158157 | 0.348025812 | -3.249751254 |
| LOC124902709 | 0.67596846 | -1.617551148 | 2.390762846 | 0.020151334 | 0.336693555 | -3.206982686 |
| COPG2IT1 | -0.675941966 | -1.29852696 | -2.746205321 | 0.008061109 | 0.235182508 | -2.517486091 |
| RORB | 0.673985912 | -2.028787404 | 2.00796295 | 0.04941232 | 0.464852959 | -3.815858357 |
| ESPNL | -0.673552807 | -1.727995758 | -2.54349443 | 0.013722571 | 0.296202043 | -2.925529492 |
| EPHA2 | -0.67319255 | -0.950360194 | -2.801233421 | 0.006947912 | 0.222855803 | -2.393838787 |
| FAM156B | 0.672654668 | -1.951799796 | 2.149863646 | 0.035838342 | 0.429489821 | -3.604597686 |
| LINC00540 | -0.672651011 | -0.89954756 | -3.059142184 | 0.003384164 | 0.159008957 | -1.831672059 |
| FAM201A | 0.672420244 | -0.562896869 | 2.761645923 | 0.007733252 | 0.229938983 | -2.47681594 |
| SLC14A2 | 0.671648915 | -1.851798382 | 2.499273104 | 0.015360283 | 0.308486683 | -3.028422955 |
| GJA3 | -0.671641492 | -1.225719245 | -2.373046217 | 0.021049795 | 0.344901123 | -3.234669958 |
| BCAT1 | 0.670868075 | 2.783104609 | 3.368324813 | 0.001363498 | 0.099417656 | -1.159662316 |
| LOC105374869 | -0.670622443 | -0.768078158 | -3.224889102 | 0.002091425 | 0.125699786 | -1.443638643 |
| CDKN3 | 0.669970103 | -0.375642964 | 2.643960774 | 0.010574635 | 0.26212655 | -2.718070565 |
| LOC105378005 | -0.669946351 | -1.01075496 | -3.750725073 | 0.00041563 | 0.05655567 | -0.192794395 |
| TRAV39 | -0.669776006 | -0.163455526 | -3.671158824 | 0.000535064 | 0.065474187 | -0.287023582 |
| LOC124908011 | 0.668098811 | -0.39819718 | 2.273567981 | 0.0267914 | 0.383581584 | -3.429338755 |
| PHGDH | -0.663847437 | 1.344249521 | -4.390008335 | 4.99E-05 | 0.017682861 | 1.855561539 |
| DCHS1 | -0.661585248 | 3.335255714 | -4.868411511 | 9.35E-06 | 0.007229835 | 3.363308828 |
| ANP32AP1 | -0.661503847 | -0.617142569 | -2.63001763 | 0.010968067 | 0.265699504 | -2.748482713 |
| DMD | 0.660923858 | -1.386606061 | 2.576211846 | 0.0126146 | 0.282318065 | -2.866918745 |
| H2BC7 | -0.660764866 | -1.152599051 | -2.873092335 | 0.005707355 | 0.202664718 | -2.25067682 |
| LINC02615 | -0.659689907 | -0.601411394 | -3.42798059 | 0.001137875 | 0.09056146 | -0.942746024 |
| ARHGEF37 | 0.657641567 | -0.825565247 | 2.051902383 | 0.044799549 | 0.454997613 | -3.785583077 |
| STYK1 | 0.655628473 | 0.774160291 | 3.877110285 | 0.000276747 | 0.045261362 | 0.308588533 |
| TRDV1 | -0.652699504 | 1.183705409 | -2.891708599 | 0.005421231 | 0.198727847 | -2.263578949 |
| RNVU1-14 | -0.650895055 | -1.425807103 | -2.690570328 | 0.009351169 | 0.250665532 | -2.633988436 |
| CYP4F12 | 0.648664949 | 2.61377769 | 2.671363418 | 0.009838727 | 0.255102003 | -2.892444044 |
| BIRC5 | 0.647656004 | 0.630553783 | 2.364729968 | 0.021483762 | 0.347685351 | -3.330854919 |
| LOC101927344 | 0.645255612 | -0.212849556 | 2.942380577 | 0.004708378 | 0.187528878 | -2.081445468 |
| CD24 | 0.644982394 | 5.328877175 | 3.625523594 | 0.000617711 | 0.071387024 | -0.690513948 |
| ADGRE4P | 0.64459319 | 4.916872959 | 2.143541383 | 0.036365333 | 0.429993066 | -4.349584478 |
| LIF | 0.639791793 | -1.835898365 | 2.188396191 | 0.032768765 | 0.417438144 | -3.543683021 |
| PKP2 | 0.639666757 | 0.681322456 | 3.160176881 | 0.002528049 | 0.137378871 | -1.570999057 |
| NT5E | -0.639473197 | 3.897755234 | -3.919297587 | 0.000241266 | 0.042746817 | 0.265243486 |
| LINC00997 | 0.639214694 | 0.536676531 | 2.199948081 | 0.0318947 | 0.413403896 | -3.637083236 |
| LINC02021 | -0.638737171 | -1.312635854 | -2.759039549 | 0.007787719 | 0.229967563 | -2.492676825 |
| HYAL3 | 0.636627303 | 1.413156699 | 4.495563333 | 3.47E-05 | 0.014331674 | 2.16072533 |
| ADAM12 | -0.634998686 | 1.1868627 | -3.079074348 | 0.003196307 | 0.153143613 | -1.8095056 |
| ZNF667-AS1 | -0.633275231 | 1.259093291 | -3.932830742 | 0.000230843 | 0.041770311 | 0.491172688 |
| SEMA5A | 0.632837604 | -0.973740359 | 2.957804943 | 0.00450932 | 0.183052568 | -2.083371523 |
| LOC105375024 | -0.631621139 | -1.612602767 | -2.50563805 | 0.015114159 | 0.308486683 | -2.992437513 |
| NUP160 | -0.631377482 | 1.586885605 | -3.277136158 | 0.001791777 | 0.115107848 | -1.332125022 |
| SPINK2 | -0.630289547 | -0.703592125 | -2.473909727 | 0.016377412 | 0.311466269 | -3.057915458 |
| LOC107986075 | -0.628290962 | -1.755298274 | -2.64184034 | 0.010633625 | 0.26212655 | -2.742845421 |
| ASPM | 0.627578939 | 0.814331065 | 2.321032441 | 0.023898026 | 0.363694293 | -3.43492402 |
| GINS2 | 0.626954869 | 0.391594769 | 2.845797085 | 0.006152216 | 0.20950801 | -2.301604268 |
| WFIKKN1 | 0.626799965 | -0.624739799 | 2.031827147 | 0.046858675 | 0.458342334 | -3.831570212 |
| ALDH7A1 | -0.623739411 | -0.578989435 | -2.764841225 | 0.007666955 | 0.229938983 | -2.467630909 |
| KIF20A | 0.623594508 | -0.830268028 | 2.204874622 | 0.03152822 | 0.411430569 | -3.532130668 |
| FDPSP2 | 0.623521741 | -1.539112031 | 2.718592583 | 0.008679445 | 0.244677002 | -2.607059419 |
| TRABD2A | -0.623479127 | 5.166049652 | -5.03365971 | 5.17E-06 | 0.004885831 | 3.833698505 |
| LOC100132249 | -0.621760409 | 0.57857245 | -2.82605325 | 0.006493749 | 0.214723553 | -2.373302554 |
| LOC105369382 | 0.620280453 | -0.454596528 | 2.028592204 | 0.04719802 | 0.459000071 | -3.849285002 |
| TXNRD3 | -0.619015706 | -0.339179802 | -3.661717031 | 0.000551245 | 0.066029071 | -0.32931507 |
| LOC124900465 | 0.618981744 | -1.345293947 | 2.800028295 | 0.006970692 | 0.222855803 | -2.43398335 |
| LOC107985876 | -0.618694331 | 2.1209199 | -3.886233548 | 0.000268672 | 0.045245974 | 0.329507915 |
| NUAK1 | 0.617991768 | -0.056021477 | 2.042923847 | 0.045710596 | 0.454997613 | -3.86022548 |
| SLC35G2 | 0.61603946 | 0.328563382 | 2.879526232 | 0.005606924 | 0.200281409 | -2.224504565 |
| LOC107986759 | -0.615285323 | -1.886373942 | -2.654451276 | 0.010287166 | 0.260573156 | -2.725905534 |
| LOC124902793 | 0.613410886 | -2.151648796 | 2.213479586 | 0.030896995 | 0.408970415 | -3.509195635 |
| LOC124907727 | 0.612662949 | -0.767905579 | 2.104998506 | 0.039727008 | 0.441356423 | -3.704487565 |
| SLC28A3 | 0.61110058 | -0.656531446 | 2.007015877 | 0.04951609 | 0.465057524 | -3.869811793 |
| LOC124902899 | 0.6110784 | -2.003119432 | 2.052500958 | 0.044739375 | 0.454997613 | -3.751188133 |
| TMIGD2 | -0.609698491 | 3.241426203 | -4.685032151 | 1.79E-05 | 0.009823645 | 2.759595202 |
| KLHL29 | -0.609419334 | -0.227621023 | -2.993756444 | 0.004075336 | 0.171577681 | -1.961771233 |
| GRAPL | -0.608495692 | 0.604041945 | -2.324299035 | 0.023709529 | 0.363694293 | -3.444435244 |
| LOC105372879 | -0.607581747 | 0.278576449 | -4.713008866 | 1.62E-05 | 0.009267503 | 2.72711301 |
| LOC283194 | 0.606763717 | -1.244082957 | 2.865389593 | 0.005829781 | 0.204451033 | -2.295580755 |
| LOC107985242 | -0.606136323 | -1.725079998 | -2.485090256 | 0.015921785 | 0.309217041 | -3.032830745 |
| EDA | 0.605042877 | 0.498268505 | 2.717615164 | 0.00870211 | 0.244677002 | -2.590573625 |
| H2AC13 | -0.604968717 | -1.969982829 | -2.056808302 | 0.044308421 | 0.454548814 | -3.749580064 |
| CLU | 0.602972342 | 5.274527362 | 2.192487266 | 0.032456838 | 0.416581922 | -4.276812191 |
| RNASE2 | 0.602503596 | 4.536998325 | 2.02875399 | 0.047180999 | 0.459000071 | -4.531594988 |
| PPP1R3G | 0.602045151 | -1.337343423 | 2.129515972 | 0.037558675 | 0.432491107 | -3.642023853 |
| EPPK1 | -0.601955493 | 1.178269909 | -3.273438341 | 0.001811577 | 0.115107848 | -1.316218214 |
| SOAT2 | -0.600293123 | -0.30277543 | -2.89876471 | 0.005316299 | 0.195358485 | -2.176803317 |
| CCR7 | -0.598716811 | 7.286999851 | -4.277069077 | 7.34E-05 | 0.023104205 | 1.337175144 |
| UPK3BL2 | -0.597598438 | -0.978422571 | -2.12072906 | 0.03832361 | 0.437451295 | -3.685934412 |
| TROAP | 0.59630284 | -0.927141044 | 2.061361781 | 0.043856756 | 0.453006502 | -3.767045518 |
| LOC105375754 | -0.596292906 | 2.517513232 | -2.662267361 | 0.010077645 | 0.257760396 | -2.954188787 |
| MTUS1 | -0.596242673 | 1.828091304 | -2.868819354 | 0.005774972 | 0.203107172 | -2.380377314 |
| HESX1 | -0.596151935 | -1.707607438 | -2.017905086 | 0.048334223 | 0.462435745 | -3.817595566 |
| SCART1 | -0.59475297 | 2.484377209 | -2.87441687 | 0.005686544 | 0.202348183 | -2.445930452 |
| LOC124902052 | -0.594401472 | 0.1510615 | -4.377950623 | 5.20E-05 | 0.017956645 | 1.710319584 |
| FRRS1 | 0.594172158 | 2.9861973 | 3.623749437 | 0.000621158 | 0.071387024 | -0.472392646 |
| RBFOX3 | -0.593198933 | -1.714371074 | -2.812923009 | 0.006730481 | 0.218470884 | -2.408585217 |
| HBA1 | 0.59307005 | 9.007572008 | 2.035165103 | 0.046510734 | 0.457560939 | -4.481983436 |
| AK5 | -0.59286334 | 2.718869602 | -3.772296162 | 0.000387941 | 0.055146448 | -0.051725439 |
| BACE2 | 0.59224772 | 3.283800235 | 3.716044369 | 0.000464159 | 0.060219706 | -0.236136061 |
| EREG | 0.592050457 | -0.739218802 | 2.557204527 | 0.013247958 | 0.289653732 | -2.889201525 |
| COL6A4P2 | -0.589185631 | -0.804146779 | -2.691155758 | 0.009336662 | 0.250665532 | -2.621253437 |
| TRAV1-2 | -0.588611721 | 2.112135156 | -2.905700162 | 0.005215 | 0.19367233 | -2.322536916 |
| GNAI1 | -0.58453695 | 0.042960667 | -3.712202604 | 0.000469857 | 0.060219706 | -0.164018914 |
| CGN | -0.584465745 | -1.307309207 | -3.168163329 | 0.002469869 | 0.135796569 | -1.622544284 |
| XKRX | -0.584227946 | 0.498217515 | -3.445484268 | 0.001078708 | 0.09056146 | -0.851549009 |
| DEPDC1B | 0.579635895 | 0.070980671 | 2.591585935 | 0.012122574 | 0.276397935 | -2.837371019 |
| NECTIN2 | 0.577846937 | 2.058869177 | 2.564493465 | 0.013001742 | 0.287951342 | -3.06201638 |
| MELK | 0.577352076 | -0.014642873 | 2.476107117 | 0.016286947 | 0.310565779 | -3.066679015 |
| ZNF718 | 0.577095353 | 2.394308202 | 2.601398954 | 0.011817717 | 0.272869908 | -3.024844634 |
| METTL21EP | 0.575508895 | -1.396542459 | 2.204738997 | 0.031538259 | 0.411430569 | -3.51845381 |
| EVPL | -0.574738357 | -0.966018644 | -2.40353947 | 0.019524874 | 0.332360726 | -3.183186447 |
| MIR641 | -0.573879764 | -2.000809647 | -2.308040741 | 0.024660978 | 0.368592121 | -3.347149263 |
| TOP2A | 0.573525636 | 2.151158484 | 2.243078618 | 0.028809966 | 0.395184444 | -3.747434477 |
| HMGB3 | 0.572805665 | 0.918244077 | 4.051903496 | 0.00015604 | 0.035406146 | 0.812583413 |
| ACOT11 | 0.572775075 | 0.331749827 | 2.108337844 | 0.039425402 | 0.439728956 | -3.785803755 |
| C4BPB | -0.571510699 | -1.932360572 | -2.01952323 | 0.048160689 | 0.462282819 | -3.80735673 |
| MINAR1 | 0.571426029 | 0.721174058 | 2.672406317 | 0.009811668 | 0.255102003 | -2.703210855 |
| LOC105371254 | -0.570539555 | -0.880169769 | -2.483644464 | 0.015980053 | 0.309217041 | -3.03357679 |
| CUBN | -0.570453095 | 2.695515104 | -3.623754515 | 0.000621148 | 0.071387024 | -0.478897069 |
| PCLAF | 0.569605196 | 0.727842793 | 2.278791656 | 0.026458431 | 0.38073727 | -3.509616519 |
| CARMIL1 | -0.569407721 | 2.170005696 | -4.354966626 | 5.63E-05 | 0.018703204 | 1.750841637 |
| SORCS2 | 0.568310328 | -0.227247666 | 3.199805358 | 0.002251495 | 0.129815868 | -1.491904196 |
| HMGA2-AS1 | 0.568285133 | -1.390249539 | 2.395541703 | 0.019914941 | 0.335047703 | -3.193252926 |
| LOC124901410 | 0.568220774 | -1.50455362 | 2.112258331 | 0.039073855 | 0.439728956 | -3.666177543 |
| TMEM272 | -0.567771068 | 4.137389803 | -3.429795971 | 0.001131598 | 0.09056146 | -1.193139045 |
| RDM1P5 | -0.563573462 | -1.380830181 | -2.753828193 | 0.007897685 | 0.232407821 | -2.507062657 |
| ZNF135 | -0.561869438 | 0.679546673 | -2.63918281 | 0.01070798 | 0.263196571 | -2.797162648 |
| LOC124901653 | -0.560851454 | -0.872531199 | -2.178049266 | 0.033569462 | 0.421078893 | -3.593322621 |
| SLC25A39 | 0.560764017 | 8.711509641 | 2.10272361 | 0.039933624 | 0.44168215 | -4.368324052 |
| EFCAB8 | 0.560431949 | 0.366032261 | 2.716079688 | 0.008737824 | 0.244846211 | -2.587129935 |
| LEF1 | -0.560360794 | 6.791758187 | -3.951222377 | 0.000217371 | 0.040725261 | 0.297143632 |
| LOC124902381 | 0.557625979 | -1.582108472 | 2.074308882 | 0.04259426 | 0.449047836 | -3.724025547 |
| DUSP4 | 0.556868712 | 0.482272825 | 3.060209237 | 0.003373851 | 0.158963526 | -1.809528652 |
| CLTRN | -0.556358705 | -1.400587497 | -3.167973015 | 0.00247124 | 0.135796569 | -1.634873089 |
| NEFL | 0.555314732 | 0.856855077 | 2.29546393 | 0.025420121 | 0.372412434 | -3.491645715 |
| THRB | 0.554691801 | -1.030965559 | 2.168457434 | 0.034327017 | 0.421868345 | -3.586764539 |
| EML5 | -0.5539032 | -0.184297823 | -4.013737301 | 0.000177014 | 0.035557323 | 0.627917024 |
| TMCC2 | 0.553900461 | 3.840272433 | 2.13359194 | 0.037208397 | 0.432395455 | -4.231967673 |
| TMEM204 | -0.551348748 | 4.857621089 | -4.021473852 | 0.000172554 | 0.035557323 | 0.510993717 |
| ZNF391 | -0.550479302 | 0.632806268 | -3.281019984 | 0.001771201 | 0.11454888 | -1.275945042 |
| TNR | -0.550444573 | -1.328875175 | -2.308432047 | 0.024637686 | 0.368592121 | -3.347868994 |
| CELA1 | -0.549989593 | -1.258596173 | -2.248237948 | 0.028459214 | 0.393344073 | -3.454879062 |
| LOC112268199 | -0.549500699 | 2.720908201 | -4.373610862 | 5.28E-05 | 0.017956645 | 1.786058928 |
| ATP8A2 | -0.547498701 | -0.216945794 | -3.213732076 | 0.002161253 | 0.127926532 | -1.444242416 |
| HJURP | 0.547472579 | 0.349117949 | 2.112626023 | 0.039041025 | 0.439728956 | -3.779096218 |
| TTC7B | 0.547097145 | 2.634544592 | 2.918488525 | 0.00503289 | 0.192293069 | -2.31030861 |
| LOC124904082 | 0.546416197 | -1.519784621 | 2.453762254 | 0.017228233 | 0.315770487 | -3.092743193 |
| LOC124904794 | 0.546281742 | -1.864257393 | 2.062113477 | 0.04378258 | 0.452979258 | -3.738043489 |
| FAM72B | 0.545835289 | -1.219819826 | 2.75989865 | 0.007769727 | 0.229938983 | -2.503845441 |
| TRPC2 | 0.545614784 | -0.753551397 | 2.304918785 | 0.024847516 | 0.369806736 | -3.359873196 |
| SLC22A16 | 0.54478176 | 0.875371676 | 2.08101357 | 0.041952959 | 0.446252137 | -3.893769339 |
| IGHJ6 | -0.544286695 | -1.394313511 | -2.31331884 | 0.024348441 | 0.366497911 | -3.338280602 |
| LOC100287467 | 0.543630574 | -1.618329748 | 2.486956936 | 0.015846839 | 0.309217041 | -3.0369497 |
| TFPI | 0.54351049 | -1.041832889 | 2.090794807 | 0.041032438 | 0.443819449 | -3.713962273 |
| LOC613038 | 0.542829559 | -0.459574216 | 2.977007791 | 0.004272436 | 0.176812306 | -2.01115062 |
| GPNMB | -0.542750195 | -0.050646628 | -2.488602363 | 0.01578104 | 0.309217041 | -3.05965404 |
| PLEKHH2 | -0.541570971 | -0.303631162 | -2.263355048 | 0.027453141 | 0.386548412 | -3.478140192 |
| ATP6V0E2-AS1 | -0.540934459 | 1.608154901 | -4.221290701 | 8.86E-05 | 0.027313533 | 1.345980615 |
| KNL1 | 0.540765899 | 1.017132264 | 2.534817688 | 0.014030851 | 0.298686799 | -3.021873028 |
| GAL3ST4 | -0.540762388 | 3.008361554 | -4.01208948 | 0.000177978 | 0.035557323 | 0.639528706 |
| CNN3 | -0.540040612 | 0.643686739 | -3.186753806 | 0.002339273 | 0.132188333 | -1.513211863 |
| RASD1 | 0.538631943 | -1.600646745 | 2.023615125 | 0.047724258 | 0.461479199 | -3.801329358 |
| RRM2 | 0.538219733 | 2.775433207 | 2.008050582 | 0.049402728 | 0.464852959 | -4.29284311 |
| RIMKLA | 0.538170619 | -1.361946133 | 2.107742279 | 0.039479046 | 0.439750553 | -3.677005132 |
| NEAT1 | 0.537620115 | 4.41807681 | 2.243099409 | 0.028808545 | 0.395184444 | -4.098342675 |
| MPP2 | -0.537563507 | 0.146453136 | -3.048460348 | 0.003489036 | 0.160355117 | -1.837202177 |
| LOC124901934 | 0.537432819 | -1.302685796 | 2.705817568 | 0.00897999 | 0.247714488 | -2.614766765 |
| ADORA2A | -0.537401827 | -1.65908085 | -2.794131454 | 0.007083155 | 0.223361139 | -2.442679889 |
| ZNF702P | -0.536535625 | 0.818296368 | -2.446127174 | 0.017560921 | 0.317545 | -3.218536974 |
| NCAPH | 0.535970526 | 1.27514699 | 3.265930435 | 0.001852414 | 0.11626461 | -1.324241317 |
| TMEM139 | 0.53531471 | -1.648824531 | 2.585776799 | 0.012306398 | 0.277980772 | -2.859766788 |
| GPRASP1 | -0.534133116 | 4.493701723 | -4.559038929 | 2.78E-05 | 0.013146783 | 2.254939025 |
| HBA2 | 0.533572377 | 12.5448543 | 2.104229528 | 0.039796746 | 0.44155437 | -4.160518579 |
| GFI1B | 0.532620041 | 3.7154994 | 2.678235099 | 0.009661683 | 0.253739111 | -3.041882245 |
| PLLP | -0.532535799 | -0.692357939 | -2.385046305 | 0.02043741 | 0.339141379 | -3.227155717 |
| TMEM254-AS1 | -0.532151629 | 0.17860566 | -2.505374263 | 0.015124289 | 0.308486683 | -3.041314657 |
| CKB | 0.53189158 | 1.689500602 | 2.422932183 | 0.018607355 | 0.326729406 | -3.325691211 |
| LINC02446 | -0.530431659 | 3.676284885 | -2.916286735 | 0.005063818 | 0.192293069 | -2.506795906 |
| ESCO2 | 0.529187797 | -0.088271938 | 2.423016713 | 0.018603442 | 0.326729406 | -3.168109534 |
| ADGRE1 | 0.528240935 | 6.109773165 | 2.496384039 | 0.015473189 | 0.308900791 | -3.647431711 |
| LOC100134368 | -0.527994399 | -0.099558022 | -3.525224055 | 0.00084424 | 0.080672346 | -0.664125709 |
| IGSF22 | -0.527497624 | -1.526063281 | -2.465718817 | 0.016718633 | 0.313179771 | -3.064664908 |
| LOC105373488 | -0.527171533 | -0.523577716 | -2.728348825 | 0.008456165 | 0.242547907 | -2.545280274 |
| RBM26-AS1 | -0.527019802 | -0.350338064 | -2.465048914 | 0.016746822 | 0.313322893 | -3.088707842 |
| PCSK4 | -0.526560737 | -0.361520566 | -3.013951273 | 0.00384895 | 0.168295083 | -1.916567313 |
| CERCAM | 0.525882023 | 1.539023793 | 2.178856799 | 0.033506359 | 0.420908164 | -3.794122339 |
| ZNF578 | -0.524298784 | -0.240565057 | -3.429086153 | 0.001134049 | 0.09056146 | -0.916061301 |
| FKBP1B | 0.523897326 | 1.393504469 | 2.716455707 | 0.008729066 | 0.244846211 | -2.656753034 |
| E2F7 | 0.523255932 | -1.087818276 | 2.109249409 | 0.039343417 | 0.439728956 | -3.683330721 |
| AMIGO1 | -0.522867284 | 4.354719313 | -4.821057897 | 1.11E-05 | 0.007677615 | 3.138731488 |
| SPMIP3 | 0.52274034 | -1.850979986 | 2.030560345 | 0.04699131 | 0.458342334 | -3.785605483 |
| N4BP3 | -0.522146938 | 2.910866326 | -4.183088761 | 0.000100737 | 0.029041205 | 1.175056549 |
| MS4A7 | 0.521347289 | 5.830709816 | 3.043369533 | 0.003540078 | 0.161483008 | -2.318275082 |
| NCAPG | 0.520263728 | 0.763121576 | 2.064042467 | 0.043592727 | 0.451839543 | -3.912537412 |
| CHST13 | 0.520072442 | 2.63049118 | 2.15949941 | 0.035048014 | 0.424595211 | -3.981915929 |
| SH3RF3 | -0.519655987 | 2.608821807 | -2.345377989 | 0.022524702 | 0.356061955 | -3.655604164 |
| LRRC63 | 0.5192065 | -1.107758488 | 2.022902392 | 0.04780003 | 0.461949269 | -3.820973764 |
| LUNAR1 | -0.518232967 | -0.922122123 | -2.214761918 | 0.030803888 | 0.408692141 | -3.525862647 |
| SEMA3G | 0.517533405 | -0.214101221 | 2.330837108 | 0.023336224 | 0.361816834 | -3.337031838 |
| CPT1A | 0.516768217 | 6.198138498 | 4.107678215 | 0.000129648 | 0.033156373 | 0.763217236 |
| OXNAD1 | -0.516212334 | 5.987995942 | -4.287901141 | 7.07E-05 | 0.022690484 | 1.339106524 |
| CD8B | -0.515831285 | 6.030968095 | -3.403237506 | 0.001226785 | 0.094847226 | -1.338348873 |
| PARVA | -0.515529687 | -1.246647303 | -2.441023912 | 0.017786501 | 0.319799778 | -3.109162615 |
| SIRPG-AS1 | -0.515467091 | -0.012018036 | -2.735018277 | 0.008306576 | 0.239435784 | -2.544846371 |
| RAB44 | 0.515367454 | 4.626609587 | 2.471054038 | 0.016495656 | 0.312085906 | -3.639457451 |
| SYBU | -0.514202158 | -0.71257412 | -2.365322669 | 0.02145257 | 0.347511206 | -3.263447163 |
| CEP15 | 0.513588821 | 0.521750289 | 3.272232127 | 0.00181808 | 0.115107848 | -1.293993119 |
| APBA2 | -0.513368769 | 0.213923433 | -2.06491628 | 0.04350696 | 0.45131138 | -3.882945832 |
| PIPSL | -0.512894847 | -1.115632143 | -2.858309848 | 0.005944445 | 0.206579288 | -2.280769002 |
| LOC107984341 | -0.512322306 | -0.603641985 | -3.586268597 | 0.000698429 | 0.073330745 | -0.554185727 |
| TBC1D8B | 0.510879525 | -0.02791843 | 3.234535606 | 0.002032768 | 0.123137505 | -1.398372684 |
| BACH2 | -0.510442582 | 1.627327384 | -3.539989638 | 0.000806522 | 0.079756565 | -0.627673208 |
| ERVH48-1 | -0.510380635 | -1.206131766 | -2.521320773 | 0.014522875 | 0.30520665 | -2.958428353 |
| IGKJ3 | -0.510070772 | -1.124065816 | -2.175985738 | 0.033731187 | 0.421215941 | -3.584316534 |
| CHEK1 | 0.509966717 | 0.776135393 | 2.540417226 | 0.013831193 | 0.297763265 | -2.99061768 |
| LOC339666 | -0.509231765 | -1.156608651 | -2.237597294 | 0.029186788 | 0.398000315 | -3.476700136 |
| LOC105369747 | -0.508851472 | 0.698708469 | -3.768799834 | 0.000392305 | 0.055146448 | 0.020039719 |
| LOC124903670 | -0.506946911 | -1.546255669 | -2.299830607 | 0.025154225 | 0.37119123 | -3.359921445 |
| ELAPOR2 | -0.506287864 | 1.867819044 | -2.522781486 | 0.014468882 | 0.304958135 | -3.17346863 |
| TMEM17 | 0.50603908 | -1.508613785 | 2.010716878 | 0.049111637 | 0.464852959 | -3.823690167 |
| INSC | -0.505698984 | -0.278207259 | -2.09062686 | 0.041048094 | 0.443819449 | -3.787963304 |
| LINC02725 | 0.50499836 | -1.790394723 | 2.005173691 | 0.049718475 | 0.465645575 | -3.82483464 |
| LOC105370689 | -0.504843732 | -0.847561225 | -3.011497831 | 0.003875814 | 0.168602845 | -1.937410737 |
| NPTXR | -0.504159512 | 2.9096096 | -3.868765472 | 0.000284336 | 0.046059721 | 0.217578582 |
| ECM1 | 0.504064141 | -0.803805355 | 3.123831022 | 0.002809447 | 0.145610261 | -1.705145049 |
| NUSAP1 | 0.503743969 | 2.536780516 | 3.319436339 | 0.001579342 | 0.109025227 | -1.272750674 |
| TCF7 | -0.502915178 | 8.125793414 | -3.108474194 | 0.002936951 | 0.14733328 | -2.067260588 |
| ZHX1-C8orf76 | 0.502883716 | -1.499720978 | 3.08387308 | 0.00315256 | 0.15190337 | -1.866091561 |
| SPNS3 | 0.502385584 | 3.211763555 | 2.091152874 | 0.040999076 | 0.443819449 | -4.214905993 |
| CENPM | 0.502188409 | 0.073971425 | 2.447509697 | 0.017500254 | 0.317545 | -3.129463184 |
| DBNDD1 | -0.501360895 | 1.76947167 | -2.361203416 | 0.02167019 | 0.348382099 | -3.502693847 |
| FUT2 | -0.500531051 | -1.442494831 | -2.490838317 | 0.015692022 | 0.309217041 | -3.017409152 |

| **Supplementary Table 4: Results of GO Enrichment** | | | | | | | | | | |
| --- | --- | --- | --- | --- | --- | --- | --- | --- | --- | --- |
| ID | ONTOLOGY | ID | Description | GeneRatio | BgRatio | pvalue | p.adjust | qvalue | geneID | Count |
| GO:0042744 | BP | GO:0042744 | hydrogen peroxide catabolic process | 6/233 | 31/18870 | 1.89E-06 | 0.005394925 | 0.004936936 | HBZ/HBG2/HBA1/MPO/HBA2/PXDN | 6 |
| GO:0015670 | BP | GO:0015670 | carbon dioxide transport | 4/233 | 15/18870 | 2.78E-05 | 0.026443641 | 0.024198771 | HBZ/HBG2/HBA1/HBA2 | 4 |
| GO:0015671 | BP | GO:0015671 | oxygen transport | 4/233 | 15/18870 | 2.78E-05 | 0.026443641 | 0.024198771 | HBZ/HBG2/HBA1/HBA2 | 4 |
| GO:0042743 | BP | GO:0042743 | hydrogen peroxide metabolic process | 6/233 | 54/18870 | 5.23E-05 | 0.037330839 | 0.034161726 | HBZ/HBG2/HBA1/MPO/HBA2/PXDN | 6 |
| GO:0006935 | BP | GO:0006935 | chemotaxis | 17/233 | 468/18870 | 7.59E-05 | 0.038059938 | 0.03482893 | CTSG/CCL23/AZU1/RNASE3/IL34/PPBP/RNASE2/PTGDR2/CCR8/CYP7B1/SEMA5A/FPR3/SUCNR1/ROBO1/CCR7/LEF1/MTUS1 | 17 |
| GO:0042330 | BP | GO:0042330 | taxis | 17/233 | 470/18870 | 8.00E-05 | 0.038059938 | 0.03482893 | CTSG/CCL23/AZU1/RNASE3/IL34/PPBP/RNASE2/PTGDR2/CCR8/CYP7B1/SEMA5A/FPR3/SUCNR1/ROBO1/CCR7/LEF1/MTUS1 | 17 |
| GO:0005766 | CC | GO:0005766 | primary lysosome | 11/235 | 155/19886 | 2.41E-06 | 0.000351102 | 0.00031624 | CTSG/AZU1/CEACAM8/RNASE3/CEACAM6/DEFA3/RNASE2/ABCA13/BPI/MPO/RAB44 | 11 |
| GO:0042582 | CC | GO:0042582 | azurophil granule | 11/235 | 155/19886 | 2.41E-06 | 0.000351102 | 0.00031624 | CTSG/AZU1/CEACAM8/RNASE3/CEACAM6/DEFA3/RNASE2/ABCA13/BPI/MPO/RAB44 | 11 |
| GO:0031838 | CC | GO:0031838 | haptoglobin-hemoglobin complex | 4/235 | 11/19886 | 5.88E-06 | 0.000570158 | 0.000513545 | HBZ/HBG2/HBA1/HBA2 | 4 |
| GO:0005833 | CC | GO:0005833 | hemoglobin complex | 4/235 | 12/19886 | 8.74E-06 | 0.000635481 | 0.000572381 | HBZ/HBG2/HBA1/HBA2 | 4 |
| GO:0034774 | CC | GO:0034774 | secretory granule lumen | 14/235 | 322/19886 | 3.14E-05 | 0.001306275 | 0.001176569 | CTSG/LTF/AZU1/OLFM4/RNASE3/MMP8/CRISP3/DEFA3/LCN2/CLU/PPBP/RNASE2/BPI/MPO | 14 |
| GO:0000793 | CC | GO:0000793 | condensed chromosome | 13/235 | 281/19886 | 3.18E-05 | 0.001306275 | 0.001176569 | BIRC5/TOP2A/TTK/CDT1/CENPU/HJURP/NCAPG/CHEK1/NCAPH/KNL1/CENPM/RNF212/NUP160 | 13 |
| GO:0060205 | CC | GO:0060205 | cytoplasmic vesicle lumen | 14/235 | 325/19886 | 3.47E-05 | 0.001306275 | 0.001176569 | CTSG/LTF/AZU1/OLFM4/RNASE3/MMP8/CRISP3/DEFA3/LCN2/CLU/PPBP/RNASE2/BPI/MPO | 14 |
| GO:0031983 | CC | GO:0031983 | vesicle lumen | 14/235 | 326/19886 | 3.59E-05 | 0.001306275 | 0.001176569 | CTSG/LTF/AZU1/OLFM4/RNASE3/MMP8/CRISP3/DEFA3/LCN2/CLU/PPBP/RNASE2/BPI/MPO | 14 |
| GO:0035580 | CC | GO:0035580 | specific granule lumen | 6/235 | 62/19886 | 9.04E-05 | 0.002921991 | 0.002631853 | LTF/OLFM4/MMP8/CRISP3/LCN2/BPI | 6 |
| GO:0035578 | CC | GO:0035578 | azurophil granule lumen | 7/235 | 91/19886 | 0.000102578 | 0.002985019 | 0.002688622 | CTSG/AZU1/RNASE3/DEFA3/RNASE2/BPI/MPO | 7 |
| GO:0042581 | CC | GO:0042581 | specific granule | 9/235 | 160/19886 | 0.000123854 | 0.003276512 | 0.002951172 | LTF/OLFM4/CEACAM8/MMP8/CRISP3/LCN2/OLR1/BPI/RAB44 | 9 |
| GO:0071682 | CC | GO:0071682 | endocytic vesicle lumen | 4/235 | 23/19886 | 0.000141074 | 0.003343342 | 0.003011366 | LTF/HBA1/MPO/HBA2 | 4 |
| GO:0070820 | CC | GO:0070820 | tertiary granule | 9/235 | 164/19886 | 0.000149359 | 0.003343342 | 0.003011366 | LTF/OLFM4/CEACAM8/MMP8/CRISP3/OLR1/SERPINB10/PPBP/DSC1 | 9 |
| GO:0005775 | CC | GO:0005775 | vacuolar lumen | 9/235 | 176/19886 | 0.000253524 | 0.005098389 | 0.004592147 | CTSG/AZU1/RNASE3/DEFA3/RNASE2/BPI/MPO/HSPG2/CUBN | 9 |
| GO:0000775 | CC | GO:0000775 | chromosome, centromeric region | 11/235 | 258/19886 | 0.000262804 | 0.005098389 | 0.004592147 | BIRC5/TOP2A/TTK/CDT1/CENPU/HJURP/NCAPG/ESCO2/KNL1/CENPM/NUP160 | 11 |
| GO:0000779 | CC | GO:0000779 | condensed chromosome, centromeric region | 9/235 | 182/19886 | 0.000324801 | 0.005670871 | 0.005107784 | BIRC5/TTK/CDT1/CENPU/HJURP/NCAPG/KNL1/CENPM/NUP160 | 9 |
| GO:1990454 | CC | GO:1990454 | L-type voltage-gated calcium channel complex | 3/235 | 12/19886 | 0.000331288 | 0.005670871 | 0.005107784 | CACNG6/CACNG8/CACNA1D | 3 |
| GO:1904724 | CC | GO:1904724 | tertiary granule lumen | 5/235 | 55/19886 | 0.000474846 | 0.007676681 | 0.006914428 | LTF/OLFM4/MMP8/CRISP3/PPBP | 5 |
| GO:0035577 | CC | GO:0035577 | azurophil granule membrane | 5/235 | 58/19886 | 0.000607764 | 0.009308384 | 0.008384111 | AZU1/CEACAM8/CEACAM6/ABCA13/RAB44 | 5 |
| GO:0000776 | CC | GO:0000776 | kinetochore | 8/235 | 171/19886 | 0.000995827 | 0.014489283 | 0.013050575 | BIRC5/TTK/CDT1/CENPU/HJURP/KNL1/CENPM/NUP160 | 8 |
| GO:0034704 | CC | GO:0034704 | calcium channel complex | 5/235 | 79/19886 | 0.002447074 | 0.033909455 | 0.030542428 | RYR2/CACNG6/CACNG8/CACNA1D/CACHD1 | 5 |
| GO:0098687 | CC | GO:0098687 | chromosomal region | 12/235 | 399/19886 | 0.00292888 | 0.038741101 | 0.034894317 | BIRC5/TOP2A/TTK/CDT1/CENPU/HJURP/NCAPG/CHEK1/ESCO2/KNL1/CENPM/NUP160 | 12 |
| GO:1901681 | MF | GO:1901681 | sulfur compound binding | 15/229 | 275/18496 | 1.83E-06 | 0.000619928 | 0.000554978 | CTSG/CCL23/LTF/AZU1/FBN1/RYR2/RRM2/LIPH/MPO/SEMA5A/GREM2/REG4/SERPINE2/NELL2/GAL3ST4 | 15 |
| GO:0046906 | MF | GO:0046906 | tetrapyrrole binding | 11/229 | 151/18496 | 2.90E-06 | 0.000619928 | 0.000554978 | HBZ/HBG2/IDO1/HBA1/MPO/HBA2/CYP7B1/CYP4F12/PXDN/CYP2J2/CUBN | 11 |
| GO:0031720 | MF | GO:0031720 | haptoglobin binding | 4/229 | 10/18496 | 4.53E-06 | 0.000646861 | 0.000579088 | HBZ/HBG2/HBA1/HBA2 | 4 |
| GO:0020037 | MF | GO:0020037 | heme binding | 10/229 | 141/18496 | 1.04E-05 | 0.000967374 | 0.000866021 | HBZ/HBG2/IDO1/HBA1/MPO/HBA2/CYP7B1/CYP4F12/PXDN/CYP2J2 | 10 |
| GO:0008201 | MF | GO:0008201 | heparin binding | 11/229 | 174/18496 | 1.13E-05 | 0.000967374 | 0.000866021 | CTSG/CCL23/LTF/AZU1/FBN1/LIPH/MPO/GREM2/REG4/SERPINE2/NELL2 | 11 |
| GO:0005344 | MF | GO:0005344 | oxygen carrier activity | 4/229 | 14/18496 | 2.08E-05 | 0.00148269 | 0.001327347 | HBZ/HBG2/HBA1/HBA2 | 4 |
| GO:0005539 | MF | GO:0005539 | glycosaminoglycan binding | 12/229 | 240/18496 | 4.70E-05 | 0.002873885 | 0.002572784 | CTSG/CCL23/LTF/AZU1/FBN1/LIPH/MPO/SEMA5A/GREM2/REG4/SERPINE2/NELL2 | 12 |
| GO:0004601 | MF | GO:0004601 | peroxidase activity | 6/229 | 55/18496 | 5.89E-05 | 0.003152336 | 0.002822062 | HBZ/HBG2/HBA1/MPO/HBA2/PXDN | 6 |
| GO:0016684 | MF | GO:0016684 | oxidoreductase activity, acting on peroxide as acceptor | 6/229 | 57/18496 | 7.23E-05 | 0.003207358 | 0.002871319 | HBZ/HBG2/HBA1/MPO/HBA2/PXDN | 6 |
| GO:0016209 | MF | GO:0016209 | antioxidant activity | 7/229 | 83/18496 | 7.60E-05 | 0.003207358 | 0.002871319 | HBZ/HBG2/HBA1/MPO/HBA2/PXDN/TXNRD3 | 7 |
| GO:0019955 | MF | GO:0019955 | cytokine binding | 9/229 | 145/18496 | 8.24E-05 | 0.003207358 | 0.002871319 | MMP8/IL5RA/ACKR1/WFIKKN1/CCR8/GREM2/PXDN/NOG/CCR7 | 9 |
| GO:0005506 | MF | GO:0005506 | iron ion binding | 9/229 | 153/18496 | 0.00012441 | 0.004437274 | 0.003972375 | LTF/HBZ/LCN2/RRM2/HBA1/HBA2/CYP7B1/CYP4F12/CYP2J2 | 9 |
| GO:0008301 | MF | GO:0008301 | DNA binding, bending | 3/229 | 18/18496 | 0.001332244 | 0.04096948 | 0.036677056 | TOP2A/HMGB3/LEF1 | 3 |
| GO:0019825 | MF | GO:0019825 | oxygen binding | 4/229 | 39/18496 | 0.001340123 | 0.04096948 | 0.036677056 | HBZ/HBG2/HBA1/HBA2 | 4 |
| GO:0030506 | MF | GO:0030506 | ankyrin binding | 3/229 | 19/18496 | 0.001567666 | 0.044730743 | 0.040044246 | CACNA1D/NRCAM/OBSCN | 3 |

| **Supplementary Table 5: Results of KEGG Enrichment** | | | | | | | | | | | |
| --- | --- | --- | --- | --- | --- | --- | --- | --- | --- | --- | --- |
| ID | category | subcategory | ID | Description | GeneRatio | BgRatio | pvalue | p.adjust | qvalue | geneID | Count |
| hsa05412 | Human Diseases | Cardiovascular disease | hsa05412 | Arrhythmogenic right ventricular cardiomyopathy | 6/114 | 86/8845 | 0.000817184 | 0.159350971 | 0.154834952 | RYR2/CACNG6/CACNG8/CACNA1D/PKP2/LEF1 | 6 |
| hsa04061 | Environmental Information Processing | Signaling molecules and interaction | hsa04061 | Viral protein interaction with cytokine and cytokine receptor | 5/114 | 100/8845 | 0.009290215 | 0.451504688 | 0.438709009 | CCL23/IL34/PPBP/CCR8/CCR7 | 5 |
| hsa05143 | Human Diseases | Infectious disease: parasitic | hsa05143 | African trypanosomiasis | 3/114 | 37/8845 | 0.01178391 | 0.451504688 | 0.438709009 | IDO1/HBA1/HBA2 | 3 |
| hsa04814 | Cellular Processes | Cell motility | hsa04814 | Motor proteins | 7/114 | 197/8845 | 0.013558204 | 0.451504688 | 0.438709009 | TNNT1/DYNC1I2/KIF26A/KIF20A/KIF18B/TNNI2/DNAH6 | 7 |
| hsa04261 | Organismal Systems | Circulatory system | hsa04261 | Adrenergic signaling in cardiomyocytes | 6/114 | 154/8845 | 0.014442574 | 0.451504688 | 0.438709009 | RYR2/CACNG6/CACNG8/CACNA1D/PIK3R6/GNAI1 | 6 |
| hsa04921 | Organismal Systems | Endocrine system | hsa04921 | Oxytocin signaling pathway | 6/114 | 154/8845 | 0.014442574 | 0.451504688 | 0.438709009 | RYR2/CACNG6/CACNG8/CACNA1D/PIK3R6/GNAI1 | 6 |
| hsa04110 | Cellular Processes | Cell growth and death | hsa04110 | Cell cycle | 6/114 | 158/8845 | 0.016207861 | 0.451504688 | 0.438709009 | TICRR/TTK/CDT1/CHEK1/ESCO2/KNL1 | 6 |
| hsa04260 | Organismal Systems | Circulatory system | hsa04260 | Cardiac muscle contraction | 4/114 | 87/8845 | 0.025716928 | 0.520292085 | 0.505546966 | RYR2/CACNG6/CACNG8/CACNA1D | 4 |
| hsa05144 | Human Diseases | Infectious disease: parasitic | hsa05144 | Malaria | 3/114 | 50/8845 | 0.026360289 | 0.520292085 | 0.505546966 | ACKR1/HBA1/HBA2 | 3 |
| hsa04610 | Organismal Systems | Immune system | hsa04610 | Complement and coagulation cascades | 4/114 | 88/8845 | 0.026681645 | 0.520292085 | 0.505546966 | CLU/TFPI/SERPINE2/C4BPB | 4 |
| hsa04820 | NA | NA | hsa04820 | Cytoskeleton in muscle cells | 7/114 | 232/8845 | 0.030097199 | 0.533541261 | 0.518420659 | TNNT1/FBN1/PKP2/FBLN2/TNNI2/HSPG2/OBSCN | 7 |
| hsa04062 | Organismal Systems | Immune system | hsa04062 | Chemokine signaling pathway | 6/114 | 193/8845 | 0.038319788 | 0.539408796 | 0.524121907 | CCL23/PPBP/CCR8/PIK3R6/CCR7/GNAI1 | 6 |
| hsa04060 | Environmental Information Processing | Signaling molecules and interaction | hsa04060 | Cytokine-cytokine receptor interaction | 8/114 | 298/8845 | 0.038357234 | 0.539408796 | 0.524121907 | CCL23/IL34/IL5RA/PPBP/CCR8/LIF/EDAR/CCR7 | 8 |
| hsa05410 | Human Diseases | Cardiovascular disease | hsa05410 | Hypertrophic cardiomyopathy | 4/114 | 99/8845 | 0.038726785 | 0.539408796 | 0.524121907 | RYR2/CACNG6/CACNG8/CACNA1D | 4 |
| hsa05414 | Human Diseases | Cardiovascular disease | hsa05414 | Dilated cardiomyopathy | 4/114 | 105/8845 | 0.046416366 | 0.603412757 | 0.586311991 | RYR2/CACNG6/CACNG8/CACNA1D | 4 |
| hsa00290 | Metabolism | Amino acid metabolism | hsa00290 | Valine, leucine and isoleucine biosynthesis | 1/114 | 4/8845 | 0.050574797 | 0.614907962 | 0.59748142 | BCAT1 | 1 |
| hsa05145 | Human Diseases | Infectious disease: parasitic | hsa05145 | Toxoplasmosis | 4/114 | 111/8845 | 0.054897224 | 0.614907962 | 0.59748142 | LAMB2/PIK3R6/HLA-DRA/GNAI1 | 4 |
| hsa05221 | Human Diseases | Cancer: specific types | hsa05221 | Acute myeloid leukemia | 3/114 | 68/8845 | 0.057166811 | 0.614907962 | 0.59748142 | CEBPE/MPO/LEF1 | 3 |
| hsa05310 | Human Diseases | Immune disease | hsa05310 | Asthma | 2/114 | 31/8845 | 0.060089111 | 0.614907962 | 0.59748142 | RNASE3/HLA-DRA | 2 |
| hsa04022 | Environmental Information Processing | Signal transduction | hsa04022 | cGMP-PKG signaling pathway | 5/114 | 166/8845 | 0.063067483 | 0.614907962 | 0.59748142 | TRPC6/ADORA3/CACNA1D/PIK3R6/GNAI1 | 5 |
| hsa04710 | Organismal Systems | Environmental adaptation | hsa04710 | Circadian rhythm | 2/114 | 34/8845 | 0.070728515 | 0.656764785 | 0.638152018 | RORB/NPAS2 | 2 |
| hsa04360 | Organismal Systems | Development and regeneration | hsa04360 | Axon guidance | 5/114 | 184/8845 | 0.088907918 | 0.788047453 | 0.765714124 | TRPC6/UNC5B/SEMA5A/ROBO1/GNAI1 | 5 |
| hsa01232 | Metabolism | Global and overview maps | hsa01232 | Nucleotide metabolism | 3/114 | 85/8845 | 0.096612657 | 0.81910731 | 0.795893742 | RRM2/NT5E/AK5 | 3 |
| hsa05210 | Human Diseases | Cancer: specific types | hsa05210 | Colorectal cancer | 3/114 | 87/8845 | 0.101833974 | 0.826809427 | 0.803377581 | BIRC5/EREG/LEF1 | 3 |
| hsa04512 | Environmental Information Processing | Signaling molecules and interaction | hsa04512 | ECM-receptor interaction | 3/114 | 89/8845 | 0.107166126 | 0.826809427 | 0.803377581 | LAMB2/HSPG2/TNR | 3 |
| hsa03266 | Genetic Information Processing | Information processing in viruses | hsa03266 | Virion - Herpesvirus | 1/114 | 9/8845 | 0.110241257 | 0.826809427 | 0.803377581 | NECTIN2 | 1 |
| hsa04713 | Organismal Systems | Environmental adaptation | hsa04713 | Circadian entrainment | 3/114 | 97/8845 | 0.129533868 | 0.870421717 | 0.845753895 | RYR2/CACNA1D/GNAI1 | 3 |
| hsa04640 | Organismal Systems | Immune system | hsa04640 | Hematopoietic cell lineage | 3/114 | 99/8845 | 0.135368019 | 0.870421717 | 0.845753895 | IL5RA/CD24/HLA-DRA | 3 |
| hsa05150 | Human Diseases | Infectious disease: bacterial | hsa05150 | Staphylococcus aureus infection | 3/114 | 100/8845 | 0.13831899 | 0.870421717 | 0.845753895 | DEFA3/FPR3/HLA-DRA | 3 |
| hsa04145 | Cellular Processes | Transport and catabolism | hsa04145 | Phagosome | 4/114 | 157/8845 | 0.144352675 | 0.870421717 | 0.845753895 | OLR1/DYNC1I2/MPO/HLA-DRA | 4 |
| hsa04514 | Environmental Information Processing | Signaling molecules and interaction | hsa04514 | Cell adhesion molecules | 4/114 | 157/8845 | 0.144352675 | 0.870421717 | 0.845753895 | NECTIN2/NRXN2/NRCAM/HLA-DRA | 4 |
| hsa00270 | Metabolism | Amino acid metabolism | hsa00270 | Cysteine and methionine metabolism | 2/114 | 52/8845 | 0.144426257 | 0.870421717 | 0.845753895 | BCAT1/PHGDH | 2 |
| hsa05146 | Human Diseases | Infectious disease: parasitic | hsa05146 | Amoebiasis | 3/114 | 103/8845 | 0.147302137 | 0.870421717 | 0.845753895 | CTSG/SERPINB10/LAMB2 | 3 |
| hsa04350 | Environmental Information Processing | Signal transduction | hsa04350 | TGF-beta signaling pathway | 3/114 | 108/8845 | 0.162681466 | 0.877803626 | 0.8529266 | FBN1/GREM2/NOG | 3 |
| hsa00240 | Metabolism | Nucleotide metabolism | hsa00240 | Pyrimidine metabolism | 2/114 | 58/8845 | 0.171587189 | 0.877803626 | 0.8529266 | RRM2/NT5E | 2 |
| hsa00730 | Metabolism | Metabolism of cofactors and vitamins | hsa00730 | Thiamine metabolism | 1/114 | 15/8845 | 0.176952548 | 0.877803626 | 0.8529266 | AK5 | 1 |
| hsa04725 | Organismal Systems | Nervous system | hsa04725 | Cholinergic synapse | 3/114 | 115/8845 | 0.184975199 | 0.877803626 | 0.8529266 | CACNA1D/PIK3R6/GNAI1 | 3 |
| hsa04726 | Organismal Systems | Nervous system | hsa04726 | Serotonergic synapse | 3/114 | 115/8845 | 0.184975199 | 0.877803626 | 0.8529266 | CACNA1D/CYP2J2/GNAI1 | 3 |
| hsa00603 | Metabolism | Glycan biosynthesis and metabolism | hsa00603 | Glycosphingolipid biosynthesis - globo and isoglobo series | 1/114 | 16/8845 | 0.187578529 | 0.877803626 | 0.8529266 | FUT2 | 1 |
| hsa04724 | Organismal Systems | Nervous system | hsa04724 | Glutamatergic synapse | 3/114 | 116/8845 | 0.188225064 | 0.877803626 | 0.8529266 | CACNA1D/SHANK1/GNAI1 | 3 |
| hsa04010 | Environmental Information Processing | Signal transduction | hsa04010 | MAPK signaling pathway | 6/114 | 300/8845 | 0.190241645 | 0.877803626 | 0.8529266 | CACNG6/CACNG8/CACNA1D/EREG/DUSP4/ERBB3 | 6 |
| hsa00120 | Metabolism | Lipid metabolism | hsa00120 | Primary bile acid biosynthesis | 1/114 | 17/8845 | 0.19806851 | 0.877803626 | 0.8529266 | CYP7B1 | 1 |
| hsa00450 | Metabolism | Metabolism of other amino acids | hsa00450 | Selenocompound metabolism | 1/114 | 17/8845 | 0.19806851 | 0.877803626 | 0.8529266 | TXNRD3 | 1 |
| hsa00910 | Metabolism | Energy metabolism | hsa00910 | Nitrogen metabolism | 1/114 | 17/8845 | 0.19806851 | 0.877803626 | 0.8529266 | CA6 | 1 |
| hsa04919 | Organismal Systems | Endocrine system | hsa04919 | Thyroid hormone signaling pathway | 3/114 | 122/8845 | 0.208023605 | 0.883478422 | 0.858440572 | RCAN2/THRB/SLC16A10 | 3 |
| hsa00531 | Metabolism | Glycan biosynthesis and metabolism | hsa00531 | Glycosaminoglycan degradation | 1/114 | 19/8845 | 0.218647356 | 0.883478422 | 0.858440572 | HYAL3 | 1 |
| hsa04924 | Organismal Systems | Endocrine system | hsa04924 | Renin secretion | 2/114 | 69/8845 | 0.223161404 | 0.883478422 | 0.858440572 | CACNA1D/GNAI1 | 2 |
| hsa00230 | Metabolism | Nucleotide metabolism | hsa00230 | Purine metabolism | 3/114 | 128/8845 | 0.228269961 | 0.883478422 | 0.858440572 | RRM2/NT5E/AK5 | 3 |
| hsa04613 | Organismal Systems | Immune system | hsa04613 | Neutrophil extracellular trap formation | 4/114 | 192/8845 | 0.235003541 | 0.883478422 | 0.858440572 | CTSG/AZU1/MPO/FPR3 | 4 |
| hsa00532 | Metabolism | Glycan biosynthesis and metabolism | hsa00532 | Glycosaminoglycan biosynthesis - chondroitin sulfate / dermatan sulfate | 1/114 | 21/8845 | 0.23870263 | 0.883478422 | 0.858440572 | CHST13 | 1 |
| hsa00770 | Metabolism | Metabolism of cofactors and vitamins | hsa00770 | Pantothenate and CoA biosynthesis | 1/114 | 21/8845 | 0.23870263 | 0.883478422 | 0.858440572 | BCAT1 | 1 |
| hsa04142 | Cellular Processes | Transport and catabolism | hsa04142 | Lysosome | 3/114 | 132/8845 | 0.241972799 | 0.883478422 | 0.858440572 | CTSG/CTSE/HYAL3 | 3 |
| hsa01230 | Metabolism | Global and overview maps | hsa01230 | Biosynthesis of amino acids | 2/114 | 75/8845 | 0.251813469 | 0.883478422 | 0.858440572 | BCAT1/PHGDH | 2 |
| hsa01524 | Human Diseases | Drug resistance: antineoplastic | hsa01524 | Platinum drug resistance | 2/114 | 75/8845 | 0.251813469 | 0.883478422 | 0.858440572 | BIRC5/TOP2A | 2 |
| hsa04115 | Cellular Processes | Cell growth and death | hsa04115 | p53 signaling pathway | 2/114 | 75/8845 | 0.251813469 | 0.883478422 | 0.858440572 | RRM2/CHEK1 | 2 |
| hsa03320 | Organismal Systems | Endocrine system | hsa03320 | PPAR signaling pathway | 2/114 | 76/8845 | 0.256603625 | 0.883478422 | 0.858440572 | OLR1/CPT1A | 2 |
| hsa04614 | Organismal Systems | Endocrine system | hsa04614 | Renin-angiotensin system | 1/114 | 23/8845 | 0.258247539 | 0.883478422 | 0.858440572 | CTSG | 1 |
| hsa05133 | Human Diseases | Infectious disease: bacterial | hsa05133 | Pertussis | 2/114 | 78/8845 | 0.266188717 | 0.891575363 | 0.866308045 | C4BPB/GNAI1 | 2 |
| hsa04371 | Environmental Information Processing | Signal transduction | hsa04371 | Apelin signaling pathway | 3/114 | 140/8845 | 0.269758699 | 0.891575363 | 0.866308045 | RYR2/PIK3R6/GNAI1 | 3 |
| hsa00983 | Metabolism | Xenobiotics biodegradation and metabolism | hsa00983 | Drug metabolism - other enzymes | 2/114 | 81/8845 | 0.280566069 | 0.908811168 | 0.883055385 | RRM2/MPO | 2 |
| hsa04977 | Organismal Systems | Digestive system | hsa04977 | Vitamin digestion and absorption | 1/114 | 26/8845 | 0.286636042 | 0.908811168 | 0.883055385 | CUBN | 1 |
| hsa04012 | Environmental Information Processing | Signal transduction | hsa04012 | ErbB signaling pathway | 2/114 | 86/8845 | 0.30447686 | 0.908811168 | 0.883055385 | EREG/ERBB3 | 2 |
| hsa04911 | Organismal Systems | Endocrine system | hsa04911 | Insulin secretion | 2/114 | 86/8845 | 0.30447686 | 0.908811168 | 0.883055385 | RYR2/CACNA1D | 2 |
| hsa00601 | Metabolism | Glycan biosynthesis and metabolism | hsa00601 | Glycosphingolipid biosynthesis - lacto and neolacto series | 1/114 | 28/8845 | 0.304960665 | 0.908811168 | 0.883055385 | FUT2 | 1 |
| hsa04392 | Environmental Information Processing | Signal transduction | hsa04392 | Hippo signaling pathway - multiple species | 1/114 | 29/8845 | 0.313947223 | 0.908811168 | 0.883055385 | DCHS1 | 1 |
| hsa01240 | Metabolism | Global and overview maps | hsa01240 | Biosynthesis of cofactors | 3/114 | 153/8845 | 0.315577178 | 0.908811168 | 0.883055385 | IDO1/BCAT1/AK5 | 3 |
| hsa04727 | Organismal Systems | Nervous system | hsa04727 | GABAergic synapse | 2/114 | 89/8845 | 0.318761711 | 0.908811168 | 0.883055385 | CACNA1D/GNAI1 | 2 |
| hsa04934 | Human Diseases | Endocrine and metabolic disease | hsa04934 | Cushing syndrome | 3/114 | 155/8845 | 0.322660206 | 0.908811168 | 0.883055385 | CACNA1D/GNAI1/LEF1 | 3 |
| hsa00591 | Metabolism | Lipid metabolism | hsa00591 | Linoleic acid metabolism | 1/114 | 30/8845 | 0.322818595 | 0.908811168 | 0.883055385 | CYP2J2 | 1 |
| hsa04148 | Cellular Processes | Transport and catabolism | hsa04148 | Efferocytosis | 3/114 | 157/8845 | 0.329745103 | 0.908811168 | 0.883055385 | CD24/DUSP4/CPT1A | 3 |
| hsa04024 | Environmental Information Processing | Signal transduction | hsa04024 | cAMP signaling pathway | 4/114 | 226/8845 | 0.332810997 | 0.908811168 | 0.883055385 | RYR2/CACNA1D/SUCNR1/GNAI1 | 4 |
| hsa04520 | Cellular Processes | Cellular community - eukaryotes | hsa04520 | Adherens junction | 2/114 | 93/8845 | 0.337700891 | 0.908811168 | 0.883055385 | NECTIN2/LEF1 | 2 |
| hsa04215 | Cellular Processes | Cell growth and death | hsa04215 | Apoptosis - multiple species | 1/114 | 32/8845 | 0.340221617 | 0.908811168 | 0.883055385 | BIRC5 | 1 |
| hsa01210 | Metabolism | Global and overview maps | hsa01210 | 2-Oxocarboxylic acid metabolism | 1/114 | 33/8845 | 0.348756138 | 0.915381699 | 0.889439708 | BCAT1 | 1 |
| hsa04750 | Organismal Systems | Sensory system | hsa04750 | Inflammatory mediator regulation of TRP channels | 2/114 | 99/8845 | 0.365807192 | 0.915381699 | 0.889439708 | ASIC1/CYP2J2 | 2 |
| hsa00512 | Metabolism | Glycan biosynthesis and metabolism | hsa00512 | Mucin type O-glycan biosynthesis | 1/114 | 36/8845 | 0.373708598 | 0.915381699 | 0.889439708 | GALNT16 | 1 |
| hsa04916 | Organismal Systems | Endocrine system | hsa04916 | Melanogenesis | 2/114 | 101/8845 | 0.375079259 | 0.915381699 | 0.889439708 | GNAI1/LEF1 | 2 |
| hsa00250 | Metabolism | Amino acid metabolism | hsa00250 | Alanine, aspartate and glutamate metabolism | 1/114 | 37/8845 | 0.381813629 | 0.915381699 | 0.889439708 | RIMKLA | 1 |
| hsa05216 | Human Diseases | Cancer: specific types | hsa05216 | Thyroid cancer | 1/114 | 37/8845 | 0.381813629 | 0.915381699 | 0.889439708 | LEF1 | 1 |
| hsa00760 | Metabolism | Metabolism of cofactors and vitamins | hsa00760 | Nicotinate and nicotinamide metabolism | 1/114 | 38/8845 | 0.389814679 | 0.915381699 | 0.889439708 | NT5E | 1 |
| hsa05330 | Human Diseases | Immune disease | hsa05330 | Allograft rejection | 1/114 | 38/8845 | 0.389814679 | 0.915381699 | 0.889439708 | HLA-DRA | 1 |
| hsa00260 | Metabolism | Amino acid metabolism | hsa00260 | Glycine, serine and threonine metabolism | 1/114 | 41/8845 | 0.41320708 | 0.915381699 | 0.889439708 | PHGDH | 1 |
| hsa00380 | Metabolism | Amino acid metabolism | hsa00380 | Tryptophan metabolism | 1/114 | 42/8845 | 0.420805262 | 0.915381699 | 0.889439708 | IDO1 | 1 |
| hsa00071 | Metabolism | Lipid metabolism | hsa00071 | Fatty acid degradation | 1/114 | 43/8845 | 0.428305909 | 0.915381699 | 0.889439708 | CPT1A | 1 |
| hsa04940 | Human Diseases | Endocrine and metabolic disease | hsa04940 | Type I diabetes mellitus | 1/114 | 43/8845 | 0.428305909 | 0.915381699 | 0.889439708 | HLA-DRA | 1 |
| hsa04962 | Organismal Systems | Excretory system | hsa04962 | Vasopressin-regulated water reabsorption | 1/114 | 44/8845 | 0.435710264 | 0.915381699 | 0.889439708 | DYNC1I2 | 1 |
| hsa05332 | Human Diseases | Immune disease | hsa05332 | Graft-versus-host disease | 1/114 | 44/8845 | 0.435710264 | 0.915381699 | 0.889439708 | HLA-DRA | 1 |
| hsa02010 | Environmental Information Processing | Membrane transport | hsa02010 | ABC transporters | 1/114 | 45/8845 | 0.44301955 | 0.915381699 | 0.889439708 | ABCA13 | 1 |
| hsa05202 | Human Diseases | Cancer: overview | hsa05202 | Transcriptional misregulation in cancer | 3/114 | 193/8845 | 0.455144736 | 0.915381699 | 0.889439708 | DEFA3/CEBPE/MPO | 3 |
| hsa00514 | Metabolism | Glycan biosynthesis and metabolism | hsa00514 | Other types of O-glycan biosynthesis | 1/114 | 47/8845 | 0.457357744 | 0.915381699 | 0.889439708 | GALNT16 | 1 |
| hsa04930 | Human Diseases | Endocrine and metabolic disease | hsa04930 | Type II diabetes mellitus | 1/114 | 47/8845 | 0.457357744 | 0.915381699 | 0.889439708 | CACNA1D | 1 |
| hsa00280 | Metabolism | Amino acid metabolism | hsa00280 | Valine, leucine and isoleucine degradation | 1/114 | 48/8845 | 0.464389026 | 0.915381699 | 0.889439708 | BCAT1 | 1 |
| hsa05167 | Human Diseases | Infectious disease: viral | hsa05167 | Kaposi sarcoma-associated herpesvirus infection | 3/114 | 196/8845 | 0.465229535 | 0.915381699 | 0.889439708 | CCR8/PIK3R6/LEF1 | 3 |
| hsa04071 | Environmental Information Processing | Signal transduction | hsa04071 | Sphingolipid signaling pathway | 2/114 | 122/8845 | 0.468600634 | 0.915381699 | 0.889439708 | ADORA3/GNAI1 | 2 |
| hsa04935 | Organismal Systems | Endocrine system | hsa04935 | Growth hormone synthesis, secretion and action | 2/114 | 122/8845 | 0.468600634 | 0.915381699 | 0.889439708 | CACNA1D/GNAI1 | 2 |
| hsa04672 | Organismal Systems | Immune system | hsa04672 | Intestinal immune network for IgA production | 1/114 | 49/8845 | 0.471329989 | 0.915381699 | 0.889439708 | HLA-DRA | 1 |
| hsa05030 | Human Diseases | Substance dependence | hsa05030 | Cocaine addiction | 1/114 | 49/8845 | 0.471329989 | 0.915381699 | 0.889439708 | GNAI1 | 1 |
| hsa00330 | Metabolism | Amino acid metabolism | hsa00330 | Arginine and proline metabolism | 1/114 | 50/8845 | 0.478181783 | 0.915381699 | 0.889439708 | CKB | 1 |
| hsa04611 | Organismal Systems | Immune system | hsa04611 | Platelet activation | 2/114 | 125/8845 | 0.481295135 | 0.915381699 | 0.889439708 | PIK3R6/GNAI1 | 2 |
| hsa04913 | Organismal Systems | Endocrine system | hsa04913 | Ovarian steroidogenesis | 1/114 | 51/8845 | 0.484945544 | 0.915381699 | 0.889439708 | CYP2J2 | 1 |
| hsa04973 | Organismal Systems | Digestive system | hsa04973 | Carbohydrate digestion and absorption | 1/114 | 52/8845 | 0.491622392 | 0.915381699 | 0.889439708 | CACNA1D | 1 |
| hsa05320 | Human Diseases | Immune disease | hsa05320 | Autoimmune thyroid disease | 1/114 | 53/8845 | 0.498213436 | 0.915381699 | 0.889439708 | HLA-DRA | 1 |
| hsa04151 | Environmental Information Processing | Signal transduction | hsa04151 | PI3K-Akt signaling pathway | 5/114 | 362/8845 | 0.502384838 | 0.915381699 | 0.889439708 | LAMB2/PIK3R6/EREG/ERBB3/TNR | 5 |
| hsa04728 | Organismal Systems | Nervous system | hsa04728 | Dopaminergic synapse | 2/114 | 132/8845 | 0.510188477 | 0.915381699 | 0.889439708 | CACNA1D/GNAI1 | 2 |
| hsa01212 | Metabolism | Global and overview maps | hsa01212 | Fatty acid metabolism | 1/114 | 57/8845 | 0.523741216 | 0.915381699 | 0.889439708 | CPT1A | 1 |
| hsa05207 | Human Diseases | Cancer: overview | hsa05207 | Chemical carcinogenesis - receptor activation | 3/114 | 215/8845 | 0.527079376 | 0.915381699 | 0.889439708 | BIRC5/CACNA1D/GNAI1 | 3 |
| hsa00480 | Metabolism | Metabolism of other amino acids | hsa00480 | Glutathione metabolism | 1/114 | 58/8845 | 0.529919356 | 0.915381699 | 0.889439708 | RRM2 | 1 |
| hsa04923 | Organismal Systems | Endocrine system | hsa04923 | Regulation of lipolysis in adipocytes | 1/114 | 59/8845 | 0.536018047 | 0.915381699 | 0.889439708 | GNAI1 | 1 |
| hsa05213 | Human Diseases | Cancer: specific types | hsa05213 | Endometrial cancer | 1/114 | 59/8845 | 0.536018047 | 0.915381699 | 0.889439708 | LEF1 | 1 |
| hsa05322 | Human Diseases | Immune disease | hsa05322 | Systemic lupus erythematosus | 2/114 | 139/8845 | 0.538024002 | 0.915381699 | 0.889439708 | CTSG/HLA-DRA | 2 |
| hsa04730 | Organismal Systems | Nervous system | hsa04730 | Long-term depression | 1/114 | 60/8845 | 0.5420383 | 0.915381699 | 0.889439708 | GNAI1 | 1 |
| hsa00590 | Metabolism | Lipid metabolism | hsa00590 | Arachidonic acid metabolism | 1/114 | 61/8845 | 0.547981115 | 0.915381699 | 0.889439708 | CYP2J2 | 1 |
| hsa00140 | Metabolism | Lipid metabolism | hsa00140 | Steroid hormone biosynthesis | 1/114 | 62/8845 | 0.553847481 | 0.915381699 | 0.889439708 | CYP7B1 | 1 |
| hsa04936 | Human Diseases | Endocrine and metabolic disease | hsa04936 | Alcoholic liver disease | 2/114 | 144/8845 | 0.5572387 | 0.915381699 | 0.889439708 | CPT1A/LEF1 | 2 |
| hsa05217 | Human Diseases | Cancer: specific types | hsa05217 | Basal cell carcinoma | 1/114 | 63/8845 | 0.559638371 | 0.915381699 | 0.889439708 | LEF1 | 1 |
| hsa04927 | Organismal Systems | Endocrine system | hsa04927 | Cortisol synthesis and secretion | 1/114 | 65/8845 | 0.570997563 | 0.915381699 | 0.889439708 | CACNA1D | 1 |
| hsa04929 | Organismal Systems | Endocrine system | hsa04929 | GnRH secretion | 1/114 | 65/8845 | 0.570997563 | 0.915381699 | 0.889439708 | CACNA1D | 1 |
| hsa05321 | Human Diseases | Immune disease | hsa05321 | Inflammatory bowel disease | 1/114 | 65/8845 | 0.570997563 | 0.915381699 | 0.889439708 | HLA-DRA | 1 |
| hsa04723 | Organismal Systems | Nervous system | hsa04723 | Retrograde endocannabinoid signaling | 2/114 | 149/8845 | 0.575886914 | 0.915381699 | 0.889439708 | CACNA1D/GNAI1 | 2 |
| hsa05226 | Human Diseases | Cancer: specific types | hsa05226 | Gastric cancer | 2/114 | 150/8845 | 0.579548068 | 0.915381699 | 0.889439708 | REG4/LEF1 | 2 |
| hsa05031 | Human Diseases | Substance dependence | hsa05031 | Amphetamine addiction | 1/114 | 69/8845 | 0.59285179 | 0.915381699 | 0.889439708 | CACNA1D | 1 |
| hsa05416 | Human Diseases | Cardiovascular disease | hsa05416 | Viral myocarditis | 1/114 | 69/8845 | 0.59285179 | 0.915381699 | 0.889439708 | HLA-DRA | 1 |
| hsa04920 | Organismal Systems | Endocrine system | hsa04920 | Adipocytokine signaling pathway | 1/114 | 70/8845 | 0.598140635 | 0.915381699 | 0.889439708 | CPT1A | 1 |
| hsa04218 | Cellular Processes | Cell growth and death | hsa04218 | Cellular senescence | 2/114 | 157/8845 | 0.604533846 | 0.915381699 | 0.889439708 | CACNA1D/CHEK1 | 2 |
| hsa04390 | Environmental Information Processing | Signal transduction | hsa04390 | Hippo signaling pathway | 2/114 | 157/8845 | 0.604533846 | 0.915381699 | 0.889439708 | BIRC5/LEF1 | 2 |
| hsa05161 | Human Diseases | Infectious disease: viral | hsa05161 | Hepatitis B | 2/114 | 163/8845 | 0.625054623 | 0.915381699 | 0.889439708 | BIRC5/HSPG2 | 2 |
| hsa04971 | Organismal Systems | Digestive system | hsa04971 | Gastric acid secretion | 1/114 | 76/8845 | 0.628473498 | 0.915381699 | 0.889439708 | GNAI1 | 1 |
| hsa05140 | Human Diseases | Infectious disease: parasitic | hsa05140 | Leishmaniasis | 1/114 | 77/8845 | 0.633303469 | 0.915381699 | 0.889439708 | HLA-DRA | 1 |
| hsa04020 | Environmental Information Processing | Signal transduction | hsa04020 | Calcium signaling pathway | 3/114 | 254/8845 | 0.640694179 | 0.915381699 | 0.889439708 | RYR2/CACNA1D/ERBB3 | 3 |
| hsa04630 | Environmental Information Processing | Signal transduction | hsa04630 | JAK-STAT signaling pathway | 2/114 | 168/8845 | 0.641525775 | 0.915381699 | 0.889439708 | IL5RA/LIF | 2 |
| hsa01521 | Human Diseases | Drug resistance: antineoplastic | hsa01521 | EGFR tyrosine kinase inhibitor resistance | 1/114 | 80/8845 | 0.647423069 | 0.915381699 | 0.889439708 | ERBB3 | 1 |
| hsa04612 | Organismal Systems | Immune system | hsa04612 | Antigen processing and presentation | 1/114 | 80/8845 | 0.647423069 | 0.915381699 | 0.889439708 | HLA-DRA | 1 |
| hsa04530 | Cellular Processes | Cellular community - eukaryotes | hsa04530 | Tight junction | 2/114 | 170/8845 | 0.647954877 | 0.915381699 | 0.889439708 | CACNA1D/CGN | 2 |
| hsa05225 | Human Diseases | Cancer: specific types | hsa05225 | Hepatocellular carcinoma | 2/114 | 170/8845 | 0.647954877 | 0.915381699 | 0.889439708 | TXNRD3/LEF1 | 2 |
| hsa04310 | Environmental Information Processing | Signal transduction | hsa04310 | Wnt signaling pathway | 2/114 | 174/8845 | 0.660541712 | 0.915381699 | 0.889439708 | SFRP5/LEF1 | 2 |
| hsa05032 | Human Diseases | Substance dependence | hsa05032 | Morphine addiction | 1/114 | 91/8845 | 0.69473553 | 0.915381699 | 0.889439708 | GNAI1 | 1 |
| hsa04540 | Cellular Processes | Cellular community - eukaryotes | hsa04540 | Gap junction | 1/114 | 92/8845 | 0.698710872 | 0.915381699 | 0.889439708 | GNAI1 | 1 |
| hsa04658 | Organismal Systems | Immune system | hsa04658 | Th1 and Th2 cell differentiation | 1/114 | 92/8845 | 0.698710872 | 0.915381699 | 0.889439708 | HLA-DRA | 1 |
| hsa05034 | Human Diseases | Substance dependence | hsa05034 | Alcoholism | 2/114 | 188/8845 | 0.701793836 | 0.915381699 | 0.889439708 | SLC29A1/GNAI1 | 2 |
| hsa04912 | Organismal Systems | Endocrine system | hsa04912 | GnRH signaling pathway | 1/114 | 93/8845 | 0.702634894 | 0.915381699 | 0.889439708 | CACNA1D | 1 |
| hsa05222 | Human Diseases | Cancer: specific types | hsa05222 | Small cell lung cancer | 1/114 | 93/8845 | 0.702634894 | 0.915381699 | 0.889439708 | LAMB2 | 1 |
| hsa04080 | Environmental Information Processing | Signaling molecules and interaction | hsa04080 | Neuroactive ligand-receptor interaction | 4/114 | 368/8845 | 0.704507875 | 0.915381699 | 0.889439708 | CTSG/ADORA3/THRB/FPR3 | 4 |
| hsa05323 | Human Diseases | Immune disease | hsa05323 | Rheumatoid arthritis | 1/114 | 94/8845 | 0.706508251 | 0.915381699 | 0.889439708 | HLA-DRA | 1 |
| hsa04657 | Organismal Systems | Immune system | hsa04657 | IL-17 signaling pathway | 1/114 | 95/8845 | 0.710331592 | 0.915381699 | 0.889439708 | LCN2 | 1 |
| hsa04070 | Environmental Information Processing | Signal transduction | hsa04070 | Phosphatidylinositol signaling system | 1/114 | 98/8845 | 0.721507882 | 0.915381699 | 0.889439708 | PIP4P1 | 1 |
| hsa04925 | Organismal Systems | Endocrine system | hsa04925 | Aldosterone synthesis and secretion | 1/114 | 98/8845 | 0.721507882 | 0.915381699 | 0.889439708 | CACNA1D | 1 |
| hsa05215 | Human Diseases | Cancer: specific types | hsa05215 | Prostate cancer | 1/114 | 98/8845 | 0.721507882 | 0.915381699 | 0.889439708 | LEF1 | 1 |
| hsa05142 | Human Diseases | Infectious disease: parasitic | hsa05142 | Chagas disease | 1/114 | 103/8845 | 0.739192893 | 0.915381699 | 0.889439708 | GNAI1 | 1 |
| hsa04510 | Cellular Processes | Cellular community - eukaryotes | hsa04510 | Focal adhesion | 2/114 | 203/8845 | 0.741332241 | 0.915381699 | 0.889439708 | LAMB2/TNR | 2 |
| hsa05205 | Human Diseases | Cancer: overview | hsa05205 | Proteoglycans in cancer | 2/114 | 204/8845 | 0.743803655 | 0.915381699 | 0.889439708 | ERBB3/HSPG2 | 2 |
| hsa04064 | Environmental Information Processing | Signal transduction | hsa04064 | NF-kappa B signaling pathway | 1/114 | 105/8845 | 0.745951033 | 0.915381699 | 0.889439708 | EDAR | 1 |
| hsa04974 | Organismal Systems | Digestive system | hsa04974 | Protein digestion and absorption | 1/114 | 105/8845 | 0.745951033 | 0.915381699 | 0.889439708 | SLC16A10 | 1 |
| hsa05203 | Human Diseases | Cancer: overview | hsa05203 | Viral carcinogenesis | 2/114 | 205/8845 | 0.746255053 | 0.915381699 | 0.889439708 | CCR8/CHEK1 | 2 |
| hsa05415 | Human Diseases | Cardiovascular disease | hsa05415 | Diabetic cardiomyopathy | 2/114 | 205/8845 | 0.746255053 | 0.915381699 | 0.889439708 | RYR2/PDK4 | 2 |
| hsa04972 | Organismal Systems | Digestive system | hsa04972 | Pancreatic secretion | 1/114 | 106/8845 | 0.749264715 | 0.915381699 | 0.889439708 | RYR2 | 1 |
| hsa04922 | Organismal Systems | Endocrine system | hsa04922 | Glucagon signaling pathway | 1/114 | 107/8845 | 0.75253555 | 0.915381699 | 0.889439708 | CPT1A | 1 |
| hsa03013 | Genetic Information Processing | Translation | hsa03013 | Nucleocytoplasmic transport | 1/114 | 108/8845 | 0.755764086 | 0.915381699 | 0.889439708 | NUP160 | 1 |
| hsa04659 | Organismal Systems | Immune system | hsa04659 | Th17 cell differentiation | 1/114 | 108/8845 | 0.755764086 | 0.915381699 | 0.889439708 | HLA-DRA | 1 |
| hsa04931 | Human Diseases | Endocrine and metabolic disease | hsa04931 | Insulin resistance | 1/114 | 109/8845 | 0.758950865 | 0.915381699 | 0.889439708 | CPT1A | 1 |
| hsa05170 | Human Diseases | Infectious disease: viral | hsa05170 | Human immunodeficiency virus 1 infection | 2/114 | 213/8845 | 0.765157795 | 0.915381699 | 0.889439708 | CHEK1/GNAI1 | 2 |
| hsa04914 | Organismal Systems | Endocrine system | hsa04914 | Progesterone-mediated oocyte maturation | 1/114 | 111/8845 | 0.765201291 | 0.915381699 | 0.889439708 | GNAI1 | 1 |
| hsa05417 | Human Diseases | Cardiovascular disease | hsa05417 | Lipid and atherosclerosis | 2/114 | 216/8845 | 0.771928443 | 0.915381699 | 0.889439708 | OLR1/CYP2J2 | 2 |
| hsa04928 | Organismal Systems | Endocrine system | hsa04928 | Parathyroid hormone synthesis, secretion and action | 1/114 | 115/8845 | 0.777224169 | 0.915381699 | 0.889439708 | GNAI1 | 1 |
| hsa01200 | Metabolism | Global and overview maps | hsa01200 | Carbon metabolism | 1/114 | 116/8845 | 0.780133269 | 0.915381699 | 0.889439708 | PHGDH | 1 |
| hsa04670 | Organismal Systems | Immune system | hsa04670 | Leukocyte transendothelial migration | 1/114 | 116/8845 | 0.780133269 | 0.915381699 | 0.889439708 | GNAI1 | 1 |
| hsa03082 | Genetic Information Processing | Chromosome | hsa03082 | ATP-dependent chromatin remodeling | 1/114 | 117/8845 | 0.78300471 | 0.915381699 | 0.889439708 | SMARCA1 | 1 |
| hsa05166 | Human Diseases | Infectious disease: viral | hsa05166 | Human T-cell leukemia virus 1 infection | 2/114 | 223/8845 | 0.787074568 | 0.915381699 | 0.889439708 | CHEK1/HLA-DRA | 2 |
| hsa04668 | Environmental Information Processing | Signal transduction | hsa04668 | TNF signaling pathway | 1/114 | 119/8845 | 0.788636541 | 0.915381699 | 0.889439708 | LIF | 1 |
| hsa04152 | Environmental Information Processing | Signal transduction | hsa04152 | AMPK signaling pathway | 1/114 | 122/8845 | 0.796813725 | 0.919400452 | 0.893344569 | CPT1A | 1 |
| hsa04926 | Organismal Systems | Endocrine system | hsa04926 | Relaxin signaling pathway | 1/114 | 130/8845 | 0.817118161 | 0.937282596 | 0.910719932 | GNAI1 | 1 |
| hsa04270 | Organismal Systems | Circulatory system | hsa04270 | Vascular smooth muscle contraction | 1/114 | 134/8845 | 0.826502652 | 0.94049066 | 0.913837079 | CACNA1D | 1 |
| hsa04210 | Cellular Processes | Cell growth and death | hsa04210 | Apoptosis | 1/114 | 136/8845 | 0.83101428 | 0.94049066 | 0.913837079 | BIRC5 | 1 |
| hsa04915 | Organismal Systems | Endocrine system | hsa04915 | Estrogen signaling pathway | 1/114 | 139/8845 | 0.837564559 | 0.94049066 | 0.913837079 | GNAI1 | 1 |
| hsa05132 | Human Diseases | Infectious disease: bacterial | hsa05132 | Salmonella infection | 2/114 | 251/8845 | 0.839207051 | 0.94049066 | 0.913837079 | DYNC1I2/LEF1 | 2 |
| hsa04550 | Cellular Processes | Cellular community - eukaryotes | hsa04550 | Signaling pathways regulating pluripotency of stem cells | 1/114 | 144/8845 | 0.84792697 | 0.944832909 | 0.918056268 | LIF | 1 |
| hsa05224 | Human Diseases | Cancer: specific types | hsa05224 | Breast cancer | 1/114 | 148/8845 | 0.855742833 | 0.944850743 | 0.918073596 | LEF1 | 1 |
| hsa04072 | Environmental Information Processing | Signal transduction | hsa04072 | Phospholipase D signaling pathway | 1/114 | 149/8845 | 0.857633751 | 0.944850743 | 0.918073596 | PIK3R6 | 1 |
| hsa05020 | Human Diseases | Neurodegenerative disease | hsa05020 | Prion disease | 2/114 | 278/8845 | 0.878338447 | 0.962224703 | 0.934955177 | RYR2/CACNA1D | 2 |
| hsa05164 | Human Diseases | Infectious disease: viral | hsa05164 | Influenza A | 1/114 | 172/8845 | 0.894942895 | 0.974937791 | 0.947307975 | HLA-DRA | 1 |
| hsa05152 | Human Diseases | Infectious disease: bacterial | hsa05152 | Tuberculosis | 1/114 | 180/8845 | 0.905499048 | 0.979659996 | 0.951896353 | HLA-DRA | 1 |
| hsa05206 | Human Diseases | Cancer: overview | hsa05206 | MicroRNAs in cancer | 2/114 | 312/8845 | 0.915198729 | 0.979659996 | 0.951896353 | ERBB3/TNR | 2 |
| hsa04621 | Organismal Systems | Immune system | hsa04621 | NOD-like receptor signaling pathway | 1/114 | 189/8845 | 0.916122174 | 0.979659996 | 0.951896353 | DEFA3 | 1 |
| hsa05169 | Human Diseases | Infectious disease: viral | hsa05169 | Epstein-Barr virus infection | 1/114 | 203/8845 | 0.930340775 | 0.979659996 | 0.951896353 | HLA-DRA | 1 |
| hsa05165 | Human Diseases | Infectious disease: viral | hsa05165 | Human papillomavirus infection | 2/114 | 333/8845 | 0.932470345 | 0.979659996 | 0.951896353 | LAMB2/TNR | 2 |
| hsa04015 | Environmental Information Processing | Signal transduction | hsa04015 | Rap1 signaling pathway | 1/114 | 212/8845 | 0.93819113 | 0.979659996 | 0.951896353 | GNAI1 | 1 |
| hsa05163 | Human Diseases | Infectious disease: viral | hsa05163 | Human cytomegalovirus infection | 1/114 | 226/8845 | 0.948694283 | 0.979659996 | 0.951896353 | GNAI1 | 1 |
| hsa04810 | Cellular Processes | Cell motility | hsa04810 | Regulation of actin cytoskeleton | 1/114 | 230/8845 | 0.951355756 | 0.979659996 | 0.951896353 | ARHGEF4 | 1 |
| hsa05022 | Human Diseases | Neurodegenerative disease | hsa05022 | Pathways of neurodegeneration - multiple diseases | 3/114 | 483/8845 | 0.952765162 | 0.979659996 | 0.951896353 | RYR2/CACNA1D/DNAH6 | 3 |
| hsa04714 | Organismal Systems | Environmental adaptation | hsa04714 | Thermogenesis | 1/114 | 235/8845 | 0.954490887 | 0.979659996 | 0.951896353 | CPT1A | 1 |
| hsa05014 | Human Diseases | Neurodegenerative disease | hsa05014 | Amyotrophic lateral sclerosis | 2/114 | 371/8845 | 0.955646614 | 0.979659996 | 0.951896353 | DNAH6/NUP160 | 2 |
| hsa04144 | Cellular Processes | Transport and catabolism | hsa04144 | Endocytosis | 1/114 | 252/8845 | 0.963725687 | 0.979659996 | 0.951896353 | ACAP1 | 1 |
| hsa05010 | Human Diseases | Neurodegenerative disease | hsa05010 | Alzheimer disease | 2/114 | 391/8845 | 0.964588304 | 0.979659996 | 0.951896353 | CACNA1D/BACE2 | 2 |
| hsa05012 | Human Diseases | Neurodegenerative disease | hsa05012 | Parkinson disease | 1/114 | 271/8845 | 0.971862891 | 0.981934009 | 0.95410592 | GNAI1 | 1 |
| hsa05016 | Human Diseases | Neurodegenerative disease | hsa05016 | Huntington disease | 1/114 | 311/8845 | 0.983547921 | 0.988617756 | 0.960600248 | DNAH6 | 1 |
| hsa04740 | Organismal Systems | Sensory system | hsa04740 | Olfactory transduction | 1/114 | 453/8845 | 0.997601787 | 0.997601787 | 0.969329672 | OR10G2 | 1 |

| **Supplementary Table 6: Genes of red module** |
| --- |
| ABTB3 |
| ACBD4 |
| ACER3 |
| ACSL3 |
| ADAMTS17 |
| AEBP1 |
| AHDC1 |
| AKAP1 |
| AKR1B1 |
| ALS2CL |
| ANKMY1 |
| ANKS6 |
| AP3S1 |
| APBB1 |
| ATG9B |
| ATN1 |
| ATP6V0E2 |
| ATP6V0E2-AS1 |
| BAG3 |
| BCAT1 |
| BCL9 |
| BCL9L |
| CACNA1I |
| CACTIN |
| CARD11 |
| CARMIL2 |
| CD27 |
| CD5 |
| CD6 |
| CD7 |
| CDC42BPG |
| CDK20 |
| CEP72 |
| CEROX1 |
| CFAP410 |
| CGAS |
| CLSTN1 |
| CNTLN |
| COQ2 |
| CRIP2 |
| CRTC1 |
| D2HGDH |
| DBNDD1 |
| DBP |
| DCHS1 |
| ECHDC2 |
| EEIG1 |
| EPHX1 |
| EPPK1 |
| ERFL |
| FAR1 |
| FBLN2 |
| FBXL16 |
| FBXO31 |
| FCGBP |
| FCMR |
| FITM2 |
| FLNB |
| FLT3LG |
| GALNT12 |
| GAMT |
| GCNT1 |
| GEMIN4 |
| GFOD3P |
| GGT7 |
| GRAP |
| GRAPL |
| GTPBP3 |
| HAPLN3 |
| HIC2 |
| HID1 |
| IDH1 |
| IFT140 |
| IFT172 |
| IGSF8 |
| IL11RA |
| IL21R |
| IPO5P1 |
| ITPR3 |
| JAK2 |
| KAT2A |
| KDM8 |
| KLHL25 |
| KNSTRN |
| LAT |
| LBH |
| LCK |
| LDLRAP1 |
| LEPROT |
| LGMN |
| LIG1 |
| LINC00954 |
| LINC03049 |
| LMTK3 |
| LOC100129534 |
| LOC101927401 |
| LOC102723566 |
| LOC105370152 |
| LOC107984658 |
| LOC124901031 |
| LOC728743 |
| LRP5 |
| LSR |
| LTBP3 |
| LUC7L2 |
| LY9 |
| LZTS3 |
| MAL |
| MARCHF9 |
| MBOAT1 |
| MCF2L |
| MFGE8 |
| MFSD1 |
| MILR1 |
| MPI |
| MTA3 |
| N4BP3 |
| NAB2 |
| NDRG2 |
| NETO2 |
| NOSIP |
| NPTXR |
| NR1D1 |
| NR2C2AP |
| NRIP2 |
| NSUN5P1 |
| OBSCN |
| OLFM2 |
| PCED1B |
| PEG13 |
| PFAS |
| PHC1 |
| PHLDB3 |
| PKD1 |
| PLCG1 |
| PLEKHB1 |
| PLEKHG4 |
| PLXDC1 |
| PLXNA1 |
| POU6F1 |
| PPP1R13B |
| PRAG1 |
| PRDX3 |
| PRR12 |
| PSD |
| PUS1 |
| PVRIG |
| QTRT1 |
| RAI1 |
| RARG |
| RHPN1 |
| RNF217 |
| ROBO3 |
| RPAP1 |
| RRP7BP |
| RUSF1 |
| S1PR1 |
| SALL2 |
| SAMD10 |
| SANBR |
| SEC14L2 |
| SEMA4C |
| SEMA6C |
| SEPTIN1 |
| SETD1A |
| SH2D3A |
| SH3PXD2A |
| SIDT1 |
| SIRPG |
| SLC22A17 |
| SLC22A23 |
| SLC25A25-AS1 |
| SLC26A11 |
| SLC41A1 |
| SLC5A10 |
| SMG1P5 |
| SMYD5 |
| SORBS3 |
| SOX12 |
| SPPL2A |
| STMN3 |
| STPG1 |
| STX12 |
| SUSD3 |
| TCF3 |
| TFAP4 |
| TJP3 |
| TLE2 |
| TMEM170B |
| TMEM204 |
| TMEM25 |
| TMEM63A |
| TMIGD2 |
| TNKS1BP1 |
| TRAP1 |
| TRAV38-2DV8 |
| TRAV4 |
| TRBC1 |
| TRIQK |
| TRRAP |
| TSNARE1 |
| TSPOAP1-AS1 |
| TSPYL2 |
| TTC28 |
| TUT1 |
| TWNK |
| TYSND1 |
| UBAP1L |
| UBIAD1 |
| URB1 |
| USP36 |
| VEGFB |
| WAC-AS1 |
| WDR41 |
| YLPM1 |
| ZC4H2 |
| ZNF512B |
| ZNF609 |
| ZNF815P |
| ZNF853 |

| **Supplementary Table 7: GSEA of FBLN2** | | | | | | | | | |  |
| --- | --- | --- | --- | --- | --- | --- | --- | --- | --- | --- |
| ID | Description | setSize | enrichmentScore | NES | pvalue | p.adjust | qvalue | rank | leading_edge | core_enrichment |
| hsa03010 | Ribosome | 78 | 0.846202823 | 3.528808897 | 1.00E-10 | 2.77E-08 | 2.25E-08 | 395 | tags=77%, list=8%, signal=72% | RPSA2/RPL37A/RPLP1/RPS14/RPS29/RPS19/RPLP2/RPL18/RPS3/RPS27A/RPS21/RPS5/RPL18A/RPL38/RPS20/RPL37/RPL29/RPL13/RPL3/RPL27A/RPS11/RPL13A/RPS23/RPL22/RPL32/RPL27/RPL23A/RPL35/RPL36/RPL11/RPL10A/RPL10/RPL19/RPS27/RPS16/RPS8/RPL35A/RPL7/RPL8/RPSA/FAU/RPS12/RPS13/RPL5/RPL30/RPL34/RPS15/RPS6/RPL41/RPS4X/RPL39/RPL9/RPL24/RPLP0/RPS7/RPL31/RPL23/RPL4/RPS15A/RPL15 |
| hsa05164 | Influenza A | 86 | -0.637567613 | -2.452588798 | 2.97E-10 | 2.77E-08 | 2.25E-08 | 723 | tags=37%, list=14%, signal=33% | MYD88/IFNGR1/BID/TRIM25/RNASEL/PYCARD/CALCOCO2/ADAR/CASP1/CHUK/BAK1/MAPK3/TLR4/TBK1/JAK2/SOCS3/STAT2/IL1B/PML/FAS/TNFSF10/HLA-DPB1/MX2/STAT1/RIGI/OAS2/IFIH1/EIF2AK2/OAS1/MX1/OAS3/RSAD2 |
| hsa05171 | Coronavirus disease - COVID-19 | 134 | 0.501776978 | 2.26567693 | 2.54E-10 | 2.77E-08 | 2.25E-08 | 400 | tags=47%, list=8%, signal=45% | RPSA2/RPL37A/RPLP1/RPS14/RPS29/RPS19/RPLP2/RPL18/RPS3/RPS27A/RPS21/RPS5/RPL18A/RPL38/RPS20/RPL37/RPL29/RPL13/RPL3/RPL27A/RPS11/RPL13A/RPS23/RPL22/RPL32/RPL27/RPL23A/RPL35/RPL36/RPL11/RPL10A/RPL10/RPL19/RPS27/RPS16/RPS8/RPL35A/RPL7/RPL8/RPSA/FAU/RPS12/RPS13/RPL5/RPL30/RPL34/RPS15/RPS6/RPL41/RPS4X/RPL39/RPL9/RPL24/PLCG1/RPLP0/RPS7/RPL31/RPL23/RPL4/PRKCA/RPS15A/RPL15/IRF3 |
| hsa05160 | Hepatitis C | 85 | -0.591459598 | -2.264673151 | 5.04E-08 | 3.53E-06 | 2.86E-06 | 391 | tags=20%, list=8%, signal=19% | CHUK/BAK1/MAPK3/TBK1/SOCS3/STAT2/FAS/MX2/STAT1/RIGI/OAS2/EIF2AK2/OAS1/MX1/OAS3/IFIT1/RSAD2 |
| hsa04621 | NOD-like receptor signaling pathway | 100 | -0.550985129 | -2.16372013 | 2.66E-07 | 1.49E-05 | 1.21E-05 | 872 | tags=37%, list=17%, signal=31% | NFKBIA/P2RX7/NLRP12/PRKCD/GABARAP/TAB2/MYD88/PKN2/RBCK1/RNASEL/PYCARD/CARD16/CASP1/CHUK/GBP3/MEFV/MAPK3/TLR4/TANK/CARD6/TBK1/NAMPT/GBP2/IFI16/TXN/BCL2L1/STAT2/IL1B/GBP4/NLRC4/STAT1/OAS2/NOD2/GBP5/OAS1/GBP1/OAS3 |
| hsa03040 | Spliceosome | 86 | 0.505274934 | 2.144143032 | 8.28E-07 | 3.86E-05 | 3.14E-05 | 1906 | tags=72%, list=37%, signal=46% | SNRPD2/SNRNP70/SRSF8/SNU13/SNRPD3/SRSF6/HNRNPA1/HNRNPM/U2AF2/SNRPB/DHX38/PRPF6/SRSF5/SF3B3/PRPF19/SNRNP200/FUS/HNRNPA3/CCDC12/SRSF3/SRSF7/HNRNPU/SF3A2/ACIN1/DDX42/SRSF2/RBMX/PPIE/XAB2/DDX23/SNRNP40/RBM17/SRSF1/TRA2B/SRSF10/TXNL4A/SF3A3/PRPF3/DHX15/SART1/EFTUD2/PRPF38B/DDX5/WBP11/PLRG1/SF3A1/TCERG1/HNRNPC/SF3B1/PRPF38A/NCBP2/LSM4/HNRNPK/RBM8A/DDX46/HSPA8/CTNNBL1/PCBP1/SRSF4/LSM8/SRSF9/SF3B2 |
| hsa05340 | Primary immunodeficiency | 15 | 0.7854179 | 2.170897625 | 1.21E-05 | 0.000482286 | 0.000391631 | 209 | tags=53%, list=4%, signal=51% | CD79A/CD8B/CD8A/CD3E/LCK/CD3D/CIITA/IL7R |
| hsa05140 | Leishmaniasis | 45 | -0.608617282 | -2.09844994 | 4.16E-05 | 0.001456952 | 0.001183089 | 943 | tags=49%, list=18%, signal=40% | MAPK14/CR1/NFKBIA/NCF2/TAB2/MYD88/IFNGR1/FOS/FCGR3A/FCGR2A/NCF1/MAPK3/TLR2/TLR4/FCGR2C/JAK2/PTGS2/IL1B/FCGR3B/HLA-DPB1/STAT1/FCGR1A |
| hsa05162 | Measles | 84 | -0.512133217 | -1.956963592 | 5.26E-05 | 0.001636964 | 0.001329264 | 397 | tags=23%, list=8%, signal=21% | ADAR/CHUK/BAK1/TLR2/TLR4/TBK1/BCL2L1/STAT2/IL1B/FAS/MX2/STAT1/RIGI/OAS2/IFIH1/EIF2AK2/OAS1/MX1/OAS3 |
| hsa04613 | Neutrophil extracellular trap formation | 78 | -0.512385751 | -1.94200699 | 0.00013447 | 0.003765164 | 0.003057427 | 769 | tags=32%, list=15%, signal=28% | NCF2/SIGLEC9/CLEC7A/H3-3A/C5AR1/ITGA2B/H4C15/FCGR3A/FCGR2A/CASP1/NCF1/FPR1/MAPK3/TLR8/TLR2/TLR4/ATG7/AQP9/FPR2/FCGR3B/H2BC12/H2BC21/H2AC6/H2BC4/FCGR1A |
| hsa05150 | Staphylococcus aureus infection | 16 | -0.758239505 | -2.064420936 | 0.000245971 | 0.006261083 | 0.005084188 | 659 | tags=62%, list=13%, signal=55% | C5AR1/C3AR1/FCGR3A/FCGR2A/FPR1/FCGR2C/FPR2/FCGR3B/HLA-DPB1/FCGR1A |
| hsa04060 | Cytokine-cytokine receptor interaction | 53 | -0.53618518 | -1.898899512 | 0.000375787 | 0.008187356 | 0.00664838 | 739 | tags=43%, list=14%, signal=38% | CXCR2/IFNGR1/TNFSF14/RELL1/TNFRSF10C/TNFSF8/IL18RAP/PF4/IL1RAP/CXCR1/LTBR/IL13RA1/CCR3/CCR1/CXCL16/IL1B/FAS/TNFSF10/IL1R2/IL5RA/IL1RN/TNFSF13B/PPBP |
| hsa04668 | TNF signaling pathway | 68 | -0.50318184 | -1.868141271 | 0.000380127 | 0.008187356 | 0.00664838 | 1206 | tags=43%, list=23%, signal=33% | PIK3CA/TAB3/TNFRSF1A/JUNB/BIRC2/PIK3CB/MAP2K3/MAPK14/NFKBIA/RPS6KA5/CASP3/RHBDF2/MAP2K4/TAB2/FOS/IRF1/MAP3K5/CHUK/CREB5/MAPK3/BCL3/CEBPB/MLKL/SOCS3/PTGS2/IL1B/FAS/NOD2/MMP9 |
| hsa00190 | Oxidative phosphorylation | 71 | 0.431833011 | 1.762212793 | 0.000636636 | 0.012732726 | 0.010339356 | 1095 | tags=38%, list=21%, signal=30% | NDUFB8/ATP8/NDUFA13/NDUFV1/COX4I1/ATP5F1A/COX6C/SDHA/ND6/UQCRH/ATP5MC3/NDUFB11/ATP6/UQCR11/COX3/NDUFA10/COX11/COX7C/CYC1/CYCS/SDHC/UQCRB/COX6A1/ND3/COX2/COX6B1/ATP5F1B |
| hsa03320 | PPAR signaling pathway | 16 | -0.7076533 | -1.926692394 | 0.001093821 | 0.020417989 | 0.016580021 | 524 | tags=44%, list=10%, signal=39% | PLIN2/CD36/ACSL1/ACOX1/ACSL4/CYP27A1/GK |
| hsa05322 | Systemic lupus erythematosus | 24 | -0.647161349 | -1.917559651 | 0.001419826 | 0.024060824 | 0.019538113 | 530 | tags=58%, list=10%, signal=53% | ACTN1/RO60/H3-3A/H4C15/FCGR3A/FCGR2A/TRIM21/FCGR3B/H2BC12/H2BC21/HLA-DPB1/H2AC6/H2BC4/FCGR1A |
| hsa04217 | Necroptosis | 79 | -0.461109373 | -1.751282728 | 0.001460836 | 0.024060824 | 0.019538113 | 732 | tags=27%, list=14%, signal=23% | GLUL/IFNGR1/BID/RBCK1/PYCARD/PYGL/CASP1/ALOX15/TLR4/CHMP2B/MLKL/JAK2/STAT2/IL1B/FAS/TNFSF10/H2AC6/CHMP5/STAT1/ZBP1/EIF2AK2 |
| hsa05152 | Tuberculosis | 95 | -0.447239964 | -1.737662027 | 0.001910597 | 0.028687276 | 0.023294931 | 1005 | tags=33%, list=20%, signal=27% | TCIRG1/APAF1/MAPK14/CR1/ITGAX/ATP6V0C/CASP3/LAMP2/TLR6/CLEC7A/MYD88/IFNGR1/BID/TLR1/FCGR3A/FCGR2A/BCL10/MAPK3/TLR2/TLR4/FCGR2C/CEBPB/RAB5A/JAK2/CLEC4E/IL1B/FCGR3B/HLA-DPB1/STAT1/NOD2/FCGR1A |
| hsa04660 | T cell receptor signaling pathway | 79 | 0.386559936 | 1.610625491 | 0.001946637 | 0.028687276 | 0.023294931 | 1143 | tags=33%, list=22%, signal=26% | CD8B/CD8A/CD247/CARD11/CD3E/LCK/FYN/CD3D/LAT/PLCG1/NFATC2/ITK/NFATC3/CBLB/GRAP2/CD3G/CD4/PPP3CC/PIK3R1/RASGRP1/PPP2R1A/IKBKB/CD28/MAPK9/MAP3K14/AKT2 |
| hsa04610 | Complement and coagulation cascades | 15 | -0.708171504 | -1.891100856 | 0.002303331 | 0.031820182 | 0.025838945 | 1026 | tags=73%, list=20%, signal=59% | THBD/CR1/ITGAX/C5AR1/C3AR1/CD46/SERPINA1/CD55/CLU/PLAUR/F5 |
| hsa03008 | Ribosome biogenesis in eukaryotes | 28 | 0.537797138 | 1.787773422 | 0.002539441 | 0.031820182 | 0.025838945 | 1663 | tags=68%, list=32%, signal=46% | MDN1/SNU13/TCOF1/IMP3/NAT10/BMS1/IMP4/GTPBP4/GNL3/DKC1/NOP58/RAN/NOL6/NOP56/CSNK2A2/CSNK2A1/WDR75/REXO1/NXF1 |
| hsa05034 | Alcoholism | 53 | -0.498684437 | -1.766090654 | 0.002389747 | 0.031820182 | 0.025838945 | 781 | tags=25%, list=15%, signal=21% | GNAI3/SOS2/H3-3A/H4C15/CREB5/MAPK3/GNG5/GNB4/SLC29A1/H2BC12/H2BC21/H2AC6/H2BC4 |
| hsa04145 | Phagosome | 72 | -0.448269773 | -1.679939201 | 0.002613801 | 0.031820182 | 0.025838945 | 1387 | tags=64%, list=27%, signal=47% | ATP6V1G1/ATP6V0E1/RAB5C/STX7/SEC22B/ATP6V0B/PIK3C3/CYBB/ATP6V1C1/ATP6V0A1/CTSS/ATP6V0D1/TFRC/NCF4/TUBB4B/ATP6V1E1/ATP6V1A/TUBA1A/ATP6V1D/RAB7A/TCIRG1/ITGB3/ATP6V1B2/ATP6V0C/LAMP2/NCF2/TLR6/TUBA4A/CLEC7A/STX12/DYNC1LI1/VAMP3/FCGR3A/TUBB1/CD36/FCGR2A/HLA-A/NCF1/TLR2/TLR4/FCGR2C/TUBA1C/RAB5A/FCGR3B/HLA-DPB1/FCGR1A |
| hsa05415 | Diabetic cardiomyopathy | 107 | 0.362043876 | 1.580911118 | 0.003118811 | 0.03638613 | 0.029546632 | 1143 | tags=36%, list=22%, signal=28% | NDUFB8/PARP1/ATP8/NDUFA13/NDUFV1/COX4I1/ATP5F1A/PRKCA/COX6C/SDHA/ND6/SMAD3/UQCRH/ATP5MC3/NDUFB11/ATP2A2/SLC25A5/ATP6/UQCR11/CYBA/COX3/NDUFA10/ATP2A3/COX7C/CYC1/PIK3R1/SDHC/PTPA/TBC1D4/UQCRB/COX6A1/ND3/COX2/COX6B1/MTOR/ATP5F1B/MAPK9/AKT2 |
| hsa05169 | Epstein-Barr virus infection | 120 | -0.407563203 | -1.643391316 | 0.003567371 | 0.039954555 | 0.032444301 | 1054 | tags=32%, list=20%, signal=26% | PIK3CB/PSMC1/PSMD12/MAP2K3/APAF1/BTK/MAPK14/RB1/E2F3/NFKBIA/RELB/CASP3/RBPJ/MAP2K4/TAB2/MYD88/BID/GADD45B/LYN/MDM2/ENTPD1/CHUK/HLA-A/BAK1/TLR2/TBK1/CIR1/CD58/STAT2/FAS/HLA-DPB1/STAT1/RIGI/OAS2/EIF2AK2/OAS1/ISG15/OAS3 |
| hsa04623 | Cytosolic DNA-sensing pathway | 31 | -0.576487616 | -1.803482408 | 0.003710229 | 0.039956317 | 0.032445731 | 397 | tags=42%, list=8%, signal=39% | NFKBIA/CASP3/PYCARD/ADAR/CASP1/CHUK/MEFV/TBK1/MLKL/IFI16/IL1B/RIGI/ZBP1 |
| hsa04622 | RIG-I-like receptor signaling pathway | 35 | -0.568992622 | -1.859710522 | 0.004621437 | 0.047926008 | 0.03891736 | 397 | tags=23%, list=8%, signal=21% | ADAR/CHUK/ZNFX1/TANK/TBK1/RIGI/IFIH1/ISG15 |
| hsa04620 | Toll-like receptor signaling pathway | 54 | -0.47752757 | -1.700837897 | 0.005801766 | 0.058017661 | 0.047112086 | 1054 | tags=39%, list=20%, signal=31% | PIK3CB/MAP2K3/MAPK14/NFKBIA/MAP2K4/TLR6/TAB2/MYD88/TLR1/IRF5/FOS/CHUK/MAPK3/TLR8/TLR2/TLR4/TBK1/STAT2/IL1B/LY96/STAT1 |
| hsa00510 | N-Glycan biosynthesis | 23 | 0.557891225 | 1.76068023 | 0.006088559 | 0.058786089 | 0.047736072 | 1934 | tags=78%, list=38%, signal=49% | ST6GAL1/GANAB/MGAT5/MGAT4A/DDOST/MOGS/STT3A/MGAT1/MAN1A2/MAN1B1/MAGT1/B4GALT1/ALG13/MGAT2/STT3B/OSTC/B4GALT3/RPN1 |
| hsa05143 | African trypanosomiasis | 12 | -0.722062192 | -1.814893516 | 0.007178832 | 0.067002433 | 0.054407991 | 723 | tags=58%, list=14%, signal=50% | MYD88/GNAQ/HBA2/APOL1/IL1B/FAS/HBA1 |
| hsa04310 | Wnt signaling pathway | 56 | 0.396078302 | 1.546652644 | 0.008309167 | 0.07505054 | 0.060943296 | 1486 | tags=39%, list=29%, signal=28% | TCF7/LEF1/NFATC2/NFATC3/CCDC88C/PRKCA/CCAR2/PPARD/SMAD3/CTBP1/PRKACB/AXIN1/CCND2/PPP3CC/MYC/MAPK9/CSNK2A2/CSNK2A1/SKP1/TP53/CHD8/CTNNB1 |
| hsa04260 | Cardiac muscle contraction | 28 | 0.492933169 | 1.638634264 | 0.009241002 | 0.077804258 | 0.063179398 | 1088 | tags=50%, list=21%, signal=40% | COX4I1/COX6C/ATP1A1/UQCRH/ATP2A2/UQCR11/COX3/ATP2A3/COX7C/CYC1/UQCRB/COX6A1/COX2/COX6B1 |
| hsa04062 | Chemokine signaling pathway | 90 | -0.41596286 | -1.604919651 | 0.00944766 | 0.077804258 | 0.063179398 | 781 | tags=26%, list=15%, signal=22% | GNAI3/PRKCD/RAP1A/CXCR2/SOS2/GNAQ/FOXO3/LYN/PF4/CXCR1/PAK1/CHUK/NCF1/MAPK3/CCR3/GNG5/JAK2/CCR1/CXCL16/GNB4/STAT2/PPBP/STAT1 |
| hsa05020 | Prion disease | 138 | 0.316482877 | 1.437956511 | 0.009111835 | 0.077804258 | 0.063179398 | 1156 | tags=33%, list=22%, signal=27% | NDUFB8/ITPR3/ATP8/FYN/NDUFA13/NDUFV1/COX4I1/ATP5F1A/PRNP/COX6C/SOD1/SDHA/ND6/HSPA5/UQCRH/PRKACB/ATP5MC3/NDUFB11/SLC25A5/ATP6/UQCR11/CYBA/COX3/ITPR1/NDUFA10/COX7C/CYC1/CYCS/PPP3CC/PIK3R1/SDHC/PSMA7/PSMD2/STIP1/PSMA5/UQCRB/COX6A1/ND3/COX2/COX6B1/ATP5F1B/MAPK9/PSMD3/PSMC3/PSMC5/CSNK2A2 |
| hsa04918 | Thyroid hormone synthesis | 22 | 0.533289165 | 1.651727886 | 0.01108087 | 0.088646956 | 0.071983995 | 734 | tags=36%, list=14%, signal=31% | ITPR3/PDIA4/HSP90B1/PRKCA/ATP1A1/HSPA5/PRKACB/ITPR1 |
| hsa05168 | Herpes simplex virus 1 infection | 131 | -0.378223237 | -1.530891853 | 0.013023935 | 0.101297269 | 0.082256429 | 734 | tags=27%, list=14%, signal=24% | NFKBIA/ZNF33A/BST2/CASP3/TAB2/MYD88/PILRA/IFNGR1/BID/TNFSF14/RNASEL/ZNF200/SP100/CHUK/HLA-A/BAK1/TLR2/SRPK1/TBK1/ZNF267/JAK2/SOCS3/BCL2L1/STAT2/IL1B/PML/FAS/HLA-DPB1/STAT1/RIGI/OAS2/IFIH1/EIF2AK2/OAS1/OAS3 |
| hsa03082 | ATP-dependent chromatin remodeling | 58 | 0.386194662 | 1.516624004 | 0.015436498 | 0.116816742 | 0.094858708 | 1883 | tags=60%, list=37%, signal=39% | MBD3/CHD3/HDAC1/SMARCE1/INO80E/CHD4/TRRAP/MTA2/SMARCA2/MTA1/EP400/BAZ1B/SRCAP/H2AZ2/MEAF6/SMARCC1/RBBP7/POLE3/RBBP4/SMARCC2/SMARCA4/ARID1B/INO80/BRD9/SS18/SMARCD1/ACTR8/MORF4L1/BRD8/MCRS1/ARID1A/BPTF/YY1/CDK2AP2/PBRM1 |
| hsa03013 | Nucleocytoplasmic transport | 64 | 0.360950229 | 1.434824128 | 0.016143933 | 0.118955295 | 0.096595277 | 1710 | tags=47%, list=33%, signal=32% | NUP210/UPF3A/NUP188/IPO5/RANGAP1/NUP43/EEF1A1/PNN/UBE2I/RANBP2/ACIN1/NUP205/POM121/POM121C/RNPS1/NUP88/RAN/XPO4/NUP62/SUMO3/SAP18/IPO9/IPO7/SUMO2/NCBP2/TPR/NXF1/CSE1L/SRRM1/RBM8A |
| hsa00513 | Various types of N-glycan biosynthesis | 21 | 0.538833318 | 1.652590027 | 0.017353273 | 0.124587603 | 0.101168881 | 1934 | tags=71%, list=38%, signal=45% | MGAT4A/DDOST/STT3A/MGAT1/MAN1A2/MAN1B1/MAGT1/HEXA/B4GALT1/ALG13/MGAT2/STT3B/OSTC/B4GALT3/RPN1 |
| hsa05219 | Bladder cancer | 20 | -0.579832283 | -1.673754788 | 0.020118708 | 0.140830958 | 0.114358974 | 909 | tags=45%, list=18%, signal=37% | RB1/E2F3/RPS6KA5/MDM2/DAPK2/MAPK3/DAPK1/TYMP/MMP9 |
| hsa04920 | Adipocytokine signaling pathway | 33 | -0.482477542 | -1.534601973 | 0.020853562 | 0.14241457 | 0.115644914 | 458 | tags=24%, list=9%, signal=22% | CD36/CHUK/PRKAG2/ACSL1/ACSL4/JAK2/SOCS3/ADIPOR1 |
| hsa05417 | Lipid and atherosclerosis | 113 | -0.37002178 | -1.486645985 | 0.022111045 | 0.147406966 | 0.11969889 | 825 | tags=29%, list=16%, signal=25% | NFKBIA/ATF6/CASP3/NCF2/HSPA6/MAP2K4/TLR6/RAP1A/TAB2/MYD88/BID/FOS/PYCARD/LYN/NFE2L2/CD36/MAP3K5/CASP1/CHUK/NCF1/MAPK3/TLR2/TLR4/TANK/TBK1/JAK2/BCL2L1/ABCA1/IL1B/FAS/TNFSF10/LY96/MMP9 |
| hsa05161 | Hepatitis B | 99 | -0.377498973 | -1.479732171 | 0.023463336 | 0.149312141 | 0.121245949 | 1003 | tags=26%, list=20%, signal=22% | MAP2K3/APAF1/MAPK14/RB1/E2F3/NFKBIA/CASP3/MAP2K4/TAB2/MYD88/SOS2/BID/FOS/CHUK/CREB5/MAPK3/TLR2/TLR4/TBK1/JAK2/STAT2/FAS/STAT1/RIGI/MMP9/IFIH1 |
| hsa04110 | Cell cycle | 71 | 0.348650687 | 1.422764554 | 0.023312952 | 0.149312141 | 0.121245949 | 1102 | tags=30%, list=21%, signal=24% | ATM/ANAPC1/TFDP2/ANAPC2/CDC25B/HDAC1/SMC1A/SMAD3/MCM3/BUB3/MCM7/YWHAQ/ANAPC16/CCND2/RBL2/PDS5A/CDC14A/PPP2R1A/MYC/ANAPC5/ATR |
| hsa04380 | Osteoclast differentiation | 80 | -0.378239357 | -1.437376642 | 0.024666387 | 0.153479743 | 0.124630167 | 975 | tags=30%, list=19%, signal=25% | ITGB3/BTK/SPI1/MAPK14/NFKBIA/RELB/NCF2/TAB2/IFNGR1/FOS/FCGR3A/GAB2/FCGR2A/CHUK/NCF1/MAPK3/FCGR2C/SOCS3/SIRPB1/STAT2/IL1B/FCGR3B/STAT1/FCGR1A |
| hsa04966 | Collecting duct acid secretion | 14 | -0.618898297 | -1.630397235 | 0.025893545 | 0.157021855 | 0.127506469 | 1726 | tags=86%, list=34%, signal=57% | ATP6V1G1/ATP6V0E1/ATP6V1C1/ATP6V0A1/ATP6V0D1/ATP6V1E1/ATP6V1A/ATP6V1D/TCIRG1/ATP6V1B2/ATP6V0C/SLC4A1 |
| hsa05016 | Huntington disease | 152 | 0.279547195 | 1.284944303 | 0.02635724 | 0.157021855 | 0.127506469 | 1425 | tags=36%, list=28%, signal=26% | NDUFB8/ATP8/NDUFA13/NDUFV1/COX4I1/ATP5F1A/COX6C/HDAC1/SOD1/SDHA/ND6/HTT/ERN1/UQCRH/ATP5MC3/NDUFB11/SIN3A/SLC25A5/ATP6/UQCR11/COX3/ITPR1/NDUFA10/COX7C/CYC1/CYCS/SDHC/PSMA7/POLR2G/PSMD2/POLR2B/PSMA5/UQCRB/COX6A1/ND3/COX2/COX6B1/MTOR/ATP5F1B/MAPK9/PSMD3/PSMC3/PSMC5/POLR2D/ND4/DCTN1/PSMB6/PSMB4/KLC1/MAP2K7/DCTN3/TFAM/TP53/POLR2E |
| hsa05166 | Human T-cell leukemia virus 1 infection | 115 | 0.30167298 | 1.339091706 | 0.029038829 | 0.169393171 | 0.137552349 | 1143 | tags=32%, list=22%, signal=26% | IL2RB/ETS1/CD3E/LCK/ATM/CD3D/TCF3/ANAPC1/NFATC2/CRTC3/KAT2A/NFATC3/ANAPC2/ELK4/CALR/HLA-C/SMAD3/TRRAP/PRKACB/CD3G/SLC25A5/BUB3/ANAPC16/CCND2/HLA-DRB1/CD4/PPP3CC/PIK3R1/RAN/JAK1/MYC/IKBKB/ANAPC5/ATR/MAPK9/MAP3K14/AKT2 |
| hsa04714 | Thermogenesis | 106 | 0.305235505 | 1.331705952 | 0.030518168 | 0.174389533 | 0.141609546 | 1264 | tags=36%, list=25%, signal=28% | NDUFB8/ATP8/RPS6/NDUFA13/NDUFV1/COX4I1/RPTOR/ATP5F1A/COX6C/SDHA/ND6/SMARCE1/UQCRH/PRKACB/ATP5MC3/NDUFB11/SMARCA2/ATP6/UQCR11/COX3/NDUFA10/COX11/COX7C/CYC1/SDHC/SMARCC1/UQCRB/COX6A1/ND3/COX2/COX6B1/MTOR/ATP5F1B/SMARCC2/SMARCA4/ARID1B/ND4/ADCY7 |
| hsa04625 | C-type lectin receptor signaling pathway | 61 | -0.407525365 | -1.477667927 | 0.032340585 | 0.181107278 | 0.147064557 | 943 | tags=31%, list=18%, signal=26% | MAPK14/NFKBIA/RELB/PRKCD/CLEC7A/PYCARD/IRF1/MDM2/PAK1/CASP1/CHUK/BCL10/MAPK3/BCL3/CLEC4E/STAT2/PTGS2/IL1B/STAT1 |
| hsa04657 | IL-17 signaling pathway | 40 | -0.458965836 | -1.534069497 | 0.035089403 | 0.192647702 | 0.156435728 | 1000 | tags=40%, list=19%, signal=32% | IL17RA/MAPK14/S100A9/NFKBIA/CASP3/TAB2/FOS/USP25/S100A8/CHUK/MAPK3/CEBPB/TBK1/PTGS2/IL1B/MMP9 |
| hsa04666 | Fc gamma R-mediated phagocytosis | 60 | -0.39911545 | -1.447820017 | 0.036290508 | 0.195410428 | 0.158679144 | 747 | tags=25%, list=15%, signal=22% | PRKCD/ARPC1A/LIMK2/ASAP1/LYN/FCGR3A/GAB2/FCGR2A/PAK1/NCF1/MAPK3/MARCKS/GSN/FCGR3B/FCGR1A |
| hsa04390 | Hippo signaling pathway | 45 | 0.392129827 | 1.451889216 | 0.037868121 | 0.196353218 | 0.159444719 | 2132 | tags=58%, list=41%, signal=34% | TCF7/RASSF1/LLGL2/LEF1/LIMD1/SMAD3/YWHAQ/AXIN1/CCND2/PPP2R1A/MYC/NF2/YWHAG/BIRC3/PPP1CA/CTNNB1/FBXW11/SMAD4/CSNK1D/PPP2R2D/ID2/DLG1/ACTB/YWHAB/ITGB2/TGFB1 |
| hsa04662 | B cell receptor signaling pathway | 52 | 0.372192417 | 1.431405473 | 0.037682984 | 0.196353218 | 0.159444719 | 342 | tags=15%, list=7%, signal=15% | CD79A/CD79B/CD22/BANK1/CARD11/CD81/NFATC2/NFATC3 |
| hsa05323 | Rheumatoid arthritis | 27 | -0.509344705 | -1.543106713 | 0.040074885 | 0.204017597 | 0.165668425 | 1780 | tags=74%, list=35%, signal=49% | ATP6AP1/ATP6V1G1/CD86/ATP6V0E1/ATP6V0B/ATP6V1C1/ATP6V0A1/ATP6V0D1/ATP6V1E1/ATP6V1A/ATP6V1D/TCIRG1/ATP6V1B2/ATP6V0C/FOS/TLR2/TLR4/IL1B/HLA-DPB1/TNFSF13B |
| hsa04064 | NF-kappa B signaling pathway | 59 | -0.397008154 | -1.432760833 | 0.040849673 | 0.204248366 | 0.165855816 | 872 | tags=32%, list=17%, signal=27% | NFKBIA/RELB/TAB2/MYD88/TRIM25/TNFSF14/GADD45B/LYN/CHUK/LTBR/BCL10/TLR4/BCL2L1/PTGS2/IL1B/LY96/TNFSF13B/RIGI/BCL2A1 |
| hsa05145 | Toxoplasmosis | 54 | -0.397581761 | -1.416090232 | 0.042207792 | 0.207336523 | 0.168363492 | 1138 | tags=37%, list=22%, signal=29% | TNFRSF1A/BIRC2/MAP2K3/MAPK14/NFKBIA/CASP3/GNAI3/HSPA6/TAB2/MYD88/IFNGR1/CHUK/MAPK3/TLR2/TLR4/JAK2/BCL2L1/LY96/HLA-DPB1/STAT1 |
| hsa00512 | Mucin type O-glycan biosynthesis | 10 | -0.668326742 | -1.597733755 | 0.046413686 | 0.214647954 | 0.174300594 | 614 | tags=50%, list=12%, signal=44% | GALNT7/C1GALT1/B4GALT5/GALNT3/ST6GALNAC2 |
| hsa05133 | Pertussis | 33 | -0.455847627 | -1.449900995 | 0.04676259 | 0.214647954 | 0.174300594 | 943 | tags=36%, list=18%, signal=30% | MAPK14/CASP3/GNAI3/MYD88/FOS/PYCARD/IRF1/CASP1/MAPK3/TLR4/IL1B/LY96 |
| hsa05167 | Kaposi sarcoma-associated herpesvirus infection | 109 | -0.352856653 | -1.411473595 | 0.045148233 | 0.214647954 | 0.174300594 | 963 | tags=28%, list=19%, signal=23% | HCK/MAPK14/RB1/E2F3/UBB/NFKBIA/CASP3/MAP2K4/IFNGR1/BID/FOS/LYN/HIF1A/CHUK/HLA-A/ATG3/BAK1/MAPK3/CCR3/GNG5/TBK1/CLEC2B/JAK2/CCR1/GNB4/STAT2/PTGS2/FAS/STAT1/EIF2AK2 |
| hsa05165 | Human papillomavirus infection | 133 | -0.33436186 | -1.362681722 | 0.045305016 | 0.214647954 | 0.174300594 | 1054 | tags=24%, list=20%, signal=20% | PIK3CB/PSMC1/TCIRG1/ITGB3/ATP6V1B2/RB1/PPP2R2A/ATP6V0C/PPP2R3C/CASP3/RBPJ/SOS2/APC/IRF1/ITGA2B/PPP2R5A/MDM2/CHUK/CREB5/HLA-A/BAK1/MAPK3/TBK1/STAT2/PTGS2/FAS/MX2/STAT1/EIF2AK2/OASL/MX1/ISG15 |
| hsa04612 | Antigen processing and presentation | 30 | 0.447558847 | 1.518860637 | 0.047637877 | 0.215138799 | 0.174699175 | 814 | tags=40%, list=16%, signal=34% | CD8B/CD8A/PSME1/HSP90AB1/KLRD1/CIITA/CD74/CALR/HLA-C/HSPA5/HLA-DRB1/CD4 |
| hsa05144 | Malaria | 17 | -0.565842526 | -1.579859394 | 0.04993342 | 0.21845871 | 0.177395043 | 458 | tags=53%, list=9%, signal=48% | CR1/MYD88/CD36/TLR2/TLR4/GYPC/HBA2/IL1B/HBA1 |
| hsa04140 | Autophagy - animal | 110 | -0.34622044 | -1.38927055 | 0.049326343 | 0.21845871 | 0.177395043 | 1728 | tags=51%, list=34%, signal=35% | ATG13/CFLAR/STX7/IGBP1/ATG16L2/PIK3C3/BECN1/IRS2/RAF1/UBC/NRAS/GORASP1/VPS41/CTSB/ZFYVE1/TRAF6/ULK1/KRAS/MAP1LC3B/RAB1A/MTMR3/PIK3CA/STX17/CAMKK2/RUBCN/RB1CC1/RAB7A/PIK3CB/GABARAPL2/SH3GLB1/GABARAPL1/RAB8A/SUPT20H/OPTN/UBB/TAX1BP1/VMP1/RRAGC/LAMP2/PRKCD/GABARAP/CALCOCO2/SNAP29/C9orf72/ATG2A/HIF1A/WDFY3/ATG3/DAPK2/MAPK3/ATG7/DAPK1/TANK/TBK1/BCL2L1/NRBF2 |
| hsa03420 | Nucleotide excision repair | 20 | 0.496473367 | 1.500035327 | 0.052017126 | 0.220678716 | 0.179197754 | 2125 | tags=70%, list=41%, signal=41% | DDB1/RPA1/POLR2G/POLR2B/RPA2/POLE3/POLR2D/POLR2E/CUL4A/RFC1/ERCC3/RAD23A/POLD4/XPC |
| hsa05012 | Parkinson disease | 146 | 0.277847437 | 1.274784151 | 0.051421785 | 0.220678716 | 0.179197754 | 1150 | tags=32%, list=22%, signal=26% | RPS27A/NDUFB8/ITPR3/ATP8/NDUFA13/NDUFV1/COX4I1/PLCG1/UBE2G2/PARK7/ATP5F1A/SLC39A10/COX6C/SOD1/SDHA/ND6/ERN1/XBP1/HSPA5/UQCRH/PRKACB/ATP5MC3/NDUFB11/SLC25A5/ATP6/UQCR11/COX3/ITPR1/NDUFA10/COX7C/CYC1/CYCS/SDHC/PSMA7/PSMD2/PSMA5/UQCRB/COX6A1/ND3/COX2/COX6B1/ATP5F1B/MAPK9/CALM1/PSMD3/PSMC3/PSMC5 |
| hsa05321 | Inflammatory bowel disease | 24 | -0.496718097 | -1.471791512 | 0.053097345 | 0.221899353 | 0.180188948 | 700 | tags=33%, list=14%, signal=29% | IFNGR1/IL18RAP/TLR2/TLR4/IL1B/HLA-DPB1/STAT1/NOD2 |
| hsa00564 | Glycerophospholipid metabolism | 32 | -0.449742592 | -1.431500772 | 0.055258467 | 0.227534864 | 0.184765153 | 1091 | tags=38%, list=21%, signal=30% | AGPAT3/LPIN2/LPGAT1/SAMD8/LYPLA1/PHOSPHO1/MBOAT1/PISD/GPAT3/MBOAT2/CHPT1/LPCAT2 |
| hsa04061 | Viral protein interaction with cytokine and cytokine receptor | 25 | -0.485799489 | -1.455498733 | 0.056239016 | 0.228216296 | 0.185318496 | 739 | tags=44%, list=14%, signal=38% | CXCR2/TNFSF14/TNFRSF10C/IL18RAP/PF4/CXCR1/LTBR/CCR3/CCR1/TNFSF10/PPBP |
| hsa00240 | Pyrimidine metabolism | 17 | -0.529149366 | -1.477410337 | 0.058076225 | 0.2323049 | 0.188638566 | 871 | tags=47%, list=17%, signal=39% | DPYD/NT5C2/DUT/RRM2B/ENTPD1/CDA/TYMP/NT5C3A |
| hsa03030 | DNA replication | 11 | 0.575114681 | 1.472035318 | 0.062098501 | 0.244895497 | 0.198862509 | 1501 | tags=64%, list=29%, signal=45% | RPA1/MCM3/MCM7/RPA2/POLE3/RNASEH1/MCM5 |
| hsa03250 | Viral life cycle - HIV-1 | 47 | -0.395921792 | -1.374706911 | 0.063758389 | 0.247949292 | 0.201342282 | 222 | tags=19%, list=4%, signal=18% | KAT2B/BST2/ELL/SUPT4H1/APOBEC3A/TRIM5/APOBEC3A_B/MX2/MX1 |

| **Supplementary Table 8: GSEA of OBSCN** | | | | | | | | | | |
| --- | --- | --- | --- | --- | --- | --- | --- | --- | --- | --- |
| ID | Description | setSize | enrichmentScore | NES | pvalue | p.adjust | qvalue | rank | leading_edge | core_enrichment |
| hsa03010 | Ribosome | 78 | 0.869915559 | 3.76012918 | 1.00E-10 | 1.40E-08 | 1.22E-08 | 504 | tags=85%, list=10%, signal=77% | RPS29/RPS21/RPLP1/RPS3/RPL37A/RPL13/RPL36/RPS19/RPS14/RPL10A/RPLP2/RPS16/RPL38/RPL18/RPL37/RPS5/RPL8/RPL29/RPL3/RPS27/RPS23/RPL27A/RPL22/RPL35/RPL18A/RPS8/RPL10/RPS27A/RPL13A/RPL27/RPL32/RPS20/RPL35A/RPS11/RPL23A/RPL19/RPS4X/RPL11/RPLP0/RPL5/RPSA/RPL34/RPL30/RPL23/RPS15/RPL7/RPS13/FAU/RPL31/RPL39/RPL4/RPS6/RPSA2/RPL14/RPS12/RPL41/RPL12/RPS7/RPL15/RPL28/RPS15A/RPL26/RPL6/RPS26/RPS24/RPS28 |
| hsa05171 | Coronavirus disease - COVID-19 | 134 | 0.611784835 | 2.912707626 | 1.00E-10 | 1.40E-08 | 1.22E-08 | 394 | tags=49%, list=8%, signal=47% | RPS29/RPS21/RPLP1/PLCG1/RPS3/RPL37A/RPL13/RPL36/RPS19/RPS14/RPL10A/RPLP2/RPS16/RPL38/RPL18/RPL37/RPS5/RPL8/RPL29/RPL3/RPS27/RPS23/RPL27A/RPL22/RPL35/RPL18A/RPS8/RPL10/RPS27A/RPL13A/RPL27/RPL32/RPS20/RPL35A/RPS11/RPL23A/RPL19/RPS4X/RPL11/RPLP0/RPL5/RPSA/RPL34/RPL30/RPL23/RPS15/RPL7/RPS13/FAU/RPL31/RPL39/PRKCA/RPL4/RPS6/RPSA2/RPL14/RPS12/RPL41/RPL12/RPS7/RPL15/RPL28/RPS15A/IRF3/RPL26/RPL6 |
| hsa03040 | Spliceosome | 86 | 0.496620281 | 2.180886347 | 3.15E-07 | 2.94E-05 | 2.57E-05 | 1443 | tags=53%, list=28%, signal=39% | SNRNP70/SNRPD2/SF3B3/HNRNPM/PRPF19/HNRNPA1/SNRNP200/SNU13/U2AF2/DHX38/PRPF6/SRSF7/SRSF6/SNRPB/HSPA8/EFTUD2/SNRPD3/FUS/SRSF5/SRSF8/RBMX/CCDC12/SF3A2/SRSF3/HNRNPA3/PPIE/ACIN1/DDX42/HNRNPU/DDX23/TRA2B/SF3A3/XAB2/RBM17/SART1/DDX5/SRSF2/SNRNP40/LSM4/WBP11/SF3B5/PRPF3/SRSF1/SF3A1/DHX15/SF3B2 |
| hsa04610 | Complement and coagulation cascades | 15 | -0.792185747 | -2.239501266 | 5.17E-06 | 0.000361732 | 0.000315496 | 863 | tags=80%, list=17%, signal=67% | ITGAX/CD59/CD46/C5AR1/SERPINA1/CLU/PLAUR/CR1/THBD/C3AR1/F5/CD55 |
| hsa05340 | Primary immunodeficiency | 15 | 0.732929211 | 2.103435059 | 0.000101456 | 0.005681549 | 0.004955336 | 739 | tags=60%, list=14%, signal=52% | CD79A/LCK/CD3E/CIITA/IL7R/CD4/CD3D/RFX5/CD8B |
| hsa04621 | NOD-like receptor signaling pathway | 100 | -0.412395678 | -1.791481594 | 0.000130292 | 0.006080282 | 0.005303103 | 1095 | tags=50%, list=21%, signal=40% | FADD/ERBIN/MAPK13/CASP1/TNFAIP3/BIRC2/TAB2/TRAF6/IRAK4/STAT1/XIAP/MAPK1/ATG12/CARD8/GBP2/NOD2/OAS1/MAP1LC3B/NLRP12/ANTXR2/GABARAPL2/CARD16/GBP5/MEFV/RNASEL/IFI16/PYCARD/MFN1/OAS3/NFKBIA/MAPK3/MAPK14/IFNAR1/GABARAPL1/TAB3/TANK/NEK7/GBP1/TBK1/CHUK/PKN2/CARD6/GBP3/BCL2L1/TXN/TLR4/IL1B/NAMPT/NLRC4/NLRP6 |
| hsa04612 | Antigen processing and presentation | 30 | 0.574204248 | 2.025147047 | 0.000215839 | 0.008633554 | 0.007530017 | 739 | tags=37%, list=14%, signal=32% | HLA-DPB1/CIITA/HLA-DRB1/CD74/CD4/HSP90AB1/HSPA8/CALR/RFX5/HSP90AA1/CD8B |
| hsa05140 | Leishmaniasis | 45 | -0.504812876 | -1.908345237 | 0.000732949 | 0.025653217 | 0.022374235 | 591 | tags=38%, list=11%, signal=34% | NFKBIA/MAPK3/MAPK14/NCF4/JAK2/FCGR2A/IFNGR1/FOS/CR1/TLR2/TLR4/FCGR2C/FCGR3A/IL1B/FCGR1A/FCGR3B/PTGS2 |
| hsa04668 | TNF signaling pathway | 68 | -0.436896185 | -1.782922209 | 0.000979979 | 0.028147566 | 0.024549757 | 1288 | tags=49%, list=25%, signal=37% | TNFAIP3/BIRC2/TNFRSF1A/CREB1/TAB2/ATF2/XIAP/MAPK1/CASP3/NOD2/MAP3K8/PIK3CB/CFLAR/PIK3CA/MAP3K5/BCL3/ADAM17/NFKBIA/MAPK3/MAPK14/MAP2K4/MLKL/TAB3/RPS6KA5/CHUK/CEBPB/FOS/FAS/IL1B/SOCS3/PTGS2/CREB5/MMP9 |
| hsa04613 | Neutrophil extracellular trap formation | 78 | -0.408902571 | -1.714342688 | 0.00100527 | 0.028147566 | 0.024549757 | 980 | tags=33%, list=19%, signal=27% | ATG7/CLEC7A/PIK3CB/PIK3CA/H3-3A/ITGA2B/C5AR1/H2AC6/MAPK3/MAPK14/FPR1/NCF4/H4C15/FCGR2A/H2BC12/CR1/FPR2/TLR2/TLR8/TLR4/H2BC21/AQP9/FCGR3A/FCGR1A/FCGR3B/H2BC4 |
| hsa03008 | Ribosome biogenesis in eukaryotes | 28 | 0.541941366 | 1.876166313 | 0.001447073 | 0.036834595 | 0.032126414 | 1705 | tags=71%, list=33%, signal=48% | MDN1/SNU13/TCOF1/IMP4/RAN/NAT10/IMP3/NOL6/NXF1/DKC1/LSG1/NOP56/BMS1/GTPBP4/NOP58/EIF6/GNL3/REXO1/CSNK2A1/CSNK2A2 |
| hsa03320 | PPAR signaling pathway | 16 | -0.654896517 | -1.88397504 | 0.002589012 | 0.060410285 | 0.052688669 | 617 | tags=38%, list=12%, signal=33% | ACSL3/CYP27A1/ACOX1/ACSL4/ACSL1/GK |
| hsa01230 | Biosynthesis of amino acids | 24 | 0.526368134 | 1.743017937 | 0.005659229 | 0.099752362 | 0.08700206 | 1323 | tags=62%, list=26%, signal=47% | PYCR2/PFKL/SHMT2/ENO1/PKM/PFKP/IDH2/MTR/TPI1/MAT2A/ACO2/PRPS1/GOT2/CS/IDH3B |
| hsa05160 | Hepatitis C | 85 | -0.375899846 | -1.602618675 | 0.004692388 | 0.099752362 | 0.08700206 | 1142 | tags=36%, list=22%, signal=29% | NRAS/STAT1/PPP2R2A/MAPK1/CASP3/OAS1/BRAF/GSK3B/PIK3CB/CFLAR/PIK3CA/PIAS1/RNASEL/APAF1/RB1/OAS3/NFKBIA/MAPK3/IFNAR1/KRAS/MX2/TBK1/CHUK/E2F3/RIGI/SOS2/FAS/EIF2AK2/SOCS3/IFIT1/RSAD2 |
| hsa04140 | Autophagy - animal | 110 | -0.348768613 | -1.543206415 | 0.005448198 | 0.099752362 | 0.08700206 | 1635 | tags=44%, list=32%, signal=30% | RAF1/GABARAP/RPS6KB1/MAP3K7/ZFYVE1/ATG2A/ULK1/RAB7A/PIK3C3/CALCOCO2/RAB1A/CAMKK2/TRAF6/STX7/SUPT20H/ERN1/NRAS/TAX1BP1/MAPK1/ATG12/MAP1LC3B/ATG7/PIK3CB/GABARAPL2/MTMR3/RRAGC/ATG3/CFLAR/PIK3CA/PRKAA1/HIF1A/MAPK3/VMP1/RB1CC1/GABARAPL1/LAMP2/SH3GLB1/KRAS/SNAP29/TANK/DAPK2/IGF1R/TBK1/C9orf72/BCL2L1/WDFY3/NRBF2/IRS2 |
| hsa05417 | Lipid and atherosclerosis | 113 | -0.337546866 | -1.503990043 | 0.005700135 | 0.099752362 | 0.08700206 | 932 | tags=32%, list=18%, signal=27% | GSK3B/PIK3CB/SOD2/PIK3CA/PPP3R1/APAF1/MAP3K5/PYCARD/PPP3CA/LYN/RAP1A/VAV3/NFE2L2/NFKBIA/MAPK3/MAPK14/MIB1/MAP2K4/NCF4/KRAS/JAK2/TANK/TNFSF10/TLR6/TBK1/CHUK/ATF6/FOS/TLR2/BCL2L1/TLR4/FAS/IL1B/ABCA1/LY96/MMP9 |
| hsa00512 | Mucin type O-glycan biosynthesis | 10 | -0.704070342 | -1.780370719 | 0.006814746 | 0.112242875 | 0.097896042 | 384 | tags=50%, list=7%, signal=46% | C1GALT1/GALNT7/B4GALT5/ST6GALNAC2/GALNT3 |
| hsa05034 | Alcoholism | 53 | -0.423279949 | -1.634426182 | 0.007846016 | 0.122049133 | 0.106448868 | 1276 | tags=42%, list=25%, signal=32% | CREB1/CAMKK2/PPP1CB/HDAC4/ATF2/SLC29A1/NRAS/MAPK1/BRAF/H3-3A/GNAI3/H2AC6/MAPK3/GNG5/KRAS/H4C15/GNB4/H2BC12/SOS2/H2BC21/CREB5/H2BC4 |
| hsa00970 | Aminoacyl-tRNA biosynthesis | 19 | 0.540481035 | 1.672262483 | 0.011127067 | 0.141617219 | 0.12351577 | 2254 | tags=84%, list=44%, signal=47% | AARS1/QARS1/LARS1/YARS1/KARS1/SARS1/EPRS1/HARS1/IARS2/NARS1/MARS1/GATC/TARS1/GARS1/DARS1/CARS2 |
| hsa04620 | Toll-like receptor signaling pathway | 54 | -0.423244039 | -1.645381847 | 0.010490553 | 0.141617219 | 0.12351577 | 1245 | tags=43%, list=24%, signal=33% | TAB2/TRAF6/IRAK4/STAT1/MAPK1/MAP3K8/PIK3CB/PIK3CA/NFKBIA/MAPK3/MAPK14/MAP2K4/IFNAR1/TLR6/TLR1/TBK1/CHUK/FOS/TLR2/TLR8/TLR4/IL1B/LY96 |
| hsa04060 | Cytokine-cytokine receptor interaction | 53 | -0.41066885 | -1.585730489 | 0.011064827 | 0.141617219 | 0.12351577 | 834 | tags=40%, list=16%, signal=34% | CCR1/PF4/TNFRSF10C/TNFSF14/IL18RAP/CXCR2/IL5RA/IFNAR1/TNFSF10/IFNGR1/CXCR1/IL13RA1/IL1RN/PPBP/FAS/IL1B/CXCL16/TNFSF13B/IL1RAP/RELL1/IL1R2 |
| hsa03082 | ATP-dependent chromatin remodeling | 58 | 0.386167045 | 1.549017165 | 0.010961524 | 0.141617219 | 0.12351577 | 1791 | tags=55%, list=35%, signal=36% | CHD3/MBD3/TRRAP/MTA2/SMARCA4/MTA1/HDAC1/CHD4/INO80E/SMARCC2/EP400/SRCAP/BAZ1B/ARID1B/RBBP7/BRD9/H2AZ2/SMARCE1/SMARCA2/RBBP4/MCRS1/POLE3/INO80/SMARCD1/ACTB/CDK2AP2/SMARCC1/NFRKB/ARID1A/SS18/SMARCD2/MEAF6 |
| hsa04514 | Cell adhesion molecules | 36 | 0.431960606 | 1.592729482 | 0.015159918 | 0.169791083 | 0.148088463 | 827 | tags=36%, list=16%, signal=31% | CD22/HLA-DPB1/CD28/CD6/HLA-DRB1/ITGB7/CD4/SPN/ITGA6/GLG1/CD8B/TIGIT/CD2 |
| hsa04917 | Prolactin signaling pathway | 36 | -0.440271494 | -1.584158564 | 0.015155513 | 0.169791083 | 0.148088463 | 1142 | tags=42%, list=22%, signal=33% | NRAS/STAT1/MAPK1/SOCS4/GSK3B/PIK3CB/PIK3CA/MAPK3/MAPK14/KRAS/JAK2/FOS/SOS2/FOXO3/SOCS3 |
| hsa04658 | Th1 and Th2 cell differentiation | 50 | 0.381755464 | 1.497518274 | 0.014969002 | 0.169791083 | 0.148088463 | 916 | tags=34%, list=18%, signal=28% | HLA-DPB1/PLCG1/IL2RB/LCK/CD3E/CD247/LAT/HLA-DRB1/CD4/CD3D/RUNX3/CD3G/IKBKB/NFATC2/TBX21/NFATC1/PPP3CC |
| hsa05150 | Staphylococcus aureus infection | 16 | -0.585493892 | -1.684320882 | 0.016091609 | 0.171249723 | 0.14936066 | 644 | tags=56%, list=13%, signal=49% | C5AR1/FPR1/FCGR2A/FPR2/FCGR2C/FCGR3A/C3AR1/FCGR1A/FCGR3B |
| hsa04080 | Neuroactive ligand-receptor interaction | 28 | -0.485369111 | -1.637624204 | 0.016513366 | 0.171249723 | 0.14936066 | 644 | tags=39%, list=13%, signal=35% | C5AR1/FPR1/F2RL1/LPAR2/PGRMC1/CYSLTR1/HRH2/P2RY13/FPR2/C3AR1/ADM |
| hsa05152 | Tuberculosis | 95 | -0.334032185 | -1.436521622 | 0.017501781 | 0.175017815 | 0.152647117 | 583 | tags=31%, list=11%, signal=28% | STAT1/MAPK1/CASP3/NOD2/CLEC7A/ITGAX/PPP3R1/APAF1/PPP3CA/MAPK3/MAPK14/LAMP2/JAK2/FCGR2A/IFNGR1/TLR6/TLR1/CEBPB/RAB5A/CR1/TLR2/BCL10/TLR4/FCGR2C/FCGR3A/IL1B/FCGR1A/FCGR3B/CLEC4E |
| hsa05143 | African trypanosomiasis | 12 | -0.612337729 | -1.651312502 | 0.019788637 | 0.178736073 | 0.155890109 | 969 | tags=58%, list=19%, signal=47% | APOL1/HBA2/F2RL1/GNAQ/HBA1/FAS/IL1B |
| hsa05320 | Autoimmune thyroid disease | 10 | 0.634761277 | 1.61078448 | 0.019668961 | 0.178736073 | 0.155890109 | 289 | tags=40%, list=6%, signal=38% | HLA-DPB1/CD28/HLA-DRB1/PRF1 |
| hsa05330 | Allograft rejection | 10 | 0.634761277 | 1.61078448 | 0.019668961 | 0.178736073 | 0.155890109 | 289 | tags=40%, list=6%, signal=38% | HLA-DPB1/CD28/HLA-DRB1/PRF1 |
| hsa05322 | Systemic lupus erythematosus | 24 | -0.479958377 | -1.560305628 | 0.022254473 | 0.183529385 | 0.160070741 | 598 | tags=42%, list=12%, signal=37% | RO60/H2AC6/H4C15/FCGR2A/H2BC12/H2BC21/FCGR3A/FCGR1A/FCGR3B/H2BC4 |
| hsa01522 | Endocrine resistance | 44 | -0.401143739 | -1.513722086 | 0.021088283 | 0.183529385 | 0.160070741 | 1142 | tags=34%, list=22%, signal=27% | NRAS/MAPK1/BRAF/PIK3CB/PIK3CA/RB1/MAPK3/MAPK14/KRAS/IGF1R/E2F3/FOS/SOS2/MDM2/MMP9 |
| hsa00190 | Oxidative phosphorylation | 71 | 0.343313531 | 1.451880119 | 0.022285711 | 0.183529385 | 0.160070741 | 2043 | tags=59%, list=40%, signal=36% | NDUFB8/NDUFV1/COX4I1/SDHA/ATP5F1A/NDUFA13/COX7C/ATP5MC2/ATP5F1B/COX1/ND6/ATP8/ATP5MC3/COX11/UQCRH/NDUFA10/ATP5MG/COX3/COX2/CYC1/ATP6V1F/UQCR11/COX6A1/ND5/UQCRC1/CYTB/ND4/COX6B1/ATP6/ATP5PO/COX8A/NDUFS2/NDUFB11/ND3/NDUFA9/COX5A/UQCRB/ATP5PB/COX6C/NDUFB4/CYCS/NDUFA4 |
| hsa05146 | Amoebiasis | 31 | -0.453867563 | -1.570925155 | 0.022963628 | 0.183709022 | 0.160227417 | 403 | tags=19%, list=8%, signal=18% | GNAQ/RAB5A/TLR2/TLR4/IL1B/IL1R2 |
| hsa04926 | Relaxin signaling pathway | 50 | -0.402822207 | -1.541642418 | 0.025846208 | 0.191185839 | 0.166748551 | 1188 | tags=38%, list=23%, signal=30% | CREB1/ATF2/NRAS/MAPK1/TGFBR1/PIK3CB/PIK3CA/GNAI3/NFKBIA/MAPK3/MAPK14/MAP2K4/GNG5/KRAS/GNB4/FOS/SOS2/CREB5/MMP9 |
| hsa04920 | Adipocytokine signaling pathway | 33 | -0.438363319 | -1.534707716 | 0.026705573 | 0.191185839 | 0.166748551 | 889 | tags=33%, list=17%, signal=28% | PRKAG2/PRKAA1/ACSL3/NFKBIA/JAK2/ADIPOR1/CHUK/ACSL4/ACSL1/SOCS3/IRS2 |
| hsa05218 | Melanoma | 24 | -0.468678196 | -1.523634676 | 0.025987434 | 0.191185839 | 0.166748551 | 1142 | tags=46%, list=22%, signal=36% | NRAS/MAPK1/BRAF/PIK3CB/PIK3CA/RB1/MAPK3/KRAS/IGF1R/E2F3/MDM2 |
| hsa05164 | Influenza A | 86 | -0.336636558 | -1.430757978 | 0.026516784 | 0.191185839 | 0.166748551 | 1095 | tags=34%, list=21%, signal=27% | STAT1/MAPK1/CASP3/OAS1/PIK3CB/PIK3CA/RNASEL/APAF1/TRIM25/PYCARD/OAS3/NFKBIA/MAPK3/IFNAR1/MX2/JAK2/IFIH1/TNFSF10/IFNGR1/TBK1/CHUK/RIGI/DNAJC3/TLR4/FAS/IL1B/EIF2AK2/SOCS3/RSAD2 |
| hsa05161 | Hepatitis B | 99 | -0.310527966 | -1.344557376 | 0.027312263 | 0.191185839 | 0.166748551 | 1276 | tags=34%, list=25%, signal=26% | CREB1/TAB2/TRAF6/ATF2/IRAK4/NRAS/STAT1/MAPK1/CASP3/BRAF/TGFBR1/PIK3CB/PIK3CA/APAF1/RB1/NFKBIA/MAPK3/MAPK14/MAP2K4/IFNAR1/KRAS/JAK2/IFIH1/TBK1/CHUK/E2F3/RIGI/FOS/SOS2/TLR2/TLR4/FAS/CREB5/MMP9 |
| hsa05219 | Bladder cancer | 20 | -0.508496509 | -1.571946393 | 0.029600617 | 0.199249231 | 0.173781285 | 1257 | tags=60%, list=24%, signal=46% | TYMP/NRAS/MAPK1/BRAF/RB1/MAPK3/KRAS/RPS6KA5/DAPK2/E2F3/MDM2/MMP9 |
| hsa00500 | Starch and sucrose metabolism | 12 | -0.582220832 | -1.570095214 | 0.031310594 | 0.199249231 | 0.173781285 | 490 | tags=42%, list=10%, signal=38% | PGM2/GYG1/PYGL/PGM1/MGAM |
| hsa04657 | IL-17 signaling pathway | 40 | -0.404644356 | -1.481895988 | 0.031193545 | 0.199249231 | 0.173781285 | 1422 | tags=57%, list=28%, signal=42% | FADD/MAPK13/TNFAIP3/TAB2/TRAF6/S100A9/IL17RA/USP25/MAPK1/CASP3/GSK3B/S100A8/NFKBIA/MAPK3/MAPK14/TAB3/TBK1/CHUK/CEBPB/FOS/IL1B/PTGS2/MMP9 |
| hsa05415 | Diabetic cardiomyopathy | 107 | 0.29984184 | 1.374269928 | 0.030320906 | 0.199249231 | 0.173781285 | 2043 | tags=59%, list=40%, signal=36% | PARP1/PRKCA/SMAD3/NDUFB8/NDUFV1/COX4I1/SDHA/ATP5F1A/TBC1D4/NDUFA13/COX7C/PTPA/MTOR/ATP5MC2/SLC25A5/ATP5F1B/COX1/AKT2/ATP2A3/ND6/ATP8/ATP5MC3/UQCRH/NDUFA10/COX3/PIK3R1/COX2/CYC1/ATP2A2/UQCR11/COX6A1/GYS1/ND5/UQCRC1/CYTB/PPP1CA/ND4/PLCB2/COX6B1/GAPDH/ATP6/CYBA/ATP5PO/CYBB/COX8A/RELA/TGFB1/NDUFS2/AKT1/NDUFB11/RAC2/SLC2A1/PDHA1/ND3/NDUFA9/NFKB1/COX5A/UQCRB/ATP5PB/COX6C/NDUFB4/CTSD/NDUFA4 |
| hsa03030 | DNA replication | 11 | 0.609043072 | 1.565133071 | 0.032739539 | 0.201741564 | 0.175955048 | 2017 | tags=100%, list=39%, signal=61% | RPA1/MCM3/MCM7/MCM5/POLE3/POLE/POLD4/RFC1/RNASEH2B/RNASEH1/RPA2 |
| hsa01200 | Carbon metabolism | 47 | 0.374748285 | 1.462988837 | 0.033447393 | 0.201741564 | 0.175955048 | 1323 | tags=43%, list=26%, signal=32% | PFKL/ACSS1/SDHA/SHMT2/ADH5/ENO1/PKM/PFKP/DLST/IDH2/OGDH/HK1/MDH2/H6PD/TPI1/ACO2/PRPS1/GOT2/CS/IDH3B |
| hsa04659 | Th17 cell differentiation | 63 | 0.340645175 | 1.408119206 | 0.033863762 | 0.201741564 | 0.175955048 | 249 | tags=19%, list=5%, signal=18% | HLA-DPB1/PLCG1/IL2RB/LCK/CD3E/CD247/LAT/HLA-DRB1/CD4/HSP90AB1/SMAD3/CD3D |
